# Family structure and phylogenetic analysis of odorant receptor genes in the large yellow croaker (*Larimichthys crocea*)

**DOI:** 10.1186/1471-2148-11-237

**Published:** 2011-08-11

**Authors:** Yingsong Zhou, Xiaojun Yan, Shanliang Xu, Peng Zhu, Xianxing He, Jianxin Liu

**Affiliations:** 1 School of Animal Sciences, Zhejiang University, Kaixuan Road, Hangzhou, China; 2 Key Laboratory of Applied Marine Biotechnology, Ningbo University, Fenghua Road, Ningbo, China

## Abstract

**Background:**

Chemosensory receptors, which are all G-protein-coupled receptors (GPCRs), come in four types: odorant receptors (ORs), vomeronasal receptors, trace-amine associated receptors and formyl peptide receptor-like proteins. The ORs are the most important receptors for detecting a wide range of environmental chemicals in daily life. Most fish OR genes have been identified from genome databases following the completion of the genome sequencing projects of many fishes. However, it remains unclear whether these OR genes from the genome databases are actually expressed in the fish olfactory epithelium. Thus, it is necessary to clone the OR mRNAs directly from the olfactory epithelium and to examine their expression status.

**Results:**

Eighty-nine full-length and 22 partial OR cDNA sequences were isolated from the olfactory epithelium of the large yellow croaker, *Larimichthys crocea*. Bayesian phylogenetic analysis classified the vertebrate OR genes into two types, with several clades within each type, and showed that the *L. crocea *OR genes of each type are more closely related to those of fugu, pufferfish and stickleback than they are to those of medaka, zebrafish and frog. The reconciled tree showed 178 duplications and 129 losses. The evolutionary relationships among OR genes in these fishes accords with their evolutionary history. The fish OR genes have experienced functional divergence, and the different clades of OR genes have evolved different functions. The result of real-time PCR shows that different clades of ORs have distinct expression levels.

**Conclusion:**

We have shown about 100 OR genes to be expressed in the olfactory epithelial tissues of *L. crocea*. The OR genes of modern fishes duplicated from their common ancestor, and were expanded over evolutionary time. The OR genes of *L. crocea *are closely related to those of fugu, pufferfish and stickleback, which is consistent with its evolutionary position. The different expression levels of OR genes of large yellow croaker may suggest varying roles of ORs in olfactory function.

## Background

Vertebrates can distinguish numerous odorants in the environment using chemosensory receptors that are expressed in the olfactory epithelium [1-3]. Four types of chemosensory receptors have been found in the vertebrate olfactory epithelium, including the main odorant receptors (ORs) [4], vomeronasal receptors (VRs) [5-7], trace-amine associated receptors (TAARs) [8] and formyl peptide receptor-like proteins [9]. The OR genes were initially identified in mouse olfactory organ by Linda Buck and Richard Axel [4], who found that each olfactory sensory neuron expressed a single OR allele [4,10,11]. The ORs, located on the surface of dendrites of sensory neurons on the olfactory epithelia, are the most important chemosensory receptors in the detection and perception of common odorants in the environment. Two types of VRs (V1R and V2R) that are located in the vomeronasal organ in mammals are mainly responsible for detection of pheromones. In fishes, ORs and VRs are both distributed in the one olfactory organ (there is no vomeronasal organ) [12,13]. The TAARs, expressed in the olfactory epithelium, are responsible for recognition of trace amines and related compounds [8,14]. The formyl peptide receptor-like proteins that are found in the vomeronasal organs in mammals have an olfactory function associated with the identification of pathogenic states.

The ORs are the most important chemosensory receptors in detecting environmental chemicals in daily life, and they detect a wide range of compounds. A large number of OR genes have now been isolated from various species. Approximately 1,068 putative functional OR genes and ~334 pseudogenes in mouse [15,16], ~340 putative functional OR genes and ~388 pseudogenes in human [17-19] and ~100 OR genes in fishes [20-22] have been identified from genome databases. The vertebrate OR genes have recently been divided into two major types, type 1 and type 2. The type 1 genes were subdivided into five groups, α, β, γ, δ, ε and ζ, and the type 2 genes into four groups, η, θ, κ and λ, but the groups θ, κ and λ are considered to likely be non-OR genes because they were identified from genome databases and found not to be expressed in the olfactory epithelium [20,21]. Mammalian OR genes are clearly classified into class I and class II [23]; here, groups α and β correspond to class I and the group γ to class II [20,21].

Currently, the most fish OR genes have been identified from genome databases. However, we do not know whether these genes are really expressed in the fish olfactory epithelia. So far, few experiments have been carried out to validate the expression status of OR genes in the fish olfactory epithelium. In addition, it is necessary to expand the knowledge of fish ORs, especially for marine fishes, as the teleost fishes from which OR genes have been reported so far are mostly not strict marine fishes but freshwater, brackish or amphidromous.

The large yellow croaker (*Larimichthys crocea*), an economically important fish in China, belongs to the family Sciaenidae of the order Perciformes and dwells on the coast of the temperate zone in China. To clarify the evolution of the *L. crocea *OR genes and to discover whether they are expressed in the olfactory epithelium, we cloned OR cDNAs from large yellow croaker by RT-PCR on the olfactory organ and isolated full-length cDNA using rapid amplification of cDNA ends (RACE). We then conducted phylogenetic analysis using these OR genes and others from 11 vertebrate species and determined the expression levels of the different subfamilies in wild-type fishes using quantitative real-time PCR.

## Results

### The number of OR genes in *L. crocea*

**Figure 1:**
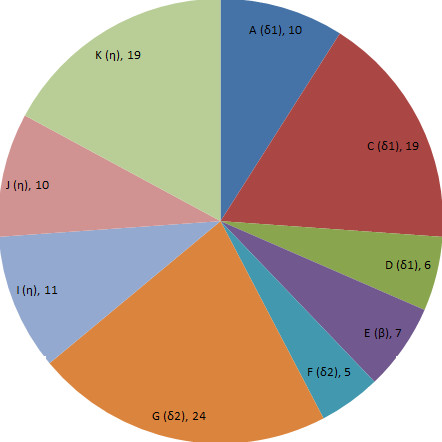
**Numbers of OR genes obtained from the large yellow croaker**. The relative sizes of the nine OR subfamilies are shown, with numbers of genes and group names in parentheses.

cDNAs corresponding to 111 OR genes were isolated from the olfactory epithelium, including 89 full-length and 22 partial cDNAs. Figure 1 shows the number of OR genes belonging to each subgroup. A disruption was found in two sequences in comparison with the complete cDNA sequence obtained at the same time from *L. crocea *olfactory epithelium. One sequence had lost a start codon and another had lost a few nucleotides in the region of the 5' primer, but the open reading frame of the both sequences was not shifted (see additional file 1).

In teleost fishes, the numbers of OR genes are highly variable. Table 1 shows the numbers of functional OR genes from six fishes [20]. The zebrafish has the largest number of functional OR genes (~154), and the pufferfish has the least (only 11). However, an accurate number of OR genes in different fishes remains unknown, because some of the sequences in the genome database of distinct species may be either intact or not completely included because of the limitation of sequencing technology. *L. crocea *contains about 111 OR genes that are expressed in the olfactory epithelium.

**Table 1:** Numbers of OR genes in six fishes

Common name	Species name	Functional
Large yellow croaker	*Larimichthys crocea*	111
Zebrafish	*Danio rerio*	154
Medaka	*Oryzias latipes*	68
Stickleback	*Gasterosteus aculeatus*	102
Fugu	*Takifugu rubripes*	47
Pufferfish	*Tetraodon nigroviridis*	11

A functional gene is a sequence that does not contain nonsense or frame shift mutation or long deletions and has initiation and stop codons at the proper positions. All the large yellow croaker OR genes are inferred from cDNA amplified from mRNA in the olfactory epithelium. Therefore, they are considered as functional genes whether full-length or partial cDNA.

### Phylogenetic analysis

**Figure 2:**
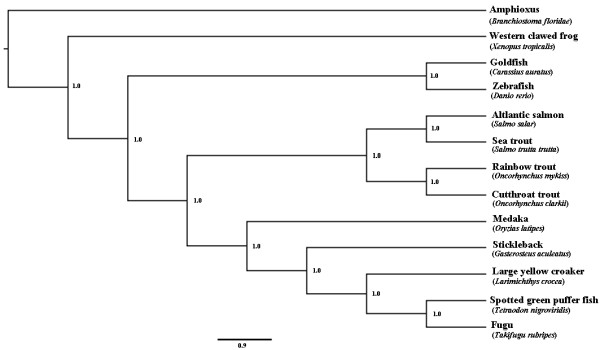
**Evolutionary history of fishes based on mitochondrial proteins**. Phylogenetic tree of 11 fishes and one amphibian using Bayesian analysis of amino acid sequences of 12 concatenated protein-coding mitochondrial genes (omitting ND6). Amphioxus is used as an outgroup. The posterior probabilities are given for each node. The scale bar represents 0.9 substitutions.

A species tree was built using mitochondrial genomes to elucidate the evolutionary relationships among 12 species, including 11 fishes and 1 amphibian (Figure 2). The species tree was in an excellent agreement with a previous study [24]. Amphioxus [25], which diverged from the vertebrates perhaps 550 million years ago (MYA), was used to root the tree. Figure 2 clearly shows the evolutionary processes among the 12 species. The large yellow croaker is more closely related to fugu, pufferfish and stickleback than it is to medaka, salmon, trout, zebrafish and goldfish.

**Figure 3:**
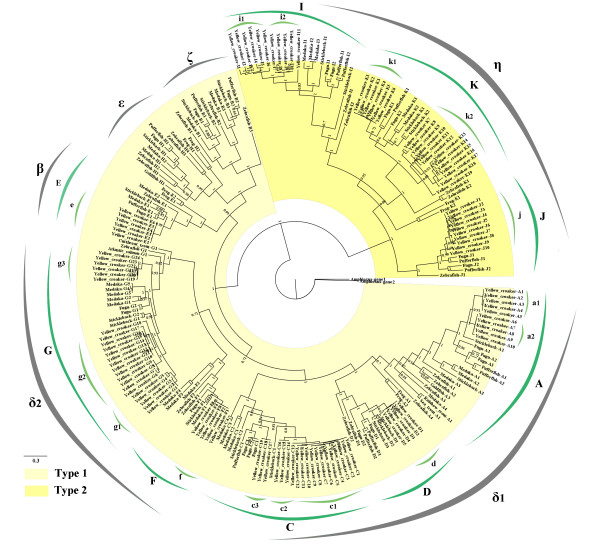
**Phylogenetic relationships of fish OR genes**. The Bayesian phylogenetic tree was constructed using Mrbayes 3.1.2. Representative type 1 and type 2 vertebrate OR genes are shown, including 109 *Larimichthys crocea *OR genes and 121 relevant OR genes from other eleven vertebrates. Two sequences from amphioxus are used as an outgroup. The posterior probabilities are given for each node in the tree. The scale bar represents 0.3 substitutions. The light and dark yellow shading in the circular tree represent type 1 and 2 genes, respectively. The OR gene group names are shown on the outside of each clade.

An OR gene tree was constructed using the relevant OR genes from large yellow croaker and 11 other species (frog, zebrafish, goldfish, Atlantic salmon, cutthroat trout, sea trout, rainbow trout, medaka, stickleback, pufferfish and fugu; Figure 3). Two representative OR genes from amphioxus that are highly divergent from vertebrate OR genes were used to root the tree [20,26]. The phylogeny shows the relationships between the species, with branch lengths proportional to the number of expected substitutions per amino acid site. From this analysis and the criteria set forth by previous studies [20,21], the OR genes from these species could be classified into two major groups, the type 1 and type 2 genes (Figure 3). The type 1 genes are subdivided into four groups, β, δ, ε and ζ, and the group δ is further split into two subgroups (δ_1 _and δ_2_). Groups α and γ of type 1, which are present in amphibians, reptiles, birds and mammals and absent in fish except for one intact gene in zebrafish and a few pseudogenes in medaka and stickleback, were not included in the phylogenetic analysis. Type 2 only contains group η in this study. Thus, five groups (β, δ, ε, ζ and η) were included in the phylogenetic tree. Five clades (A, C, D, F and G) of *L. crocea *OR genes were assigned to group δ (δ_1 _and δ_2_), three clades (I, J, K) were assigned to group η, and clade E was assigned in the group β.

**Figure 4:**
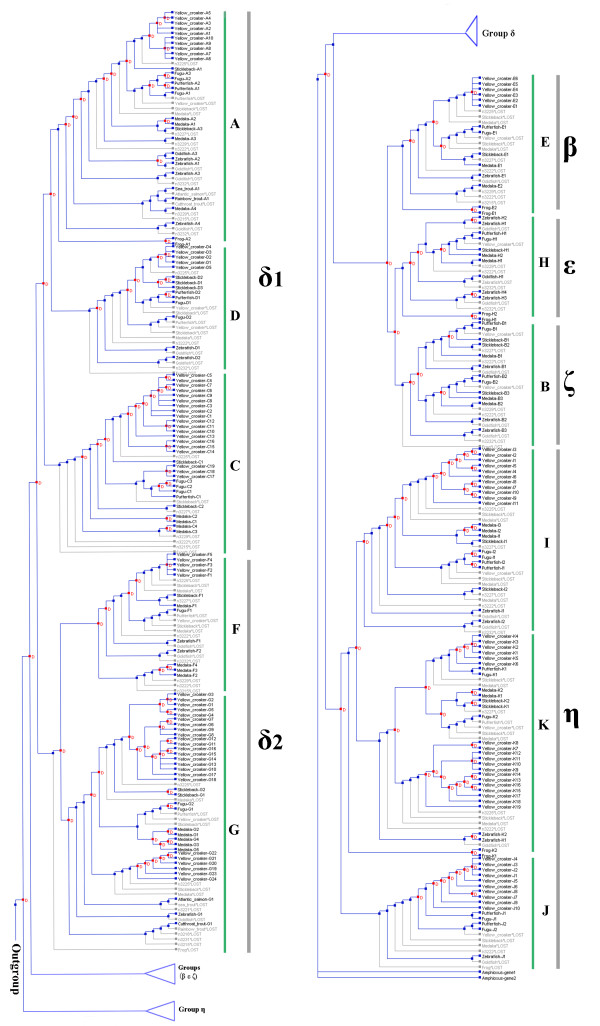
**Reconciled tree of the OR gene and species trees constructed using Notung 2.6**. Gray branches represent gene loss, and D on a node represents duplication.

To further analyze the evolutionary processes and infer the OR gene duplications and losses among these different fishes, a reconciled tree was constructed using Notung 2.6 [27,28] based on the above gene and species trees (Figure 4). The tree shows 178 duplications and 129 losses. Some lost OR genes from unknown species were inferred, and these species were not included in this study owing to the limitations of the current OR gene database.

From the above phylogenetic analysis, we can see that the *L. crocea *OR genes are more closely related to those of fugu, pufferfish and stickleback than they are to medaka, zebrafish and frog (Figures 3, 4). The evolutionary pattern of the OR genes thus corresponds to the phylogenetic tree based on mitochondrial genome sequences. Each subfamily of OR genes evolved from common ancestral genes and was expanded in extant species. Many genes have duplicated, and many have been lost.

### Functional divergence among fish OR genes

**Figure 5:**
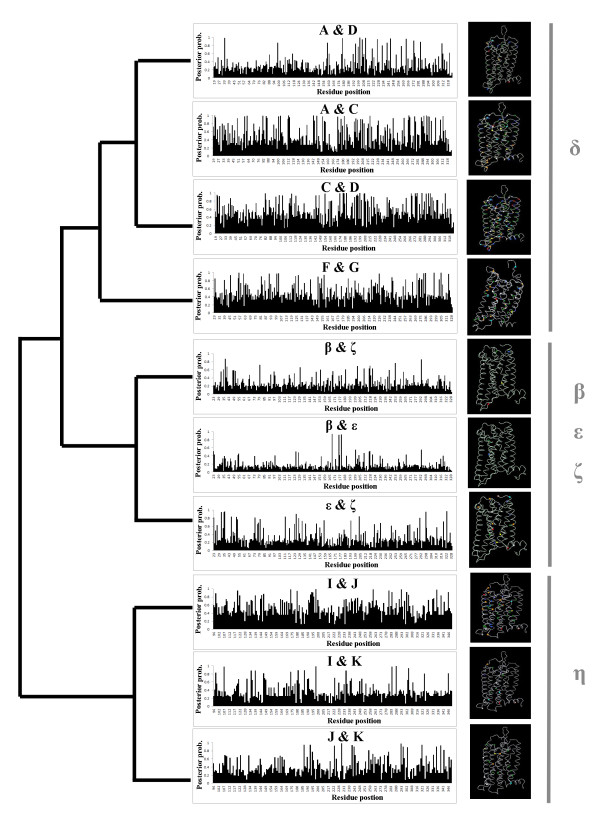
**Functional divergence of different clades of OR genes**. The posterior probability for predicting critical amino acid residues responsible for the functional divergence in pairwise comparison within the different groups is plotted against residue position, and the residues where the posterior probability of the site-specific functional shift is larger than 0.7 are highlighted using arbitrary colors on the three-dimensional structure. The structure prediction was made using CPHmodels 3.0 [52].

Gu [29-31] has developed a statistical method to test the significance of Type I and Type II functional divergence between duplicate genes. Type I divergence results in site-specific rate shifts after gene duplication, and Type II divergence results in site-specific property (hydrophobicity and hydrophilicity) shifts. We used the software DIVERGE 2.0, which uses this method [32-34], to analyze the inferred OR protein sequences, and to infer the functional divergence of OR proteins and to predict the amino acid site changes involved in functional divergence among sequences. The results indicate that the functional divergence among subgroups of odorant receptors is of Type I (Table 2). The coefficient of Type I functional divergence between duplicate genes, denoted θ_ML_, is defined as the probability of functional divergence. A large value of θ_ML _indicates a high level of Type I functional divergence, and vice versa. Figure 5 shows posterior probabilities of functional variation of amino acid residues. The variation sites with posterior probability larger than 0.7 are highlighted in the three-dimensional structure of an odorant receptor shown.

**Table 2:** Statistics of functional divergence among different clades

		Group δ			Group (β, ε and ζ)			Group η		
	
Statistic	A/D	C/D	A/C	F/G	β/ε	β/ζ	ε/ζ	I/K	I/J	K/J
θ_ML_	0.31	0.54	0.48	0.49	0.27	0.17	0.31	0.37	0.51	0.40
SE θ_ML_	0.06	0.06	0.06	0.07	0.08	0.07	0.06	0.08	0.08	0.08

The statistical results represent functional divergence among different clades in each group. θ_ML _represents the coefficient of Type I functional divergence; SE θ_ML _is the standard deviation of θ_ML_.

The analysis of group δ_1 _ORs suggests that the level of functional divergence between clades C and D (θ_ML _= 0.54) is higher than that between clades C and A (θ_ML _= 0.48), and the functional divergence between clades A and D is the lowest in the pairwise comparisons (θ_ML _= 0.31). The value of θ_ML _between clades F and G in δ_2 _is 0.49, which means that these two groups of genes have evolved different functions. Only one clade of OR genes was found in the group β, and it was clustered with the group ζ and ε in the phylogenetic analysis (Figure 3). The level of functional divergence between groups β and ζ (θ_ML_= 0.27) is higher than that between groups β and ε (θ_ML_= 0.17), but the functional divergence between groups ζ and ε is the highest (θ_ML_= 0.31) among these three groups.

The type 2 genes are more diverse than type 1. Three clades of OR genes in group η were included in this study. These clades were analyzed in pairwise comparison to predict the functional divergence. The results showed that the θ_ML _values generated by pairwise comparison among the three clades were 0.37 (I/K), 0.51 (I/J) and 0.40 (K/J) (Table 2). This suggests that all three clades have experienced functional divergence and have evolved different functions from their ancestors.

However, the sequences in the same clade were found to be highly conserved. The OR genes from different species in the same clade may perform similar functions, for example, detecting similar chemicals.

### Expression levels of OR genes in the *L. crocea *olfactory epithelium

Sixteen pairs of primers were designed according to the sequences in each clade (A*-*K) to detect the expression levels of OR genes using quantitative real-time PCR. To evaluate whether each pair of primers conform to the efficiencies within the range 0.9-1.1, their efficiencies were examined by establishing standard curves based on regression analyses of the Ct (Cycle threshold) value versus the log value of 10-times dilution of each target gene (Copies) for each pair of primers (see additional file 2). The primers that had efficiencies within the range 0.9-1.1 were used in quantitative real-time PCR.

**Figure 6:**
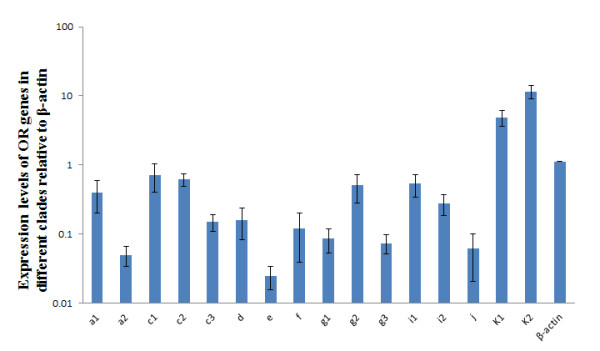
**OR genes expression levels in the *L. crocea *olfactory epithelium**. The expression levels of OR genes from different clades were determined by quantitative real-time PCR. Expression levels relative to the control gene β-actin were calculated using the standard curve method and are reported on a log_10 _scale. The letters *a*-*k *indicate clades.

The average Ct values of each gene expression obtained by real-time PCR are shown in additional file 3. The mRNA copies were calculated according to the Ct values and the standard curves. Expression of each target gene was normalized to the housekeeping control gene β-actin. The results showed that the different clades of OR genes have distinct expression levels (Figure 6). Comparisons between groups were tested by one-way analysis of variance (ANOVA). The OR genes in clade K (*k_1 _*and *k_2_*) in group η were expressed at the highest levels of all groups (*P *< 0.001), followed by the clade *c*_*1*_, *c*_*2 *_and *g*_*2 *_within group δ, and then clade i_1 _and i_2 _within group η in decreasing order, but no significant differences were found between each other. The expression levels of genes in clades *a_2_*, *c_3_*, *d*, *f*, *g_1 _*and *g_3 _*were similar (*P *> 0.05), but they were significantly lower than those of the above clades (*P *< 0.05). The expression levels of OR genes in clade *a_1 _*is twice as high as that in clade *a_2 _*(*P *< 0.01). The OR genes in clade *e *within group β were expressed at the lowest level among all groups (*P *< 0.001). These results suggest that the OR genes in the clade K are expressed at the highest level in the olfactory epithelium, and OR genes in clade *e *were expressed at the lowest level.

## Discussion

### Variable numbers of fish OR genes

The numbers of OR genes in teleost fishes are highly variable: zebrafish has the largest number of functional OR genes of all the fishes, the large yellow croaker and stickleback have ~100 OR genes, fugu has ~50 and the pufferfish has ~11. From these data, we can see the numbers of OR genes vary 2- to 10-fold among these teleost fishes. The numbers of tetrapod OR genes are much higher; for example, ~1000 functional OR genes were identified from the genome in mouse, ~800 in frog and ~340 in human. Most mammalian OR genes in group α and group γ are thought to be airborne substance receptors [20,21]. The conservation of OR genes in mammals is higher than that in fishes [22], and it may be that the mammals evolved more OR genes to adapt to the environment on the land [20].

### The evolutionary pattern of fish OR genes

OR genes originated at a very early time in chordate evolution, before appearance of amphioxus, which diverged from its most recent common ancestor with vertebrates 550 MYA [25,26]. The vertebrate OR genes have evolved into different families and subfamilies from their ancestor. The type 1 and type 2 genes diverged before the divergence of jawless and jawed vertebrates. The seven groups (α, β, γ, δ, ε, ζ and η) diverged at around the separation of the jawless and jawed fishes. The tetrapods evolved groups α and γ, which were absent in fishes, but they lost most of fish-like groups (β, δ, ε, ζ and η) except in amphibians [20]. We have clarified the evolutionary relationship of OR genes among modern teleost fishes using phylogenetic analysis. The results are consistent with previous reports [14,20-22,32]. The OR genes of extant teleost fish evolved from the common ancestor of Actinopterygii (ray finned fish) [22]. The OR gene relationships among extant teleost fishes fit with their evolutionary history of the fishes. The large yellow croaker, fugu, pufferfish and stickleback are close evolutionary relatives, and thus their OR genes are more closely related to each other than to those of other fishes in this study. Modern teleosts have inherited the OR genes and gradually expanded genes from their common ancestors in the process of speciation and duplication. However, in the process of gene duplication, some genes were lost, as shown in the reconciled tree (Figure 4). Some OR genes have developed species-specific functions [33], but others have been lost and pseudogenized. For example, about 10-55% of OR genes in zebrafish, medaka, stickleback, fugu and pufferfish have become pseudogenes [20]. Half the human OR genes have become pseudogenes and lost their function [17-19,34].

In summary, extant teleost fishes inherited OR genes from their common ancestors during speciation and duplication. Some OR genes have been lost or pseudogenized during evolution. The fishes with close evolutionary relationships show a closer relationship between their OR genes.

### Functional divergence among OR families

Functional divergence is the process by which genes, after gene duplication, shift in function from an ancestral function. It is thought that this process of gene duplication and functional divergence is a major originator of molecular novelty and has produced many large protein families that exist today [29,35,36]. Because of this, it is desirable, from sequence analysis, to identify amino acid sites that are responsible for functional diversity. It is important to know the level of functional divergence after gene (or genome) duplication, as well as how many amino acid substitutions are involved in functional innovations [29]. The OR genes have been greatly expanded from their ancestral genes, and have developed into one of the largest multi-gene families in the vertebrates. The results of evolutionary divergence analysis indicate that all subfamilies of OR genes have functionally diverged from their most recent common ancestors, and they have evolved new functions, because the coefficient of functional divergence (θ_ML_) between each pair of clades is significantly larger than 0. However, whether or not the different clades of OR genes have different functions needs to be verified by future experiments. Site-specific divergence can occur at some amino acid residues (Figure 5). We suggest that the amino acid sites (highlighted in the three-dimensional structure in Figure 5) that show a posterior probability of a functional shift of over 0.7 were the key sites involved in functional divergence between each pair of clades.

### OR gene expression and functions

We have shown that ~100 OR genes are expressed in the *L. crocea *olfactory epithelium. A previous study found that only a small proportion of the mouse OR genes are expressed in the olfactory epithelium, and the remaining OR genes are transcriptionally inactive [37]. Therefore, we inferred that many *L. crocea *OR genes were not transcriptionally active, and that this is why they were not isolated from the epithelium using RT-PCR and RACE. For example, we do not find the groups ε and ζ in *L. crocea*, even though they are present in the other fish OR gene families. However, we plan to confirm this inference when genomic sequence information is available. The different clades of OR genes are expressed at different levels. The expression levels of OR genes in the olfactory epithelium appears to control the likelihood that a cDNA will be cloned [37]. The higher the expression level of an OR gene, the easier it will be to isolate it from the epithelium. We found that two sequences, each with one disruption, are still transcribed in the *L. crocea *olfactory epithelium and inferred that these two sequences may have lost their functions. In a previous study, Zhang and Firestein [15] found, from the available expression data for mouse OR genes [38] and the National Center for Biotechnology Information (NCBI) mouse expressed sequence tag (EST) database, that some of the mouse OR sequences identified from genome databases with one or two disruptions were still expressed in the olfactory organ. Other researchers [37] found that a few pseudogenes were also expressed in the mouse olfactory epithelium.

In previous studies, many researchers have shown expression of teleost OR genes in the olfactory organ using *in situ *hybridization [32,39]. More than 419 OR genes were found to be expressed in the mouse olfactory epithelium, and some OR genes were expressed at significantly higher levels than others [37]. Similarly, we found in the large yellow croaker that different OR genes have different expression levels; for example, clades K (*k_1 _*and *k_2_*), *c_1_*, *c_2 _*and *g_2 _*were shown to be expressed at high levels. However, whether these genes have essential olfactory functions in *L. crocea *life needs to be further verified. The previous report showed that the monoallelic OR expression was achieved through a mechanism in which OR protein functions in olfactory neurons to abrogate expression of other OR genes [40]. Data from a number of previous studies also showed that different OR genes, or even copies of the same OR transgene in different genomic locations, are expressed in different numbers of cells [41-43], but these studies did not address the issue of transcript levels per cell. The fact that some OR genes are frequently chosen to be expressed, and when chosen are expressed at high levels per cell, is intriguing given each olfactory neuron's single-allele expression regime [37]. The transcription level of OR genes is associated with control regions in the genome locus. McIntyre *et al*. [44] found that homeobox transcript factors such as Emx2 stimulated the expression levels of OR genes in mouse, but it was not certain whether the rule would also apply to fishes. The regulation of OR gene expression is intriguing, and it will be important to clarify the mechanism of gene expression regulation. Whether OR gene expression is correlated with the location of the genes in the genome and what the regulatory mechanism of OR gene expression is in the fish olfactory system are questions awaiting further study. However, it is most important that the expression of OR genes in fish must maintain the basic abilities to survive in the environment.

The olfactory system uses a combinatorial receptor coding scheme to encode odor identities [11]. Each family of OR genes is mainly responsible for detecting one class of similar chemicals. Many studies of OR genes in human and mouse have revealed that one OR can be activated by various chemicals, and OR genes in the same subfamily might distinguish a similar class of substance or a structural feature in substances [4,11,17]. Fish live in the water and frequently encounter water-soluble substances, and most ORs detect water-soluble substances [39,45,46]. However, the specificity between receptors and ligands has not been extensively reported in fish. The question of whether or not each subfamily of receptors is mainly responsible for detecting a specific array of chemicals in fish awaits an answer.

## Conclusions

We have shown that about 100 OR genes are expressed in olfactory epithelium of large yellow croaker. The evolutionary relationships among teleost fish ORs accords with the evolutionary history of the fishes themselves. The OR genes of modern fishes duplicated from their common ancestor and have been expanded during evolution. The *L. crocea *OR genes are more closely related to those of stickleback, fugu and pufferfish than they are to those of medaka, zebrafish and western clawed frog. The different expression levels of the OR genes of the large yellow croaker suggest different roles in olfactory function.

## Methods

### Sample collection

One-year-old large yellow croakers (*Larimichthys crocea*) were collected from Xiangshan Bay in Zhejiang province of China. The fish olfactory organs were dissected and preserved in RNAlater reagent (Ambion, Texas, USA), and stored at -20°C until use.

### Total RNA extraction and first-strand cDNA synthesis

Total RNA was extracted and purified from approximately 100 mg olfactory epithelium tissue using Trizol reagent (Invitrogen, California, USA), and was then treated with DNase I (0.1 unit per μg RNA) (Takara, Otsu, Japan) to remove the residual DNA. The RNA quality and quantity were determined using a NanoDrop ND-1000 Spectrophotometer (NanoDrop Technologies Inc., Wilmington, DE, USA). First-strand cDNA synthesis was performed using a PrimeScript RT-PCR Kit (Takara, Otsu, Japan) following the user manual.

### Amplification of OR genes

Nine pairs of primers for RT-PCR were designed according to the conserved domain of fugu, pufferfish and zebrafish OR genes (Table 3) to amplify cDNA that had been reversely transcribed from mRNA isolated from the olfactory epithelium. Amplifications were optimized in 50 μl reaction volumes containing about 50 ng cDNA using a PrimeScript RT-PCR Kit (Takara, Otsu, Japan). The annealing temperature is different for each pair of primers. The PCR reaction condition were as follows: initial denaturation at 95°C for 5 min, then 30 cycles of 95°C for 30 s, 37-50°C for 35 s and 72°C for 47s, finally followed by an extension step at 72°C for 10 min.

**Table 3:** Primers designed for RT-PCR for amplification of each group of OR genes

Target gene	Group	Primer set 5'-3'
ORA	δ1	F:GGCTATATGTCAACCTCTG
		R:TATACCATAGATTAAAGGATT
ORC	δ1	F:TGTACGTTCTGATTGCAGC
		R:TCAGATGTGGTAAACAGGT
ORD	δ1	F:GTACGGCTCTGCGGGCTTCT
		R:GGAGGAATCACAACAAACTC
ORE	β	F:ATGTATTTTGTTCACTTTTTAGG
		R:AGGCATGTAATACAGACACGTGA
ORF	δ2	F:GATCGTTATGTTGCCATCTG
		R:GAGAAATATCAGCATGCCCA
ORG	δ2	F:CCTCCCTCTGAACGCCTCAT
		R:CTGCAGGTCTTCAAGGCTTT
ORI	η	F:TGTCTTTGGAGAGGTATGTA
		R:GCACTGAGACATCTGGGAAG
ORJ	η	F:ACACGCCTCTGAACCTGGCC
		R:ATGATGGTGTTTCTGGCCTT
ORK	η	F:GAGCGGTACGTGGCCATTTG
		R:AGACCGTAGATGAGAGGACT
β-actin		F:ATGGAAGATGAAATCGCCGC
		R:TGCCAGATCTTCTCCATGTCG

Nine pairs of primers that could amplify OR genes are shown. The primers were designed according to the conserved domain of fugu, pufferfish and zebrafish OR genes. The primers were named according to the subgroup that they amplify.

### Obtaining full-length cDNA

Full-length cDNA of odorant receptor genes was obtained through 3' and 5' rapid amplification of cDNA ends (RACE). Nested PCR was used in RACE, and the appropriate annealing temperature was chosen for each pair of primers. The outer and inner primers for RACE were designed by sequences obtained by RT-PCR. When obtaining the sequences from results of the previous RACE, the primers that were located closer to the 5' or 3' end of cDNA were designed to obtain longer sequences and increase the sequencing accuracy. RACE was carried out using a smarter RACE cDNA amplification kit (Takara Bio, USA) and a 3' full RACE core kit (Takara, Otsu, Japan) according to the manufacturers' instructions.

### Cloning and sequencing of PCR products

PCR products were excised from agarose gels and purified using agarose gel DNA purification kit (Takara, Otsu, Japan) before ligation to the PMD-18T vector (Takara, Otsu, Japan) and transformation into *Escherichia coli *strain DH-5α (GIBCO/BRL, USA). More than 30 inserted clones identified by blue/white selection in each plate were sequenced. DNA sequencing was supplied by Invitrogen. Each of the sequences obtained from RT-PCR and RACE was translated into protein sequence, and then used to search with BLASTP [47] against the NCBI non-redundant database. When the best hit of a given query was an OR gene from another species, we considered the query sequence as an OR gene.

### Phylogenetic analysis of mitochondrial proteins from 12 species

The mitochondrial complete genome sequences were retrieved from GenBank. The accession numbers are given in additional file 4. Twelve proteins expressed in the mitochondrion from each species, concatenated into one long sequence as a data set (3,639 amino acids), were aligned using ClustalW [48] (see additional file 5). The NADH dehydrogenase subunit 6 gene was not used in the analysis because of their heterogeneous base composition and consistently poor phylogenetic performance [49]. The resulting alignments were analyzed using Mrbayes 3.1.2 under the Mtmam model [50] with gamma (G) distribution. The Markov chain reached a stationary distribution after 1,000,000 generations with a tree sampled every 100 generations in two runs. The first 2,500 trees (25%) were discarded as 'burn-in' in each run. The consensus tree was then built and the posterior probabilities of the tree and its branches were calculated based on 15,000 pooled trees.

### Phylogenetic analysis of OR gene families

The amino acid sequences of functional OR genes, including 109 from *L. crocea *and 121 related OR genes from one amphibian and 11 ray-finned fish (accession numbers in additional file 4) were aligned using ClustalW [48]. The N- and C-termini of aligned sequences was removed by hand (see additional files 6 and 7). Two OR sequences of *L. crocea *were removed from the adjusted alignment because they were found to be the same as two other sequences after removal of the N- and C-termini. The resulting alignments were analyzed using Mrbayes 3.1.2 [51] under the Poisson model of sequence evolution for 10,000,000 cycles with a tree sampled every 1,000 generations in two runs. The parameters set for the consensus tree and the posterior probabilities of the phylogeny and its branches were as same as in the construction of the mitochondrial tree based on the 15,000 pooled trees from the two runs for the two datasets.

### Functional divergence analysis among specific groups

The functional divergence between specific clades was analyzed using DIVERGE 2.0 [36], which can predict the functional divergence of a protein family based on maximum likelihood and posterior probabilities. The closely related subfamilies of OR genes based on phylogenetic analysis were clustered together, and the full-length protein sequences were aligned using ClustalW. The results were used to analyze the functional divergence between each pair of clades and to infer the functional variation of amino acid sites among the distinct OR subfamilies using the method of Gu [29].

### Quantification of OR gene expression

The quantification of *L. crocea *OR genes expression was assayed using a real-time PCR instrument Mastercycler^® ^ep realplex (Eppendorf, Germany) with a SYBR Premix Ex Taq™ II kit (Takara, Japan). The mRNA extracted from the olfactory epithelia of large yellow croakers (*n *= 8) was assayed for OR gene expression. Sixteen pairs of primers representing the different subgroups of OR genes in the phylogenetic tree and one pair of β-actin primers as a reference were designed as shown in additional file 2. To assess the efficiency of amplification, standard curves were constructed using regression analyses of the Ct value versus the log value of 10-times dilution of each target gene for each pair of primers. The initial quantities of each OR gene for building the standard curve were determined using a NanoDrop ND-1000 Spectrophotometer (NanoDrop Technologies, Inc., Wilmington, DE, USA). Three steps of real-time PCR were adopted. Briefly, denaturation at 95°C for 20 s, then 40 cycles of 95°C for 10 s, 59°C for 18 s, 72°C for 20 s. The number of copies of each OR gene were calculated according to the standard curve (see additional file 2). The relative expression levels of OR genes were normalized by the ratio of the copies of OR genes divided by the copies of β-actin, then the ratios were log_10 _transformed. The relative expression levels (log_10 _transformed) of OR genes were analyzed using one-way ANOVA.

### GenBank accession numbers

All the *L. crocea *OR genes described in this study have been deposited in GenBank under the accession numbers: HQ424579-HQ424651 and HQ424653-HQ424690.

## Authors' contributions

YSZ and XJY conceived and designed this study. YSZ performed the experiments and analyzed the data. Both YSZ and XJY contributed to the writing of the manuscript and approved the final manuscript. All the other authors assisted with the sample collection and approved the final manuscript.

## Supplementary Material

Additional file 1 **Two sequences with one disruption aligned with an intact sequence obtained at the same time are shown in this file**. Click here for file

Additional file 2 **The primers for quantitative real-time PCR and standard curves for examining the efficiencies of each pair of primers are included in this file**. Click here for file

Additional file 3 **The average Ct value of each gene amplification is included in this file**. Click here for file

Additional file 4 **A list of the mitochondrial genes and OR genes of the species used in the phylogenetic analysis is included in this file, together with GenBank accession numbers and retrieval address**. Click here for file

Additional file 5 **A multiple sequence alignment of 12 concatenated mitochondrial proteins from 12 organisms, including amphioxus, one amphibian, and 11 ray-finned fishes, are shown in the file**. Click here for file

Additional file 6 **The result of a multiple sequence alignment of 230 OR genes from different species is included in this file**. Click here for file

Additional file 7 **The result of a multiple sequence alignment of 230 OR genes from different species after manual adjustment is included in this file**. Click here for file
