# Supplementary material for: Family structure and phylogenetic analysis of odorant receptor genes in the large yellow croaker (Larimichthys crocea)
Source: BMC Evol Biol. 2011 Aug 11;11:237. doi: 10.1186/1471-2148-11-237 (PMC3162931; doi:10.1186/1471-2148-11-237)
Supplement: Additional file 1 — Two sequences with one disruption aligned with an intact sequence obtained at the same time are shown in this file. [file 1471-2148-11-237-S1.PDF]

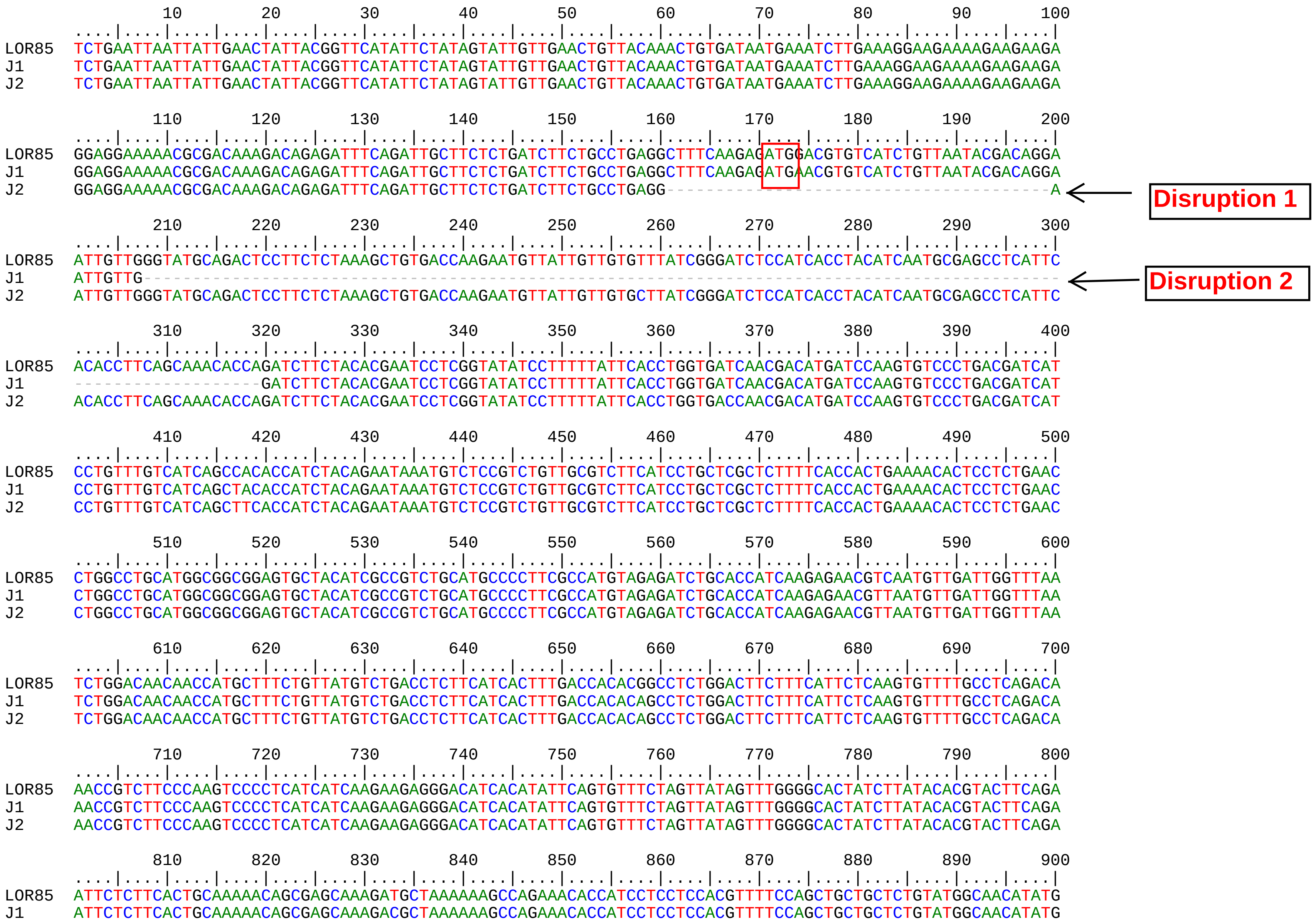
LOR85
J1
J2
Disruption 1
Disruption 2

```
J2      ATTCTCTTCACTGCAAAAACAGCGAGCAAAGATGCTAAAAAAGCCAGAAACACCATCCTCCTTCACGTTTTCCAGCTGCTGCTCTGTATGGCAACATATG

                 910       920       930       940       950       960       970       980       990       1000
        ....|....|....|....|....|....|....|....|....|....|....|....|....|....|....|....|....|....|....|....|
LOR85   TAGCCCCCCAGTTATTAGACATCCTGCAGCAATGGTTCCCTAAGAATCGTACAGACTCTCTCTTTGCTCACTATATCATTGTACAAATCCTGCCGCGATC
J1      TAGCCCCCCAGTTATTAGACATCCTGCAGCAATGGTTCCCTAAGAATCGTACAGACTCTCTCTTTGCTTACTATATCATTGTACAAATCCTGCCGCGATC
J2      TAGCCCCCCAGTTATTAGACATCCTGCAGCAATGGTTCCCTAAGAATCGTACAGACTCTCTCTTTGCTTACTATATCATTGTACAAATCCTGCCGCGATC

                1010      1020      1030      1040      1050      1060      1070      1080      1090      1100
        ....|....|....|....|....|....|....|....|....|....|....|....|....|....|....|....|....|....|....|....|
LOR85   TGTTAGTCCAATCATCTACGGAATACGAGACAATACTTTCAGGAAGTACTTGAAAAGGTATCTGTTTTGTAAAGACTCCATACTGTAAGCGACCATTGAA
J1      TGTTAGTCCAATCATCTACGGAATACGAGACAATACTTTCAGGAAGTACTTGAAAAGGTATCTGTTTTGTAAAGACTCCATACTGTAAGCGACCATTGAA
J2      TGTTAGTCCAATCATCTACGGAATACGAGACAATACTTTCAGGAAGTACTTGAAAAGGTATCTGTTTTGTAAAGACTCCATACTGTAAGCGACCATTGAA

                1110      1120      1130      1140      1150
        ....|....|....|....|....|....|....|....|....|....|....
LOR85   AACTTTCCTGAGAAGGTTTTTTCCAATAAAAGGAGAATTAAAAGAAATAAAAAA
J1      AACTTTCCTGAGAAGGTTTTTTCCAATAAAAGGAGAATTAAAAGAAATAAAAAA
J2      AACTTTACTGAGAAGTTTTTTTCCAATAAAAGGAGAATTAAAAGAAAAAAAAAA
```
