# Supplementary material for: Family structure and phylogenetic analysis of odorant receptor genes in the large yellow croaker (Larimichthys crocea)
Source: BMC Evol Biol. 2011 Aug 11;11:237. doi: 10.1186/1471-2148-11-237 (PMC3162931; doi:10.1186/1471-2148-11-237)
Supplement: Additional file 2 — The primers for quantitative real-time PCR and standard curves for examining the efficiencies of each pair of primers are included in this file. [file 1471-2148-11-237-S2.PDF]

Table 4. The primers for quantitative real time PCR

Target gene	primer set 5'-3'	Target gene	primer set 5'-3'
a_1	F:TGTGTTGCATTCATCAGCTTGTG	g_2	F:GCATCTATCTACCCTGTCTATCT
	R:ACCTTGACCTTGCTGTTGTTATTG		R:GGCCGCAACATGTCTAATAG
a_2	F:CTGTCTACCACTCATGTCATCTC	g_3	F:CTGACCATACGACTGAACCGATG
	R:AAGCCATCAATGCCAGAAGG		R:CCAATAGAGGACGAGAGCAACAC
c_1	F:GGTGGTTCAGAGTTCTTATTGTTG	i_1	F:TCCTATGTTGGTGTGATGGTAG
	R: TGACAGGCAGGCAGAAGC		R:GGTTGTTGAGAGTGATATAATAATAGAG
c_2	F:GGTGGTTCAGAGTTCTTATTGTTG	i_2	F:TGCAGATGAAAGACTATTGTCGCAG
	R: GGCACTGCGACCTGAGAG		R:AGCAGCAGTGTCTTACTAGCCTT
c_3	F:GCCTGTCTCTTTCAGTGGT	j	F:TGATCGTTATGCTGCCATCTGC
	R:AACCAAGAACATAATCACAGTCT		R:CACCTGGGAAGTCTTGTCTGC
d	F:AGCTGGTCTCTAAGTTGGTCAC	k_1	F:ATCCTCATCATTCACAGCCTCAG
	R:TTGGCACAGAACACCTTTGG		R:TGCAGCTTTGGCCACTTTCAT
e	F:GCTGGTCGGTTTGGCGGATTC	k_2	F:CTGTGCTCCACACGCAGCACT
	R:GGAGAAGAGGATGAGCGGTATGTC		R:TCCTGATGCAGCTTTGGCCACTT
f	F:TGTCTTGATTGTGTCATTGTTCTC	β-actin	F:TGGCATCACACCTTCTACAAC
	R:TTGCCTCGGACCTCCTTG		R:ACGACCAGAGGCATACAGG
g_1	F:GCCTCGTTTGCTTGTAGATATTC		
	R:GTTGGTCATTATGGCAGTATAGTG		

Note: the letters a-k represent the different clades of ORs in the phylogenetic tree, the primers were designed according to the conserved domains of ORs in each clade.

Figure 7. The standard curves and efficiencies for each pair of primers

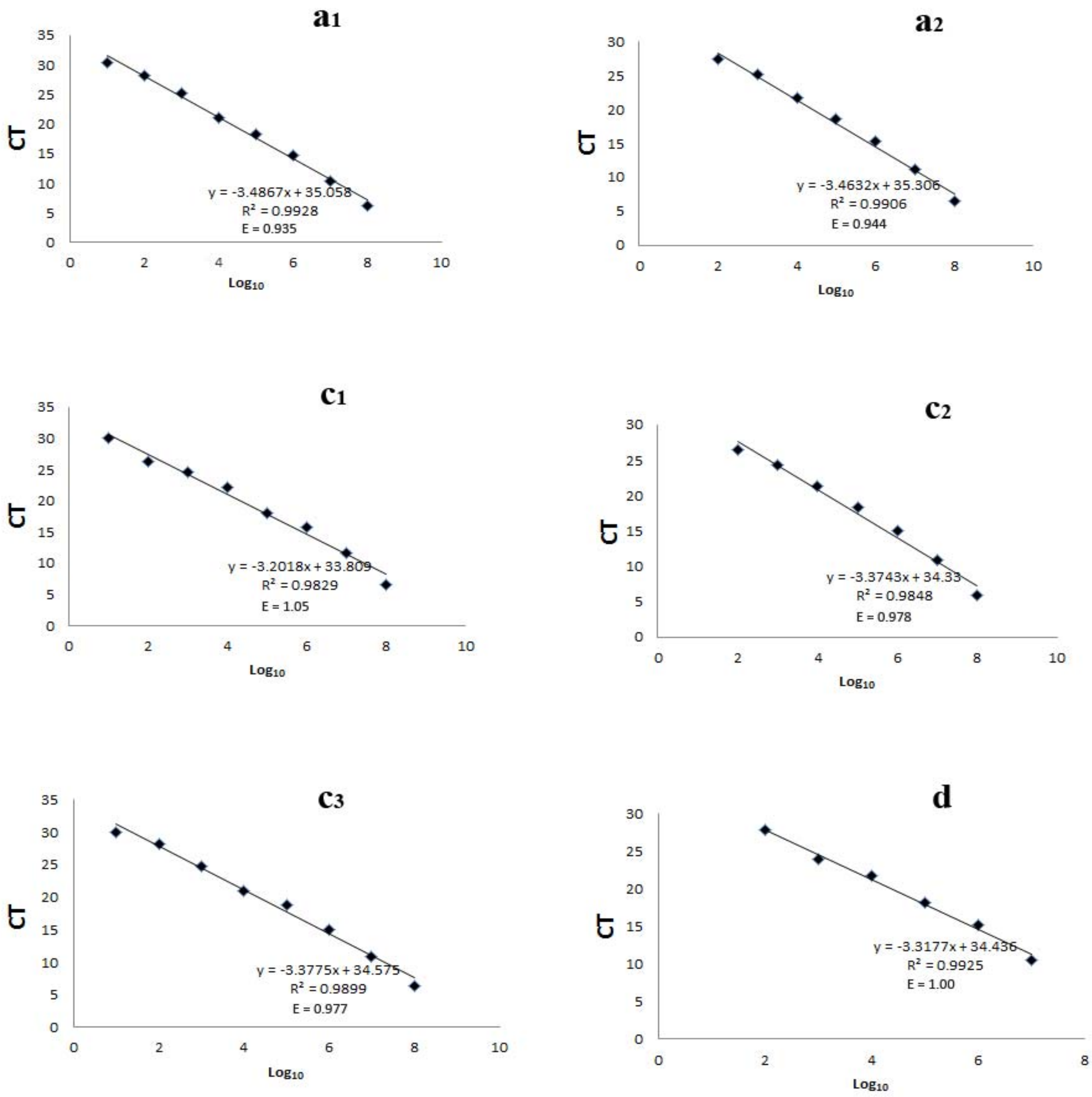

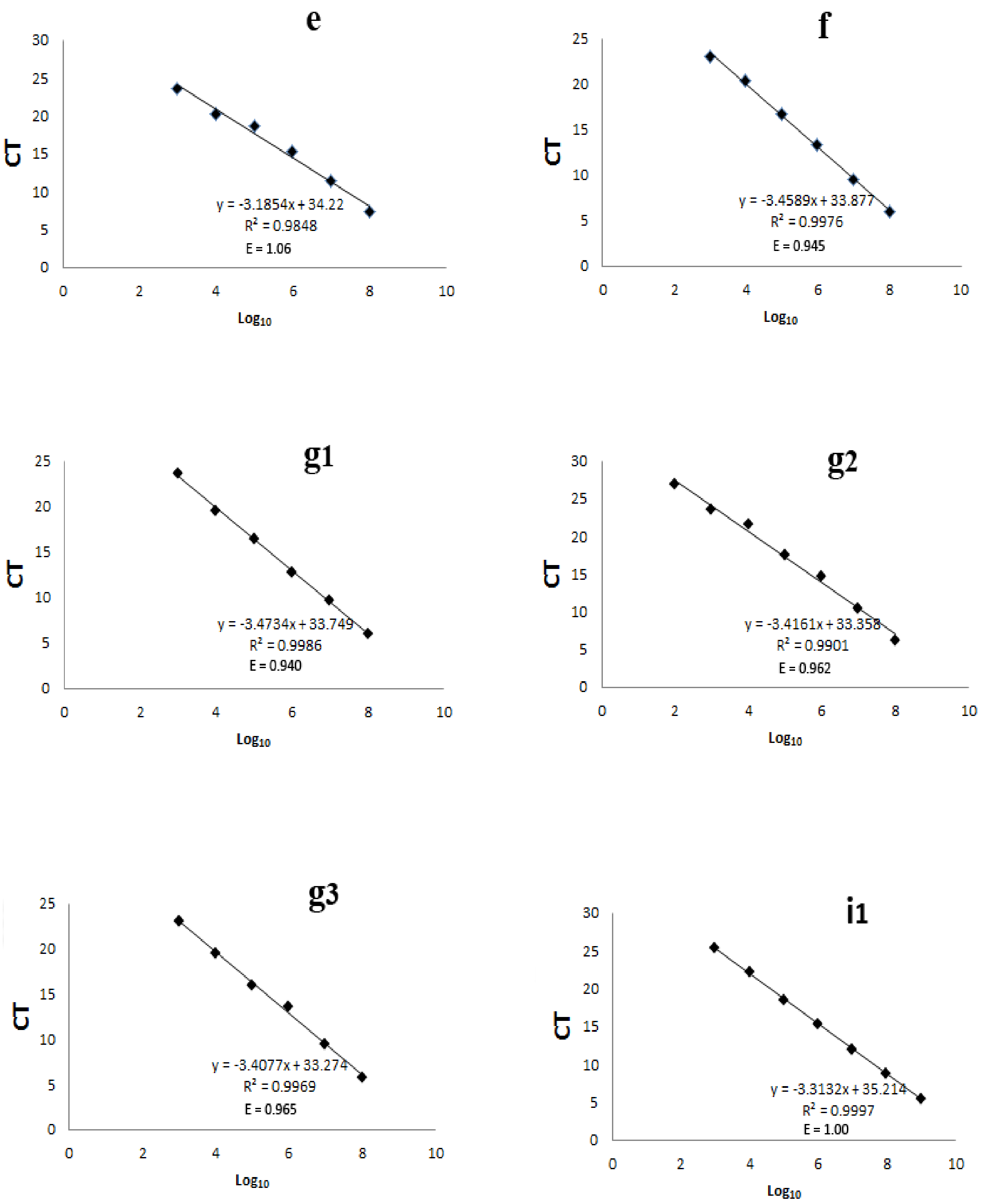

e
CT
Log10
y = -3.1854x + 34.22
R² = 0.9848
E = 1.06
f
CT
Log10
y = -3.4589x + 33.877
R² = 0.9976
E = 0.945
g1
CT
Log10
y = -3.4734x + 33.749
R² = 0.9986
E = 0.940
g2
CT
Log10
y = -3.4161x + 33.358
R² = 0.9901
E = 0.962
g3
CT
Log10
y = -3.4077x + 33.274
R² = 0.9969
E = 0.965
i1
CT
Log10
y = -3.3132x + 35.214
R² = 0.9997
E = 1.00

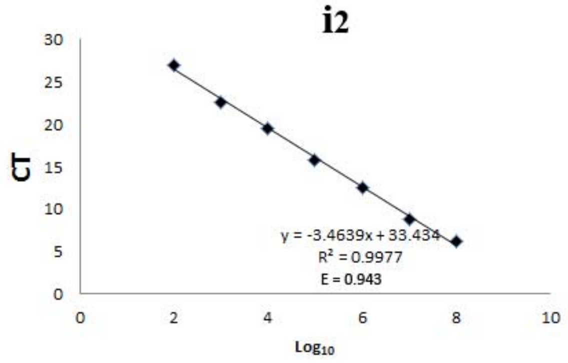
i2
CT
Log10
y = -3.4639x + 33.434
R² = 0.9977
E = 0.943

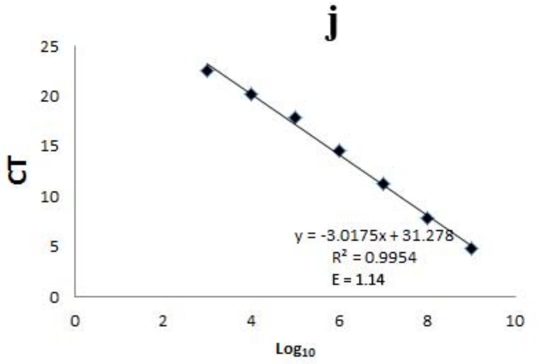
j
CT
Log10
y = -3.0175x + 31.278
R² = 0.9954
E = 1.14

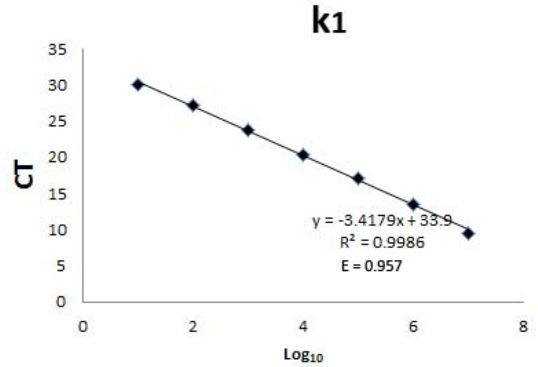
k1
CT
Log10
y = -3.4179x + 33.9
R² = 0.9986
E = 0.957

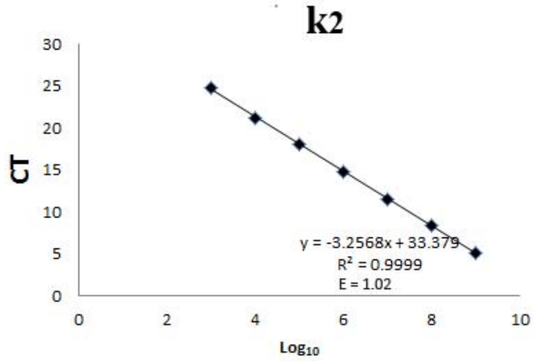
k2
CT
Log10
y = -3.2568x + 33.379
R² = 0.9999
E = 1.02

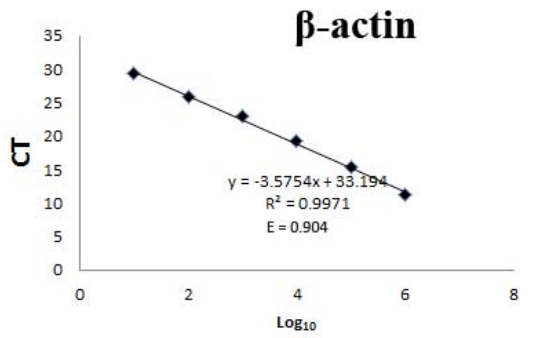
β-actin
CT
Log10
y = -3.5754x + 33.194
R² = 0.9971
E = 0.904
