# Supplementary material for: Family structure and phylogenetic analysis of odorant receptor genes in the large yellow croaker (Larimichthys crocea)
Source: BMC Evol Biol. 2011 Aug 11;11:237. doi: 10.1186/1471-2148-11-237 (PMC3162931; doi:10.1186/1471-2148-11-237)
Supplement: Additional file 3 — The average Ct value of each gene amplification is included in this file. [file 1471-2148-11-237-S3.PDF]

Table 5 The average Ct values of each OR gene expression

**Clades**	***a_1***	***a_2***	***c_1***	***c_2***	***c_3***	***d***	***e***	***f***	***g_1***
**Average Ct value**	18.51	21.19	17.42	17.19	19.51	19.65	22.96	19.05	19.12
**Clades**	***g_2***	***g_3***	***i_1***	***i_2***	***j***	***k_1***	***k_2***	***β-actin***	
**Average Ct value**	17.45	19.13	18.44	17.06	19.21	13.47	12.68	14.24	

The letters (*a*-*k*) represent each clade of OR genes among phylogenetic tree.
