# Supplementary material for: Family structure and phylogenetic analysis of odorant receptor genes in the large yellow croaker (Larimichthys crocea)
Source: BMC Evol Biol. 2011 Aug 11;11:237. doi: 10.1186/1471-2148-11-237 (PMC3162931; doi:10.1186/1471-2148-11-237)
Supplement: Additional file 4 — A list of the mitochondrial genes and OR genes of the species used in the phylogenetic analysis is included in this file, together with GenBank accession numbers and retrieval address. [file 1471-2148-11-237-S4.PDF]

The accession numbers of mitochondrial genome of 13 species

Species name	Accession number
Larimichthys crocea	EU339149.1
Tetraodon nigroviridis	AP006046
Takifugu rubripes	AP006045
Gasterosteus aculeatus	AP002944
Oryzias latipes	AP004421
Danio rerio	AC024175
Carassius auratus	NC_006580.1
Salmo trutta trutta	NC_010007
Salmo salar	NC_001960.1
Oncorhynchus mykiss	L29771.1
Oncorhynchus clarkii	AY886762.1
Xenopus tropicalis	AY789013
Branchiostoma floridae	NC_000834.1

The accession numbers of OR genes from different species

Species name	Accession number
Takifugu rubripes OR123-1	ABC43430.1
Takifugu rubripes OR6765-1	ABC43466.1
Takifugu rubripes OR4133-1	ABC43449.1
Takifugu rubripes OR3630-2	ABC43445.1
Takifugu rubripes OR8617-1	ABC43474.1
Takifugu rubripes OR5510-1	ABC43461.1
Takifugu rubripes OR117-1	ABC43428.1
Takifugu rubripes OR2346-5	ABC43442.1
Takifugu rubripes OR5287-2	ABC43460.1
Takifugu rubripes OR142-1	ABC43432.1
Takifugu rubripes OR1026-2	ABC43425.1
Takifugu rubripes OR8298-1	ABC43473.1
Takifugu rubripes OR6030-1	ABC43464.1
Takifugu rubripes OR59-1	ABC43463.1
Takifugu rubripes OR4208-3	ABC43452.1
Takifugu rubripes OR4208-2	ABC43451.1
Takifugu rubripes OR7903-1	ABC43472.1
Takifugu rubripes OR6818-1	ABC43467.1
Tetraodon nigroviridis OR8981-2	ABC43423.1
Tetraodon nigroviridis OR8981-3	ABC43424.1
Tetraodon nigroviridis OR15134-1	ABC43418.1
Tetraodon nigroviridis OR14339-2	ABC43398.1
Tetraodon nigroviridis OR14328-1	ABC43397.1
Tetraodon nigroviridis OR14677-8	ABC43410.1
Tetraodon nigroviridis OR14536-2	ABC43401.1
Tetraodon nigroviridis OR14536-1	ABC43402.1
Tetraodon nigroviridis OR10960-2	ABC43389.1
Tetraodon nigroviridis OR10960-3	ABC43388.1
Tetraodon nigroviridis OR12434-1	ABC43395.1
Danio rerio OR126-7	ABC43316.1

Danio rerio OR126-3	NP_001124271.1
Danio rerio OR123-1	NP_001124277.1
Danio rerio OR125-1	XP_002666035.1
Danio rerio OR127-1	NP_001121876.1
Danio rerio OR128-10	NP_001121842.1
Danio rerio OR112-1	ABC43383.1
Danio rerio OR116-2	NP_001122045.1
Danio rerio OR117-1	NP_001121860.1
Danio rerio OR115-15	NP_571158.1
Danio rerio OR133-7	ABC43375.1
Danio rerio OR132-1	NP_001124269.1
Danio rerio OR134-1	NP_001082920.1
Danio rerio OR131-2	NP_001034727.1
Danio rerio OR131-1	NP_001122051.1
Carassius auratus OR3	AAC64074.1
Carassius auratus OR2	AAC64072.1
Oncorhynchus mykiss OR500-1	ACU68493.1
Oncorhynchus clarkii OR600-1	ACU68496.1
Salmo salar SORB	ABD33497.1
Salmo trutta OR500-1	ACU68495.1
Oryzias latipes mfOR2	BAA84276.1
Oryzias latipes mfOR1	BAA84275.1
Oryzias latipes ORY3	BAA85099.1
Oryzias latipes ORY1	BAA85097.1
Xenopus tropicalis OR52D1	XP_002943650.1
Xenopus tropicalis OR52E8	XP_002943651.1
Xenopus tropicalis OR52A1	XP_002942410.1
Xenopus tropicalis OR52P1	XP_002942423.1
Xenopus tropicalis OR52A5	XP_002942454.1
Xenopus tropicalis OR2M5	XP_002942450.1
Xenopus tropicalis OR2M2	XP_002941611.1
Xenopus tropicalis OR24	XP_002940791.1

The OR genes from reference [20] as shown in the following

Oryzias latipes ORUn.33	reference [20]
Oryzias latipes ORUn.2	reference [20]
Oryzias latipes OR21.18	reference [20]
Oryzias latipes OR21.19	reference [20]
Oryzias latipes OR21.2	reference [20]
Oryzias latipes OR13.14	reference [20]
Oryzias latipes OR13.12	reference [20]
Oryzias latipes ORUn.23	reference [20]
Oryzias latipes ORUn.21	reference [20]
Oryzias latipes OR14.15	reference [20]
Oryzias latipes OR14.13	reference [20]
Oryzias latipes OR14.4	reference [20]
Oryzias latipes OR14.6	reference [20]
Oryzias latipes OR13.4	reference [20]
Oryzias latipes OR13.5	reference [20]
Oryzias latipes OR13.6	reference [20]

Oryzias latipes OR13.7	reference [20]
Oryzias latipes ORUn.1	reference [20]
Oryzias latipes OR21.2	reference [20]
Oryzias latipes OR21.3	reference [20]
Oryzias latipes Un.6	reference [20]
Oryzias latipes Un.28	reference [20]
Oryzias latipes Un.27	reference [20]
Oryzias latipes OR13.1	reference [20]
Oryzias latipes OR13.3	reference [20]
Gasterosteus aculeatus OR1.1	reference [20]
Gasterosteus aculeatus OR16.3	reference [20]
Gasterosteus aculeatus OR1.12	reference [20]
Gasterosteus aculeatus OR1.13	reference [20]
Gasterosteus aculeatus ORUn.9	reference [20]
Gasterosteus aculeatus OR7.1	reference [20]
Gasterosteus aculeatus ORUn.6	reference [20]
Gasterosteus aculeatus ORUn.8	reference [20]
Gasterosteus aculeatus OR1.18	reference [20]
Gasterosteus aculeatus OR16.1	reference [20]
Gasterosteus aculeatus OR16.32	reference [20]
Gasterosteus aculeatus OR16.1	reference [20]
Gasterosteus aculeatus OR16.5	reference [20]
Gasterosteus aculeatus ORUn.1	reference [20]
Gasterosteus aculeatus ORUn.17	reference [20]
Gasterosteus aculeatus OR7.3	reference [20]
Gasterosteus aculeatus OR7.4	reference [20]
Gasterosteus aculeatus OR7.8	reference [20]
Gasterosteus aculeatus OR1.25	reference [20]
Takifugu rubripes ORUn.8	reference [20]
Takifugu rubripes ORUn.20	reference [20]
Takifugu rubripes ORUn.83	reference [20]
Tetraodon nigroviridis OR7.1	reference [20]
Tetraodon nigroviridis OR7.3	reference [20]
Tetraodon nigroviridis OR16.7	reference [20]
Danio rerio OR10.1	reference [20]
Danio rerio OR10.14	reference [20]
Danio rerio OR10.29	reference [20]
Danio rerio OR10.31	reference [20]
Danio rerio OR15.39	reference [20]
Danio rerio OR15.57	reference [20]
Danio rerio OR15.58	reference [20]
Branchiostoma floridae OR105.1	reference [20]
Branchiostoma floridae OR9.1	reference [20]
