# Supplementary material for: Family structure and phylogenetic analysis of odorant receptor genes in the large yellow croaker (Larimichthys crocea)
Source: BMC Evol Biol. 2011 Aug 11;11:237. doi: 10.1186/1471-2148-11-237 (PMC3162931; doi:10.1186/1471-2148-11-237)
Supplement: Additional file 5 — A multiple sequence alignment of 12 concatenated mitochondrial proteins from 12 organisms, including amphioxus, one amphibian, and 11 ray-finned fishes, are shown in the file. [file 1471-2148-11-237-S5.PDF]

```
                                 10        20        30        40        50        60        70        80        90       100
                        ....|....|....|....|....|....|....|....|....|....|....|....|....|....|....|....|....|....|....|....|
Pufferfish              MITTLITHILNPLAFIVPVLLAVAFLTLLERKVLGYMQLRKGPNIVGPYGLLQPIADGVKLFIKEPVRPSTSSPILFILTPMLALTLAMTLWAPLPLPYP
Fugu                    MTTMLITHLIHPLTIIVPVLLAVAFLTLIERKVLGYMQLRKGPNIVGPYGLLQPIADGVKLFIKEPVRPSTSAPILFIIAPTLALTLAMMMWTPMPLPYP
Stickleback             MMSTIITHIINPLTFIVPVLLAVAFLTLLERKVLGYMQLRKGPNIVGPYGLLQPIADGLKLFIKEPIRPSTASPLLFLLAPILALTLALALWAPMPIPHP
Large_yellow_croaker    MIHTLMTHIINPLTLIVPVLLAVAFLTLLERKVLGYMQLRKGPNIVGPYGLLQPIADGIKLFIKEPIRPSTSSPILFIITPMLALTLALTLWAPMPLPYP
Goldfish                MLNTLMTHLINPLAYIVPVLLAVAFLTLIERKVLGYMQLRKGPNVVGPYGLLQPIADGVKLFIKEPVRPSTSSPFLFLAAPVLALTLAMTLWAPMPMPHP
Medaka                  MLSKILSYLINPLVIMIFVLLAVALLTLVERKVLSYMQLRKGPNVVGPYGLLQPFADGLKLFTKEPVRPSTSSPALFLITPIMALTLALTLWAPLPLPFP
Salmon                  MTTTLITHIINPLAYIVPVLLAVAFLTLLERKVLGYMQLRKGPNIVGPYGLLQPIADGLKLFIKEPVRPSTSSPFLFLATPMLALTLALTLWAPMPIPYP
Rainbow_trout           -MITLITHVINPLAYIVPVLLAVAFLTLLERKVLGYMQLRKGPNIVGPYGLLQPIADGLKLFIKETVRPSTSSPFLFLATPMLALTLALTLWAPMPIPYP
sea_trout               MITTLITHVVNPLAYIIPILLAVAFLTLLERKVLGYMQLRKGPNIVGPYGLLQPIADGLKLFIKEPVRPSTSSPFLFLATPMLALTLALTLWAPMPIPYP
Cutthroat_trout         MITTLITHVINPLAYIIPVLLAVAFLTLLERKVLGYMQLRKGPNIVGPYGLLQPIADGLKLFIKEPVRPSTSSPFLFLATPMLALTLALTLWAPMPIPYP
Zebrafish               MLDILTSHLINPLAYAVPVLIAVAFLTLVERKVLGYMQLRKGPNVMGPRGLLQSVADGVKLFIKEPIRPSMASPILFLTAPVLALILAMMLWAPMPMPYP
Frog                    -MSTLITHLINPLLYMVPILLAVAFLTLIERKVLGYMQHRKGPNIVGPMGLLQPIADGVKLFVKEPIRPSTSSQALFLLAPTMAFALAMSIWAPIPMPFS
Amphioxus               ----MILSVIHIFLYFVPVLLAVAFLTLTERKVIGYVQLRKGPNVVGPYGLLQPIADGVKLFIKEPIKPSSSNPSVFFFAPMLALILALLLWMPVPLMDA

                                110       120       130       140       150       160       170       180       190       200
                        ....|....|....|....|....|....|....|....|....|....|....|....|....|....|....|....|....|....|....|....|
Pufferfish              VLDLNLAILFILALSSLAVYSILGSGWASNSKYALVGALRAVAQTISYEVSLGLILLSLIIFTGGFTLQTFNTAQESIWLIIPAWPLAAMWYISTLAETN
Fugu                    ILDLNLAILFVLAISSLAVYSILGSGWASNSKYALMGSLRAVAQMISYEVSLGLILLSLIIFTGNFTLQTFNVTQESIWLIIPTWPLAAMWYISTLAETN
Stickleback             VIDLNLGIMFILALSSLAVYSVLGSGWASNSKYALIGALRAVAQTISYEVSLGLILLNIIIFTGGFTLQTFNVAQESVWLIIPAWPLAAMWYISTLAETN
Large_yellow_croaker    LIDLNLSILFILALSSLAVYSILGSGWASNSKYALIGALRAVAQTISYEVSLGLILLCTIIFTGGFTLQTFNTAQESIWLILPAWPLAAMWYISTLAETN
Goldfish                VTDLNLGILFILALSSFAVYSILGSGWASNSKYALIGALRAVAQTISYEVSLGLILLSVIIFSGGYTLQTFNTTQESIWLLIPAWPLAAMWYISTLAETN
Medaka                  IADLNLGVLFILALSSLAVYSILGSGWASNSKYALIGALRAVAQTISYEVSLGLILLNAIVFTGGFTLQTFSTAQEATWLLLPAWPLAAMWYISTLAETN
Salmon                  ITDLNLGVLFVLALSSLAVYSILGSGWASNSKYALIGALRAVAQTISYEVSLGLILLSVIIFTGGFTLQTFNVAQESIWLLVPAWPLAAMWYISTLAETN
Rainbow_trout           VTDLNLGVLFVLALSSLAVYSILGSGWASNSKYALIGELRAVAQTISYEVSLGLILLSVIIITGGFTLQTFNVAQESIWLLVPAWPLAAMWYISTLAETN
sea_trout               ITDLNLGVLFILALSSLAVYSILGSGWASNSKYALIGALRAVAQTISYEVSLGLILLSVIIFTGGFTLQTFNVAQESIWLLVPAWPLAAMWYISTLAETN
Cutthroat_trout         VTDLNLGVLFVLALSSLAVYSILGSGWASNSKYALIGALRAVAQTISYEVSLGLILLSVIVITGGFTLQTFNVAQESIWLLVPAWPLAAMWYISTLAETN
Zebrafish               VLDLNLGILFIMAISSLAVYSILGSGWASNSKYALIGALRAVAQTISYEVSLGLILLSAVIFSGGYTLQTFNTTQEDTWLLLPLWPLAIMWFISTLAETN
Frog                    LADLNLGILFILALSSLTVYTILGSGWSSNSKYALIGALRAVAQTISYEVTLGLILLCMIMLTGGFTLFNFNTTQEHMWLVIPGWPMAAMWYISTLAETN
Amphioxus               FIELNFAVLFVLAISSLSVYSIMASGWSSNSKYALLGALRAVAQMVSYEVSLGLIILSLICLVGGFNLAQFFSAQEEVMLMLSCWPLGIMWFISTVAETN

                                210       220       230       240       250       260       270       280       290       300
                        ....|....|....|....|....|....|....|....|....|....|....|....|....|....|....|....|....|....|....|....|
Pufferfish              RAPFDLTEGESELVSGFNVEYAGGPFALFFLAEYANILFMNTLSASLFLGASHIPMLPELTTMNLMTKAAVLSLVFLWVRASYPRFRYDQLMHLIWKNFL
Fugu                    RAPFDLTEGESELVSGFNVEYAGGPFALFFLAEYANILLMNTLSTILFLGALHMPALPELTSINLMSKTAILSLIFLWARASYPRFRYDQLMHLTWKNFL
Stickleback             RAPFDLTEGESELVSGFNVEYAGGPFALFFLAEYANILLMNTLSATLFLGAYHIPSFPELTAVNLMTKAALLSVVFLWVRASYPRFRSDQLMHLIWKNFL
Large_yellow_croaker    RAPFDLTEGESELVSGFNVEYAGGPFALFFLAEYANILLMNTLSATLFLGASHIPTIPELTAMNIMTKAALLSVLFLWVRASYPRFRYDQLMHLIWKNFL
Goldfish                RAPFDLTEGESELVSGFNVEYAGGPFALFFLAEYANILLMNTLSAVLFLGASHIPNMPELTTINLMTKAALLSILFLWVRASYPRFRYDQLMHLVWKNFL
Medaka                  RAPFDLTEGESELVSGFNVEYAGGPFALFFLAEYGNILLMNTLSAVLFLGTSTYHNLPELSATLLMLKATLLSVVFLWVRASYPRFRYDQLMHLIWKNFL
Salmon                  RAPFDLTEGESELVSGFNVEYAGGPFALFFLAEYANILLMNTLSAILFLGASHIPAFPELTAVNLMTKAALLSVVFLWVRASYPRFRYDQLMHLVWKSFL
Rainbow_trout           RAPFDLTEGESELVSGFNVEYAGGPFALFFLAEYANILLMNTLSAVLFLGASHIPAFPELTALNLMTKAALLSVVFLWVRASYPRFRYDQLMHLVWKSFL
sea_trout               RAPFDLTEGESELVSGFNVEYAGGPFALFFLAEYANILLMNTLSTILFLGASHIPAFPELTAVNLMTKAALLSVVFLWVRASYPRFRYDQLMHLVWKSFL
Cutthroat_trout         RAPFDLTEGESELVSGFNVEYAGGPFALFFLAEYANILLMNTLSAVLFLGASHIPAFPELTAMNLMTKAALLSVVFLWVRASYPRFRYDQLMHLVWKSFL
Zebrafish               RAPFDLTEGESELVSGFNVEYAAGPFALFFLAEYSNILLMNTHSTVLFLGASFTPDAPELMTISIATKTAMLSILFLWMRASYPRFRYDQLMHLIWKNFL
Frog                    RAPFDLTEGESELVSGFNVEYAGGPFVLFFLAEYANILMMNTLSTILFLGSSFI-DTPELTTISIMFKASLLSLLFLWVRASYPRFRYDQLMHLVWKNFL
Amphioxus               RSPFDLTEGESELVSGFNVEYSGGPFALFFLAEYANILFMNVLSALLFLAAHFS-------LLGVAVKVGLLAGLYLWFRASYPRFRYDQLMHLAWKSFL

                                310       320       330       340       350       360       370       380       390       400
                        ....|....|....|....|....|....|....|....|....|....|....|....|....|....|....|....|....|....|....|....|
Pufferfish              PLTLALVIWHLALPITFAGLPPQMMSPYITASLLFGLLLGTTITATSSHWLIAWMGLEINTLAIIPLMAQHHHPRAVEATTKYFLTQATAAAMLLFASTT
Fugu                    PLTLAFIIWHLALPTTMAGLPPQMMNPYITASLLFGLLLGTTITTTSTHWLIAWMGLEINTLAIIPLMAQQHHPRAIEATTKYFLTQAAAAATLLLAATT
Stickleback             PLTLALVIWHLSLPIAFAGLPPQVMNPFILATLLFGLGLGTTITFASSHWLLAWMGLEMNTLAIIPLMAQHHHPRAVEATTKYFLTQATGAAMLLFASTT
Large_yellow_croaker    PLTLALVIWHLALPIALAGLPPQAMNPIVSCTLLITLGLGTTITFASSHWFLAWMGLEINTLAILPLMAQYHHPRATEATLKYFLTQATAASTLLFATTT
Goldfish                PLTLAFVLWHTALPIALAGLPPQLMNPYVLMILLSSLGLGTTLTFASSHWLLAWMGLEINTLAITPLMAQHHHPRAVEATTKYFLTQATAAAMILFASTT
Medaka                  PLTLALVIWHLSLPISFNGLPPQLMNPYVLSILLMGLGLGTTVTFASSHWLLAWMGLEMNTLAILPLMAQHHHPRAVEAATKYFLIQSAAAATILFASST
Salmon                  PLTLALVLWHLALPTAMAGLPPQLMNPYVLTILLSSLGLGTILTFASSHWLLAWMGLEINTLAIIPIMAQQSHPRAIEATTKYFLTQATAAAMILFASTT
Rainbow_trout           PLTLALVLWHLALPIALAGLPPQLMNPYVLTILLSSLGLGTVLTFASSHWLLAWMGLEINTLAIIPIMAQQHHPRAIEATTKYFLTQATAAAMILFASTT
```

```
sea_trout           PLTLALVLWHLALPTATAGLPPQLMNPYVLTILLSSLGLGTVLTFASSHWLLAWMGLEINTLAIIPIMAQQHHPRAIEATTKYFLTQATAAAMILFASTT
Cutthroat_trout     PLTLALVLWHLALPIALAGLPPQLMNPYVLTILLSSLGLGTVLTFASSHWLLAWMGLEINTLAIIPIMAQQHHPRAIEATTKYFLTQATAAAMILFASTT
Zebrafish           PITLVLVLWHIALPIALAGLPPQTMNPYVLMILMSSLGLGTTLTFSSSHWILAWMGLEINTLAIVPLMAQQHHPRAVEATTKYFLIQAAAAAMILFTSTT
Frog                PITLAMTLWHISLPISLSGLPPQMMNPIALSMILSSLALGTILTVSSNHWFLAWMGLEINTLAIIPLMAQNHHPRAVEASTKYFLTQAAASALLLFSALN
Amphioxus           PLSLGLLMLNFSLPLTFSGIGGGLMSPYISPLFSITMIMSVMLISSSGHWVFMWLGLELGTLAFIPILVWWHSSLEVEATVKYFIVQAMAAAVFFLGGMV

                            410       420       430       440       450       460       470       480       490       500
                    ....|....|....|....|....|....|....|....|....|....|....|....|....|....|....|....|....|....|....|....|
Pufferfish          NAWLTGQWELQQMTHPLPSTLIILALALKIGLAPLHTWLPEVLQGLDLTTGLVLSTWQKLAPFALLLQLQPNN-PTLLVILGVLSTLIGGWGGLNQTQLR
Fugu                NAWMTGQWELQQATHPVPTTMITIALALKIGLAPLHTWLPEVMQGLDLTTGLILATWQKIAPFSLLLQIQPNN-QTLLILLAILSMLVGGWGGLNQTQVR
Stickleback         NAWMTGQWEIQQMTHPLPMTLITLALALKIGLAPLHSWLPEVLQGLDLTTGLIMSTWQKLAPFALLLHLDPSN-STLLVVLGLTSTLVGGWGGLNQTQLR
Large_yellow_croaker NAWLNGQWDIQHMTHPLPITLFTIALALKIGLAPPTIWLPEVLQGLDLITGLIMSTWQKLAPFALLLQIYPAN-STLLIMLGLTSTLIGGWGGLNQTQLR
Goldfish            NAWMTGEWNITDLSDPLANTMFMTALALKIGLAPMHFWMPEVMQGLDLLTGLILSTWQKLAPFALIIQTAQNIDPLLLTLLGVTSTLVGGWGGLNQTQLR
Medaka              NAWLSGQWDIQHITHPLPTVMITIALSLKLGLAPLHAWLPEVVQGLDLTTGLVLSTWQKLAPFALLLQISPEI-PLLITSLGLISMLAGGWGGLNHTQLR
Salmon              NAWLVGEWEIHQLSHPLATTTVMLALALKLGLAPVHFWLPEVLQGLELTTGLILSTWQKLAPFALMIQVAPTINSSLLIAMGLLSTLVGGWGGLNQTQLR
Rainbow_trout       NAWLVGEWEIHQLSHPLATTTVMLALALKLGLAPVHFWLPEVLQGLELTTGLILSTWQKLAPFALMIQVAPTINSSLLVTIGLLSTLVGGWGGLNQTQLR
sea_trout           NAWLVGEWEIHQLSHPLATTTAMLALALKLGLAPVHFWLPEVLQGLELTTGLILSTWQKLAPFALMIQVAPTINSSLLIAMGLLSTLVGGWGGLNQTQLR
Cutthroat_trout     NAWLMGEWEIHQLSHPLATTTVMLALALKLGLAPVHFWLPEVLQGLELTTGLILSTWQKLAPFALMIQVAPTINSSLLVAIGLLSTLVGGWGGLNQTQLR
Zebrafish           NAWISGQWDVTGMPGPATSTAMMFALALKIGLAPMHFWLPEVLQGLDLLTGLILSTWQKLAPMALIIQTTQTTDPLILTSLGIASSLIGGWSGLNQTQLR
Frog                NAWFTGEWSIMNLTNSISCTTMTIAICMKLGLAPFHFWLPEVLQGLNLTTGLILSTWQKLAPMAILYQISPSMNTSLLLLIGLTSTLIGGWGGLNQTQLR
Amphioxus           SLSGDFMGGVNQLMGNIGDMMIMLAVVTKLGLAPFHYWVVDVVQGLNYIPGAVLLTWQKVPGLAVLTQLATCNNSSMLLLFGMVSALVGGLGGLGQTQMR

                            510       520       530       540       550       560       570       580       590       600
                    ....|....|....|....|....|....|....|....|....|....|....|....|....|....|....|....|....|....|....|....|
Pufferfish          KILAYSSIAHLGWMILILQFSPTLTLLTLMLYLIMTSSAFLTFILNKTTTINALATSWAKTPILTSLLPLVLLSLGGLPPLTGFMPKWLILQELTKHDLA
Fugu                KILAYSSIAHLGWMVIILQFSPTLAVMTLALYIIMTSSTFLAFMMNKTTTIGTLTISWAKTPIISSLMPLILLSLGGLPPLTGFMPKWLILQELTKQDLG
Stickleback         KILAYSSIAHLGWMTLVMHFSPSLTLLTLIMYFIMTFPAFLVFKLNNSTTINSLATSWAKAPALTSLTPFILLSLGGLPPLVGFMPKWLILQELTKQELA
Large_yellow_croaker KILAYSSIAHLGWMILVLQFSPALTLLTLFMYIIMTFSTFLVFHLNNATNINTLATTWAKTPALTALTPLLLLSLGGLPPLSGFMPKWLILQELAKQDLA
Goldfish            KILAYSSIAHMGWMIIVIQYAPQLTLIALGTYIIMTSAAFLTLKMSLTTKLSTLATTWSKSPILTATTALVLLSLGGLPPLTGFMPKWLILQELTKQDLP
Medaka              KVLAYSSVAHLGWMMVVMQFSTSLTALALLMYVIMTTSAFLTFKLLKSTDMNSLAMSWAKTPSVTALAPMMLLSLGGLPPLSGFLPKWLIIQELTKQELG
Salmon              KILAYSSIAHLGWMVLILQYAPSLTLLSLFLYIIMTSSAFLTLKTNNSLTINTLATSWTKSPTLAALTALVLLSLGGLPPLSGFMPKWLILQELTKQGLP
Rainbow_trout       KILAYLPIAHLGWMVLILQFAPSLTLLSLSLYIVMTSSAFLTLKTNNSLTINTLATSWTKSPTLAALTALVLLSLGGLPPLSGFMPKWLILQELTKQGLP
sea_trout           KILAYSSIAHLGWMVLILQYAPSLTLLSLFLYIIMTSSAFLTLKTNNSLTINTLATSWTKSPTLAALTALVLLSLGGLPPLSGFMPKWLILQELTKQELP
Cutthroat_trout     KILAYSSIAHLGWMVLILQFAPSLTLLSLSLYIIMTSSAFLTLKTNNALTINTLATSWTKSPTLAALAALVLLSLGGLPPLSGFMPKWLILQELTKQGLP
Zebrafish           KILAYSSIAHMGWMIIVIQYAPQLTLIALGTYIFMTSAAFLTLKVLSATKINTLTTTWPKSPILAAIATLVMLSLGGLPPLTGFMPKWLILQELTKQDLP
Frog                KILAFSSIAHLGWMASILPIMPQLMILNLIIYLIMTSTLFLTLKTISSTKISTLATSWPKTPTTTALTLLTLLSLGGLPPLSGFMPKWLILQELTNQNTP
Amphioxus           KLLAFSSISHLGWLVVGCVAGSLLGLSYFTLYVVLSIPLFSILHMLNGGHLNQLRTGLMFNPLMSVLLGVGFLSLGGLPPFFGFFGKWLLLTHFVGQLLL

                            610       620       630       640       650       660       670       680       690       700
                    ....|....|....|....|....|....|....|....|....|....|....|....|....|....|....|....|....|....|....|....|
Pufferfish          PTATLAALSALLSLYFYLRLSYAMTLTIAPNNLTGTLPWRTQTTQPNMMTATMTASSILLLPMTPGILTLFN--IMAITRWFFSTNHKDIGTLYLVFGAW
Fugu                MTATLAALSALLSLYFYLRLSYAMTLTISPNNLTGTLPWRTQTNKKTLPTAILLSSSILLLPLTPEVLTLFN--TMAITRWFFSTNHKDIGTLYLVFGAW
Stickleback         AVATLAAFAALLSLYFYLRLSYAMALTMSPNSTTATTPWRYSSSQATLPLAISTTATLLLLPLAPAAIALFT--IMAITRWFFSTNHKDIGTLYLVFGAW
Large_yellow_croaker LTATLAALTALLSLYFYLRLSYALALTMFPNNPTGTPPWRLQSPQIVYPLALSITATLTLLPLTPTITALMT--PMTITRWFFSTNHKDIGTLYLIFGAW
Goldfish            IIATIMALAALISLYFYLRLCYAMTLTISPNTTNSTTPWRTQTTQTSMPLALFTMATLGLLPMTPTILMLAT---MAITRWFFSTNHKDIGTLYLVFGAW
Medaka              LVATVAALSALLSLFFYLRVCYCLTFTSSPNNLAGVAPWRLESKQISLPLATSTSLSILLLPISPAILSLTLPLFMAITRWFFSTNHKDIGTLYLIFGAW
Salmon              LSATLAAMTALLSLYFYLRLCYAMTLTIYPNTLTATAPWRLNFTLITLPLSIITILALGLLPLTPAVTTLLT--LMAITRWFFSTNHKDIGTLYLVFGAW
Rainbow_trout       LSATLAAMTALLSLYFYLRLCYALTLTIYPNTLTATAPWRLNFTMITLPLSITTIMALGLLPLTPAVTAMLA--LMAITRWFFSTNHKDIGTLYLVFGAW
sea_trout           LSATLAAMTALLSLYFYLRLCYAMTLTIYPNTLAATTPWRLNFTFITLPLSIVTILALGLLPLTPAVTAMLT--LMAITRWFFSTNHKDIGTLYLVFGAW
Cutthroat_trout     LSATLAAMTALLSLYFYLRLCYALTLTIYPNTLTATAPWRLNFTMITLPLSIITILALGLLPLTPAVTAMLA--LMAITRWFFSTNHKDIGTLYLVFGAW
Zebrafish           ATATIMALTALLSLFFYLRLCHAMTLTTSPNTINSAPHWRVQTTQNSLPLTISVTVTMGLLPLTPAILMLTT---MTITRWFFSTNHKDIGTLYLVFGAW
Frog                ILATMLALSALLSLFFYLRLSYSITLTSSPNTSNSPLTWRHNSKQPTTLLSLMLISSFFLLPISP---LMMS---MAITRWLFSTNHKDIGTLYLVFGAW
Amphioxus           GVSVVLITGTLISLFYYLRVSYLCIVVLGPQQIMSGLNWRK--MQLSGLMSGLLVLNMLGLFLVGGASCLPK---MYITRWLFSTNHKDIGTLYFIFGAW

                            710       720       730       740       750       760       770       780       790       800
                    ....|....|....|....|....|....|....|....|....|....|....|....|....|....|....|....|....|....|....|....|
Pufferfish          AGMVGTALSLLIRAELSQPGALLGDDQIYNVIVTAHAFVMIFFMVMPIMIGGFGNWLVPLMIGAPDLAFPWMSNMSFWLLPPSFLLLLASSGVEAGAGTG
Fugu                AGMVGTALSLLIRAELSQPGALLGDDQIYNVIVTAHAFVMIFFMVMPIMIGGFGNWLIPLMIGAPDMAFPRMNNMSFWLLPPSFLLLLASSGVEAGAGTG
```

```
Stickleback             AGMVGTALSLLIRAELSQPGALLGDDQIYNVIVTAHAFVMIFFMVMPIMIGGFGNWLIPLMIGAPDMAFPRMNNMSFWLLPPSFLLLLASSGVEAGAGTG
Large_yellow_croaker    AGMVGTALSLLIRAELSQPGSLLGDDQIFNVIVTAHAFVMIFFMVMPVMIGGFGNWLVPLMIGAPDMAFPRMNNMSFWLIPPSFLLLLASSGVEAGAGTG
Goldfish                AGMVGTALSLLIRAELSQPGSLLGDDQIYNVIVTAHAFVMIFFMVMPILIGGFGNWLVPLMIGAPDMAFPRMNNMSFWLLPPSFLLLLASSGVEAGAGTG
Medaka                  AGMVGTALSLLIRAELSQPGSLLGDDQIYNVIVTAHAFVMIFFMVMPIMIGGFGNWLIPLMIGAPDMAFPRMNNMSFWLLPPSFLLLLASSGVEAGAGTG
Salmon                  AGMVGTALSLLIRAELSQPGALLGDDQIYNVIVTAHAFVMIFFMVMPIMIGGFGNWLIPLMIGAPDMAFPRMNNMSFWLLPPSFLLLLASSGVEAGAGTG
Rainbow_trout           AGMVGTALSLLIRAELSQPGALLGDDQIYNVIVTAHAFVMIFFMVMPIMIGGFGNWLIPLMIGAPDMAFPRMNNMSFWLLPPSFLLLLSSSGVEAGAGTG
sea_trout               AGMVGTALSLLIRAELSQPGALLGDDQIYNVIVTAHAFVMIFFMVMPIMIGGFGNWLIPLMIGAPDMAFPRMNNMSFWLLPPSFLLLLASSGVEAGAGTG
Cutthroat_trout         AGMVGTALSLLIRAELSQPGALLGDDQIYNVIVTAHAFVMIFFMVMPIMIGGFGNWLIPLMIGAPDMAFPRMNNMSFWLLPPSFLLLLSSSGVEAGAGTG
Zebrafish               AGMVGTALSLLIRAELSQPGALLGDDQIYNVIVTAHAFVMIFFMVMPILIGGFGNWLVPLMIGAPDMAFPRMNNMSFWLLPPSFLLLLASSGVEAGAGTG
Frog                    AGMVGTALSLLIRAELSQPGTLLGDDQIYNVIVTAHAFIMIFFMVMPIMIGGFGNWLIPLMIGAPDMAFPRMNNMSFWLLPPSFLLLLASSGVEAGAGTG
Amphioxus               AAMVGTAMSLLIRAELSQPGALLGDDHLYNVIVTAHAFVMIFFMVMPIMIGGFGNWLVPMMIGAPDMAFPRMNNMSFWMLPPSFSLLLASSAVEAGVGTG

                                810       820       830       840       850       860       870       880       890       900
                        ....|....|....|....|....|....|....|....|....|....|....|....|....|....|....|....|....|....|....|....|
Pufferfish              WTVYPPLAGNLAHAGASVDLTIFSLHLAGVSSILGAINFITTIINMKPPAISQYQTPLFVWAVLITAVLLLLSLPVLAAGITMLLTDRNLNTTFFDPAGG
Fugu                    WTVYPPLAGNLAHAGASVDLTIFSLHLAGVSSILGAINFITTIINMKPPAISQYQTPLFVWAVLITAVLLLLSLPVLAAGITMLLTDRNLNTTFFDPAGG
Stickleback             WTVYPPLSGNLAHAGASVDLTIFSLHLAGISSILGAINFITTIINMKPPAISQYQTPLFVWSVLITAVLLLLSLPVLAAGITMLLTDRNLNTTFFDPAGG
Large_yellow_croaker    WTVYPPLAGNLAHAGPSVDLAIFSLHLAGVSSILGAINFITTIINMKPPGITQYQTPLFVWAVLITAVLLLLSLPVLAAGITMLLTDRNLNTTFFDPSGG
Goldfish                WTVYPPLAGNLAHAGASVDLTIFSLHLAGVSSILGAINFITTTINMKPPAISQYQTPLFVWSVLVTAVLLLLSLPVLAAGITMLLTDRNLNTTFFDPAGG
Medaka                  WTVYPPLSGNLAHAGASVDLTIFSLHLAGISSILGAINFITTIINMKPPAISQYQTPLFVWAVLITAVLLLLSLPVLAAGITMLLTDRNLNTTFFDPAGG
Salmon                  WTVYPPLAGNLAHAGASVDLTIFSLHLAGISSILGAINFITTIINMKPPAISQYQTPLFVWAVLVTAVLLLLSLPVLAAGITMLLTDRNLNTTFFDPAGG
Rainbow_trout           WTVYPPLAGNLAHAGASVDLTIFSLHLAGISSILGAINFITTIINMKPPAISQYQTPLFVWAVLVTAVLLLLSLPVLAAGITMLLTDRNLNTTFFDPAGG
sea_trout               WTVYPPLAGNLAHAGASVDLTIFSLHLAGISSILGAINFITTIINMKPPAISQYQTPLFVWAVLVTAVLLLLSLPVLAAGITMLLTDRNLNTTFFDPAGG
Cutthroat_trout         WTVYPPLAGNLAHAGASVDLTIFSLHLAGISSILGAINFITTIINMKPPAISQYQTPLFVWAVLVTAVLLLLSLPVLAAGITMLLTDRNLNTTFFDPAGG
Zebrafish               WTVYPPLAGNLAHAGASVDLTIFSLHLAGVSSILGAINFITTTINMKPPTISQYQTPLFVWAVLVTAVLLLLSLPVLAAGITMLLTDRNLNTTFFDPAGG
Frog                    WTVYPPLAGNLAHAGASVDLTIFSLHLAGVSSILGAINFITTTINMKPPAMSQYQTPLFVWSVLITAVLLLLSLPVLAAGITMLLTDRNLNTTFFDPAGG
Amphioxus               WTVYPPLSSNIAHAGASVDLAIFSLHLAGVSSILGAINFITTIHNMR-ASIEWNRVPLFVWSIWVTAYLLLLSLPVLAGAITMLLTDRNINTTFFDPSGG

                                910       920       930       940       950       960       970       980       990      1000
                        ....|....|....|....|....|....|....|....|....|....|....|....|....|....|....|....|....|....|....|....|
Pufferfish              GDPILYQHLFWFFGHPEVYILILPGFGMISHIVAYYAGKKEPFGYMGMVWAMMAIGLLGFIVWAHHMFTVGMDVDTRAYFTSATMIIAIPTGVKVFSWLA
Fugu                    GDPILYQHLFWFFGHPEVYILILPGFGMISHIVAYYSGKKEPFGYMGMVWAMMAIGLLGFIVWAHHMFTVGMDVDTRAYFTSATMIIAIPTGVKVFSWLA
Stickleback             GDPILYQHLFWFFGHPEVYILILPGFGMISHIVAYYSGKKEPFGYMGMVWAMMAIGLLGFIVWAHHMFTVGMDVDTRAYFTSATMIIAIPTGVKVFSWLA
Large_yellow_croaker    GDPILYQHLFWFFGHPEVYILILPGFGMVSHIVAYYAGKKEPFGYMGMVWAMMAIGLLGFIVWAHHMFTVGMDVDTRAYFTSATMVIAIPTGVKVFSWLA
Goldfish                GDPILYQHLFWFFGHPEVYILILPGFGIISHVVAYYSGKKEPFGYMGMVWAMMAIGLLGFIVWAHHMFTVGMDVDTRAYFTSATMIIAIPTGVKVFSWLA
Medaka                  GDPILYQHLFWFFGHPEVYILILPGFGMISHIVAYYSGKKEPFGYMGMVWAMMAIGLLGFIVWAHHMFTVGMDVDTRAYFTSATMIIAIPTGVKVFSWLA
Salmon                  GDPILYQHLFWFFGHPEVYILILPGFGMISHIVAYYSGKKEPFGYMGMVWAMMAIGLLGFIVWAHHMFTVGMDVDTRAYFTSATMIIAIPTGVKVFSWLA
Rainbow_trout           GDPILYQHLFWFFSHPEVYILILPGFGMISHIVAYYSGKKEPFGYMGMVWAMMAIGLLGFIVWAHHMFTVGMDVDTRAYFTSATMIIAIPTGVKVFSWLA
sea_trout               GDPILYQHLFWFFGHPEVYILILPGFGMISHIVAYYSGKKEPFGYMGMVWAMMAIGLLGFIVWAHHMFTVGMDVDTRAYFTSATMIIAIPTGVKVFSWLA
Cutthroat_trout         GDPILYQHLFWFFGHPEVYILILPGFGMISHIVAYYSGKKEPFGYMGMVWAMMAIGLLGFIVWAHHMFTVGMDVDTRAYFTSATMIIAIPTGVKVFSWLA
Zebrafish               GDPILYQHLFWFFGHPEVYILILPGFGIISHVVAYYAGKKEPFGYMGMVWAMMAIGLLGFIVWAHHMFTVGMDVDTRAYFTSATMIIAIPTGVKVFSWLA
Frog                    GDPVLYQHLFWFFGHPEVYILILPGFGMISHIVTYYSGKKEPFGYMGMVWAMMSIGLLGFIVWAHHMFTVDLNVDTRAYFTSATMIIAIPTGVKVFSWLA
Amphioxus               GDPILYEHLFWFFGHPEVYILILPGFGIISHIIIHYAGKLRSFGYLGMTWAMFTIGLLGFWVWAHHMFTVGMDVDTRSYFTAATMVIAVPTGIKVFSWLA

                                1010      1020      1030      1040      1050      1060      1070      1080      1090      1100
                        ....|....|....|....|....|....|....|....|....|....|....|....|....|....|....|....|....|....|....|....|
Pufferfish              TLHGG-SIKWETPMLWALGFIFLFTVGGLTGIVLANSSLDVVLHDTYYVVAHFHYVLSMGAVFAIMGAFVHWFPLFSGYTLHSTWTKIHFGVMFVGVNLT
Fugu                    TLHGG-SIKWETPMLWALGFIFLFTVGGLTGIVLANSSLDIVLHDTYYVVAHFHYVLSMGAVFAIMGAFVHWFPLFSGYTLHSTWTKIHFGVMFIGVNLT
Stickleback             TLHGG-SIKWETPLLWALGFIFLFTVGGLTGIVLANSSLDIVLHDTYYVVAHFHYVLSMGAVFAIIAGFVHWFPLFSGYTLHSTWTKVHFGVMFAGVNLT
Large_yellow_croaker    TLHGG-DLKWEAPLLWALGFIFLFTVGGLTGIVLANSSLDIVLHDTYYVVAHFHYVLSMGAVFAIMGGFVHWFPLFTGYTLHSTWTKAHFTIMFMGVNIT
Goldfish                TLHGG-SIKWETPMLWALGFIFLFTVGGLTGIVLSNSSLDIVLHDTYYVVAHFHYVLSMGAVFAIMAAFVHWFPLLTGYTLHSAWTKIHFGVMFIGVNLT
Medaka                  TLHGG-SIKWETPLLWALGFIFLFTVGGLTGIVLANSSLDIMLHDTYYVVAHFHYVLSMGAVFAIMGAFVHWFPLFSGYTLHGTWTKIHFGVMFVGVNLT
Salmon                  TLHGG-SIKWETPLLWALGFIFLFTVGGLTGIVLANSSLDIVLHDTYYVVAHFHYVLSMGAVFAIMGAFVHWFPLFTGYTLHSTWTKIHFGIMFIGVNLT
Rainbow_trout           TLHGG-SIKWETPLLWALGFIFLFTVGGLTGIVLANSSLDIVLHDTYYVVAHFHYVLSMGAVFAIMGAFVHWFPLFTGYTLHSTWTKIHFGIMFIGVNLT
sea_trout               TLHGG-SIKWETPLLWALGFIFLFTVGGLTGIVLANSSLDIVLHDTYYVVAHFHYVLSMGAVFAIMGAFVHWFPLFTGYTLHSTWTKIHFGIMFIGVNLT
Cutthroat_trout         TLHGG-SIKWETPLLWALGFIFLFTVGGLTGIVLANSSLDIVLHDTYYVVAHFHYVLSMGAVFAIMGAFVHWFPLFTGYTLHSTWTKIHFGIMFIGVNLT
Zebrafish               TLHGG-AIKWETPMLWALGFIFLFTVGGLTGIVLANSSLDIVLHDTYYVVAHFHYVLSMGAVFAIMAGFVHWFPLFTGYTLNSVWTKIHFGVMFIGVNLT
Frog                    TMHGG-TIKWDAPMLWALGFIFLFTVGGLTGIVLANSSLDIVLHDTYYVVAHFHYVLSMGAVFAIMGGFVHWFPLFTGYTLHETWAKIHFGVMFAGVNLT
```

```
Amphioxus             TLSGSKQLKWETPLLWALGFIFLFTVGGLTGIVLANSSLDIVLHDTYYVVAHFHYVLSMGAVFSILAGLTYWFPLFTGFTLQESWTKIHFFVMFVGVNLT

                                1110      1120      1130      1140      1150      1160      1170      1180      1190      1200
                      ....|....|....|....|....|....|....|....|....|....|....|....|....|....|....|....|....|....|....|....|
Pufferfish            FFPQHFLGLAGMPRRYSDYPDAYTLWNTVSSIGSLISLIAVIMFLFILWEAFAAKREVESVELTTTNVEWLHGCPPPYHTFEEPAFVQVQPLYREMAHPS
Fugu                  FFPQHFLGLAGMPRRYSDYPDAYALWNSVSSIGSMVSLVAVIMFLFILWEAFTAKREVQSVELTMTNVEWLHGCPPPYHTFEEPAFVQTQTSIREMAHPS
Stickleback           FFPQHFLGLAGMPRRYSDYPDAYTLWNTVSSIGSLVSLVAVIMFLFIIWEAFAAKREVLAVEMTTTNVEWLHGCPPPYHTFEEPAFVQVQ---SNMANPS
Large_yellow_croaker  FFPQHFLGLAGMPRRYSDYPDGYALWNTISSLGSLLSLVAVIILLFIIWEAFAAKREVLEVRFTSTNVEWLHGCPPPYHTFEEPPFVQVQ-EGRAMAHPA
Goldfish              FFPQHFLGLAGMPRRYSDYPDAYALWNTVSSIGSLISLVAVIMFLFILWEAFAAKREVLSVELTMTNVEWLHGCPPPYHTYEEPAFVQIQ---SNMAHPT
Medaka                FFPQHFLGLAGMPRRYSDYPDAYTLWNTISSLGSLISLIAVIMFLFILWEAFAAKREVLSVELTATNVEWLHGCPPPYHTFEEPAFVQIQ-QPKFMAHPS
Salmon                FFPQHFLGLAGMPRRYSDYPDAYTLWNTISSIGSLISLVAVIMFLFILWEAFAAKREVASIEMTSTNVEWLHGCPPPYHTFEEPAFVQVQ---ASMAHPS
Rainbow_trout         FFPQHFLGLAGMPRRYSDYPDAYTLWNTVSSIGSLVSLVAVIMFLFILWEAFAAKREVASIELTSTNVEWLHGCPPPYHTFEEPAFVQVQ---ANMAHPS
sea_trout             FFPQHFLGLAGMPRRYSDYPDAYTLWNTVSSIGSLVSLVAVIMFLFILWEAFAAKREVASIELTSTNVEWLHGCPPPYHTFEEPAFVQVQ---ASMAHPS
Cutthroat_trout       FFPQHFLGLAGMPRRYSDYPDAYTLWNTVSSIGSLVSLVAVIMFLFILWEAFAAKREVASIELTSTNVEWLHGCPPPYHTFEEPAFVQVQ---ANMAHPS
Zebrafish             FFPQHFLGLAGMPRRYSDYPDAYALWNTVSSIGSLISLVAVIMFLFILWEAFTAKREVLSVELTATNVEWLHGCPPPYHTFEEPAFVQIQ---SNMAHPA
Frog                  FFPQHFLGLAGMPRRYSDYPDAYTLWNTVSSVGSLISLVAVIMMMFIIWEAFAAKREVTLTELTSTNIEWLHGCPPPYHTFEEPAFVQIHPS-HNMAHPS
Amphioxus             FFPQHFLGLAGMPRRYSDYPDAYTIWNVISSLGSIISLGSVVFFLFILWEAFSSQRKAVPASHASHSAEWLMGCPPLFHTHEELPFISKK----YMATPA

                                1210      1220      1230      1240      1250      1260      1270      1280      1290      1300
                      ....|....|....|....|....|....|....|....|....|....|....|....|....|....|....|....|....|....|....|....|
Pufferfish            QLGFQDAASPVMEELLHFHDHALMIVFLISTLVLYIIVATVSTKLTDKYILDSQEIEVIWTIMPAVILILIALPSLRILYLMDEINDPHLTVKTMGHQWY
Fugu                  QLGFQDAASPVMEELLHFHDHALMIVFLISTLVLYIIVAMVSTKLTNKYILDSQEIEIIWTILPAIILILIALPSLRILYLMDEINDPHLTIKAMGHQWY
Stickleback           QLGFQDAASPVMEELLHFHDHTLMIVFLISTLVLYIIVAMVSTKLTNKYILDSQEIEIIWTILPAIILILIALPSLRILYLMDEVNNPLLTIKAVGHQWY
Large_yellow_croaker  QLGFQDATSPLMEELLHFHDHALMIVLLISVMVLYIITCLITTKMTDKHILDSQEIEIVWTVLPAITLILIALPSLRILYLMDEINDPHLTIKAMGHQWY
Goldfish              QLGFQDAASPVMEELLHFHDHALMIVFLISTLVLYIIIAMVSTKLTNKYILDSQEIEIVWTILPAVILVLIALPSLRILYLMDEINDPHLTIKAMGHQWY
Medaka                QLGFQDAASPVMEELLHFHDHALMIVFLISTLVLYIIVAMVTTKLTNKFILDSQEIEIIWTLLPAIILILIALPSLRILYLMDEINDPHLTIKAMGHQWY
Salmon                QLGFQDAASPVMEELLHFHDHALMIVLLISTLVLYIIVAMVSTKLTNKYILDSQEIEIVWTVLPAVILILIALPSLRILYLMDEINDPHLTIKAMGHQWY
Rainbow_trout         QLGFQDAASPVMEELLHFHDHALMIVLLISTLVLYIIVAMVSTKLTNMYILDSQEIEIVWTVLPAVILILIALPSLRILYLMDEINDPHLTIKAMGHQWY
sea_trout             QLGFQDAASPVMEELLHFHDHALMIVLLISTLVLYIIVAMVSTKLTNKYILDSQEIEIIWTVLPAIILILIALPSLRILYLMDEINDPHLTIKAMGHQWY
Cutthroat_trout       QLGFQDAASPVMEELLHFHDHALMIVLLISTLVLYIIVAMVSTKLTNKYILDSQEIEIVWTVLPAVILILIALPSLRILYLMDEINDPHLTIKAMGHQWY
Zebrafish             QLGFQDAASPVMEELLCFHDHALMIVFLISTLVLYIIIAMVSTKLTNKFILDSQEIEIVWTVLPAIILILIALPSLRILYLMDEINDPHVTIKAVGHQWY
Frog                  QLGFQDAASPIMEELLHFHDHTLMAVFLISTLVLYIITIMMTTKLTNTNSMDAQEIEMVWTIMPAIILIMIALPSLRILYLMDEVNDPHLTVKTIGHQWY
Amphioxus             QLGLMDAASPVMEEMIYFHDHVMLVLILITCLIFYSMLVLISSKYIYRFLTDGHVIETVWTVIPAIILVVVALPSLKLLYLTDELDNPQLTIKSVGHQWY

                                1310      1320      1330      1340      1350      1360      1370      1380      1390      1400
                      ....|....|....|....|....|....|....|....|....|....|....|....|....|....|....|....|....|....|....|....|
Pufferfish            WSYEYTDYDDLSFDSYMIPTQDLTPGQFRLLETDHRMVIPVDSPIRVLVSAEDVLHSWAVPSLGVKMDAVPGRLNQTAFIVSRPGVFYGQCSEICGANHS
Fugu                  WSYEYTDYSDLAFDSYMVPTQDLAPGQFRLLETDHRMVVPVDSPIRILVSAEDVLHSWAVPSLGVKMDAVPGRLNQTAFILSRPGVFYGQCSEICGANHS
Stickleback           WSYEYTDYEDLGFDAYMIPTQDLAPGQFRLMETDHRMVIPAESPIRVLVSADDVLHSWAVPSLGIKMDAVPGRLNQTAFIASRPGIFYGQCSEICGANHS
Large_yellow_croaker  WSYEYTDYEDLAFDSYMLPTQDLSPGQFRLLEADHRMVVPVESPIRVLISAEDVLHSWVVPTLGIKMDAVPGRLNQTAFITARPGVYYGQCSEICGANHS
Goldfish              WSYEYTDYENLGFDSYMVPTQDLAPGQFRLLETDHRMVVPMESPVRVLVSAEDVLHSWAVPSLGVKMDAVPGRLNQTAFIASRPGVFYGQCSEICGANHS
Medaka                WSYEYTDYEDLGFDSYMIPTQDLTPGQFRLLETDHRMVIPVESPIRVLVSAEDVLHSWAVPSLGVKMDAVPGRLNQTAFITSRPGVFYGQCSEICGANHS
Salmon                WSYEYTDYEDLGFDSYMVPTQDLTPGQFRLLETDHRMVVPVESPIRVLVSAEDVLHSWAVPSLGVKMDAVPGRLNQTAFIASRPGVFYGQCSEICGANHS
Rainbow_trout         WSYEYTDYEDLGFDSYMVPTQDLVPGQFRLLETDHRMVVPVESPIRVLVSAEDVLHSWAVPSLGVKMDAVPGRLNQTAFIASRPGVFYGQCSEICGANHS
sea_trout             WSYEYTDYEDLGFDSYMVPTQDLVPGQFRLLETDHRMVVPVESPIRVLVSAEDVLHSWAVPSLGVKMDAVPGRLNQTAFIASRPGVFYGQCSEICGANHS
Cutthroat_trout       WSYEYTDYEDLGFDSYMVPTQDLAPGQFRLLETDHRMVVPVESPIRVLVSAEDVLHSWAVPSLGVKMDAVPGRLNQTAFIASRPGVFYGQCSEICGANHS
Zebrafish             WSYEYTDYENLEFDSYMVPTQDLTPGGFRLLETDHRMVVPKESPIRILVSAEDVLHSWAVPSLGIKMDAVPGRLNQTAFIVSRPGVFYGQCSEICGANHS
Frog                  WSYEYTNYEDLSFDSYMIPTSDLTPGQFRLLEVDNRMVVPMESPIRLLVTAEDVLHSWALPSFGVKTDAIPGRLNQTSFIATRPGVFYGQCSEICGANHS
Amphioxus             WSYEYTDYYDIEFDSYMLPLGDLSKGDARLLEVDNRVVLPVDTSVRVLVTAADVIHSWTVPSLGLKMDAVPGRLNQLALQCSRVGTFYGQCSEICGANHS

                                1410      1420      1430      1440      1450      1460      1470      1480      1490      1500
                      ....|....|....|....|....|....|....|....|....|....|....|....|....|....|....|....|....|....|....|....|
Pufferfish            FMPIVVEAVPLEHFENWSSLMLEDAMPQLNPSPWFAIMVFSWFVFLTFLPPKIMAHTFPNEPTAQSTQTLKTKSWTWPWHMTLSFFDQFLSPTLFGIPLI
Fugu                  FMPIVVEAVPLEHFENWSSLMLEDAMPQLNPAPWFLIMVFSWCVFLIFLPPKIMAHLFPNEPSSQNTCPKEIKPWPWSWHMTLSFFDQFLSPTAFGIPLI
Stickleback           FMPIVVEAVPMTYFEDWSSRMLEDAMPQLDPSPWFAMLIFSWLVFLVVIPPKVMAHIFSNEPAMQSAEKRFTDPWTWPWPMTLSFFDQFMSPVFMGIPLA
Large_yellow_croaker  FMPIVVEAVPLNHFEAWTTLMLEEAMPQLNPSPWLAIMIFSWLAFLIIVPPKVMAHLFPNEPSPQSTETLNMETWNWPWLMTVSLFDQFMSPVYLGVPLL
Goldfish              FMPIVVEAVPLEHFENWSSLMLEDAMPQLNPGPWFAILVFSWLVFLTIIPTKILSHISPNEPTPVSAEKHKTESWDWPWMMVS-FFDQFASPSYLGIPLI
Medaka                FMPIVVEAVPLEHFENWSFIMLQDAMPQLNPAPWFAIMVFSWLVFLAVLPPKVLAHYFPNDPTPQSVKKSKSETWTWPWLMTLSLFDQFMSPTLLGVPLI
```

```
Salmon                FMPIVVEAVPLEHFEKWSTMMLEDAMPQLNPAPWFAILVFSWLVFLTVIPPKVLGHTFTNEPTSQSTEKAKPEPWNWPWHMTLSFFDQFMSPTYLGIPLI
Rainbow_trout         FMPIVVEAVPLEHFEKWSTMMLEDAMPQLNPAPWFAILVFSWLVFLTVIPPKVLGHTFTNEPTSQSTEKAKPEPWNWPWHMTLSFFDQFMSPTYLGIPLI
sea_trout             FMPIVVEAVPLEHFEKWSTMMLEDAMPQLNPAPWFAILVFSWLVFLTVIPPKVLGHTFTNEPTSQSTEKAKPEPWNWPWHMTLSFFDQFMSPTYLGIPLI
Cutthroat_trout       FMPIVVEAVPLEHFEKWSTMMLEDAMPQLNPAPWFAILVFSWLVFLTVIPPKVLGHTFTNEPTSQSTEKAKPEPWNWPWHMTLSFFDQFMSPTYLGIPLI
Zebrafish             FMPIVVEAVPLEFFENWSSAMLEDAMPQLNPKPWFMILFFSWVIFLTIIPTKIINHIQPNDPTQVDAKEHKNDTWNWPWMMTS-FFDQFASPYLLGIPLI
Frog                  FMPIVVEAVPLNDFENWSSSMLE-AMPQLNPGPWFAILIFSWLVLLVIIPPKILKHKTFNEPTTQTTEKQKPNPWNWPWTMNLSFFDQFMSPTLLGVPLI
Amphioxus             FMPIVIEAVPVEVFEGWCDMMLDEEMPQLNPIPWVFLFFLVWLVLGFLGLQKFTSIVTTTLDDSSEEVEVKSKEYSWPWMMVS-LFSQFDSPWLLNIPLV

                                1510      1520      1530      1540      1550      1560      1570      1580      1590      1600
                      ....|....|....|....|....|....|....|....|....|....|....|....|....|....|....|....|....|....|....|....|
Pufferfish            ALALLLPWTLFPAPSSRWVNSRLLTLQSWFINRFTQQLLLPLNMGGHKWALMFASLMVFLISINMLGLLPYTFTPTTQLSLNMALAVPLWLMTVIIGLRK
Fugu                  ALALLLPWTLFPTPTNRWTNNRLLTLQSQFINRFTQQLLLPLNMGGHKWALMFASLMVFLITINMLGLLPYTFTPTTQLSVNMALAVPLWLATVIIGMRN
Stickleback           AIAIALPWVLLPTPTARWTSNRFLGLQGWFINRFTQQLLLPVNLGGHKWAALLTSLMIFLITLNMLGLLPYTFTPTTQLSINLGLATPLWLATVIIGMRN
Large_yellow_croaker  AIALMLPWVLFPSPALRWIHNRLLILQSWFISRFTHQILMPLNQGGHNWALMLASLMIFLVTLNVLGLLPHTFTPTTQLSLNLAFAFPLWLATVLIGLRN
Goldfish              AIAIALPWVLYPTSSSRWINNRLITIQGWFINRFTNQLMLPLNVGGHKWALLLASLMIFLITINMLGLLPYTFTPTTQLSLNMGFAVPLWLATVIIGMRN
Medaka                GLALALPWVLFFQPGARWMNNRLVTLQAVFMNWFVKQIFQPMSLGGHKWAALLMSLMLFLITLNMLGLLPYTFTPTTQLSLNMAFAVPLWLATVIIGMRN
Salmon                AVALTLPWILFPTPSTRWLNNRLITLQGWFINRFTQQLLLPLNLGGHKWAVLLTSLMLFLITLNMLGLLPYTFTPTTQLSLNMGLAVPLWLATVIIGMRN
Rainbow_trout         AVALTLPWILFPTPSARWLNNRLITLQGWFINRFTQQLLLPLNLGGHKWAALLTSLMLFLITLNMLGLLPYTFTPTTQLSLNMGLAVPLWLATVIIGMRN
sea_trout             AVALTLPWILFPTPSTRWLNNRLITLQGWFINRFTQQLLLPLNLGGHKWAVLLTSLMLFLITLNMLGLLPYTFTPTTQLSLNMGLAVPLWLATVIIGMRN
Cutthroat_trout       AVALTLPWILFPTPSARWLNNRLITLQGWFINRFTQQLLLPLNLGGHKWAALLTSLMLFLITLNMLGLLPYTFTPTTQLSLNMGLAVPLWLATVIIGMRN
Zebrafish             LVAMLLPWLLFPAPTSRWINNRLITVQTWLTGRFTNQLMTPLNFSGHKWALLFASLMVFLITINLLGLLPYTFTPTTQLSLNMGFAVPLWLATVIIGMKN
Frog                  AIAMLIPWLLFPNPSNRWLNNRLMTLQSWFIHNFTKQIFLPINTPGHKWALILASLMLLLMSLNLLGLLPYTFTPTTQLSLNMGLAVPLWLATVIIGLRN
Amphioxus             LLALIMPWKLFVSFGPSWAGTRSSRLVYATMETLMSQVMQPLNKLGFRWVVLFSSLMLMLMTLNVIGLFPYTFTPTTQLSMNLGLAVPLWLGTVVYGFRN

                                1610      1620      1630      1640      1650      1660      1670      1680      1690      1700
                      ....|....|....|....|....|....|....|....|....|....|....|....|....|....|....|....|....|....|....|....|
Pufferfish            NPTAALGHLLPEGTPVPLIPALILIETISLFIRPLALGVRLTANLTAGHLLIQLIATAAFVLLPLMPTVAILTTILLFLLTLLEVAVAMIQAYVFVLLLS
Fugu                  NPTAALGHLLPEGTPNALIPILIIIETVSLFIRPLALGVRLTANLTAGHLLIQLIATAAFVLLPLMPTVAILTSTLLFLLTLLEVAVAMIQAYVFVLLLS
Stickleback           QPTHALGHLLPEGTPGPLIPVLIIIETISLFIRPLALGVRLTANLTAGHLLIQLIATASFVLLSLMPAVAITVTVVLFLLTLLEVAVAMIQAYVFVLLLT
Large_yellow_croaker  NPTAALGHLLPEGTPTLLIPILIIIETISLLIRPLALGVRLTANLTAGHLLMHLTSSAAYYLMFTMPAAAALTMGLLLLLTLLEMAVAVIQAYVFVLLLS
Goldfish              QPTVALGHLLPEGTPIPLIPVLIIIETISLFIRPLALGVRLTANLTAGHLLIQLIATAVFVLLPMMPTVAILTATVLFLLTLLEVAVAMIQAYVFLLLLS
Medaka                QPTHALGHLLPEGTPTALIPVLIVIETISLFIRPLALGVRLTANLTAGHLLIQLIATAAFVLFPMMPSVAILTSILLFLLTLLEIAVAMIQAYVFVLLLS
Salmon                QPTAALGHLLPEGTPVPLIPVLIIIETISLFIRPLALGVRLTANLTAGHLLIQLIATAAFVLMPIMPTVAILTSIVLFLLTLLEIAVAMIQAYVFVLLLS
Rainbow_trout         QPTAALGHLLPEGTPVPLIPVLIIIETISLFIRP-ALGVRLTANLTAGH---QLIATAAFVLLPMMPTVAILTSIVLFLLTLLEIAVAMIQAYVFVLLLS
sea_trout             QPTAALGHLLPEGTPVPLIPVLIIIETISLFIRPLALGVRLTANLTAGHLLIQLIATAAFVLLPMMPTVAILTSIVLFLLTLLEIAVAMIQAYVFVLLLS
Cutthroat_trout       QPTAALGHLLPEGTPVPLIPVLIIIETISLFIRPLALGVRLTANLTAGHLLIQLIATAAFVLLPMMPTVAILTSIVLFLLTLLEIAVAMIQAYVFVLLLS
Zebrafish             QPTIALGHLLPEGTPIPLIPALIIIETISLFIRPLALGVRLTANLTAGHLLIQLIATAVFVLLPMMPAVAILTASVLFLLTLLEVAVAMIQAYVFILLLS
Frog                  QPTVALGHLLPEGTPTPLIPVLIIIETISLFIRPLALGVRLTANLTAGHLLIQLIATAAFVLLPIMPTVSILTSIVLFLLTLLEVAVAMIQAYVFVLLLS
Amphioxus             HPVIALAHLCPEGAPNLLVPVLVVVETLSILMRPLALGLRLTANLTAGHLLMHLISSAVLGLMELSVMLSGITLLLLVFLTMLEIAVALIQGYVFAILVT

                                1710      1720      1730      1740      1750      1760      1770      1780      1790      1800
                      ....|....|....|....|....|....|....|....|....|....|....|....|....|....|....|....|....|....|....|....|
Pufferfish            LYLQENVMA-HQAHAYHMVDPSPWPLTGAVAALLLTSGLAIWFHFNSLILLTLGLVLLLLTMYQWWRDIVREGTFQGHHTPPVQKGLRYGMILFITSEVF
Fugu                  LYLQENVMA-HQAHPYHMVDPSPWPLTGAVAALLLTSGLAIWFHFNSTILMTLGLVLLLLTMLQWWRDIVREGTFQGHHTPPVQKGLRYGMILFITSEVF
Stickleback           LYLQENVMT-HQAHPYHMVDPSPWPLTGAIAALLMTSGLATWFHFQSTTLMSLGMALLLLTMYQWWRDIVREGTFQGHHTPPVQKGLRYGMILFITSEVF
Large_yellow_croaker  LYLQENVMA-HQAHAYHMVDPSPWPLTGAVGALLLTSGLAVWFHFHSTILLSLGLILLLLTMYQWWRDIIREGTFQGHHTPPVQKGLRFGMILFITSEVF
Goldfish              LYLQENVMA-HQAHAYHMVDPSPWPLTGAIAALLMTSGLAIWFHFHSTTLMTLGLILLLLTMYQWWRDIIREGTFQGHHTPPVQKGLRYGMILFITSEVF
Medaka                LYLQENVMA-HQAHAYHMVDPSPWPLTGAVAALLLTSGTAIWMHFNSLVLMTLGLVLLLLTMYQWWRDIIREGTFQGHHTPPVQKGLRYGMILFITSEVF
Salmon                LYLQENVMA-HQAHAYHMVDPSPWPLTGAIAALLLTSGTAVWFHFHSLTLLTMGNILLLLTMYQWWRDIIREGTFQGHHTPPVQKGLRYGMILFITSEVF
Rainbow_trout         LYLQENVMA-HQAHAYHMVDPSPWPLTGAIAALLLTSGTAVWFHFHSLTLLTLGNILLLLTMYQWWRDIIREGTFQGHHTPPVQKGLRYGMILFITSEVF
sea_trout             LYLQENVMA-HQAHAYHMVDPSPWPLTGAIAALLLTSGTAVWFHFHSLTLLTMGNILLLLTMYQWWRDIIREGTFQGHHTPPVQKGLRYGMILFITSEVF
Cutthroat_trout       LYLQENVMA-HQAHAYHMVDPSPWPLTGAIAALLLTSGTAVWFHFHSLTLLTLGNILLLLTMYQWWRDIIREGTFQGHHTPPVQKGLRYGMILFITSEVF
Zebrafish             LYLQENIMA-HQAHAYHMVDPSPWPLTGAVAALLMSSGLAIWFHLHSMTLIVLGMILLILTMIQWWRDIIREGTFQGHHTPPVQKGLRYGMILFITSEVF
Frog                  LYLQENVMA-HQAHAYHMVDPSPWPLTGAVAALLLTSGLAMWFHFGSLILLTLGLITMILTMIQWWRDIIREGTFQGHHTPPVQKGLRYGMILFITSEVF
Amphioxus             LYLDENLMTGYQPHPWHLVEPSPWPLVGGSAAFTLTVGLVMWFHYNSISLMILGLVMIVATMIQWWRDVIREATFQGCHTSYVLSGLRRGMVLFIVSEVF

                                1810      1820      1830      1840      1850      1860      1870      1880      1890      1900
                      ....|....|....|....|....|....|....|....|....|....|....|....|....|....|....|....|....|....|....|....|
```

```
Pufferfish           FFLGFFWAFYHSSLAPTPELGGCWPPTGIVPLNPFEVPLLNTAVLLASGVTVTWAHHSIMEGERKQAIHSLTLTILLGFYFTFLQAMEYYEAPFTIADGV
Fugu                 FFLGFFWAFYHASLAPTPELGGCWPPTGIIPLNPFEVPLLNTAVLLASGVTVTWAHHSIMEGERKQAIQSLTLTILLGFYFTFLQAMEYYEAPFTIADGV
Stickleback          FFLGFFWAFYHASLAPTPELGGCWPPTGITTLDPFEVPLLNTAVLLASGVTVTWAHHSIMEGERKQAIQSLALTILLGFYFTFLQGMEYYEAPFTIADGV
Large_yellow_croaker FFLGFFWAFYHSSLAPTPELGGCWPPTGITTLDPFEVPLLNTAVLLASGVTVTWAHHSIMEGEREQAIHSLTLTILLGFYFTFLQAMEYYEAPFTIADGV
Goldfish             FFLGFFWAFYHSSLAPTPELGGCWPPTGITPLDPFEVPLLNTAVLLASGVTVTWAHHSIMEGERKQAIQSLALTILLGLYFTALQAMEYYEAPFTIADGV
Medaka               FFLGFFWAFYHSSLAPTPELGGCWPPTGITTLDPFEVPLLNTAVLLASGVTVTWAHHSIMEGQRKQAIQSLTLTILLGFYFTFLQAMEYFEAPFTIADGV
Salmon               FFLGFFWAFYHSSLSPTPELGGCWPPTGIITLDPFEVPLLNTAVLLASGVTVTWAHHSIMEGERKQTIQALTLTILLGFYFTFLQGMEYYEAPFTIADGV
Rainbow_trout        FFLGFFWAFYHASLAPTPELGGCWPPAGITTLDPFEVPLLNTAVLLASGVTVTWAHHSIMEGERKQTIQALTLTILLGFYFTFLQGMEYYEAPFTIADGV
sea_trout            FFLGFFWAFYHSSLAPSPELGGCWPPTGIITLDPFEVPLLNTAVLLASGVTVTWAHHSIMEGERKQTIQALTLTILLGFYFTFLQGMEYYEAPFTIADGV
Cutthroat_trout      FFLGFFWAFYHASLAPTPELGGCWPPTGITTLDPFEVPLLNTAVLLASGVTVTWAHHSIMEGERKQTIQALTLTILLGFYFTFLQGMEYYEAPFTIADGV
Zebrafish            FFLGFFWAFYHSSLAPTPELGGCWPPTGLTTLDPFEVPLLNTAVLLASGVTVTWAHHSLMEGERKQAIQSLALTILLGLYFTALQAMEYYEAPFTIADGV
Frog                 FFIGFFWAFYNSSLAPTYELGECWPPTGITPLNPFEVPLLNTAVLLASGVTVTWAHHSIMHGNRKEAIQSLGLTILLGLYFTALQAMEYYEAPFTIADGV
Amphioxus            FFLAFFWAFFHSSLAPTVELGVTWPPVGVHPLNAFAVPLLNTAVLLSSGVTVTWAHHALMEGKRTEAIQSLAITVMLGLYFTGLQAWEYYEAPFTIADSV

                              1910      1920      1930      1940      1950      1960      1970      1980      1990      2000
                     ....|....|....|....|....|....|....|....|....|....|....|....|....|....|....|....|....|....|....|....|
Pufferfish           YGSTFFVATGFHGLHVIIGSTFLAICLLRQIRYHFTSEHHFGFEAAAWYWHFVDVVWLFLYISIYWWGSMNLLLTILFITTILSLILAIVSFWLPLMTPD
Fugu                 YGSTFFVATGFHGLHVIIGSTFLAVCLLRQIRFHFTSEHHFGFEAAAWYWHFVDVVWLFLYISIYWWGSMNLLTTMLIITTALSLILMTVSFWLPALTPD
Stickleback          YGSSFFVATGFHGLHVIIGSSFLAVCFLRQIRHHFTAEHHFGFEAAAWYWHFVDVVWLFLYISIYWWGSMNLVTTVVSITAALSLVLALVSFWLPQMTPD
Large_yellow_croaker YGSTFFVATGFHGLHVIIGSTFLAVCLLRQVQYHFTSGHHFGFEAAAWYWHFVDVVWLFLYISIYWWGSMNLVMVILIIATLLSALLATVSFWLPQMTPD
Goldfish             YGSTFFVATGFHGLHVIIGSTFLAVCLLRQIQYHFTSEHHFGFEAAAWYWHFVDVVWLFLYVSIYWWGSMNLIMTILTITAALSLILATISFWLPQMNPD
Medaka               YGSTFFVATGFHGLHVIIGSTFLAVCLLRQVQFHFTSEHHFGFEAAAWYWHFVDVVWLFLYISIYWWGSMNLVMVTLLISLVLATVLAIASFFLPQMTPD
Salmon               YGSTFFVATGFHGLHVIIGSTFLAICLLRQIQYHFTSEHHFGFEAAAWYWHFVDVVWLFLYVSIYWWGSMNLITTIIAITITLSAVLATISFWLPQMTPD
Rainbow_trout        YGSTFFVATGFHGLHVIIGSTFLAVCLLRQVQYHFTSEHHFGFEAAAWYWHFVDVVWLFLYVSIYWWGSMNLITTIITITITLSAVLATISFWLPQISPD
sea_trout            YGSTFFVATGFHGLHVIIGSTFLAVCLLRQIQYHFTSEHHFGFEAAAWYWHFVDVVWLFLYVSIYWWGSMNLITTIITITITLSAVLATVSFWLPQITPD
Cutthroat_trout      YGSTFFVATGFHGLHVIIGSTFLAVCLLRQVQYHFTSEHHFGFEAAAWYWHFVDVVWLFLYVSIYWWGSMNLITTIITITITLSAVLATISFWLPQISPD
Zebrafish            YGSTFFVATGFHGLHVIIGSTFLAVCLLRQVLFHFTSDHHFGFEAAAWYWHFVDVVWLFLYVSIYWWGSMNLFATILIIMTTLSLVLALVSFWLPQMNSD
Frog                 YGSTFFVATGFHGLHVIIGSLFLSVCFIRQIQYHFTSKHHFGFEAAAWYWHFVDVVWLFLYVSIYWWGSM--TATILAIATALSLILLTVSFWLPMMTPD
Amphioxus            YGSTFFVATGFHGLHVIIGSTFLMVCLGRQVFYHYTSSHHFGFEAAAWYWHFVDVVWLFLYVCIYWWGSMLSLTYIVGIASALVIILLLVGLHLPSVMPD

                              2010      2020      2030      2040      2050      2060      2070      2080      2090      2100
                     ....|....|....|....|....|....|....|....|....|....|....|....|....|....|....|....|....|....|....|....|
Pufferfish           YQKLSPYECGFDPLGSARLPFSIRFFLVAILFLLFDLEIALLLPLPWGDQLPS-PMFTLLWASALLIMLTLGLIYEWLQGGLEWAEMTPIQFTFSSAFLL
Fugu                 YQKLSPYECGFDPLGSARLPFSLRFFLVAILFLLFDLEIALLLPLPWGDQLPS-PTLTLMWTSALLILLTIGLAYEWLQGGLEWAEMTLIQLSFTSVFFL
Stickleback          HEKLSPYECGFDPLGSARLPFSLRFFLVAILFLLFDLEIALLLPLPWGDQLAS-PLFTFLWATAVLTLLTLGLIYEWMQGGLEWAEMTPVHFAFSSAFML
Large_yellow_croaker HEKLSPYECGFDPLGTARLPFSLRFFLIAILFLLFDLEIALLLPLPWGDQLAS-PLLTFFWATAVLLLLTLGLVYEWLQGGLEWAEMTPTHFAFSAAFIL
Goldfish             AEKLSPYECGFDPLGSARLPFSLRFFLVAILFLLFDLEIALLLPLPWGDQLNN-PTGTFFWATTVLILLTLGLIYEWTQGGLEWAEMTPVHFSFSSAFIL
Medaka               YEKLSPYECGFDPVGSARLPFSIRFFLVAILFLLFDLEIALLLPLPWGDQLSS-PLMTFFWASAILMLLTLGLIYEWLQGGLEWAEMTLTHYAFSSSYFM
Salmon               AEKLSPYECGFDPLGSARLPFSLRFFLIAILFLLFDLEIALLLPLPWGDQLTT-PALTLAWSAAVLALLTLGLIYEWTQGGLEWAEMTPVHFSFTSAFIL
Rainbow_trout        AEKLSPYECGFDPLGSARLPFSLRFFLIAILFLLFDLEIALLLPLPWGDQLHT-PTLTLIWSTAVLALLTLGLIYEWTQGGLEWAEMTPVHFSFTSAFIL
sea_trout            AEKLSPYECGFDPLGSARLPFSLRFFLIAILFLLFDLEIALLLPLPWGDQLAT-PALTLAWSAAVLALLTLGLIYEWTQGGLEWAEMTPVHFSFTSAFIL
Cutthroat_trout      AEKLSPYECGFDPLGSARLPFSLRFFLIAILFLLFDLEIALLLPLPWGDQLNT-PTLTLIWSTAVLALLTLGLIYEWTQGGLEWAEMTPVHFSFTSAFIL
Zebrafish            TEKLSPYECGFDPLGSARLPFSLRFFLVAVLFPLFDLEIALLLPLPWGDQLNN-PMETLFWAMTVLILLTLGLAYEWAQGGLEWAEMTPTHFSLNAAFML
Frog                 SEKLSPYECGFDPLGSARLPFSMRFFLIAILFLLFDLEIALLLPSPWAAQLNS-PTTTIIWATLIITLLTLGLIYEWIQGGLEWAEMTLVHFSFCSAFTL
Amphioxus            NEKLSAYECGFDPMGNARLPFSLRFFLVAILFLLFDLEIALILPYPLGVVFSENTFYNYWLVMLLVVVLTFGLMYEWLKGGLEWTEMLIMIL----IFLI

                              2110      2120      2130      2140      2150      2160      2170      2180      2190      2200
                     ....|....|....|....|....|....|....|....|....|....|....|....|....|....|....|....|....|....|....|....|
Pufferfish           GLSGLAFHRTHLLSALLCLEGMMLSLFIALSLWSLQLSSISFSSAPMLLLAFSACEASVGLALMVATARTHGSDHLQGLNLLQCMLKILIPTVMLIPTAW
Fugu                 GLFGLAFYRVHLLSALLCLESMMLALFLALSTWSLQMSSTSFSAAPLLLLAFSACEAGVGLALMVATARTHGSDHLQNLNLLQCMLKILIPTTMLILATW
Stickleback          GLTGLAFHRTHLLSALLCLEGMMLSLFIALSLWTLQLDSTNFSGAPMLLLAFSACEASAGLALLVAAARTHGTDHLQNLNLLQCMLKILIPTLMLIPTTW
Large_yellow_croaker GLAGLAFHRTHLLSALLCLEGMMLSLFIALSLWTLQLDSTSLSASPMLLLAFSACEASTGLALLVATTRTHGTDRLQSLNLLQCMLKILIPTLMLIPTTW
Goldfish             GLMGLAFHRTHLLSALLCLEGMMLSLFIALALWALQFESTGFSTAPMLLLAFSACEASTGLALLVATARTHGTDRLQNLNLLQCMLKVLIPTFMLFPTIW
Medaka               SFLGLIFYRKHLLSALLCLEAMMLILFISLCLWGLILASTVFSAGPMILLAFSACEASAGLALLVAMARTHGTDRLKNLSLLRCMLMVLAPTMMLLPMIW
Salmon               GLMGLAFHRTHLLSALLCLEGMMLSLFIALSLWALQMEAIGYSVAPMLLLAFSACEASAGLALLVATARTHGTDRLQSLNLLQCMLKILIPTLMLFPTIW
Rainbow_trout        GLMGLAFHRTHLLSALLCLEGMMLSLFIALSLWALQMEATGYSVAPMLLLAFSACEASAGLALLVATARTHGTDRLQSLNLLQCMLKILIPTLMLFPTIW
sea_trout            GLMGLAFHRTHLLSALLCLEGMMLSLFIALSLWALQMEATGYSVAPMLLLAFSACEASAGLALLVATARTHGTDRLQSLNLLQCMLKILIPTLMLFPTIW
Cutthroat_trout      GLMGLAFHRTHLLSALLCLEGMMLSLFIALSLWALQMEATGYSVAPMLLLAFSACEASAGLALLVATARTHGTDRLQSLNLLQCMLKILIPTLMLFPTIW
```

```
Zebrafish            GLAGLTFHRVHLLSALLCLEGMMLSLFISMALWTLKTESMSLSTAPMLLLAFSACEASAGLALLVATARTHGSDHMKNLNLLQCMLKVLIPTIMLFPTIW
Frog                 GLTGLALHRAHLLSALLCLEGVMLSMYVGLSSWPTQISLFSFSLVPMLMLTFSACEAGTGLALMVATTRTHGTDNLHNLNLLQCMLKILIPTLMLIPSTW
Amphioxus            ALLGLGLSQTHLLSVLLCLEMMMVSLYLGLGMVSISGLHYPLMIA-LVLLTFSACEASSGLALLVLISRSHGSDLLKSFNLS--MNHLVLGYVGLVIGVI

                               2210      2220      2230      2240      2250      2260      2270      2280      2290      2300
                     ....|....|....|....|....|....|....|....|....|....|....|....|....|....|....|....|....|....|....|....|
Pufferfish           LAPAKWLWPTTLLHSLLIALASLSWLKNASETGWSSLNPYMATDPLSTPLLILSCWLLPLMILASQNHTAHEPINRQRMYISLLTSLQFFLILAFSATEM
Fugu                 LTPPKWLWPSSLLNSLLIALTSLLWLKNASETGWTFLNPYLATDPLSTPLLILSCWLLPLMILASQNHTSHEPINRQRMYITLLASLQFFLILAFSATEM
Stickleback          LTKPNWLWPSTLAHSLLIAVFSLTWFANMSETGWTSLNYYLATDSLSTPLLVLTCWLLPLMIIASQNHTAAEPCNRQRVYITLLTSLQIFLILAFSATEI
Large_yellow_croaker LTPAKWLWPTTLMHSLLIALASLSWLKSLSETGWTSLNLYMATDPLSTPLLVLTCWLLPLMILASQNHTTSEPINRQRMYITLLTSLQIFLILAFGATEM
Goldfish             LTSPKWLWTATTAHSLLIASISLMWFKWTSETGWTSSNTYLATDPLSTPLLVLTCWLLPLMILASQNHINPEPISRQRLYITLLASLQAFLIMAFGATQT
Medaka               LCSSAHLWTSALAHSMAIALISLSWLCLPMEAGWSSLNTFVATDPLSTPLLVLTCWLLPLMILASQNHMAKEPINRQRTYISLLVSLQIFLVLAFGATEM
Salmon               FSPAKWLWTTSIAQSLVIALASLSWLKWSSETGWSSSNLYLATDPLSTPLLVLTCWLLPLMVLASQNHISPEPLNRQRTYISLLVSLQMFLILAFGATEI
Rainbow_trout        LSPAKWLWTTSIAQSLIIALASLSWLKWSSETGWSSSNLYLATDPLSTPLLVLTCWLLPLMILASQSHLSPEPLNRQRAYISLLVSLQTFLVLAFGATEI
sea_trout            LSPAKWLWTTSIAQSLVIALASLSWFKWSSETGWSSSNLYLATDPLSTPLLVLTCWLLPLMILASQNHISPEPLNRQRTYISLLVSLQTFLILAFGATEI
Cutthroat_trout      LSPAKWLWTTSIAQSLAIALASLSWLKWSSETGWSSSNLYLATDPLSTPLLVLTCWLLPLMILASQNHLSPEPLNRQRAYISLLVSLQTFLILAFGATEI
Zebrafish            LSSSKWLWTTTTMNSFLIAFISLTWLKWTSDTGWNASNSYMAADPLSTPLLVLTCWLLPLMILASQNHINSEPVNRQRMYITLLASLQTFMIMAFGATKI
Frog                 LINKKWLWPSLTSQSLIISLLSLSWFFNQTETTHFS-NHLMSIDQLSTPLLILTCWLLPLMILASQNHLSTEPISRQRTFITMLVFLQLSLIMAFSATEL
Amphioxus            VTKKSMVWRVGQVGSVLLMLPATVLVN--MNMTISNVSYMMTSDFVSLGLTVLSIWLLPLMLLASQQHMVSESLIYQRVFVGCQVFLTGALVLAFMASDL

                               2310      2320      2330      2340      2350      2360      2370      2380      2390      2400
                     ....|....|....|....|....|....|....|....|....|....|....|....|....|....|....|....|....|....|....|....|
Pufferfish           IMFYVMFEATLIPTLILITRWGNQTERLNAGTYFLFYTLAGSLPLLVALLLLQNSNGSLSLLTLLHSTPPQLSTYADKIWWTGCILAFLVKMPLYGVHLW
Fugu                 IMFYVMFEATLIPTLILITRWGNQAERLNAGTYFLFYTLAGSLPLLIALLLLQNSNGSLSLLMLPHLGQLELTSYADKIWWAGCILAFLVKMPLYGVHLW
Stickleback          IMFYVMFEATLIPTLVIITRWGNQTERLNAGVYFLFYTLAGSLPLLVALLLLQNSTGTLSLLTIQYSDPFHLSTFADKLWWASCLLAFLVKMPLYGVHLW
Large_yellow_croaker IMFYVMFEATLIPTLFLITRWGNQAERLNAGTYFLFYTLAGSLPLLVALLLLQNSTGTLSLLTLQLSGPTMLISFADKLWWAGCLLAFLVKMPLYGVHLW
Goldfish             IMFYIMFEATLIPTLIIITRWGNQTERLNAGTYFLFYTLAGSLPLLVALLLLQQSTGTLSMLVLQYSQPLQLNSWGHMIWWAGCLIAFLVKMPLYGVHLW
Medaka               IMFYIMFEATLIPTLIIITRWGNQTERLNAGIYFLFYTLAGSLPLLVALLLLQNSTGSLSFLTTQFFPPLQLHTEASKFWWAGCLLAFLVKMPLYGAHLW
Salmon               IMFYIMFEATLLPTLIIITRWGNQTERLNAGTYFLFYTLAGSLPLLVALLLLQNDSGTLSMFTLQYTQPMHLLTWGNKLWWAACLLAFLVKMPVYGVHLW
Rainbow_trout        IMFYVMFEATLLPTLIIITRWGNQTERLNAGTYFLFYTLAGSLPLLVALLLMQNDNGTLSMFTLQYTQPLHLLTWGDKLWWAACLLAFLVKMPLYGVHLW
sea_trout            IMFYIMFEATLLPTLIIITRWGNQTERLNAGTYFLFYTLAGSLPLLVALLLLQNDSGTLSMFTLQYTQPLHLLTWGDKLWWAACLLAFLVKMPLYGVHLW
Cutthroat_trout      IMFYIMFEATLLPTLIIITRWGNQTERLNAGTYFLFYTLAGSLPLLVALLLMQNDNGTLSMFTLQYTQPLHLLTWGDKLWWAACLLAFLVKMPLYGVHLW
Zebrafish            IMFYIMFEATLIPTLIIITRWGNQAERLNAGTYFLFYTLAGSLPLLVALLLLQQSTGTLSMLVLQYSDPLLLNSWGHKIWWAGCLIAFLVKMPLYGMHLW
Frog                 ILFYIMFEITLIPTLIIITRWGNQAERLNAGTYFLFYTLAGSLPLLVALLSLYSYTGTLSLSLLQLLPNHIPLSWANKLWWFACLLAFMVKMPLYGTHLW
Amphioxus            LLFYIAFESTLLPTLMLITRWGAQKERYQAGTYFMFFTLVGSLPLLICLIGQYQMVGSLALDLS--YEGVFQLSYLVNFWWVGCILAFLVKLPLYGVHLW

                               2410      2420      2430      2440      2450      2460      2470      2480      2490      2500
                     ....|....|....|....|....|....|....|....|....|....|....|....|....|....|....|....|....|....|....|....|
Pufferfish           LPKAHVEAPIAGSMVLAAVLLKLGGYGMMRILMTLEPLTKELSYPFIILALWGVIMTGSICMRQTDLKSLIAYSSVSHMGLVVGGILIQTPWGFTGALIL
Fugu                 LPKAHVEAPIAGSMVLAAVLLKLGGYGMMRILVTLDPLTKELSYPFIVLALWGVIMTGSICMRQTDLKSLIAYSSVSHMGLVVGGILIQTPWGFTGALIL
Stickleback          LPKAHVEAPVAGSMILAAVLLKLGGYGMIRMIPILDPLTKELGYPFIIFALWGVVMTGSICLRQTDLKSLIAYSSVSHMGLVVAAILIQTPWSLSGALIL
Large_yellow_croaker LPKAHVEAPIAGSMILAAVLLKLGGYGMMRILPMLEPLTKELSYPFIIFALWGVIMTGSICLRQTDLKSLIAYSSVGHMGLVVGGILIQTPWGLTGALIL
Goldfish             LPKAHVEAPVAGSMVLAAVLLKLGGYGMMRMMVMLDPLSKELAYPFIILALWGIIMTGSICLRQTDLKSLIAYSSVSHMGLVAGGILIQTPWGFSGAIIL
Medaka               LPKAHVEAPIAGSMVLAAVLLKLGGYGMMRIIIILDPLTKQLSYPFIILALWGVVMTGSICLRQTDLKSLIAYSSVSHMGLVAAGILIQTPWGLTGALIL
Salmon               LPKAHVEAPIAGSMILAAVLLKLGGYGMMRMMVMLDPLTKELAYPFIVLALWGIIMTGSICLRQTDLKSLIAYSSVGHMGLVAGGILIQTPWGFTGAIIL
Rainbow_trout        LPKAHVEAPIAGSMILAAVLLKLGGYGMMRMMVMLDPLTKELAYPFIVLALWGIIMTGSICLRQTDLKSLIAYSSVGHMGLVAGGILIQTPWGFTGAIIL
sea_trout            LPKAHVEAPIAGSMILAAVLLKLGGYGMMRMMVMLDPLTKELAYPFIVLALWGIIMTGSICLRQTDLKSLIAYSSVGHMGLVAGGILIQTPWGFTGAIIL
Cutthroat_trout      LPKAHVEAPIAGSMILAAVLLKLGGYGMMRMMVMLDPLTKELAYPFIVLALWGVIMTGSICLRQTDLKSLIAYSSVGHMGLVAGGILIQTPWGFTGAIIL
Zebrafish            LPKAHVEAPVAGSMILAAVLLKLGGYGMMRMMVMLDPLSKQLAYPFIILALWGVIMTGLVCLRQTDLKSLIAYSSVGHMGLVAGGILIQTPWGFTGAIIL
Frog                 LPKAHVEAPIAGSMVLAAILLKLGGYGIIRISITLTPAMKDLAYPFLILSLWGIIMTSSICLRQTDLKSMIAYSSVSHMGLVISAAMIQTPWSLTGAMIL
Amphioxus            LPKAHVEAPIAGSMVLAGVLLKLGGYGMMRVSLMWGATAMLSSEVFLALALWGIVVMGGICLRQTDLKSLIAYSSVGHMALVVGGVLTGVAWGYNGAMVL

                               2510      2520      2530      2540      2550      2560      2570      2580      2590      2600
                     ....|....|....|....|....|....|....|....|....|....|....|....|....|....|....|....|....|....|....|....|
Pufferfish           MIAHGLTSSALFCLANTNYERTHSRTMLLARGLQMALPLMTAWWFITSLANLALPPLPNLMGELMIVTSLFNWSWWTIALTGLGMLITASYSLYMFLMTQ
Fugu                 MIAHGLTSSALFCLANTSYERTHSRTMLLARGMQMILPLMAAWWFIASLANLALPPLPNLMGELMIITSLFNWSPWTIGLTGLGTLITAGYSLYMFLMTQ
Stickleback          MIAHGLASSALFCLANTNYERTHSRTLLLGRGLQMALPLMTTWWFIASLANLGLPPLPNLMGELMIITSLFSWSWWTLALTGAGTLITVSYSLYMFIMTQ
Large_yellow_croaker MIAHGLTSSALFCLANTNYERTHSRTMALARGLQMALPLMAAWWFIASLANLALPPLPNLMAELMIITSLFNWSWWTLALTGTGTLITAGYSLYMFLMTQ
```

```
Goldfish             MIAHGLVSSALFCLANTAYERTHSRTMILARGLQIIFPLTAVWWFIANLANLALPPLPNLMGELMIITTLFNWSPWTILLTGLGTLITAGYSLYMFLMSQ
Medaka               MIAHGLTSSALFCLANTNYERTHSRTMLLARGLQMILPLLATWWFLFTLANLALPPLPNLMGELMIITSLFNWSNWTLLLTGAGTLITASYSLHMFLTTQ
Salmon               MIAHGLASSALFCLANTSYERTHSRTMLLARGMQMILPLMTTWWFVASLANLALPPLPNLMGELMIITSMFNWSHWTLLLTGLGTLITASYSLYLFLMTQ
Rainbow_trout        MIAHGLASSALFCLANTSYERTHSRTMLLARGMQMILPLMTTWWFVASLANLALPPLPNLMGELMIITSMFNWSYWTLILTGLGTLITASYSLYLFLMTQ
sea_trout            MIAHGLASSALFCLANTSYERTHSRTMLLARGMQMILPLMTTWWFVASLANLALPPLPNLMGELMIITSMFNWSHWTLLLTGLGTLITASYSLYLFLMTQ
Cutthroat_trout      MIAHGLASSALFCLANTSYERTHSRTMLLARGMQMILPLMTTWWFMASLANLALPPLPNLMGELMIITSMFNWSYWTLILTGLGTLITASYSLYLFLMTQ
Zebrafish            MIAHGLTSSALFCLANTSYERTHSRTMILARGLQMVLPLATVWWFIANLANLALPPLPNLMGELMIITALFNWSPWTIIITGMGTLITANYSLYMFLTSQ
Frog                 MIAHGLISSALFCLANTNYERTHSRALLLSRGLQSILPLMGTWWLLSNLANMALPPSPNLMGEITIMTALFNWSNWTIILTGIGTLLTASYSLYMFLITQ
Amphioxus            MIAHGLVSSCLFCLANLWYERSSTRNLSGSRGLIMIFPLISSGWFLMSLMNMALPPAINLFGELVAMVALYNWSPYSIVYMSLGAVLTAAYSLYLFGMSQ
```

```
                              2610      2620      2630      2640      2650      2660      2670      2680      2690      2700
                     ....|....|....|....|....|....|....|....|....|....|....|....|....|....|....|....|....|....|....|....|
Pufferfish           RGPLPSHIMALEPSHTREHLLIALHLLPLLLLILKPELIWGWAV-MHSTPLIMTSSLVIIFTLLIYPILTTLSPSPQSPDWALKQVKTAVKLAFLVSLLP
Fugu                 RGPAPSHLLALEPSHTREHLLIALHLLPLILIITKPELIWGWTA-MHSTPLIMTSTLIIIFALLIYPVLTTFSPKPQNPNWALIQVKTAVKYAFLVSLLP
Stickleback          RGPLPAHMIALDPSHTREHLIMALHLIPLLLLVLKPELVWGWAT-MWQTPSVLYSGMIIILVVLSFALLSALVQGNSVLNQTVPNITSAVKLSFFISLLP
Large_yellow_croaker RGPLPAHIMALEPSHSREHLLAALHLLPLILLILKPELIWGWTA-MHTTSLIMTSSLITMLLLLAYPVLTTLSPNPQGPDWSLSQVKTAVKLAFFVSLLP
Goldfish             RGPTPNHITGLQPFHTREHLLMTLHLVPVILLVTKPELMWGWCY---MT-LIMHSSLLLIFFILVYPLLTTLNPNQQG-SNMAGMTKIAVSSAFFISLLP
Medaka               RGPVTSPVVAIEPTHTREHLLMVLHLLPLLLLVLKPTLIWGWTL-MHLTTMVLSSSLLTIFSILILPVLGTLNPSPPGSLWATNSVKTAVKVAFFVSLLP
Salmon               RGPLPSHIIALEPTHTREHLLITLHLIPIILLILKPELMWGWCF-MHPTTLILSSTLLMIFALLLYPLITTLNPTPQQENWALTHVKTAIKMAFLVSLLP
Rainbow_trout        RGPLPSHIIALEPTHTREHLLIILHLIPIVLLILKPELMWGWCF-MHPTTLILSSSLLMIFTLLIYPLITTLTPTPQHKNWALTHVKTAIKMAFLVSLLP
sea_trout            RGPLPSHIIALEPTHTREHLLITLHLIPIILLILKPELMWGWCF-MHPTTLILSSTLLMIFALLLYPLITTLNPSPQQENWALTHVKTAVKMAFLVSLLP
Cutthroat_trout      RGPLPSHVIALEPTHTREHLLIILHLTPIALLILKPELMWGWCFCMHPTTLILSSSLLMIFALLIYPLVTTLTPTPQHKNWALTHVKTAIKMAFLVSLLP
Zebrafish            RGSIPEHITNLSPSHTREHLLMTLHLIPIILLMLKPELMWGWCN---MTDMIMPLTLILIFAVLSYPLLKPKSYSKSN-NSFQAWNAVHVS--FLISLIP
Frog                 RGPTPEHLTAITPTHTREHTLMTLHLIPIIPLMMKPELIWGLFF--MNLPLIFNSSLMITITTLLTPIL--LASTSLNTQHLHKMIKTAVKTSFFISLIP
Amphioxus            WGNTMKNYKNLYTITSREYLLTTLHLVPAIYLIFYLGLMFMLEL----WGVLSLTSLGVMVIFLFSKIKSSFAESVKYAGYMN------------AVLL
```

```
                              2710      2720      2730      2740      2750      2760      2770      2780      2790      2800
                     ....|....|....|....|....|....|....|....|....|....|....|....|....|....|....|....|....|....|....|....|
Pufferfish           LFLFLNEGAEAVITNWSWMNTHTFDINISLKFDHYSIIFTPVALYVTWSILEFASWYMHADPFMNRFFKYLLTFLIAMIILVTANNMFQLFIGWEGVGIM
Fugu                 LCLHLNEGTESIITNLNWMNTLTFDINISLKFDSYSIIFTPVALYVTWSILEFASWYMHSDPFMNRFFKYLLVFLIAMIILVTANNMFQLFIGWEGVGIM
Stickleback          LFMFFNEGAETIVSSWTWMNTTCFEINLSFKFDQYSVIFTTVALYVTWSILEFASWYMHSDPNISRFFKYLLIFLMAMLTLVTANNMFQLFIGWEGVGIM
Large_yellow_croaker LFLFLNEGAETIVTNWTWMNTLTFDINISFKFDHYSIIFTPIALYVTWSILEFASWYMHADPNMNRFFKYLLIFLIAMVILVTANNMFQLFIGWEGVGIM
Goldfish             LMIFLNLKTEGIITNWQWMNTQTFDVNISFKFDHYSLIFVPIALYVTWSILEFALWYMHSDPYIDRFFKYLLTFLVAMIILVTANNMFQLFIGWEGVGIM
Medaka               LFIFLNEGVEAIMTNWEWMNTLMFNINISFKFDLYSIVFTPVALYVTWSILEFASWYMHDDPNMNRFFKYLLIFLIAMIVLVTANNMFQLFIGWEGVGIM
Salmon               LFIFLDQGTETIVTNWQWMNTTTFDINLSFKFDHYSIIFTPIALYVTWSILEFASWYMHADPNMNRFFKYLLLFLIAMIILVTANNMFQLFIGWEGVGIM
Rainbow_trout        LFVFLDQGTETIVTNWQWMNTTTFDINLSFKFDHYSIIFTPIALYVTWSILEFASWYMHADPNMNRFFKYLLLFLIAMIILVTANNMFQLFIGWEGVGIM
sea_trout            LFIFLDQGTETIVTNWQWMNTTTFDINLSFKFDHYSIIFTPIALYVTWSILEFASWYMHADPNVNRFFKYLLLFLIAMIILVTANNMFQLFIGWEGVGIM
Cutthroat_trout      LFVFLDQGTETIVTNWQWMNTTTFDINLSFKFDHYSIIFTPIALYVTWSILEFASWYMHADPNMNRFFKYLLLFLIAMIILVTANNMFQLFIGWEGVGIM
Zebrafish            LTMMLYKESDHVVMCWSWMNTQAFNVDLSFKFDYYSVTFTSIALFITWSILEFASWYMASYPQKELFYKYLLLFLMSMIILVTANNLFQLFIGWEGVGIM
Frog                 LSIFLDQGLESITTNFHWMNINSFDINMSFKFDIYSSIFLPIALFVTWSILEFATWYMASDPLITRFFKYLLTFLVAMVILVTANNFFQLFIGWEGVGIM
Amphioxus            SILLMSDESEMLFLKWEWVKLGGYSLMISFRFDLYTCCFFVVGLYVTWNILMFSFYYMSTDPRIDLFCKYLGLFLIAMLLLVSAESLFQLLIGWEGVGIM
```

```
                              2810      2820      2830      2840      2850      2860      2870      2880      2890      2900
                     ....|....|....|....|....|....|....|....|....|....|....|....|....|....|....|....|....|....|....|....|
Pufferfish           SFLLIGWWYARADANTAALQAVLYNRVGDIGLVFSMAWMATHLNSWEIQQIFFSSKDMDLTLPLIALILAATGKSAQFGLHPWLPSAMEGPTPVSALLHS
Fugu                 SFLLIGWWYARADANTAALQAVIYNRVGDIGLIIAMAWMATHLNSWELQQIFFSTKNMDMTLPLVALILAATGKSAQFGLHPWLPSAMEGPTPVSALLHS
Stickleback          SFLLIGWWGGRADANTAALQAVVYNRIGDIGLIFAMAWMATKLNSWEIQQIFVASKDFDLTFPLLGLIVAAAGKSAQFGLHPWLPSAMEGPTPVSALLHS
Large_yellow_croaker SFLLIGWWYGRADANTAALQAVLYNRVGDIGLIFAMAWMATNLNSWEMQQMFTTAKNQDLTFPLLGLILAATGKSAQFGLHPWLPSAMEGPTPVSALLHS
Goldfish             SFLLIGWWHGRADANTAALQAVIYNRVGDIGLIMTMAWFAMNLNSWEIQQIFVLSKNFDLTIPLMGLALTATGKSAQFGLHPWLPSAMEGPTPVSALLHS
Medaka               SFLLIGWWFGRADANTAALQAVVYNRVGDIGLILAMAWMAVNLNSWDMQQLFSMTTNQDMTLPLLGLVLAATGKSAQFGLHPWLPSAMEGPTPVSALLHS
Salmon               SFLLIGWWYGRADANTAAMQAVIYNRVGDIGLILSMAWFATNLNSWEIQQMFASSKELDLTLPLMGLILAATGKSAQFGLHPWLPSAMEGPTPVSALLHS
Rainbow_trout        SFLLIGWWHGRADANTAAMQAVIYNRVGDIGLILSMAWFATNLNSWEIQQMFASSKGLDLTLPLMGLILAATGKSAQFGLHPWLPSAMEGPTPVSALLHS
sea_trout            SFLLIGWWYGRADANTAAMQAVIYNRVGDIGLILSMAWFATNLNSWEIQQMFASSKELDLTLPLMGLILAATGKSAQFGLHPWLPSAMEGPTPVSALLHS
Cutthroat_trout      SFLLIGWWHGRADANTAAMQAVIYNRVGDIGLILSMAWFATNLNSWEIQQMFASSKGLDLTLPLMGLILAATGKSAQFGLHPWLPSAMEGPTPVSALLHS
Zebrafish            SFLLIGWWFGRTEANTASLQAVIYNRMGDIGFILTLAWMAMYLNSWDIQQIFILSKDFDMTIPQIGLIIAATGKSAQFTLHPWLPSAMEGPTPVSALLHS
Frog                 SFLLIGWWYARADANTAALQAVIYNRVGDIGLILSMAWVAMNLNSWEMQQVFMLNPE-NLTLPLLGLILAATGKSAQFGLHPWLPAAMEGPTPVSALLHS
Amphioxus            SYLLISWWYARSDANTAALQAIFYNRVGDIGLLIMLMWSLVTLGDWSFTGLYA--LDFVNTFFLLGVVLAAAGKSAQLGLHPWLPAAMEGPTPVSSLLHS
```

```
                              2910      2920      2930      2940      2950      2960      2970      2980      2990      3000
                      ....|....|....|....|....|....|....|....|....|....|....|....|....|....|....|....|....|....|....|....|
Pufferfish            STMVVAGIFLLIRTSPLMENNPTALTLCLCLGALTTLFTATCALTQNDIKKIVAFSTSSQLGLMMVTIG-LNQPQLAFLHICTHAFFKAMLFLCSGSIIH
Fugu                  STMVVAGIFLMIRISPLLETNPTALTLCLCLGALTTLFTATCALTQNDIKKIVAFSTSSQLGLMMVTIG-LNQPQLAFLHICTHAFFKAMLFLCSGSIIH
Stickleback           STMVVAGIFLLVRLSPLLEGNQTALTICLCLGALTTLFTATCALTQNDIKKIVAFSTSSQLGLMMVAIG-LNQPHLAFLHICTHAFFKAMLFLCSGSVIH
Large_yellow_croaker  STMVVAGIFLLIRMSPLLENNQTALTLCLCLGALTTLFTATCALTQNDIKKIVAFSTSSQLGLMMVTIG-LNQPQLAFLHICTHAFFKAMLFLCSGSIIH
Goldfish              STMVVAGIFLFIRLHPLMENNQLALTTCLCLGALTSLFTATCALTQNDIKKIVAFSTSSQLGLMMVTIG-LNQPQLAFLHICTHAFFKAMLFLCSGSIIH
Medaka                STMVVAGIFLLIRLNPLMDNNPLVLSTCLCLGALTTVFTATCALTQNDIKKIIAFSTSSQLGLMMVTIG-LNQPQLAFLHICTHAFFKAMLFLCSGSIIH
Salmon                STMVVAGIFLLIRLHPLMENNQTALTTCLCLGALTTLFTATCALTQNDIKKIVAFSTSSQLGLMMVTIG-LNQPQLAFLHICTHAFFKAMLFLCSGSIIH
Rainbow_trout         STMVVAGIFLLIRLHPLMEDNQTALTVCLCLGALTTLFTATCALTQNDIKKIVAFSTSSQLGLMMVTIG-LNQPQLAFLHICTHAFFKAMLFLCSGSIIH
sea_trout             STMVVAGIFLLIRLHPLMENNQTALTTCLCLGALTTLFTATCALTQNDIKKIVAFSTSSQLGLMMVTIG-LNQPQLAFLHICTHAFFKAMLFLCSGSIIH
Cutthroat_trout       STMVVAGIFLLIRLHPLMEDNQTALTTCLCLGALTTLFTATCALTQNDIKKIIAFSTSSQLGLMMVTIG-LNQPQLAFLHICTHAFFKAMLFLCSGSIIH
Zebrafish             STMVVAGIFLLIRLHPIMETNKLAPTICLCLGALTTLFAATCALTQNDIKKIVAFSTSSQLGLMMVAIG-LNQPHLAFFHICTHAFFKAMLFLCSGAIIH
Frog                  STMVVAGIFLLIRIHPMIQNNNTALTICLCLGAITTLFTAACALTQNDIKKIVAFSTSSQLGLMMVTIG-LNLPQLAFFHICTHAFFKAMLFLCSGSIIH
Amphioxus             STMVVAGVFLLIRFSPIILNHKEIQLMVFFLGTMTTLFSAICALAQNDMKKVVAFSTASQLGLMVTVVAGAGAPQLAFLHICMHAFFKAMLFMCSGGFIH

                              3010      3020      3030      3040      3050      3060      3070      3080      3090      3100
                      ....|....|....|....|....|....|....|....|....|....|....|....|....|....|....|....|....|....|....|....|
Pufferfish            SLNDEQDIRKMGGMHHLTPFTSSCLTIGSLALTGTPFLAGFFSKDAIIEALNTSYLNAWALSLTLLATSFTAIYSLRVVFFVSMGHPRFNTFSPINENNP
Fugu                  SLNDEQDIRKMGGMHHLTPVTSSCLTIGSLALTGTPFLAGFFSKDAIIESLTTSQLNAWALCLTLLATSFTAIYSLRVVFYVSMGHPRFNSLSPINENNP
Stickleback           SLNDEQDIRKMGGMHFLTPFTSSCLTIGSLALAGTPFLAGFFSKDAILEALNTSHLNAWALVLTLLATSFTAIYSLRVIFFVSMGYPRFNSLSPINENNP
Large_yellow_croaker  SLNDEQDIRKMGGMHHLTPFTSSCLTIGSLALTGTPFLAGFFSKDAIIEALNTSHLNAWALALTLLATSFTAIYSLRVVFFVSMGHPRFNTLSPINENNP
Goldfish              SLNDEQDIRKMGGLFNIMPATSTYFTIGSLALTGTPFLAGFFSKDAIIEALNTSHLNAWALTLTLIATSFTAVYSFRLVYFVVMGTPRFLALSPINENNP
Medaka                SLNDEQDIRKMGGLHHLLPFTSSCLTIGSLALTGTPFLAGFFSKDAIIEALNTSYLNAWALVLTLVATSFTAVYSLRVVFFVSMGHPRFNPISPINENNL
Salmon                SLNDEQDIRKMGGMHNLTPFTSSCLTIGSLALTGTPFLAGFFSKDAIIEALNTSHLNAWALTLTLLATSFTAVYSLRVVFFVSMGHPRFTATAPINENNP
Rainbow_trout         SLNDEQDIRKMGGMHNLTPSTSSCLTIGSLALTGTPFLAGFFSKDAIIEALNTSHLNAWALTLTLLATSFTAIYSLRVIFFVSMGHPRFTATAPVNENNP
sea_trout             SLNDEQDIRKMGGMHNLTPFTSSCLTIGSLALTGTPFLAGFFSKDAIIEALNTSHLNAWALTLTLLATSFTAIYSLRVVFFVSMGHPRFTATAPINENNP
Cutthroat_trout       SLNDEQDIRKMGGMHNLTPLTSSCLTIGSLALTGTPFLAGFFSKDAIIEALNTSHLNAWALTLTLLATSFTAVYSLRVIFFVSMGHPRFTATAPINENNP
Zebrafish             SLNNEQDIRKMGGTYHTLPMTTNYLTIGKMALMGTPFLAGFFSKDAILEAMTTSHLNAWALTLTLIATSFTAVYSFRMIYLVCLGSPRHKTYETIDEN-H
Frog                  SLNDEQDIRKMGGLQNSLPTTTTCLTIGSLALTGTPFLAGFFSKDAIIEALNTSQTNAWALALTLIATSFTAVYSFRIIFFASMGHPRSNPLSPINENNP
Amphioxus             GLQNEQDVRKMGGLHSAAPITSVCFFIGSAALMGVPFLAGFFSKDPIIEIININNLNSWAVGLVLIATSFTAAYSVRLLYFSVGGVSRMLVLQPMNEEYG

                              3110      3120      3130      3140      3150      3160      3170      3180      3190      3200
                      ....|....|....|....|....|....|....|....|....|....|....|....|....|....|....|....|....|....|....|....|
Pufferfish            AVINPIKRLAWGSILAGLLITANILPMKTPVMSMPPLLKLAALAVTLLGLLTALELASLTTKQIKTTPHLTPHHFSNMLGFFPSIIHRLSPKMNLILGQT
Fugu                  SVINPIKRLAWGSIIAGLLITTNLLPTKTPVMSMPMVVKLTALIVTILGLLIALELASLTSKQLKPTPHLSPHHFSNMLGFFPTIVHRASPKINLILGQT
Stickleback           AVINPIKRLAWGSIIAGFLITSTIIPMKTPIITMPPVLKLAALIVSVLGLLIALELASLTAKQYHPTPRLTPHHFSNMLGFFPSVIHRLTPKFGLTLGQT
Large_yellow_croaker  AVLNPIKRLAWGSIIAGLLITSNMTPLKTPIMTMPPLLKLAALTVTILGLLLALELASLTNKQFKPSPKLTPHHFSNMLGFFPAIIHRTLPKLNLTLGQT
Goldfish              LVINSIKRLAWGSIIAGLIITQNFLPMKTPIMTMPAPLKMAALLVTIAGLLVAMELANMTSKQVKIIPMIPLHHFSNMLGFFPAIIHRLLPKLKLTLGQS
Medaka                TVMNPIKRLAWGSILAGLLITANISPLKTPLMTMPFVLKMAALTVTIVGLLMALELASLASQQFKIKPASAPHHFSNMLGFYPSVVHRLAPKTNLVLGQL
Salmon                SVINPIKRLAWGSIIAGLLITSNFLPSKTPIMTMPLPLKLAALLVTISGLLIALELASLTNKQFKTTPNLITHNFSNMLGFFPAIIHRLAPKLNLTLGQT
Rainbow_trout         SVINPIKRLAWGSIIAGLLITSNFLPTNTPVMTMPTHLKLAALLVTISGLLIALELASLTNKQFKLHPTLTLHNFSNMLGFFPAIIHRLTPKLNLTLGQT
sea_trout             SVINPIKRLAWGSIIAGLVITSNFLPSKTPVMTMPLPLKLAALLVTISGLLIALELASLTNKQFKTTPNLATHNFSNMLGFFPTIIHRLAPKLNLVLGQT
Cutthroat_trout       SVINPIKRLAWGSIIAGLLITSNFLPTNTPVMTMPTHLKLAALLVTISGLLIALELASLTNKQFKTTPNLILHNFSNMLGFFPAIIHRLAPKLNLTLGQT
Zebrafish             IPTNTIQRLAWGSIIAGLIISYTMIPLKTPILTMPIYLKLAAILVTLLGIILGLEISTLANKINKNTTPGIPFHFSISLIFFP-ILHRLIPMRKLFMGES
Frog                  TVMNPIKRLAWGSIISGLVIASNMLPIKSPIMTMPLMAKQAAILVTMAGLIIAIDLANLTTSINTPT-KTKVHSFSNLLGFFPLIIHRLTPKTNLNMAQK
Amphioxus             NLIGPLQRLAYSSVIAGVVFIYFLSPNQISCLSLPLSLKLAAVFVTLVGGLIAWDVVNLLHRE-ESVTNIPELAFEAQVGFYPLIMHKLIPKVWLNMGEM

                              3210      3220      3230      3240      3250      3260      3270      3280      3290      3300
                      ....|....|....|....|....|....|....|....|....|....|....|....|....|....|....|....|....|....|....|....|
Pufferfish            IASQTIDLTWLEKIGPKATANLNTPLVSTISNVQQGSIKTFMALFLLTLAFSALALLT--MASLRKTHPLMKIANDMVVDLPTPSNISAWWNFGSLLGLC
Fugu                  IATQIIDLTWLEKVGPKTISSINTPLISTISNIQQGSIKTYLVLFLTTLALSTLVLLT--MASLRKTHPLLKIVNDMVIDLPTPSNISAWWNFGSLLGLC
Stickleback           IASQMLDQTWIEKIGPKAIVYHTTPLITTTSNTQRGLVKTYLALFLLTLALAMLLASF--MASLRKTHPLLKIANNALVDLPAPSNISVWWNFGSLLGLC
Large_yellow_croaker  IATQIIDLTWLEKTGPKAMTSLNMPLIKSTSNAQQGMIKTYLSFFLLTLALTTLVFVL--MANLRKTHPLLKIANDALVDLPAPSNISAWWNFGSLLALC
Goldfish              AATQ-LDKTWLEAMGPKGLALTQMTMAKVTNDISRGMIKTYLTIFLLTLILAILPVLL--MASLRKTHPLIKIANDALVDLPTPSNISAWWNFGSLLGLC
Medaka                IANQTIDQTWLEKTGPKMTASVNLPLISSTSNLQQGVIKTYFLMFFFTMILAVLILVI--MANLRKTHPLLKIANDALVDLPAPSNISVWWNFGSLLGLC
Salmon                IASQMVDQTWFEKIGPKGVVSTHLPMVTTTSNIQQGMIKTYLTLFFLSTALAVLLTLT--MANLRKTHPLLKIANDALVDLPAPSNISVWWNFGSLLGLC
Rainbow_trout         IASQMVDHTWFEKVGPKELFQLTCLMVTTTSNIQQGMIKTYLTLFFLSTTLAVLLTLT--MANLRKTHPLLKIANDALVDLPAPSNISVWWNFGSLLGLC
```

```
sea_trout             IASQMVDQTWFEKIGPKGIVSTHLPMVTTTSNIQQGMIKTYLTLFFLSTTLAVLLTLT--MANLRKTHPLLKIANDALVDLPAPSNISVWWNFGSLLGLC
Cutthroat_trout       IASQMVDQTWFEKVGPKGIVSTHLPMVTTTSNIQQGMIKTYLTLFFLSTTLAVLLTLA--MANLRKTHPLLKIANDALVDLPAPSNISVWWNFGSLLGLC
Zebrafish             AATK-IEKSWSELFGPCGIAFTLMTVATFVKDHRMASIKSYLAVFLTSIILKMMMLKLYTMTSLRKTHPVLKIANDALVDLPTPLNISAWWNFGSLLGLC
Frog                  IATHIVDMSWYEKAGPQGLANQQLPMIKTTSNIQQGLIKTYLTLFLMTSAILISLLMA---PNIRKSHPLIKIVNNSFIDLPTPSNLSSWWNFGSLLGVC
Amphioxus             YQMQVMDRGWTELALPQGLGGNYKIMADNVVNAQTSLIKMYIAVMVMMGGLILGIMICLMSGPLRKHHPLLKVVNHSVIDLPVPSNISVMWNFGSLLGLC

                               3310      3320      3330      3340      3350      3360      3370      3380      3390      3400
                      ....|....|....|....|....|....|....|....|....|....|....|....|....|....|....|....|....|....|....|....|
Pufferfish            LVAQILTGLFLAMHYTSDIATAFSSVAHICRDVNYGWLIRNLHANGASFFFICLYLHIGRGLYYGSYLQKETWNIGVVLLLLVMATAFVGYVLPWGQMSF
Fugu                  LITQIITGLFLAMHYTSDISTAFSSVAHICRDVNYGWLIRNLHANGASFFFICLYSHIGRGLYYGSYLSKETWNVGVVLLLLVMATAFVGYVLPWGQMSF
Stickleback           LIIQILTGLFLAMHYTSDIATAFSSVGHICRDVNYGWLIRNLHANGASFFFICIYMHIGRGLYYGSYLYKETWNIGVVLLLLVMMTAFVGYVLPWGQMSF
Large_yellow_croaker  LGAQILTGLFLAMHYTSDVTMAFSSVAHICRDVNYGWLIRNLHANGASFFFICLYLHIGRGLYYGSYLYKETWNIGVVLFLLVMMTAFVGYVLPWGQMSF
Goldfish              LITQILTGLFLAMHYTSDISTAFSSVTHICRDVNYGWLIRNIHANGASFFFICIYMHIGRGLYYGSYLNKETWNIGVVLLLLVMMTAFVGYVLPWGQMSF
Medaka                LAAQIITGLFLAMHYTSDIATAFSSVAHICRDVNYGWLIRNMHANGASFFFICIYLHIGRGLYYGSYLYKETWNVGVILLLLVMMTAFVGYVLPWGQMSF
Salmon                LATQILTGLFLAMHYTSDISTAFSSVCHICRDVSYGWLIRNIHANGASFFFICIYMHIARGLYYGSYLYKETWNIGVVLLLLTMMTAFVGYVLPWGQMSF
Rainbow_trout         LATQILTGLFLAMHYTSDISTAFSSVCHICRDVSYGWLIRNIHANGASFFFICIYMHIARGLYYGSYLYKETWNIGVVLLLLTMMTAFVGYVLPWGQMSF
sea_trout             LATQILTGLFLAMHYTSDISTAFSSVCHICRDVSYGWLIRNIHANGASFFFICIYMHIARGLYYGSYLYKETWNIGVVLLLLTMMTAFVGYVLPWGQMSF
Cutthroat_trout       LATQILTGLFLAMHYTSDISTAFSSVCHICRDVSYGWLIRNIHANGASFFFICIYMHIARGLYYGSYLYKETWNIGVVLLLLTMMTAFVGYVLPWGQMSF
Zebrafish             LITQILTGLFLAMHYTSDISTAFSSVVHICRDVNFGWLIRSIHANGASFFFICLYIHIARGLYYGSYLYNETWNIGVVLFLLVMMTAFVGYVLPWGQMSF
Frog                  LIAQIATGLFLAMHYTADTSMAFSSVAHICRDVNYGWLIRNLHANGASFFFICIYLHIGRGLYYGSFLYKETWNIGVILLFLVMATAFVGYVLPWGQMSF
Amphioxus             LVSQILTGLFLAMHYTADVNLAFSSVAHICRDVNYGWLLRNLHANGASFMFICLYMHIGRGLYYGSYFYRETWNIGVMLLVLTMATAFLGYVLPWGQMSF

                               3410      3420      3430      3440      3450      3460      3470      3480      3490      3500
                      ....|....|....|....|....|....|....|....|....|....|....|....|....|....|....|....|....|....|....|....|
Pufferfish            WGATVITNLLSAVPYVGNTLVQWVWGGFSVDHATLTRFFAFHFLLPFIVAAAVIVHLIFLHETGSNNPLGLNSDTDKIPFHPYFSYKDLIGFTVLLTTLT
Fugu                  WGATVITNLLSAVPYVGNTLVQWVWGGFSVDSATLTRFFAFHFLLPFIVAAAAIVHLIFLHETGSNNPLGLNSNADKIPFHPYFSYKDLLGFTIMLSALA
Stickleback           WGATVITNLLSAVPYVGNSLVQWIWGGFSVDNATLTRFFAFHFLFPFVIAGATLVHLLFLHQTGSNNPLGLNSDADKISFHPYFSYKDLLGFAALLIALT
Large_yellow_croaker  WGATVITNLLSAVPYVGNTLVQWIWGGFSVDNATLTRFFAFHFLLPFIVAAATFLHLLFLHETGSNNPLGLNSDMDKIPFHPYFTYKDLLGFAILIICLT
Goldfish              WGATVITNLLSAVPYMGDMLVQWIWGGFSVDNATLTRFFAFHFLLPFIIAAATVIHLLFLHETGSNNPIGLNSDADKISFHPYFSYKDLLGFVIMLLALT
Medaka                WGATVITNLLSAVPYVGNALVQWIWGGFSVDNATLTRFFAFHFLLPFVIAAATVVHLIFLHETGSNNPTGLNSDSDKVSFHPYFSYKDLLGFAALLVALI
Salmon                WGATVITNLLSAVPYVGGALVQWIWGGFSVDNATLTRFFAFHFLFPFVIAAATVLHLLFLHETGSNNPAGINSDADKISFHPYFSYKDLLGFVAMLLGLT
Rainbow_trout         WGATVITNLLSAVPYVGGALVQWIWGGFSVDNATLTRFFAFHFLFPFVIAAATVLHLLFLHETGSNNPAGINSDADKISFHPYFSYKDLLGFVAMLLGLT
sea_trout             WGATVITNLLSAVPYVGGALVQWIWGGFSVDNATLTRFFAFHFLFPFVIAAATVLHLLFLHETGSNNPAGINSDADKISFHPYFSYKDLLGFVAMLLGLT
Cutthroat_trout       WGATVITNLLSAVPYVGGALVQWIWGGFSVDNATLTRFFAFHFLFPFVIAAATVLHLLFLHETGSNNPAGINSDADKISFHPYFSYKDLLGFVAMLLGLT
Zebrafish             WGATVITNLLSAVPYVGDTLVQWIWGGFSVDNATLTRFFAFHFLLPFIIIAMVILHLLFLHETGSNNPLGLNPNMDKIPFHPYFSNKDLLGFVIMLFSLS
Frog                  WGATVITNLLSAIPYIGNVLVQWIWGGFSVDNATLTRFFAFHFLLPFLIAGASILHLLFLHETGSTNPTGLNSDPDKIPFHPYFSYKDLLGFLIMLTSLT
Amphioxus             WGATVITNLFSAIPYLGPDLVQWLWGGFSVDNATLTRFFAFHFFLPFMIAGLSVVHLLFLHQTGANNPTGLAGDVDKVPFHAYFSYKDVVGFVVLLAGLV

                               3510      3520      3530      3540      3550      3560      3570      3580      3590      3600
                      ....|....|....|....|....|....|....|....|....|....|....|....|....|....|....|....|....|....|....|....|
Pufferfish            MLALFSPNYLGDPDNFTPANPLVTPAHIKPEWYFLFAYAILRSIPNKLGGVLALLASILVLMVVPLLHTSKQRSLTFRPFTQFLFWTLIADVAILTWIGG
Fugu                  TLALFSPNYLGDPDNFTPANPLVTPAHIKPEWYFLFAYAILRSIPNKLGGVLALLASILILMVVPFLHTSKQRSLTFRPLSQFLFWTLIADVVILTWIGG
Stickleback           SLALFAPNLLGDPDNFTPANPLVTPPHIKPEWYFLFAYAILRSIPNKLGGVLALLASILVLMVVPILHTSKQRGLTFRPITQFLFWTLIADVAILTWIGG
Large_yellow_croaker  TLALFSPNLLGDPDNFTPANPLVTPPHIKPEWYFLFAYAILRSIPNKLGGVLALLASILVLMVVPILHTSKQRGLTFRPITQILFWTLIADVFILTWIGG
Goldfish              LLALFSPNLLGDPENFTPANPLVTPPHIKPEWYFLFAYAILRSIPNKLGGVLALLFSILVLMVVPLLHTSKQRGLTFRPITQFLFWTLVADMIILTWIGG
Medaka                SLALFSPNLLGDPDNFTPANPLVTPPHIKPEWYFLFAYAILRSIPNKLGGVLALLASILVLFLVPILHTSKQRSLTFRPFTQFLFWLLVADVMVLTWIGG
Salmon                SLALFAPNLLGDPDNFTPANPLVTPPHIKPEWYFLFAYAILRSIPNKLGGVLALLFSILVLMVVPILHTSKQRGLTFRPLTQFLFWTLVADMLILTWIGG
Rainbow_trout         SLALFAPNLLGDPDNFTPANPLVTPPHIKPEWYFLFAYAILRSIPNKLGGVLALLFSILVLMVVPILHTSKQRGLTFRPLTQFLFWALVADMLILTWIGG
sea_trout             SLALFAPNLLGDPDNFTPANPLVTPPHIKPEWYFLFAYAILRSIPNKLGGVLALLFSILVLMVVPILHTSKQRGLTFRPLTQFLFWTLVADMLILTWIGG
Cutthroat_trout       SLALFAPNLLGDPDNFTPANPLVTPPHIKPEWYFLFAYAILRSIPNKLGGVLALLFSILVLMVVPILHTSKQRGLTFRPLTQFLFWALVADMLILTWIGG
Zebrafish             LLALFSPNLLGDPENFTPANPLVTPPHIKPEWYFLFAYAILRSIPNKLGGVLALLFSILVLMVVPILHTSKQRGMAFRPVTQFLFWTLVADMLVLTWIGG
Frog                  LLAMFAPNLLGDPENFTPANPLVTPPHIKPEWYFLFAYAILRSIPNKLGGVLALVFSILILALMPLLHTSKQRSLMFRPLTQITFWALVADTLILTWIGG
Amphioxus             FIALFSPNLLTDPENYIPANPLVTPVHIQPEWYFLFAYAILRSIPNKLGGVVALAMSIVVLFFMPFVHSSRQTSHNFRPLAQVLFWLMVVNVLLLTWLGG

                               3610      3620      3630
                      ....|....|....|....|....|....|....|....
Pufferfish            MPVEHPYVIIGQAASILYFSLFLVLMPAAGWLENKTLQ-
Fugu                  MPVEHPYIIIGQIASVLYFSLFLILMPMAGWLENKMLN-
```

```
Stickleback           MPVEHPFIIIGQVASVLYFSLFLVLYPGAAVMENKMLEW
Large_yellow_croaker  MPVEHPFIIIGQVASVLYFALFLIFLPVAGWLENKILE-
Goldfish              MPVEHPFIIIGQIASVLYFALFLVLFPLAGWLENKALKW
Medaka                MPVEHPFIIIGQIASFLYFSLFLIMAPAAGWLENKVLKW
Salmon                MPVEHPFIIIGQIASVIYFTIFLVLAPLAGWAENKALEW
Rainbow_trout         MPVEHPFIIIGQVASVIYFTIFLVLSPLAGWAEIKALQW
sea_trout             MPVEHPFIIIGQVASVIYFTIFLVLAPLAGWAENKALEW
Cutthroat_trout       MPVEHPFIIIGQVASVIYFAIFLVLSPLAGWAENKALQW
Zebrafish             MPVEHPYIIIGQMASILYFSLFLVLFPITGILENKALQW
Frog                  QPVEDPYIMIGQLASVIYFAIFLVLIPTIGWVENKLLNW
Amphioxus             QPVEYPYIFLGQAASVIYFVNILLLIPIVGYVENKLLFS
```
