# Supplementary material for: Family structure and phylogenetic analysis of odorant receptor genes in the large yellow croaker (Larimichthys crocea)
Source: BMC Evol Biol. 2011 Aug 11;11:237. doi: 10.1186/1471-2148-11-237 (PMC3162931; doi:10.1186/1471-2148-11-237)
Supplement: Additional file 6 — The result of a multiple sequence alignment of 230 OR genes from different species is included in this file. [file 1471-2148-11-237-S6.PDF]

```
                             10        20        30        40        50        60        70        80        90       100
                     ....|....|....|....|....|....|....|....|....|....|....|....|....|....|....|....|....|....|....|....|
LOR1                 -----------------------------------------MMDNVSKLTIFTLSG-LHEIANYRVTLFVLTLLCYCVIWLINLAIIVTIIMDK-SLHEP
LOR2                 -----------------------------------------MMDNVSKLTIFTLSG-LHEIANYRVTLFVLTLLCYCVIWLINLAIIVTIIMDK-SLHEP
LOR3                 -----------------------------------------MMDNVSKLTIFTLSG-LHEIANYRVTLFVLTLLCYCVIWLINLAIIVTIIMDK-CLHEP
LOR4                 -----------------------------------------MMDNVSKLTIFTLSG-LHEIANYRVTLFVLTLLCYCVIWLINLAIIVTIIMDK-SLHEP
LOR5                 -----------------------------------------MMDNVSKLTIFTLSG-LHEIANYRVTLFVLTLLCYCVIWLINLAIIVTIIMDK-SLHEP
LOR6                 ------------------------------------------MDNNSVITMFTLSG-LNGMANYRGTLFALTLLCYCVIWLVNLTIIVVIIMDK-SLHEP
LOR7                 ------------------------------------------MDNNSVITMFTLSG-LNGMANYRGTLFALTLLCYCVIWLVNLTIIVVIIMDK-SLHEP
LOR8                 ------------------------------------------MDNNSVITMFTLSG-LNGMANYRGTLFALTLLCYCVIWLVNLTIIVVIIMDK-SLHEP
LOR9                 ------------------------------------------MDNNSVITMFTLSG-LNGMANYRGTLFALTLLCYCVIWLVNLTIIVVIIMDK-SLHEP
LOR10                ------------------------------------------MDNNSVITMFTLSG-LNGMANYRGTLFALTLLCYCVIWLVNLTIIVVIIMDK-SLHEP
LOR11                ------------------------------------------MDDTLNVTYLTFGG-HVDVDRYRYVYFVIMFTIYILIICSNSVIVYLIWVHP-NLHEP
LOR12                ------------------------------------------MDDTLNVTYLTFGG-HVDVDRYRYVYFVIMFTIYILIICSNSVIVYLIWVHP-NLHEP
LOR13                ------------------------------------------MDDTLNVTYLTFGG-HVDVDRYRYVYFVIMFTIYILIICSNSVIVYLIWVHP-NLHEP
LOR14                ------------------------------------------MDDTLNVTYLTFGG-HVDVDRYRYVYFVIMFTIYILIICSNSVIVYLIWVHP-NLHEP
LOR15                ------------------------------------------MDDTLNVTYLTFGG-HVDVDRYRYVYFVIMFTIYILIICSNSVIVYLIWVHP-NLHEP
LOR16                ------------------------------------------MDDTLNVTYLTFGG-HVDVDRYRYVYFVIMFTIYILIICSNSVIVYLIWVHP-NLHEP
LOR17                ------------------------------------------MDDTLNVTYLTFGG-HVDVDRYRYVYFVIMFTIYILIICSNSVIVYLIWVHP-NLHEP
LOR18                ------------------------------------------MDDTLNVTYLTFGG-HVDVDRYRYVYFVIMFTIYILIICSNSVIVYLIWVHP-NLHEP
LOR19                ------------------------------------------MDDTLNVTYLTFGG-HVDVDRYRYVYFVIMFTIYILIICSNSVIVYLIWVHP-NLHEP
LOR20                ------------------------------------------MDDTLNVTYLTFGG-HVDVDRYRYVYFVIMFTIYILIICSNSVIVYLIWVHP-NLHEP
LOR21                ------------------------------------------MDDTLNVTYLTFGG-HVDVDRYRYVYFVIMFTIYILIICSNSVIVYLIWVHP-NLHEP
LOR22                ------------------------------------------MDDTLNVTYLTFGG-HVDVDRYRYVYFVIMFTIYILIICSNSVIVYLIWVHP-NLHEP
LOR23                ------------------------------------------MDDTLNVTYLTFGG-HVDVDRYRYVYFVIMFTIYILIICSNSVIVYLIWVHP-NLHEP
LOR24                ------------------------------------------MDDTLNVTYVTLGG-HVEVHKYRYLYFVIMFTIYILIICSNSVIVYLIWVHP-NLHEP
LOR25                ------------------------------------------MDDTLNVTYVTLGG-HVEVHKYRYLYFVIMFTIYILIICSNSVIVYLIWVHP-NLHEP
LOR26                ------------------------------------------MDDTLNVTYVTLGG-HVEVHKYRYLYFVIMFTIYILIICSNSVIVYLIWVHP-NLHEP
LOR27                ----------------------------------------------------------------------------------------------------
LOR28                ----------------------------------------------------------------------------------------------------
LOR29                ----------------------------------------------------------------------------------------------------
LOR31                ------------------------------------------MGNNTYPPYFNLTM-FVNIGTYRYPTFVLCLLLYALIVLANFLIIVVISQEK-TLHEP
LOR32                ------------------------------------------MGNNTYPPYFNLTM-FVNIGTYRYPTFVLCLLLYALIVLANFLIIVVISQEK-TLHEP
LOR33                ------------------------------------------MGNNTYPPYFNLTM-FVNIGTYRYPTFVLCLLLYALIVLANFLIIVVISQEK-TLHEP
LOR34                ------------------------------------------MGNNTYPPYFNLTM-FVNIGTYRCPTFVLCLLLYALIVLANFLIIVVISREK-TLHGP
LOR35                ------------------------------------------MGNNTYPPYFNLTM-FVNIGTCRYPTFVLCLLLYALIVLANFLIIVVISQEK-TLHEP
LOR37                ------------------------------------MLEA-----WNFSHSTFVFRGFPALQEHRRLLALPFTASYLSVLLGNSLLLYVICSVE-SLHSP
LOR38                ------------------------------------MLEA-----WNFSHSTFVFRGFPALQEHRRPLALPFTASYLSVLLGNSLLLYVICSVE-SLHSP
LOR39                ------------------------------------MLEA-----WNFSHSTFVFRGFPALQEHRRLLALPFTASYLSVLLGNSLLLYVICSVE-SLHSP
LOR40                ------------------------------------MLEA-----WNFSHSTFVFRGFPALQEHRRLLALPFTASYLSVLLGNSLLLYVICSVE-SLHSP
LOR41                ------------------------------------MLEA-----WNFSHSTFVFRGFPALQEHRRLLALPFTASYLSVLLGNSLLLYVICSVE-SLHSP
LOR42                ------------------------------------MLEA-----WNFSHSTFVFRGFPALQEHRRLLALPFTASYLSVLLGNSLLLYVICSVE-SLHSP
LOR43                -----------------------------------------MENKSFGFNSELTLDPFVIPPGGKYPIFFLGLIIYLFGIFCNLTLLSLIILQK-NLHKP
LOR44                -----------------------------------------MENKSFGFNSELTLDPFVIPPGGKYPIFFLGLIIYLFGIFCNLTLLSLIILQK-NLHKP
LOR45                -----------------------------------------MENKSFGFNSELTLDPFVIPPGGKYPIFFLGLIIYLFGIFCNLTLLSLIILQK-NLHKP
LOR46                -----------------------------------------MENKSFGFNSELTLDPFVIPPGGKYPIFFLGLIIYLFGIFCNLTLLSLIILQK-NLHKP
LOR47                -----------------------------------------MENKSFGFNSELTLDPFVIPPGGKYPIFFLGLIIYLFGIFCNLTLLSLIILQK-NLHKP
LOR48                ------------------------------------------MENYTYNSLTLQLEGLNVSKKWIYPVFLSILFSYLFIMVTNVGIAALVFIDK-NLHQP
LOR49                ------------------------------------------MENYTYNSLTLQLEGLNVSKKWIYPVFLSILFSYLFIMVTNVGIAALVFIDK-NLHQP
LOR50                ------------------------------------------MENYTYNSLTLQLEGLNVSKKWIYPVFLSILFSYLFIMVTNVGIAALVFIDK-NLHQP
LOR51                ------------------------------------------MENYTYNSLTLQLEGLNVSKKWIYPVFLSILFSYLFIMVTNVGIAALVFIDK-NLHQP
LOR52                ------------------------------------------MENYTYNSLTLQLEGLNVSKKWIYPVFLSILFSYLFIMVTNVGIAALVFIDK-NLHQP
LOR53                ------------------------------------------MENYTYNSLTLQLEGLNVSKKWIYPVFLSILFSYLFIMVTNVGIAALVFIDK-NLHQP
LOR54                ------------------------------------------MENYSYNSYTLQLQGLNVLKASIYPVYLFFFFSYLFIMVTNVGITVLIFIDK-NLHQP
LOR55                ------------------------------------------MENYSYNSYTLQLQGLNVLKASIYPVYLFFFFSYLFIMVTNVGITVLIFIDK-NLHQP
LOR56                ------------------------------------------MENYSYNSYTLQLQGLNVLKASIYPVYLFFFFSYLFIMVTNVGITVPIFIDK-NLHQP
LOR57                ------------------------------------------MENYSYHSYTLQLQELNVLKASIYPVYLFFFLSYLFIMVTNVGITVLIFIDK-NLHQP
LOR58                ------------------------------------------MENYSYNSYTLQLQGLNALKASIYPVYLFFFFSYLFIMVTNVGITVLIFIDK-NPHQP
LOR59                ------------------------------------------MENYSYNSYTLQLQGLNVLKASIYPVYLFFFFSYLFIMITNVGITVLIFIDK-NLHQP
LOR60                ------------------------------------------MENYSYNSYTLQLQGLNVLKASIYPVYLFFFFSYLFIMITNVGITVLIFIDK-NLHQP
LOR61                ------------------------------------------MENYSYNSYTLQLQGLNVLKASIYPVYLFFFFSYLFIMVTNVGITVLIFIDK-NLHQP
LOR62                ------------------------------------------MENYSYNSYTLQLQGLNVLKASIYPVYLFFFFSYLFIMVTNVGITVLIFIDK-NLHQP
LOR63                ------------------------------------------MENYSYNSYTLQLQGLNVLKASIYPVYLFFFFSYLFIMVTNVGITVLIFIDK-NLHQP
LOR64                ------------------------------------------MENYSYNSYTLQLQGLNVLKASIYPVYLFFLFSYLFIMVTNVGITVLIFIDK-NLHQP
LOR65                ------------------------------------------MENYSYNSYTLQLQGLNVLKASIYPVYLFFFFSYLFIMVTNVGITVLIFIDK-NLHQP
LOR66                ---------------------------------------------RRFNSLTLQLEGLKITKTNKYPIFIFLLLSYMFILIANVGIVILIWKER-SLHQP
LOR67                ---------------------------------------------RRFNSLTLQLEGLKITKTNKYPIFIFLLLSYMFILIANVGIVILIWKER-SLHQP
LOR68                ---------------------------------------------RRFNSLTLQLEGLKITKTNKYPIFIFLLLSYMFILIANVGIVILIWKER-SLHQP
LOR69                ---------------------------------------------RRFNSLTLQLEGLKITKTNKYPIFIFLLLSYMFILIANVGIVILIWKER-SLHQP
LOR70                ---------------------------------------------RRFNSLTLQLEGLKITKTNKYPIFIFLLLSYMFILIANVGIVILIWKER-SLHQP
LOR71                ---------------------------------------------RRFNSLTLQLEGLKITKTNKYPIFIFLLLSYMFILIANVGIVILIWKER-SLHQP
LOR72                -----MSHCFSNRNQRKLCSDTSTGDKCIFRVKFSSKMSYGNQTQTLNITAEQQ----YVGLLERVVFSTLIGMPCCLFLFINGIMLFTLRSKAAFRETC
LOR73                -----MSHCFSNRNQRKLCSDTSTGDKCIFRVKFSSKMSYGNQTQTLNITAEQQ----YVGLLKRVVFSMLIGMPCCLFLFINGIMLFTLRSKTVFRETC
LOR74                -----MSHCFSNRNQRKLCSDTSTGDKCIFRVKFSSKMSYGNQTQTLNITAEQQ----YVGLLERVVFSTLIGMPCCLFLFINGIMLFTLRSKAAFRETC
LOR75                -----MSHCFSNRNQRKLCSDTSTGDKCIFRVKFSSKMSYANQSQTLNITTKQQ----YLGLLERVVFSMLIEMPCCLFLFINGIMLFTLRSKAVFRETS
LOR76                -----MSHCFSNRNQRKLCSDTSTGDKCIFRVKFSSKMSYGNQTQTLNITAEQQ----YVGLLERVVFSTLIGMPCCLFLFINGIMLFTLRSKAAFRETC
LOR77                -----MSHCFSNRNQRKLCSDTSTGDKCIFRVKFSSKMSYGNQTQTLNITAEQQ----YVGLLERVVFSTLIGMPCCLFLFINGIMLFTLRSKAAFRETC
LOR78                -------------------------------------MLNANLSQTLNVSTEQT----YAGLLGRILFTTLTGMPCCLFLLINGIMLFTLRSKAVFRETS
LOR79                -------------------------------------MLNANLSQTLNVSTEQT----YAGLLGRILFTTLTGMPCCLFLLINGIMLFTLRSKAVFRETS
LOR80                -------------------------------------MLNANLSQTLNVSTEQT----YAGLLGRILFTTLTGMPCCLFLLINGIMLFTLRSKAVFRETS
LOR81                -------------------------------------MLNANLSQTLNVSTEQT----YAGLLGRILFTTLTGMPCCLFLLINGIMLFTLRSKAVFRETS
LOR82                -------------------------------------MLNANLSQTLNVSTEQT----YAGLLGRILFTTLTGMPCCLFLLINGIMLFTLRSKAVFRETS
LOR83                -----------------------------------MDVSSVNTTGIVGYADSFSK---------AVTKNVIVVFIGISITYINASLIHTFSKHQIFYTNP
LOR84                -----------------------------------MDVSSVNTTGIVGYADSFSK---------AVTKNVIVVFIGISITYINASLIHTFSKHQIFYTNP
LOR85                -----------------------------------MDVSSVNTTGIVGYADSFSK---------AVTKNVIVVLIGISITYINASLIHTFSKHQIFYTNP
LOR86                -----------------------------------MDVSSVNTTGIVGYADSFSK---------AVTKNVIVVLIGISITYINASLIHTFSKHQIFYTNP
LOR87                -----------------------------------MDVSSVNTTGIVGYADSFSK---------AVTKNVIVVFIGISITYINASLIHTFSKHQIFYTNP
LOR88                -----------------------------------MDVSSVNTTGIVGYADSFSK---------AVTKNVIVVFIGISITYINASLIHTFSKHQIFYTNP
LOR89                -----------------------------------MDVSSVNTTGIVGYADSFSK---------AVTKNVIVVFIGISITYINASLIHTFSKHQIFYTNP
LOR90                -----------------------------------MDVSSVNTTGIVGYADSFSK---------AVTKNVIVVFIGISITYINASLIHTFSKHQIFYTNP
LOR91                -----------------------------------MDVSSVNTTGIVGYADSFSK---------AVTKNVIVVFIGISITYINASLIHTFSKHQIFYTNP
LOR92                -----------------------------------MDVSSVNTTGIVGYADSFSK---------AVTKNVIVVFIGISITYINASLIHTFSKHQIFYTNP
LOR93                ----------------------------------------------------------------MNGLVIIVQILVVIFLCVNMLLIVTFFSKEVFYTTM
LOR94                ----------------------------------------------------------------MNDLVIIVQILVVIFLCVNMLLIVTFFSKEVFYTTM
LOR95                ----------------------------------------------------------------MNDLVIIVQILVVIFLCVNMLLIVTFFSKEVFYTTM
LOR96                ----------------------------------------------------------------MNDLVIIVQILVVIFLCVNMLLIVTFFSKEVFYTTM
LOR97                ----------------------------------------------------------------MNDLVIIVQILVVIFLCVNMLLIVTFFSKEVFYTTM
LOR98                ----------------------------------------------------------------MNDLVIIVQILVVIFLCVNMLLIVTFFSKEVFYTTM
LOR99                ----------------------------------------------------------------------------------------------------
LOR100               ----------------------------------------------------------------------------------------------------
LOR101               ----------------------------------------------------------------------------------------------------
LOR102               ----------------------------------------------------------------------------------------------------
LOR103               ----------------------------------------------------------------------------------------------------
LOR104               ----------------------------------------------------------------------------------------------------
LOR105               ----------------------------------------------------------------------------------------------------
LOR106               ----------------------------------------------------------------------------------------------------
LOR107               ----------------------------------------------------------------------------------------------------
LOR108               ----------------------------------------------------------------------------------------------------
LOR109               ----------------------------------------------------------------------------------------------------
LOR110               ----------------------------------------------------------------------------------------------------
LOR111               ----------------------------------------------------------------------------------------------------
Fugu_OR123-1         -----------------------------------------MSDNTSVVNMFTLSG-LGGITNYKITLFVFTFLYYCVILQVNLTVILTIIVDK-SLHEP
Fugu_OR6765-1        ------------------------------------------MDNASDVTMFTLSG-FNGIVNYKFTLFALTFVCYCVIVQVNVTLILTIIMDK-GLHEP
Fugu_OR4133-1        ----------------------------------MVTTLSHLVMDNASDVTMFTLSG-FNGIINYRFTLFALTFVCYCVIVQVNVTLILTIIMDK-SLHEP
Fugu_OR3630-2        ------------------------------------------MDNRANVTYITVEG-YVELQKYRYLYFVIMLTAYILIICSNSTIVFLICFHR-NLHEP
Fugu_OR8617-1        ------------------------------------------MDNRANVTYITIEG-YVELQKYRYLYFVIMLTAYILIICSNSTIVFLICFHR-NLHEP
Fugu_OR5510-1        ------------------------------------------MDNRANVTYIIVEG-YVELQKYRYLYFVIMLTAYILIICSNSTIVFLICFHR-NLHEP
Fugu_OR117-1         ------------------------------------------MDNFTTPPYFNLTM-FLNIGQYRYLAFVLCFILYASIVFANVIIIVVISREN-ALHEP
Fugu_OR2346-5        ------------------------------------------MENNSASFHFNLTM-FVNIGHYRYLAFVFCLLLYSFMISANFVIILVVSRER-TLHEP
Fugu_OR5287-2        ------------------------------------MLDGS--LGQNFSHSTFSFRGFPELHCYRRLLALPFSVSYLSVLLGNCLLVYVICSVE-HLHSP
Fugu_OR142-1         -----------------------------------------MENRSFGLSSELTLDTFLIPPQGKYPIFFLGVTIYCFGVFCNMTLLTLIILQR-NLHKP
Fugu_OR1026-2        ------------------------------------------MENYTFNSFTLQLEGLKVTESSVYPVFFFFLVSYIFIMLSNIGIVALIFIDS-SLHQP
Fugu_OR8298-1        --------------------------------MNFCFSGFRLMDNYTFNSFTLQLEGLKVTEVSVYPVFFFFLVSYIFIIVANIAIVALIFIDS-SLHQP
Fugu_OR6030-1        ---------------------------------------MNEIWSLVNVTVEQQ----YQGLLERMMFSSLTTVPCCVFLYINGIMLFSLRSKTVFCETS
Fugu_OR59-1          ---------------------------------------MDEIQPLVNFTVEQQ----YQGLLERMMFSSLTTVPCCAFLYINGIMLFSLRSKTVFCETS
Fugu_OR4208-3        -------------------------------------TPRLKFTSVDPGEAGSHR---------LITR-----------CGLTCCVCPRCPVPQVFYTNP
Fugu_OR4208-2        ----------------------------------------------------------------------------------------------MFYMNP
Fugu_OR6818-1        ---------------------------------------MAANSSEPAGQSSAR---------KINSRVILVQVLVLVFLCINFLLIVTFSMRHFFYTTM
Fugu_OR7903-1        ---------------------------------------MSDNASVAGGVSLRV---------QIFERIIITQLIVSIFVSDNLFLIATVFSNQVFLSTM
Pufferfish_OR8981-2  ------------------------------------------MNNTSVVTMFTLSG-FDSITEHRFILFALTFVFYCVIVQVNVTLIVIIIIDK-SLHEP
Pufferfish_SCAF8981  ------------------------------------------MNNTSVVTMFTLSG-FDSITEHRFTLFALTFVCYCVIVQVNVTLILIIIIDK-SLHEP
```

```
Pufferfish_OR15134-1      ------------------------------------------MEPRANVSYITVDG-YVEVHRYRYLYFVLMLTAYLLILCTNSTVVYLICLHR-DLHQP
Pufferfish_OR14339-2      ------------------------------------------MDNSTNPPYFNLTM-FFNIGQSRYLAFVLFFILYLFIVFANIIIIVVTAREN-TLHEP
Pufferfish_OR14328-1      ------------------------------------------MDNSTNPPYFNLTM-FFNIGQSRYLAFVLFFILYLFIVFANVIIIVVTAREN-ALHEP
Pufferfish_OR14677-8      ------------------------------------MLDGS--PGHNLSHSTFIFRGFPELHRYRRLLVLPFSATYLLVLLGNSLLVYVICNME-HLHSP
Pufferfish_OR14536-2      ---------------------------------------MDKMRSLSNVTAEQQ----YQGLLERLMLSFLTTVPCCIFFYINTIMLFTLRSKTVFCETS
Pufferfish_OR14536-1      ---------------------------------------MDKVLSSSNVTDELQ----NRGTVERLMFSFLTTVPCCIFFYINAIMLFTLRSKTVFCETS
Pufferfish_OR10960-2      -----------------------------------MSAPDANVSVVVEYRDSLSK---------AVTKNVIVVVLSISINYVNVGLVQTFHRHQVFYSNP
Pufferfish_OR10960-3      -----------------------------------MNASAANASGTAAFRDSFSK---------AVTKNAVVLVLGLFINYINRGLIHTFCKHQVFYTNS
Pufferfish_OR12434-1      -----------------------------------------------------Q---------ILFERVLITQILAFMFISTNLVLLATVFSKPVFYSTM
Medaka_ORUn.33            ------------------------------------MWEAA--MDGNSSHSSFVFRCFPELHAHRRLLALPFFLSLLSVLLGNSALILVIRREE-RLRGP
Medaka_ORUn.2             -------------------------------------MEN------GSFHTYFVLNGFDELGVLRPLLFLPFSFAFLVSLLANGLLVYVVFSQR-SLHQP
Medaka_OR21.18            ------------------------------------------MNNSTGVVLFSLSG-FSGSATHRLTLFCLSLLCYCLIWMVNVFLILVIIFDQ-SLREP
Medaka_OR21.19            ------------------------------------------MNN---LSVIFLSG-LNETRVHRPTLFVLSLLCYCLILLVNVSLVIIIILDK-SLHEP
Medaka_OR21.20            ------------------------------------------MNN---LSVIFLSG-LNETRVHRPTLFVLSLLCYCLILLVNVSLVIIIILDK-SLHEP
Medaka_OR13.14            ---------------------------------------MANNSSVISGNFPVK---------LISKDIIIVQVLIAIFLSINFFLISTFFRKNVFHTSM
Medaka_OR13.12            ---------------------------------------MSLNSSASGGGFPVK---------IINDRIIIVQVSIAVFLSINFFLISTFFRRDVFYNTM
Medaka_ORUn.23            ----------------------------------------MSSSSAMNVTSSLL----DEGLMRVIILSTLSTVPACVFLVINGIMLFTLRSKPVFRETC
Medaka_ORUn.21            ----------------------------------------MANISQGEVTSESL----YQNFVRTLIFSIISTVTSCVFLVINGIMLFTLRSKPVFRETC
Medaka_OR14.15            ----------------------------------------MANISQGEVTSESL----YQNFVRTLIFSIISTVTSCVFLVINGIMLFTLRSKPVFRETC
Medaka_OR14.13            -----------------------------MFHEPKISLSYRSMENYTLNSYYLQLESLNVSEASKYPVFFLFLFIYLLIMLTNVGILVLIIIDR-NLHQP
Medaka_OR14.4             ------------------------------------------MENITYNSIVLQVEGFKVTKESTEYAFFLFFFSYIVIMILNLGILAIIAIDK-SLHQP
Medaka_OR14.6             ------------------------------------------MENITYNSIVLQVEGFKVTKESTEYAFFLFFFSYIVIMILNLGILAIIAIDK-SLHQP
Medaka_OR13.4             ----------------------------------------------MNASSVIHFSGFNLTAESVVPAFLFATLSYMVILFCNLTLILTIVLNK-SLHQP
Medaka_OR13.5             ------------------------------------------MFSNFSSETLLTLSSLGFSSIYIYPAFVFGTLFYLIIVFCNLLVLTTIAVSK-KLHKP
Medaka_OR13.6             ------------------------------------------MLSNSSSVDILTLSSLDLQPTYIYPSFVFGTLLYLITVFCNLLVLLTIAVSK-KLHKP
Medaka_OR13.7             ---------------------------------------MKMENSSFGFYSKLTMDTFFIPAGGKYPIILLGIAIYLFGMSCNLTLLTLIILKK-NLHKP
Medaka_ORUn.1             ------------------------------------------MENNSQI-VFVLHG-LNQSLLNRQVSFGLALTAYLFTLFVNLTLIGTVLLEKTHLHQP
Medaka_OR21.2             ----------------------------------------------MNVTYITLNG-FVEIEKYRYFYFFMMLPVYIFIVCSNCTIISLIVIHK-NLHEP
Medaka_OR21.3             ----------------------------------------------MNVTYISLDG-LVEIEKYRYFYFFMMLTFYILIVCSNCTIISLIVIHK-NLHEP
Stickleback_OR1.1         -------------------------------------MEAS--PGRNFSHASFVFSGFDGLQKHRQLLALPLCASYLVVLLGNGLLVLVVLRTE-SLKGP
Stickleback_OR16.3        -----------------------------------------MMHNSSHVKVFFLSG-LNGTMTNRVALFSVMLLCYFFIVLVNVVLVVTIILDK-NMHEP
Stickleback_OR1.12        ---------------------------------------MAANNSVVGVDSPMN---------NFNYRVVIVQVLVSVFLFINFWLIRTFFVRD-FYTVM
Stickleback_OR1.13        ---------------------------------------MAANNSVVGVESPTY---------KINYRLVVVQVLVSVFLFINFLLIRTFFVRD-FYTVM
Stickleback_ORUn.9        ----------------------------------------MMS-PLTNTTGGLQ----YQGLLESVAFSMLTTVPCCVFLLVNAAMLLTLRSRPLFRETP
Stickleback_OR7.1         ---------------------------------------MLHDAVAMNNTTG-------VMRFQSPVRVLLSMLPCILFLYINGVMLFALLSKPLLLESS
Stickleback_ORUn.6        ------------------------------------------MENFTFNSPTLQLEALNISKAFFYPVFIFLLFSYLFIIIANVGIAAVILLDK-SLHQP
Stickleback_ORUn.8        ------------------------------------------MENDTFNSLTLRLEGLNVWKSYFYPAFIFLLLSYIFIMLMNVGIAAVILLDK-SLHQP
Stickleback_OR1.18        -----------------------------------------MENKSFGFRSELTLDTFVIPPGGKYPMFLFGIAIYLFSVFCNLMLLSLIILKR-NLHKP
Stickleback_OR16.10       ------------------------------------------MENNTFPQYFNLTM-FVNIGNYRYLAFVLCLLLYAFIIFSNLVIIVLISREK-TLHEP
Stickleback_OR16.32       ------------------------------------------MDNVSVITMFTLSG-LSETTHYRGFLFALTLLCYSVIWLVNLTIIVTVIADK-KLHEP
Stickleback_OR16.1        ------------------------------------------MENNTFPQYFNLTM-FVKLGNYRYLAFVLCLLLYAFIIFSNLVIIVLISREI-TLHEP
Stickleback_OR16.5        ------------------------------------------MENNTFPQYFNLTM-FVKLGNYRYLAFVLCLLLYAFIIFSNLVIIVLISREI-TLHEP
Stickleback_ORUn.1        ------------------------------------------MDAHLNVTYVTLDG-HLDLNTYKYIYFVMMLTMYILIICTNSTIVYLIVTHN-NLHQP
Stickleback_ORUn.17       ------------------------------------------MDYRLNVTYITFGG-YVEVHIYRYLYFVVMFTVYILIISSNVTIVCIIMIHK-NLHEP
Zebrafish_OR126-7         ------------------------------------------LENESSVSVFTLSG-LDETMENRYVLFSLTVLFYPLMVFCNVTVIVAIISHK-KLHEP
Zebrafish_or126-3         --------------------------------------MQSPLKNESNVFVFALSG-LNETMENRYVLLSFTALFYPVVIFCNVTVILTIFLHK-KLHEP
Zebrafish_OR123-1         ------------------------------------------MDNNSSFSMFSLTA-LNGSRSSRITIFSFALPGYFLTIFVNTLLILIIVLEK-VLHQP
Zebrafish_OR125-1         ------------------------------------------MDNMSYPVILTLMV-PKETKLYRHIYFICFLVLYLLILSINICLVMVIIIEK-VLHEP
Zebrafish_OR127-1         ------------------------------------------MENGTMSSSFYFTL-FKEFVHIRYVILTLTLTVYLAIIVCNAIILFVVFKER-SLHEP
Zebrafish_OR128-10        ------------------------------------------MENGTT---FYLML-FENIGCTRYALFSLGIILYSAILLFNFLVILAIFLER-TLHQP
Zebrafish_OR116-2         -----------------------------------------MENLSIIVSFELTLDPFSIPPGAKYPIFIFGIFIYMFGAFCNLTLLLLIFLTE-SLHKP
Zebrafish_zor6a           ------------------------------------MTEEL--QGANFSHTKFLLTGFPEIYKYRRFLFLPFFLTYLLLLVGNSLLLFVIKSTE-SLHSP
Zebrafish_OR117-1         -------------------------------------MAGNLSNPTFTFY--LKIAKFDIDPKAKYPVFFAGILVYMFSVICNTTILGLIISQK-SLHKP
Zebrafish_OR115-15        ------------------------------------------MENYTFNSIILQLEGLRVSEEATYPVFVFFLLFYVAIMVLNIGIFIVITTHR-SLHEP
Zebrafish_OR133-7         --------------------------------------MSNLNASASNLTQR-------LMDQDAPTKAFLFVTPCVIFLYVNGVMLFTLRKKAVFQETS
Zebrafish_OR132-1         --------------------------------------MAGSNGTYGNVPFTHQQVFKLDMDAGTSTKTAVAVLTSLFFFSVNCVMISVLKSKRMFYETP
Zebrafish_OR134-1         -----------------------------------MNSTAGQQLLLR---DTFAM---------AFMKNFIVVLVWFLLSYINGSVVATFFRHQIFYEDP
Zebrafish_OR131-2         --------------------------------------MNSTSNSSLGNTFISK---------TLKEKSLTVQVLVGILLYVNGLMIFTFLKKETFRDT-
Zebrafish_OR131-1         --------------------------------------MNSTSNSSLSNTFIMK---------SLKEKALLVQVLVGILLYVNGLMIFTFLKKETFRDT-
Goldfish_OR3              --------------------------------------MQPPLENQSIVLVFTLSG-LNETMGKRFMFFSLTALYYPFIVLCNVIIIYTVISHK-KLHEP
Goldfish_OR2              ----------------------------------------MSSGNNSFVKDFFIVGFPGLQPGYYGAVAALLLFAYVCILVGNGIFIALFTLEK-RLHKP
Rainbow trout OR500-1     ------------------------------------------MENSSQVKFFYLFG-LQETFNNKSVYFILPLITYLLIITVNLPLIITIIQEK-GLHEP
Cutthroat trout OR600-1   ------------------------------------------MENHTYPNHILLLEGLKVTQQSAYPAFILFLIIYIFTMVANIGLISLISMER-SLHQP
Brown trout OR500-1       ------------------------------------------MENSTQVKFFYLFG-LQETFNNKSVYFILSLITYLLIITVNLTLIKTIIQEK-GLHEP
Atlantic salmon SORB      ------------------------------------------MVNLTYNSYMLHLEGLKVTEETMYPVFIILFFSYVIIMFTNVGIVVLISMEK-SLHQP
Medaka_mfOR2              ------------------------------------------MELEFNVSLITLDG-FIEIEKYRYLYFLIVFTVFILILLCNCTIIFIIVVQK-SLHEP
Medaka_mfOR1              ------------------------------------------MELEFNVSLITLDG-FIEIEKYRCLYFLIVFTVFILILFCNCTIIFIIVVQK-SLHEP
Medaka_OR_Y3              ------------------------------------------MENITFNSFYLQLEGLKVTETSKYYIFLIFFFSYIFIMVTNVGILVLIIIDR-NLHQP
Medaka_ORY1               ------------------------------------------MENITYNSIVLQVEGFKVTKESTEYAFFLFFFSYIVIMILNLGILAIIAIDK-SLHQP
Frog_OR-52D1              ------------------------------------------MENSSYSQPSMLTLGFGQLREIKYFYSTLVLLCFMIIVVSNSAVISAIAMHR-SLQEP
Frog_OR-52E8              ------------------------------------------MANATYSQPTVLALSFGELTAVKYLYGAIVFLIFLMIVASSSAVIGTIILHR-SLHEP
Frog_OR-52A1              ------------------------------------MEPGISNQSFIFSYTDFTLLGFPGISRWRPLLAIPFFSVYLVILSGNSLLICLICIKK-TLHSP
Frog_OR-52P1              ------------------------------------MEPGISNQSFIFSYTDFTLLGFPGISRWRPLLAIPFFSIYLVILSGNSLLICLICIKK-TLHSP
Frog_OR-52A5              MEVGATKDYYELLAVSVEHDSLEPKSNPTQQDGALRPNAMWNVTSAPGNTTQVSPTNSQLGVIIRTTFFVSVLVCLFFFAYLVTIILTVFFTKPSLRENS
Frog_OR-2M5               ---------------------------------------MSNRTSET----------------VRMTFLILSFLCFGFFIYLVAIILYIFLSNAQVRESA
Frog_OR-2M2               ------------------------------------------MSNQSTISEFILMGFPGLQQKYFIPVSITMFLVYCVSLVANSSVIILVLQKE-QLHQP
zebrafish_OR10.1          ----------------------------------MQNNIASSNSTLIQPAGFYIVGLRSMP--YSNVYVMFLTVLFVITVICNVFLISIIVYDQ-RLHVP
zebrafish_OR10.14         --------------------------------MNDF--AENRNIS--IPSYFYISGFSGIP--HMRYYYIFLSFVYIISFLGNSCLMFVIIIDR-NLHTA
zebrafish_OR15.39         ----------------------------------MNLSVTFSNGTASFNEGFYLTAFQTLP--TKNYLILGLAIIYIITLLGNLVLLSVVLMNS-SLQNP
Medaka_ORUn.6             ---------------------------------MSFLRTALNSSVIIHPPGFYIVGFESLP--SVNVFFIFLAIVYVVTVLFNGLLIIVVVINP-SLHTP
Medaka_ORUn.28            --------------------------------MEFFNSALGKNITFVRPAYFKIAGFIGIP--NMNYYYIFLFFVFFISVLGNTAVMTVIYLDH-NLRTA
Medaka_ORUn.27            --------------------------MLASLMMDLFNSALGRNITFVRPPYFIITGFIGIP--NVKYYYIFLFFVYIVSVVGNTAVMAVIYMDN-SLRTP
Stickleback_OR7.3         ---------------------------------MSSIRTVLNDSAIIHPPGFYIIAFQTLP--YISVYIIFLAFVYAVTLVFNILLISLIARDH-CLHTP
Stickleback_OR7.4         ---------------------------------MTFLRTSLNNSLIIRPPGFYIVGFETLP--FISVYFIFLSFVYVVTVMFNSLVIYVIAFNH-CLHSP
Stickleback_OR7.8         --------------------------------MNLFNSALGENITFVRPAYFVISGLSGIQ--EMKHYYVFLCFVYIVSVLGNTVVMAVIYLDH-NLRTP
Fugu_ORUn.83              --------------------------------MELFNAAPGKNVSFVHPPYFIISGLKGIP--HIKFYYVFLFFVYIVSVLGNSVVMAVICLDH-NLRTP
Fugu_ORUn.20              ---------------------------------MSLLRTVSNGSIIIHPPGFYVVGFETFP--HIRFYFIFLAFVYVVTVFFNCLLIYVIVSNR-CLHTP
Pufferfish_OR7.1          --------------------------------MGLFNAAPGKNISFVHPPYFIISGLMGIP--HIRFYYIFLFFVYIVSVLGNSSVMAVICLDR-SLRTP
Pufferfish_OR7.3          ---------------------------------MLFLQTVSNDTVIIHPPGFYVIGFETFP--HIRFYFIFLAFVYVVTLLFNCLLIYVIASHP-CLHTP
zebrafish_OR10.29         -------------------------------------MLSKN---WTTISEFIIVGIPGLHPDYYGLVSAIFFVIYITTVAGNMFFLVLFVTTE-SLRKP
zebrafish_OR10.31         -------------------------------------MSQANDTTASTVTEFFIMGFPGLLPKYYSLTAALLFCIYIAVITGNSLIVVLFVIER-SLHKP
zebrafish_OR15.58         ---------------------------------MGSRSNVETYQNTTIITEFVLRGFPGLPPEYHGSVGALLFILYLVLATGNIFIIVFVMIEK-SIQKP
zebrafish_OR15.57         --------------------------------------MTAYYTN---ITEFYLLGFPGLHPEYYGAVGFLLFFVYLTLAGGNIFIIAFIAYEK-SLQKP
Medaka_OR13.1             ----------------------------------------MISSNVTKVKDFIIVGIPGLLPEYHGLASFVFLAVYLTILFGNSFILAVIIFER-VLHKP
Medaka_OR13.3             -------------------------------------MALLIIPNISRTESFHILGFPGLSPQYYAPVSALFFFIYLTTATGSVFILALVICEK-SLQKP
Stickleback_OR1.25        ----------------------------------------MMNSNLTVMKSFFILGFPGLSPTYYRPVSALLFFFYLAIAIGNIFIFTFVVCEK-SLQKP
Fugu_ORUn.8               -----------------------------------------MIDNVTGTESFFILGFPGLAPEYYRPVSALFLIIYLVIAFGNIFILTFVMCQK-SLQKP
Pufferfish_OR16.7         -----------------------------------------MTNNVTGTNSFVILGFPGLAPRYYGPVSALFLIIYLAIVLGNVFILTFVMCHK-SLQKP
Frog_OR669.1              -------------------------------------MIHQSMANRTRVSEFILLGFPGLDESYDVPVSIGLFLAYIISLFANATVILLIAFSR-HLHQP
Amphioxus_105.1           -----------------------------------------MGDQQDGSQDSDDADIAVSLGPASQGLQTAYLVISLIVIVAANLLIILLVCRKEYLRQP
Amphioxus_39.1            -------MRNLGRSRRRRIFIRRGTVVSDSIRRRLVIVKSGAMDQSVFSLNLDNIHDVISNSPLSRGLQTLYLVTSLVVAVGCSSLVIFLVWKTESLQKP

                                   110       120       130       140       150       160       170       180       190       200
                          ....|....|....|....|....|....|....|....|....|....|....|....|....|....|....|....|....|....|....|....|
LOR1                      MYIFLCNLCINGLYETAGFYPKFLIDLLS--TFHVISYAGCLLQGFVLHSSACADFSILVLMAYDRYVAICRPLVYHSVMTTQRVCVFIFFAWLIPLYLV
LOR2                      MYIFLCNLCINGLYGTAGFYPKFLIDLLS--TFHVISYAGCLLQGFVLHSSACADFSILVLMAYDRYVAICRPLVYHSVMTTQRVCVFIFFAWLIPLYLV
LOR3                      MYIFLRNLCINGLYGTAGFYPKFLIDLLS--TFHVISYAGCLLQGFVLHSSACADFSILVLMAYDRYVAICRPLVYHSVMTTQRVCASIFFAWLIPLYLV
LOR4                      MYIFLCNLRINGLYGTAGFYPKFLIDLLS--TFHVISYAGCLLQGFVLHSSACADFSILVLMAYDRYVAICRPLVYHSVMTTQRVCASIFFAWLIPLYLV
LOR5                      MYIFLCNLCINGLCGTAGFYPKFLIDLLS--TFHVISYAGCLLQGFVLHSSACADFSILVLMAYDRYVAICRPLVYHSVMTTQRVCASIFFAWLTPLYLV
LOR6                      MYIFLCNLCINGLYGTTGFYPKFLIDLLS--TTHVISYAGCFLQGFVLHSSACADFSLLALMAFDRYVAICRPLVYHSVMTKQRVCLFVFFAWLIPFYLM
LOR7                      MYIFLCNLCINGLYGTTGFYPKFLIDLLS--TTHVISYAGCFLQGFVLHSSACADFSLLALMAFDRYVAICRPLVYHSVMTKQRVCLFVFFAWLIPFYLM
LOR8                      MYIFLCNLCINGLYGTTGFYPKFLIDLLS--TTHVISYAGCFLQGFVLHSSACADFSLLALMAFDRYVAICRPLVYHSVMTKQRVCLFVFFAWLIPFYLM
LOR9                      MYIFLCNLCINGLYGTTGFYPKFLIDLLS--TTHVISYAGCFLQGFVLHSSACADFSLLALMAFDRYVAICRPLVYHSVMTKQRVCLFVFFAWLIPFYLM
LOR10                     MYIFLCNLCINGLYGTTGFYPKFLIDLLS--TTHVISYAGCFLQGFVLHSSACADFSLLALMAFDRYVAICRPLAYHSVMTKQRVCLFVFFAWLIPFYLI
LOR11                     MYIFIAALLINSVLFSTTIYPKLLIDFLS--DKQIISHQACLVQFHMFYSLAGAEFLLLAAMAYDRYVSICKPLQYPTIMSKTTVSIFLVSAWLLPACQL
LOR12                     MYIFIAALLINSVIFSTTIYPKLLIDFLS--DKQIISHQACLVQFHMFYSLAGAEFLLLAAMAYDRYVSICKPLQYPTIMSKTTVSIFLVSAWLLPACQL
LOR13                     MYIFIAALLINSVLFSTTIYPKLLIDFLS--DKQSISHQACLVQFHMFYSLAGAEFLLLAAMAYDRYVSICKPLQYPTIMSKTTVSIFLVSAWLLPACQL
LOR14                     MYIFIAALLINSVLFSTTIYPKLLIDFLS--DKQIISHQACLVQFHMFYSLVGAEFLLLAAMAYDRYVSICKPLQYPTIMSKTTVSIFLVSAWLLPACQL
LOR15                     MYIFIAALLINSVLFSTTIYPKLLIDFLS--DKQIISHQACLVQFHMFYSLAGAEFLLLAAMAYDRYVSICKPLQYPTIMSKTTVSIFLVSAWLLPACQL
LOR16                     MYIFIAALLINSVLFSTTIYPKLLIDFLS--DKQIITYQACLVQFHMFYSLGGSEFLLLAAMAYDRYVSICKPLQYPTIMSKTTVSIFLVSAWLLPACQL
LOR17                     MYIFIAALLINSVLFSTTIYPKLLIDFLS--DKQIITYQACLVQFHMFYSLGGSEFLLLAAMAYDRYVSICKPLQYPTIMSKTTVKIFLVSAWLLPACQL
LOR18                     MYIFIAALLINSVLFSTTIYPKLLIDFLS--DKQIITYSACLLQSFFYYSLGCSEFLLLAAMSYDRYVSICKPLQYSTIMSKTTVKIFLVSAWLLPACQL
LOR19                     MYIFIAALLVNSVPFSTTIYPKLLIDFLS--DKQIITYQACLVQFHMFYSLGGSEFLLLAAMAHDRYVSICKPLQYPTIMGKTTVSIFLVSAWLLPACQL
LOR20                     MYIFIAALLINSVLFSTTIYPKLLIDFLS--DKQIITYQACLVQFHMFYSLGGSEFLLLAAMAYDRYVSICKPLQYPTIMGKTTVSIFLVSAWLLPACQL
LOR21                     MYIFIAALLINSVLFSTTIYPKLLIDFLS--DKQIITYQACLVQFHMFYSLGGSEFLLLAAMAYDRYVSICKPLQYPTIMSKTTVSIFLVSAWLLPACQL
LOR22                     MYIFIAALLINSVLFSTTIYPKLLIDFLS--DKQIITYLACLVQFHMFYSLGGSEFLLLAAMAYDRYVSICKPLQYPTIMSKTTVSIFLVSAWLLPACQL
LOR23                     MYIFIAALLINSVLFSTTIYPKLLIDFLS--DKQIISHQACLVQFHMFYSLAGAEFLLLAAMAYDRYVSICKPLQYPTIMSKTTVSIFLVSAWLLPACQL
LOR24                     MYMFIAALLINSVLFSTTIYPKLLIDFLS--DKQIITYQACLVQFHMFYSLGGSEFLLLAAMAYDRYVSICKPLQYPTIMSKTTISIFLVLAWILPASQV
LOR25                     MYIFIAALLINSVLFSTTIYPKLLIDFLS--DKQIITYQACLVQFHMFYSLGGSEFLLLAAMAYDRYVSICKPLQYPTIMSKTTISIFLVLAWILPASQV
LOR26                     MYIFIAALLINSVLFSTTIYPKLLIDFLS--DKQIITYQACLVQFHMFYSLGGSEFLLLAAMAYDRYVSICKPLQYPTIMSKTTISIFLVLAWILPASQV
LOR27                     -YVLIAALLINSVVYSTVFYPKLLIDFLS--DKQIITYSACLFQWFIFYSLAGSEFLLLAAMSYDRYVSICKPLQYPTIMRKKTVIMFLVLAWLVPFCKI
```

```
LOR28                 -YVLIAALLINSVVYSTVFYPKLLIDFLS--DKQIITYSACLFQWFIFYSLAGSEFLLLAAMSYDRYVSICKPLQYPTIMRKKTVIMFLVVAWLVPFCKI
LOR29                 -YVLIAALLINSVVYSTVFYPKLLIDFLS--DKQIITYSACLFQWFIFYSLAGSEFLLLAVMSYDRYVSICKPLQYPTIMRKKTVIMFLVLAWLVPFCKI
LOR31                 MYFFIMCLSINSLYGSAGFFFRFLRDLLY--DTHLISRSACFTQTYVIYSYASYELTLLGIMAYDRYVAVCQPLHYHNKMTSKLVSKLVTFAWTYPAFSV
LOR32                 MYFFIMCLSINSLYGSAGFFFRFLRDLLY--DTHLISRSACFTQTYVIYSYASYELTILGIMAYDRYVAVCQPLHYHNKMTSKLVSKLVTFAWIYPAFSV
LOR33                 MYFFIMCLSINSLYGSAGFFFRFLRDLLY--DTHLISRSACFTQTYVIYSYASYELTLLGIMAYDRYVAVCQPLHYHNKMTSKLVSKLVTFAWIYPAFSV
LOR34                 MYFFIMCLSINSLYGSAGFFFRFLRDLLY--DTHLISRSACFTQTYVIYSYASYELTLLGIMAYDRYVAVCQPLHYHNKMTSKLVSKLVTFAWIYLAFSV
LOR35                 MYFFIMCLSINSLYGSAGFFFRFLRDLLY--DTHLISRSACFTQTYVIYSYASYELTLLGIMAYDRYVAVCQPLHYHNKMTSKLVSKLVTFAWIYPAFSV
LOR37                 MYLLICMLCVIDILVVTAIIPNMLLGLLF--DWDETSLAGCLTQMFFTHFLSSLESTLLLVMALDRYVAICHPLRYTEIVDSSLFMKLLLFTLLRSGSIM
LOR38                 MYLLICMLCVIDILVVTAIIPNMLLGLLF--DWDEISLAGCLTQMFFTHFLSSLESTLLLVMALDRYVAICHPLRYTEIVDSSLFMKLLLFTLLRSGSIM
LOR39                 MYLLICMLCVIDILVVTAIIPNMLLGLLF--DWDEISLAGCLTQMFFTHFLSSLESTLLLVMALDRYVAICHPLRYTEIVDSSLFMKLLLFTLLRSGSIM
LOR40                 MYLLICMLCVIDILVVTAIIPNMLLGLLF--DWDEISLAGCLTQMFFTHFLSSLESTLLLVMALDRYVAICHPLRYTEIVDSSLFMKLLLFTLLRSGSIM
LOR41                 MYLLICMLCVIDILVVTAIIPNMLLGLLF--DWDEISLAGCLTQMFFTHFLSSLESTLLLVMALDRYVAICHPLRYTEIVDSSLFMKLLLFTLLRSGSIM
LOR42                 MYLLICMLCVIDILVVTAIIPNMLLGLLF--DWDEISLAGCLTQMFFTHFLSSLESTLLLVMALDRYVAICHPLRYTEIVDSSLFMKLLLFTLLRSGSIM
LOR43                 MYFILFSLPLNDLVGITAMLPKVLSDIVTETNK--VYYPLCVLQAFLLHMYGGGILFILAAMSFDRYAAICMPLRYSTIMTPRIVVCIISLVWGLDFVLI
LOR44                 MYFILFSLPLNDLVGITAMLPKVLSDIVTETNK--VYYPLCVLQAFLLHMYGGGILFILAAMSFDRYVAICMPLRYSTIMTPRIVVCIISLVWGLDFVLI
LOR45                 MYFILFSLPLNDLVGITAMLPKVLSDIVTETNK--VYYPLCVLQAFLLHMYGGGILFILAAMSFDRYAAICMPLRYSTIMTPRIVVCIISLVWGLDFVLI
LOR46                 MYFILFSLPLNDLVGITAMLPKVLSDIVTETNK--VYYPLCVLQAFLLHMYGGGILFILAAMSFDRYVAICMPLRYSTIMTPRIVVRIISLVWGLDFVLI
LOR47                 MYFILFSLPLNDLVGITAMLPKVLSDIVTETNK--VYYPLCVLQAFLLHMYGGGILFILAAMSFDRYVAICMPLRYSTITTPRIVVCIISLVWGLDFVLI
LOR48                 MYLLFCNLPINDILGNSILLPRLLVDILLPPSERLISYYECVVQAFTTHMFGTTSHTVLMIMAFDRYVAICKPLHYTAIMTNKMVMKLTVSAWGVAFVLV
LOR49                 MYLLFCNLPINDILGNSILLPRLLVDILLPPSERLISYYECVVQAFTTHMFGTTSHTVLMIMAFDRYVAICKPLHYTAIMTNKMVMKLTVSAWGVAFVSV
LOR50                 MYLLFRNLPINDILGNSILLPRLLVDILLPPSERLISYYECVVQAFTTHMFGTTSHTVLMIMAFDRYVAICKPLHYTAIMTNKMVMKLTVSAWGVAFVLV
LOR51                 MYLLFRNLLINDILGNSIKLPRLLLDMLRPPSERLISYYECVVQAFTTHMYGTASHTVLMIMAFDRYVAICNPLRYSTIMNDKMVMKLTVSAWGVAFVLV
LOR52                 MYLLFCNLPINDILGNSILLPRLLVDILLPPSERLISYYGCVVQAFLTQFFGTTSHTVLMIMAFDRYVAICYPLRYSAIMSNKMVTKLSVSAWGSAFVLV
LOR53                 MYLLFCNLPINDILGNSILLPRLLVDILLPPSERLISYYACVVQAFITQFFGTTSHTVLMIMAFDRFVAICYPLRYSTIMTNKMVMKLTVFAWGSAFFFV
LOR54                 MYLLFCNLPVNDILGNSILLPRLLLDMLRPPSERLISYYECVVQAFTSHMFGTTSHTVLMIMAFDRYVAICKPLHYTAIMTNKMVMKLTVSAWGVAFVLV
LOR55                 MYLLFCNLPVNDILGNSILLPRLLLDMLRPPSERLISYYECVVQAFTSHMFGTTSHTVLMIMAFDRYVAICKPLHYTAIMTNKMVMKLTVSAWGVAFVLV
LOR56                 MYLLFCNLPVNDILGNSILLPRLLLDMLRPPSERLISYYECVVQAFTSHMFGTTSHTVLMIMAFDRYVAICKPLHYTAIMTNKMVMELTVSAWGVAFVLV
LOR57                 MYLLFCNLPVNDILGNSILLPRLLLDMLRPPSERLISYYECVVQAFTSHMFGTTSHTVLMIMAFDRYVAICKPLHYTAIMTNKMVMKLTVSAWGVAFVLV
LOR58                 MYLLFCNLPVNDILGNSILLPRLLLDMLRPPSERLISYYECVVQAFTSHMFGTTSHTVLMIMAFDRYVAICKPLHYTAIMTNKMVMKLTVSAWGVAFVLV
LOR59                 MYLLFCNPPVNDILGNSILLPRLLLDMLRPPSERLISYYECVVQAFTSHMFGTTSHTVLMITAFDRYVAICKPLHYTAIMTNKMVMKLTVSAWGVAFVLV
LOR60                 MYLLFCNLPVNDILGNSILLPRLLLDMLRPPSERLISYYECVVQAFTSHMFGTTSHTVLMIMAFDRYVAICKPLHYTAIMTNKMVMKLTVSAWGVAFVLV
LOR61                 MYLLFCNLPVNDILGNSIMLPRLLLDMLRPPSERLISYYECVVQAFISHMFGTASHTVLMIMAFDRYVAICKPLHYTAIMTNKMVMKLTVSAWGVSFVLV
LOR62                 MYFLFRNLLINDILGNSIKLPRLLLDMLRPPSERLISYYECVVQAFSTHMFGTTSHTVLMIMAFDRYVAICYPLRYSAIMSNKMVMKLTVSAWGVAFVLV
LOR63                 MYFLFRNLLINDILGNSIKLPRLLLDMLRPPSERLISYYECVVQAFTTHMYGTASHTVLMIMAFDRYVAICNPLRYSTIMNDKMVMKLTVSAWGVAFVLV
LOR64                 MYLLFRNLLINDILGNSIKLPRLLLDMLRPPSERLISYYECVVQAFTTHMYGTASHTVLMIMAFDRYVAICNPLRYSTIMNDKMVMKLTVSAWGVAFVLV
LOR65                 MYLLFRNLLINDILGNSIKLPRLLLDMLRPPSERLISYYECVVQAFTTHMYGTASHTVLMIMAFDRYVSICNPLRYSTIMNDKMVMKLTVSAWGVAFVLV
LOR66                 MYLLFCNLSINDVMGNSLLVPRVLADILVPPSDRLIHYYECVMQAFTTHMFSTNAHTVLMIMAFDRYVAICNPLRYSIIMNDEMVLKLTVSAWGVAFVLV
LOR67                 MYLLFCNLSINDVMGNSLLVPRVLADILVPPSNRLIHYYECVMQAFTTHMFSTNAHTVLMIMAFDRYVAICNPLRYSIIMNDKMVLKLTVSAWGVAFVLV
LOR68                 MYLLFCNLSINDVMGNSLLVPRVLADILVPPSDRLIHYYECVMQAFTTHMFGTNAHTVLMIMAFDRYVAICNPLHYSIIMNDKMVIKLTVSAWGVAFVLV
LOR69                 MYLLFCNLSINDVMGNSLLVPRVLADILVPPSNRLIHYYECVMQAFTTHMFSTNAHTVLMIMAFDRYVAICNPLHYSIIMNDKMVIKLTVSAWGVAFVLV
LOR70                 MYLLFCNLSINDVMGNSLLVPRVLADILVPPSERLIHYYECVMQAFTTHMFSTNAHTVLMIMAFDRYVAICNPLRYSAIMSNKMVMKLTVSAWGVSFVLV
LOR71                 MYLLFCNLSINDVMGNSLLVPRVLADILVPPSERLIHYYECVMQAFTTHMFSTNAHTVLMIMAFDRYVAICNPLRYSIIMNDKMVLKLTVSAWGVAFVLV
LOR72                 RYILLYNLLFSDTVL---LALSQLMYIMA-VCRILPTYPVCGVLTMLNRLTNEISPLTLVVMCMERYVAVCYPLRHATIITVRNTAIAIIVVWAFSSLNV
LOR73                 RYILLYNLLFADTVM---LGLSQLLYILS-VSRIFPVYPVCGVLIMLTSLTNEISPLTLVVMCLERYVAVCYPLRHATIITIRNTAVAIIVVWAFSSLNV
LOR74                 RYILLYNLLFSDTVL---LALSQLMYIMA-VCRILPTYPVCGVLTMLNRLTNEISPLTLVVMCMERYVAVCYPLRHATIITVRNTAIAIIAVWAFSSLNV
LOR75                 RYILLYNLLFADTVL---LALSQLLYIMA-AFRIFTTYPVCGVLAMLNKLTNEISPLTLVVMSLERYVAVCYPLRHATIITVRNTAVAIIVVWAFSSLNV
LOR76                 RYILLYNLLFSDTVL---LALSQLMYIMA-VCRILPTYPVCGVLTMLNRLTNEISPLTLVVMCMERYVAVCYPPRHATIITIRNTAVAIIVVWAFSSLNV
LOR77                 RYILLYNLLFSDTVL---LALSQLMYIMA-VCRILPTYPVCGVLTMLNRLTNEISPLTLVVMCMERYVAVCYPLRHATIITVRNTAVAIIVVWAFSSLNV
LOR78                 RYILLYNLLFADTFM---LAVSQLLYIMA-ACRLITTYPVCGVVTLLTILTNEISPLTLVVMCLERYVAVCYPLRHATIITVRNTAGAIVVVWAFSSLNV
LOR79                 RYILLYNLLFADTFM---LALSQLLYIMA-ACRLITTYPVCGVITLLTILTNETSPLTLVVMCLERYVAVCYPLRHATIITVRNTAGAIVVVWAFSSLNV
LOR80                 RYILLYNLLFADTFM---LALSQLLYIMA-ACRLITTYPVCGVITLLTILTNEISPLTLVVMCLERYVAVCYPLRHATIITVRNTAGAIVVVWAFSSLNV
LOR81                 RYILLYNLLFADAFM---LALSQLLYIMA-ACRLITTYPVCGVITLLTILTNEISPLTLVVMCLERYVAVCYPLRHATIITVRNTAGAIVVVWAFSSLNV
LOR82                 RYILLYNLLFADAFM---LALSQLLYIMA-ACRLITTYPVCGVITLLTILTNEISPLTLVVMCLERYVAVCYPPRHATIITIRNTGLAIVVIWAICSLNV
LOR83                 RYILFIHLVINDMIQ---VSLTIILFVIS-YTIYRINVSVCCVFILLALFTTENTPLNLACMAAECYIAVCMPLRHVEICTIKRTLMLIGLIWTTTMLSV
LOR84                 RYILFIHLVINDMIQ---VSLTIILFVIS-HTIYRINVSVCCVFILLALFTTENTPLNLACMAAECYIAVCMPLRHVEICTIKRTSMLIGLIWTTTMLSV
LOR85                 RYILFIHLVTNDMIQ---VSLTIILFVIS-FTIYRINVSVCCVFILLALFTTENTPLNLACMAAECYIAVCMPLRHVEICTIKRTLMLIGLIWTTTMLSV
LOR86                 RYILFIHLVTNDMIQ---VSLTIILFVIS-FTIYRINVSVCCVFILLALFTTENTPLNLACMAVECYIAVCIPLRYVQICTIKRTLMLIALTWTTTMLSV
LOR87                 RYILFIHLVINDMIQ---VSLTIILFVIS-YTIYRINVSVCCVFILLALFTTENTPLNLACMAVECYIAVCMPLRHVQICTIQRTLILIGLIWTVTSFNV
LOR88                 RYILFIHLVINDMIQ---VSLTIILFVIS-YTIYRINVSVCCVFILLALFTTENTPLNLACMAVECYIAVCMPLRHVQICTIQRTLILIGFIWTVTSFNV
LOR89                 RYILFIHLVINDMIQ---VSLTIILFVIS-YTIYRINVSVCCVFILLALFTTENTPLNLACMAAECYIAVCMPLRHVEICTIKRTLMLIGLIWTTTMLSV
LOR90                 RYILFIHLVINDMIQ---VSLTIILFVIS-HTIYRINVSVCCVFILLALFTTENTPLNLACTAAECYIAVCMPLRHVEICTIKRTLMLIGLIWTTTMLSV
LOR91                 RYILFIHLVINDMIQ---VSLTIILFVIS-HTIYRINVSVCCVFILLALFTTENTPLNLACMAVECYIAICMPLRHVQICTVRRTLMLIGLIWATSMLSV
LOR92                 RYILFIHLVINDMIQ---VSLTIILFVIS-YTIYRINVSVCCVFILLALFTTENTPLNLACMAVECYIAVCMPLRHVEICTIKRTLMLIGLIWTTTMLSV
LOR93                 RYILFAVTLLSDSLM---LFVSDILLILS-SYRVTMQFGLCIIFYILSVICIFVTPVTLTAMALERYVAICMPLRHAQLCSTRSTMHCILIIHSLSSVPC
LOR94                 RYILFAVTLLSDSLM---LFVSDILLILS-SYRVTMQFGLCIIFYILSVICIFVTPVTLTAMALERYVAICMPLRHAQLCSTRSTMHCILIIHSLSSVPC
LOR95                 RYILFAVTLLSDSLM---LFVSDILLILS-SYRVTTQFGLCIIFYILSVICIFVTPVTLTAMALERYVAICMPLRHAQLCSTRSTMHCILIIHSLSSVPC
LOR96                 RYILFAVTLLSDSLM---LFVSDILLILS-SYRVTMQFGLCIIFYILSVICIFVTPVTLTAMALERYVVICMPLRHAQLCSTRSTMHCILIIHSLSSVPY
LOR97                 RYILFAVTLLSDSLM---LFVSDILLILS-SYRVTMQFGLCIIFYILSVICIFVTPVTLTAMALERYVAICMPLRHAQLCSTRSTMHCILIIHSLSSVPC
LOR98                 RYILFAVTLLSDSLM---LFVSDILLILS-SYRVTMQFGLCIIFYILSVICIFVTPVTLTAMALERYVAICMPLRHAQLCSTRSTMHCILIIHSLSSVPC
LOR99                 ----------------------------------------------------------------ERYVAICMPLRHAELCSTRSTMHCIFIIHSLSSVPC
LOR100                ----------------------------------------------------------------ERYVAICMPMRHAELCSTRSTMHCILIIHGLSSVPC
LOR101                ----------------------------------------------------------------ERYVAICMPLRHAELCSTRSTMYCIIIIHGLSSVPC
LOR102                ----------------------------------------------------------------ERYVAICMPLRHAELCSTRSTMYCILIIHGLSSVPC
LOR103                ----------------------------------------------------------------ERYVAICMPLRHAELCSTRSTMYCIFIIHGLSSVPC
LOR104                ----------------------------------------------------------------ERYVAICMPLRHAELCSTRSTMYCILIIHGLSSVPC
LOR105                ----------------------------------------------------------------ERYVAICMPLRHAELCSTRSTMHCILIIHGFSSVPC
LOR106                ----------------------------------------------------------------ERYVAICMPLRHAELCSTRSTMYCILIIHGLSSVPC
LOR107                ----------------------------------------------------------------ERYVAICMPLRHAELCSTRSTMHCILIIHGLSSVPC
LOR108                ----------------------------------------------------------------ERYVAICMPLRHSQLCSIHSTKHCILIIHSLSSVPC
LOR109                ----------------------------------------------------------------ERYVAICMPLRHAELCSTRSTMHCIIIIHGFSSVPC
LOR110                ----------------------------------------------------------------ERYVAICMPLRHAELCSTRSTMHCILIIHGLSSVPC
LOR111                ----------------------------------------------------------------ERYVAICMPLRHAELCSTRSTMHCILIIHGLSSVPC
Fugu_OR123-1          MYIFLCNLCINGLYGTTGFYPKFLIDILS--TSHVISYVGCLLQAFVLHSSACADFSILVLMAYDRYVAICRPLAYHSVMNPQRVYLLIFITWLIPFCSL
Fugu_OR6765-1         MYIFLCNLCINSLYGTAGFYPKFLIDILS--TSHVISYAGCLVQSLVVHSSVCADFSILVLMAYDRYVAICQPLVYHSVMNPQRVSALVFAAWMLPLTQF
Fugu_OR4133-1         MYIFLCNLCINSLYGTAGFYPKFLIDILS--TSHIISYAGCLVQSLVVNSSACAVFSFLALMAYDRYVAICQPLVYHSVMNPQRMCLLVFAAWILPVGQL
Fugu_OR3630-2         MYVLIAALLINSVLYSTAIYPKLLIDFLS--EEQTITYSVCLFQWFMFYSLGGSEFLLLAVMAYDRYVAICKPLQYSAIMRRRTVTIFLLLAWIAPTAQV
Fugu_OR8617-1         MYVLIAALLINSVLYSTAIYPKLLIDFLS--EEQTITYSACLFQYFMFYSLGASEFLLLAVMAYDRYLAICKPLQYSAIMRRRTVTIFLLLAWIAPTAQV
Fugu_OR5510-1         MYVLIAALLINSVLYSTAIYPKLLIDFLS--EEQTITYSVCLFQWFMFYSLGGSEILLLAVMAYDRYLAICKPLQYSAIMRRRTVTIFLLLAWIAPTAQV
Fugu_OR117-1          MYIFIMCLSVNSLYGSAGFFFRFLKDLLS--STHLISRWGCFAQIYVIYTYASYELTLLSIMAYDRYVAVCQPLHYHNKITKRSVMRLILLASLYPAFSV
Fugu_OR2346-5         MYIFIALLSVNSLYGSTGFFPRFLMDLLL--DTHFMSRSACFTQIYVFYTYISFEMTILGIIAYDRHVAICNPLHYHRKMTHKTVVLLASSACLIPVFTI
Fugu_OR5287-2         MYLLICSLCVVDVLAATAILPSMLLSLLF--DVDTISLASCLTQMFFTHFLSSLESTLLLAMALDRYVAICQPLRYARIINLSMLARLLLFTLVRSGSIM
Fugu_OR142-1          MYFILFSLPLNDLIGITAMLPKVLSDIVMETNR--VHYGLCVFQAFILHMYGGGILFILAAMSFDRYVAICMPLHYNAIMVPRVVVFIISLVWGLDFSLI
Fugu_OR1026-2         MYLLFCNLPFNDVLGNSILIPRLLMDLLHPPSERLITYYECVVQAFTTHMFGTTSHTVLMIMAFDRYVAICNPLRYAAIMNHKMVIKLTVSAWAVAFVLV
Fugu_OR8298-1         MYLLFCNLPFNDVLGNSILIPHLLMDLLRPPSERLITYYECVAQAFTTHMFGTTSHTVLIIMAFDRYVAICNPLRYAAIMNHRMVIKLTISAWAVAFVLV
Fugu_OR6030-1         RYILLFNLLFADSIQ---MVLGQALYILA-VCLIKMTYPVCGILTALGVLTSNISPLTLVVMSLERYVAVCYPLRHAALITIGNTGLMISVIWAFSLLSV
Fugu_OR59-1           RYILLFNLLFADSIQ---MVFSQVLYILA-TCLIKMSYPLCGIVNVVGVITSDISPLTLVVMSLERYVAVCYPLRHAAVITIRNTGLMIYVIWAFSLLSG
Fugu_OR4208-3         RYILFFHLVVNDMIQ---VMLTVMLFIIS-YVLYKINVSFCGVFILLALITTENTPLNLACMALECYIAVCFPLRHGQICTIRRTLLLIGLIWTTSMFSV
Fugu_OR4208-2         RYILFIHLVINDLIQ---VNVTIMLFVIS-YTIYRINICMCWVFMLLAVVATENSPLNLACMALECYSAVCFPLHHLQVWTIRRTLTLILLMWTTTTVSA
Fugu_OR6818-1         RYILFAVTLLSDSVL---LLLTDVMLILS-YFAVTIEMWVCVTLYVVLALYTFVTPVTLTVMTLERYVAICLPLRHSELCSMRRALHCILLVHGVSFLPC
Fugu_OR7903-1         RYILFVITLVSDSVF---LIIANLLLILH-STYFTIEVSLCIVFYLIVCLSVFVTPVTLTAMTLERYVAICLPLRHPELCSLHNTQKCILIILTVSSVPC
Pufferfish_OR8981-2   MFIFICNLCINALYRTAGFYPKFLMDILS--DSHVISYAGCFVQSLVVTSSTCVDFSFLVLMAYDRYVAICRPLVYHSVMNTQRVSVLVFAAWFVPVVQM
Pufferfish_SCAF8981   MFIFLCNLCINALYGTAGFYPKFLMDILS--TSHVISYAGCLVQSLVVTSSTCNDFFFLVIMGYDRYVAICRPLVYHSVMNTQRVSVLVFAAWFVSVGQM
Pufferfish_OR15134-1  MYVLIAALLLNSVIYSSTIYPKLLQDFLS--EEQTITYSACLFQYFAFYSLAGSEFLLLAVMAYDRYVAICTPLRYPAVMRRRRVAALLLLAWVAPSILV
Pufferfish_OR14339-2  MYIFIMCLAVNSLYGSAGFFLRFLRDLLS--SSHLFSRSECFTQIYVIYTYASYELTFLSVMAYDRYVAVCQPLHYHNKITKRTVILLIGLAVTIPAITV
Pufferfish_OR14328-1  MYIFIMCLAVNSLYGSAGFFLRFLRDLLS--SSHLISRPECFVQIYVIYTYGSFELSFLSVMAYDRYVAVCQPLHYHNKITKKTVILLIGLAVTIPAIIV
Pufferfish_OR14677-8  MYLLICSLCVVDILAATAIFPSMLLSLLF--DMDKISLASCLTQMFFTHFLSSLESTLLLAMALDRYVAICQPLRYARIINFSMLVRLLVFTFVRSSSIM
Pufferfish_OR14536-2  RYVLLFNLLFADTLQ---MVLCQVLFILA-NCWIKMSYPVCGFLNVIGVLTSDISPLTLVVMSLERYVAVCFPLRHAAVITTRNTGLAIFAIWAFSLFSS
Pufferfish_OR14536-1  RYVLLFNLLLADTIQ---MVTSQLLYILA-ACLIRLTYPVCGFLNVIGVLTSDISPLTLVVMSLERYVAVCFPLRHAAVITTRNTGLAIFAIWAFSFFSS
Pufferfish_OR10960-2  RYILFFHLVVNDMIQ---VTLTVLLFVIS-YVLYKIEVSVCCVFILLALFTTENTPLNLAAMALECYVAVCRPLRHAQICTVRRTLLLIALIWTTSCVSI
Pufferfish_OR10960-3  RYVLFIHLVINDMLH---VNLTMVLFLIS-YTVYRVNVCVCWLLLLPALITTENTPLNLAAMALECYVAVCLPLRHAQICTVRRTRTLILLMWTTTVCSS
Pufferfish_OR12434-1  RYILFTITLVSDSIT---LIISDLLLLLN-VFSVPLQVSLCIICYLIVLFCGFITPVTLTAMTLERYVAICLPLQHPRLCSLHNTWNCMLIINALSCVPC
Medaka_ORUn.33        MHLLICALCLVDILVVVAILPNMLLGLTL--DHSRISLAACLTQMFFTHFLSSVESTLLLTMALDRLAAICEPLRYRFIMDSCMLLRLLLFTLLRSGSIM
Medaka_ORUn.2         MSVLIAGMAVVEVCLPVFFVPNMLLSFLF--GWRDISLIGCLTQMHFIHTFGAFQTTFLVWMALDRYFAICRPLYYHQQMALQSFIRFVVPLVLRNVVVI
Medaka_OR21.18        MYIILCSLCINGLYGTAGFYPKFLSDLLS--DRYVISYIGCFLQIYVIYSYAKVDFSIIALMAYDRYVAICRPLEYHSVMSVQRTAVLVMLSWLVPLCCE
Medaka_OR21.19        MYILLCAFCINGLYGTTGFYPKFLWDLLS--PVHMISYYGCLIQAQVMYSFACSDLSILSVMAYDRYVAICRPLEYHFRMSKQNVVLLVCFSWFTPFCIM
Medaka_OR21.20        MYILLCAFCINGLYGTTGFYPKFLWDLLS--PVHMISYYGCLIQVQVMYSFACSDLSILSVMAYDRYVAICRPLEYRSLVSKQKVLVLVGFSWFTPFCIV
Medaka_OR13.14        RYILFAVTLLSDCLF---LMLANVTLIMT-YFGFTIQMWLCLIIYLLTSVYSYVTPVTLTAMTLERYVAICMPLRHAELCTPHGALQLILIIHSISAVPC
Medaka_OR13.12        RYILFAETLLSDCLF---LILTDSLLILS-FFGCTMQMWLCLFIYIILSVYSFVTPITLTAMTLERYVAICMPLRHAELCTPHGALQLILIIHSISAVSC
Medaka_ORUn.23        RFVLLFNLLLADTLQ---LAQSQLHLLFS-ACRVQLAYSLCIALNMIVNLTTGISPLTLVLMPLERYVAVCYPLRHASIITIRNTAAAVIAVWAVSSLNN
Medaka_ORUn.21        RFILLYNLLLADTLQ---LAQSQLHLLLS-VCRVKVAYSLCIALNMIVNLATGISPLTLVLMPLERYVAVCYPLRHASVITVRNTVAAVIAVWAVSSLNS
Medaka_OR14.15        RFILLYNLLLADTLQ---LAQSQLHLLLS-VCRVKVAYSLCIALNMIVNLATGISPLTLVLMPLERYVAVCYPLRHASVITVRNTVAAVIAVWAVSSLNS
Medaka_OR14.13        MYLLFCNLSVSDVIGNTYMIPRLLSDILKPPSERLISYYECVLQAFTSHMFGTTSHTVLMIMAFDRYVAICYPLRYANIMTNRMVLKLTVFAWGVAFVLV
Medaka_OR14.4         MYLLFCNLPVSDIVGCTHILPRLLSDMLVPPSERLISYYECVVQAFTTHMFGTTSHTVLMVMAFDRYVAICNPLRYANIMTSRMIIKLTVFAWGVPFVWV
Medaka_OR14.6         MYLLFCNLPVSDIVGCTHILPRLLSDMLVPPSERLISYYECVVQAFTTHMFGTTSHTVLMVMAFDRYVAICNPLRYANIMTSRMIIKLTVFAWGVPFVWV
Medaka_OR13.4         MHLILLNLPINDLIGSSALFPQLIKEILTNSRR--IQYSACVAQAFFIHVYAAGTVFHLSAMAYDRFLAICYPLQYNTVMTNAHIMKLIALVWSSSLVLI
Medaka_OR13.5         MFILLFSLPVSDIVGATAFFPHLVFSILTQNRL--ISSEACITQAFFVHFYGTGNLMFLTAMAYDRYMAICSPLKYNAVMNSQNLLRIICLIWFLNFALM
Medaka_OR13.6         MFILLLNLPISDIVGATALFPHFLFSIITESRL--ISSEACITQAFFIHIYGTGNLIILSAMAFDRYIAICCPLNYNSILTSQNLMRIIFLVWFLNVSMI
Medaka_OR13.7         MYFILFSLPLNDLIGITAMMPKVLSDIVTETND--VYYPLCVLQAFLLHMYGGAVLFILAAMSFDRYVAICMPLRYSSIMTLRMVTGIVTLVWGLDFVLI
Medaka_ORUn.1         MYFFLCNLCINGICGASSFYPKLLHDLLA--DAHVVSYAGCVAQMFVGYSYIFCEFTSLTVMAYDRYVAICSPLRYQELMTLQKVALLLLLTWTFSLLET
Medaka_OR21.2         MYLFIAGLLINSVLFSTNIYPKLLTDLLA--TKQVISYQACLFQVFVFYSFSCSEFLLLAAMSYDRYVSICKPLQYTIIMRRTIVFVLLGLAWIVPFCHL
Medaka_OR21.3         MYIFIASLLINSVLFSTNIYPKLLADFLS--NEQVISFQACLLQRIFFYSFGGSEFFLLAVMSYDRYVSICKPLQYAIIMKKIKAVVFVGLAWGVPVCHL
```

```
Stickleback_OR1.1        MYVLIAALCAVDLLAATAIVPKMLLGLLF--DWNRISLASCLAQMFSTHFLSSLESTLLLAMALDRYLAVCQPLRYTEMVGASALSRLLLLALVRSGSIM
Stickleback_OR16.3       MYILLCSLCMNGLYGTTGFYPKILWDLLS--PVHVISYSGCLVQAFVIHAFVSNDMSILAVMSYDRYVAICRPLVYHSVMSKKRLLILVSFSWFTTFCIS
Stickleback_OR1.12       RYILFATTLLSDSIF---LIITDILLILS-YIKITMQVWLCVIIYIISSLCIFVTPVTLTAMTLERYVAVCRPLRHGELCSTRRALHCVLIIHSISSIPC
Stickleback_OR1.13       RYILFATTLLSDSLF---LIITDILLIFS-YIKITMQMWLCVIIFIVSSLYTFVTPVTLTAMTLERYVAICKPLRHGELCSTRRALHCVLIIHSISAIPS
Stickleback_ORUn.9       RYVLLLNLLCADTAQ---MALSQLLYLLA-ASRAPLTYPACGVLVMLADLTNAVSPLTLVAMSLERYVAVCRPLRHAALVTPRSTMAAVAAVWAFSSLNV
Stickleback_OR7.1        RYILFANLLIADSLQ---LLSAMLMYIFA-VTMIKLSSFLCALVVLVARVTVKTSPLNLAMMSLERYVAICFPLRHASIVTTRMTGAAVAVMWTVGSLES
Stickleback_ORUn.6       MYLLFCNLPLNDLLGNSILLPRLLLDMLRPRSELFISYYECVVQAFTTHVFGTTSNTVLMIMAFDRYVAICYPLQYNMIMTNKMVMKLTASAWGVALVLV
Stickleback_ORUn.8       MYLLFCNLPLNDLLGNSILLPRLLLDMLRPRSELFISYYECVVQAFTTHMSGTTAHTVLMIMAFDRYVAICYPLQYNMIMTNKMVMKLTASAWGVALVLV
Stickleback_OR1.18       MYFILFSLPLNDLIGVSAMLPKVLSDLVTETNT--VYYPLCVLQAFLLHMYGGGILFILAAMSFDRYAAICMPLRYSSIMTPRVIICIISLVWGLDFVLI
Stickleback_OR16.10      MYIFIMCLSINSLYGSAGFFFRFLRDLLS--DTNLISRTACFAQIYVIYTYASYELTLLGIMAYDRYVAICQPLNYHNKITSKAVSKLVAFAWIYPSFSI
Stickleback_OR16.32      MYIFLCNLCINGLYGTAGFYPKFLMDLLS--PTHVISYAGCLLQGFVLHSSACADFSLLVLMAYDRYVAICRPLVYHSGMTRQRMCVFVFFAWLIPFLLI
Stickleback_OR16.1       MYIFIMCLSINSLYGSAGFFFRFLRDLLS--DTHLISRTACFAQIYVIYTYASYEMTLLGIMAYDRYVAICQPLNYHNKITSKAVSKLVAFAWTYPCFSI
Stickleback_OR16.5       MYIFIMCLSINSLYGSAGFFFRFLRDLLS--DTHLISRTACFAQIYVIYTYASYELTLLGIMAYDRYVAICQPLNYHNKMTSKAVSKLVAFAWIYPCFSV
Stickleback_ORUn.1       MYIFIAALLGNSVVFSTAIYPKLLMDFLS--EEQVISYPACLFQCFLYYSLDGSEFLLLSAMAYDRYVSICKPLQYHTIMKKTTVRVLLAAAWCFPAAQI
Stickleback_ORUn.17      MYIFIAALLINSVLFSTAIYPKLLIDFLS--EKQIISYPACLLQWFSSHWLAVSDFFLLSIMAYDRYVSICNPLQYTTIMQRTTVYIFLCSAWILPACLV
Zebrafish_OR126-7        MYVFICNLCANALFGTAGFYPKFLYDLLS--QDHVISYAGCMIQTFVIYSYALCDVSTLTVMAYDRYVAICRPLEYHSVMTSQRVVEWILFCWLAPLFCM
Zebrafish_or126-3        MYIFLCNLCINGIFGTSGFYPKFMYDLLA--PEHVISYAGCLIQIFVIYSSVFCDFSTLTVMAYDRYVAICRPLEYHSMMTNQKILKCVLFCWLSPFFCM
Zebrafish_OR123-1        MYIFLCNLCINGLYGATGFYPPLLYYLLS--ESNVISLKECAIQSFAIFTYAIGEFTNLSVMAFDRYLAICRPLYYHNIMTTVTVWRLLTFIWMFPCCTA
Zebrafish_OR125-1        MYIFLCHLCINGVYGATGFYPKILSDLIL--DSYVISSHMCALQTYVIYSSLLSEITILTVMSYDRYVAICKPLDYHSRLTKNTCVKLILFSWIVPTSFS
Zebrafish_OR127-1        MYILISCLSVNDLYGSAGFFPRLIADLLS--DTNAISRPACFVQIFTIYSYAMSEYTILTLMAYDRYVAICNPLKYHKIMSLRLTARYMAMASFCVVFSM
Zebrafish_OR128-10       MYILISCLSFNSVFGTAAFFPRLLTDLLS--DTHLVPREACLLQAFVIYSYASNENTILMLMAFDRYVAICKPLQYNSIMTPRNLSVLIALSWIYPMLCV
Zebrafish_OR116-2        MYFILFSLPLNDFVGITSMLPKVLSDILTETNK--TYYPLCVLQGFLLHMYGGGVLFVLAAMAFDRYIAICMPLRYNALMTPRAVMGIVALVWGLDLFFI
Zebrafish_zor6a          MYIFVSALAVVDIIVPTAIIPAMLLGFLF--DLNEITLSGCLTQMFVTHFFSSVESTILLAMALDRFVAICKPLHYNEIMNSSMFLKLFLFTLIRSGTIM
Zebrafish_OR117-1        MFYILFSLPLIDLIGITAGLPRVLVDIVTETND--VYYPTCVLQAFLIHLYGGGTLFLLAAMSLDRYIAICNPLRYNSIMTPSRIFGLIAFAWGLDFAVI
Zebrafish_OR115-15       MYMLFCNLPINDILGNSIMVPRLLMDILKSPSQRYISYVECVVQAFSTHTYGTTSHTILIIMAIDRYVAICNPLRYQTIMTNKTVITLSALAWGIALLFI
Zebrafish_OR133-7        RYILFGHMLWVDTLN---LFMSVVLYICA-VSRILIMKNVCLLLLIAATALFQVSTLNLALMSLERYVAICFPLRHAEFTSFKRTHTAICVVWVLGLIQC
Zebrafish_OR132-1        RYILFGHMLMNDSVL---LLVTTVMYTLA-LCFLPIHKSICTLLVLISYCTFFNAPLTLALMSLERYVAICFPLXHCTIAHPKRTGIAIGIIWFLSSVNV
Zebrafish_OR134-1        RYILFIHMVINDAVQ---LTVTIALFLVS-YILYTISVGVCCFFILVAVFTTRSTPVNLAGMAIERYIAICYPLRHAQICTVHRAYVLIGVIWFVSVVPD
Zebrafish_OR131-2        RYILFAQTLFVDSAL---MLFADLTLVGS-AYELFIHIISCYIFCTVMALLSICSPVTLVAMCLERYVAICLPLRHASISSPKNTINGLLIIWGVSSVIP
Zebrafish_OR131-1        RYILFAQTLFVDSSF---MIFADLTLLCS-AYQLIIHNISCYVFCTVMSVLSICSPVTLVAMCLERYVAICLPLRHASISSPKNIINGLLIVWGVSSVIP
Goldfish_OR3             MYVFICNLCMNGLFGTAGFYPKFMYDLLS--QYHLISYAGCLVQIFVIYSSVLCDFSTLTVMAYDRYVAICRPLEYHSVMTNQRIIECILFCWLSPFFCM
Goldfish_OR2             MYNIILNLVVSDLLFSTTTLPKIISRYWFQ--DGSISFTGCFIQMYFVHYFGSVSSVILAIMAFDRYVAICNPLRYPNIVTKLNIFCLCLAAWVLTNTCP
Rainbow trout OR500-1    MYIFLCSLCVNGLYGTAGFYPKFLLDLQS--DVQVISYGGCFTQAYVIYTSVMCELSTLTVMSYDRYVAICRPLLYHTIVTSLTVRKLLLFSWCYPLFIA
Cutthroat trout OR600-1  MYILFCNLPLNDIVGATVMIPRLLWDILTTAPERYITYTECVIQAFYTHVYATTSHTVLMIMAFDRYVAICNPLRYATIMTNKMVIRLSMSAWGVAIVLV
Brown trout OR500-1      MYIFLCSLCVNGLYGTTGFYPKLLLDLQS--DVHVISYGGCFTQAYVIYTSVMCEISTLTVMSYDRYVAICRPLLYHTIVTSLTVRKLLLFSWCYPLFAG
Atlantic salmon SORB     MYILSCNLPFNDIIGNSITFPRLLADILVPASERYITYSECVVQAFCTQMFGTTSHTILMIMAFDRYVAICNPLRYSIIMTNKMIIRLSVSAWGVAIVLV
Medaka_mfOR2             MYIFIAALPTNSVMFSMAVYPKLLVDILS--KRQIITYYACFFQHGVYYCLGASEFLLLTAMSFDRYVSICKPLQYQSLMSKRRVTVMLILSWLWPLFQL
Medaka_mfOR1             MYIFIAALLTNSVMFSMAVYPKLLVDILS--KRQIITYYACLLQYVVYYCLGASEFLLLTAMSFDRYVSICKPLQYQSLMSKRRVTVMLILSWLWPLFQL
Medaka_OR_Y3             MYLLFCNLPINDVMGNSIMIPRLLSVILKPPSERLISYYECVLQAFTAHMFGTTSNTVLMIMAFDRYVAICNPLRYTNIMTNRMVLKLTVFAWGVAFVLV
Medaka_ORY1              MYLLFCNLPVSDIVGCTHILPRLLSDMLVPPSERLISYYECVVQAFTAQMFGSTSHTVLMVMAFDRYVAICNPLRYANIMTSRMIIKLTVFAWGVPFVWV
Frog_OR-52D1             MFIFIAFLCINGLYGSVIFFPFLFVNLLS--KTQVISYVGCIIQVFSIHTYISCEVTILAVMALDRYVCICNPLRYSSIMSLATVFKLIGAAWLYVIALI
Frog_OR-52E8             MYMFIAALCMNGLYGGICFFPALFVNLLS--QVQTVSYIGCLIQVFGIHSYYGCEITVLAVMAFDRYMCICNPLRYHSLMTSGTVLKLIGAAWLYIIVLV
Frog_OR-52A1             MYLLISVLFAINITSCTAILPKFLLDLLF--QLNQVSLTGCLLQMFVIYFMAVCESNVMLLMSLDRYVAICRPLRYHNIMTKNLLAWLTVTVIVRSFILV
Frog_OR-52P1             MYFLISLLFAINITSITTTLPKFLLDLLF--HLNQVSLTGCLLQMFVIYFVTACESTVLLLMSLDRYIAICRPLRYHNIMTTSFLAWLTVIVLIRNCLLI
Frog_OR-52A5             RYVLFAHMLINDVVY---LVMTLYLAIITSYLPMRLPAHVCYAIVMLSSASYKVTAYNLGVMALERYVAICFPLRHSEFCTRQRSAIALVGIWAIGLIPN
Frog_OR-2M5              RYVLFAHILVNDLLY---LSVGFYLLVSYLYLVF-IPVPVCYIIVTLASCTFWVTPYNLALMSLERYVAICHPLRHTEICSGHRLTMAIALMWTLGLTPN
Frog_OR-2M2              MYIIIRNLALSDLLFDTITLPKIIAKYWFG--AGSITFYGCFFQLFCVNSLGSLYSFIIMLMAIDRYVAICQPLRYHSIISNKLVTLLCYFLWVFAALIG
zebrafish_OR10.1         KFMAVGNLAAVDLILSTALVPGMIKTYIAL--DNFVPFNLCLLQMYIYYTCTCLESFSLCILSYDRFIAICFPLRHESINTNSRMAYIISAVWFFCVVTL
zebrafish_OR10.14        KYIAVFNLSLSDICESTAVIPQLLDTFLFR--NQLIPYRLCLYNMFSVFVSISTQSLTLAVLSFDRLVAICLPLRYHMIVTFRSMLVIIGLTWALTLLMV
zebrafish_OR15.39        KYLAVCNLAVVDISINSVIIPQMVPVFVFN--QNYISFGACFSQMFFMHFFGDMESFSLALLAYDRLIAICWPLRYSTINTNLRMLLIIALIWILVTLLD
Medaka_ORUn.6            KFLAVINLAVIDLCFTACSIPGMIKIFLFK--DNFVPFNQCLVQMFFYYVFAALESYALMILAYDRLVAICFALRQNSINTVRSMCCIITITWCYILGVT
Medaka_ORUn.28           KYVAVFNLAFVDLLGNTALVPKVIDTFLFN--HYYISYNECLTYFFFCYMCLSMQALNLVALCYDRLVAIMFPLHYQVTVTPKIMLSIILAFWLFVIIAV
Medaka_ORUn.27           KYIAVFNLAFVDLLGNSALVPKVLDIFLFN--HNVIPYNDCMTFLFFCYTCLCMQSFNLVALSYDRLVAIIFPLHYQLRVNQTFMLSMIASFWVFTITAV
Stickleback_OR7.3        KFMAVFNLAVIDVILSTCTIPSMIKVFLFK--DNFVPFNLCLVQMYFYYAILSMESYALAILAYDRFIAICLPMRQNLFNTLPIMLCIVGGTWVYNLGRI
Stickleback_OR7.4        KFLAVINLAVIDIVLNTTTIPGMVKIFLVK--DNFIPFNLCLVQMFSYYCFASMESFALAILAYDRLIAICFPLRHNSVNTLQSMSCIIGFTWSFSVGVI
Stickleback_OR7.8        KYIAVFNLAFVDLLGSSTLMPKLLDIFLFN--HRYISYNDCLTFLFFSYTFLCMQSFNLLALSYDRLIAIIHPLHYQVRVTHRFMLSLIAFFWVFATTAV
Fugu_ORUn.83             KYVAVFNLAFVDLFGNSALVPKLLDIFLFD--HRHIPYNDCLTFLFFCYTCLSMQALNLVALAYDRLVAIIFPLHYQMRVTHRIMMSLIASFWAFIIVAV
Fugu_ORUn.20             KFLAVVNLAVIDIVLNSTTIPSMIKTFLLK--DNFFPFNICLLQMFVYYSSISLESFALAILAYDRLIAICFPLQQNSINTLRSMSCIVAVTWSYSLGLV
Pufferfish_OR7.1         KYVAVFNLAFVDLFGNSALVPKLLDIFLFD--HRRIPYNDCLTFLFFCYTCLSMQALNLVALAYDRLVAITYPLHYQVRVTHRFMISMIASFWAFIIVAV
Pufferfish_OR7.3         KFLAVVNLAVIDIVLSSSTIPSMIRTFLLK--ENFFPFNLCLLQMFTYYSFATLESLALAILAYDRLIAICFPLQHNSINTLWSMSWIVAVTWCYSLGLA
zebrafish_OR10.29        MYIILASLALSDMCFSTVALPKIISKYWFN--AGATPFHACFFQMELIHYFGSLNSLIMMIMALDRYVAICYSLRYQTVMTNRVTYILITTAWITAFIAP
zebrafish_OR10.31        MYIIMLSLSVSDICFCTVALPKVISRYWFN--DGFISFYVCLFQRQLIHYFGTLNSLIMMLMALDRYLAICHPLRYPVLMTNRTMRLLVGFSWVTAMIAP
zebrafish_OR15.58        TYIIFCNLAMADLALGTTTYPRVIAKYWMS--DKIISFSGCFTQMFFIHFLGATNSFLMALMALDRFIAICNPLRYHTLIKNSTILIICAFVWTANMLQL
zebrafish_OR15.57        TYLIFCNLAVCDIAIGTVTLPKAIAKYFFD--SSEITFLSCFVQMFFVHYLGSINSFLMLLMAVDRFVAVCNPLRYCVLITNKTILLACALIWMLCISWL
Medaka_OR13.1            SYLIFAHLAMTDLIFGTLTLPKVIARYLWN--DMITPFTTCFAQMFFVHSLGAIHSFILLVMALDRFVAVWFPLRYPAIITNKTTSIACSLCWTLTFIRM
Medaka_OR13.3            SYLVFCHLALNDLTFSTFTIPKIIAKYWFA--DSDISFNSCFTQMFMVHYLGSVMSFLLLVMALDRFIAVCIPLRYPVLITNNIISVLCGFAWFIPAPLM
Stickleback_OR1.25       TYLLFCHLALNDLTFGTLTLPKIISKYWFG--DGFISFYGCFTQMFFVHYSGSVTSFMLLAMALDRFIAICIPLRYPVLVTNNTISVICGFAWFIPLPLM
Fugu_ORUn.8              MYLVFCHLALIDLTFSTVTIPKIISKYWFS--GGDISFYGCFAQMFFVHYLGSVTSFILLVMALDRFISICFPLRYTALVSKKVISALCGIAWFIPVSLL
Pufferfish_OR16.7        MYLVFCHLALIDLMFGTVTLPKIISKYWFG--NGDISFHGCFTQMFFVHYLGSVTSFILLVMALDRFISICLPLRYTALVSTKFISALCGIAWFFPVSLM
Frog_OR669.1             MYKIMVNLAVSDLLFDTITLPKIIAKYWFG--AGSIPYCVCLLQLFCVHFLGSFDAYIIMLMALDRYIAICHPLRYACIMTNRTTAILCCFFWIFAAAIS
Amphioxus_105.1          RYFLRCHLAVNDILHALVIIPRYLDLIMQ--SNSRFYGISCSDVRIFTSAVALSKYGIYLLMAIDIYYFICYPLQYETKVTTSRLAIGMVLVDTFSFICT
Amphioxus_39.1           RHFLRCNLALDDIIFTGSMIPLEIYGLFV---DDSVVRRLCGVQNMLIPTFTASMFGAYLLMATELYHFICNPLHYRSKVTTRRVIIGIVSVRAFALIFG

                                210       220       230       240       250       260       270       280       290       300
                         ....|....|....|....|....|....|....|....|....|....|....|....|....|....|....|....|
LOR1                     FMSSI--TTARSRLCG--SHIPKIYCINFLVGKLACTTS--IANVIIPAFNYTFYFLHVMFIAWSYMYLI-RKCLI--SSENRSKFMQTCLPHLICLIIV
LOR2                     FMSSI--TTARSRLCG--SHIPKIYCINFLVGKLACTTS--IANVIIPAFNYTFYFLHVMFIAWSYMYLI-RKCLI--SSENRSKFMQTCLPHLICLIIV
LOR3                     FMGSI--TTARSRLCG--SHIPKIYCINFLVGKLACTTS--IANVIIPAFNYTFYFLHVMFIAWSYMYLI-RKCLI--SSETRSKFMQTCLPHLICLIIV
LOR4                     FMGSI--TTARSRLCG--SHIPKIYCINFLVGKLACTTS--IANVIIPAFNYTFYFLHVMFIAWSYMYLI-RKCLI--SSENRSKFMQTCLPHLICLIIV
LOR5                     FMGSI--TTARSRLCG--SHIPKIYCINFLVGKLACTTS--IANVIIPAFNYTFYFLHVMFIAWSYMYLI-RKCLI--SSENRSKFMQTCLPHLICLIIV
LOR6                     FLSSI--TTLISKLCG--SHIPKIYCVNTLIGNLACSAS--IAKIIIPAFNYTFYFGHVISVVWSYIYLI-RTCRT--SKEGRSKFMQTCLPHLISLIVV
LOR7                     FLSSI--TTLISKLCG--SHIPKIYCVNTLIGNLACSAS--IAKIIIPAFNYTFYFGHVIFVVWSYIYLI-RTCRT--SKEGRSKFMQTCLPHLISLIVV
LOR8                     FLSSI--TTLISKLCG--SHIPKICCVNTLIGNLACSAS--IAKIIIPAFNYTFYFGHVIFVVWSYIYLI-RTCRT--SKEGRSKFMQTCLPHLISLIVV
LOR9                     FLSSI--TTLISKLCG--SHIPKIYCVNTLIGNLACSAS--IAKIIIPAFNYTFYFGHVIFVVWSYIYLI-RACRT--SKEGRSKFMQTCLPHLISLIVV
LOR10                    FLSSI--TTLISKLCG--SHIPKIHCVNTLIGNLARSAS--IAKIIIPAFNYTFYFGHVIFVIWSYIYLI-RTCRT--SKEGRSKFMQTCLPHLISLIVV
LOR11                    VLPTA--MSAKEKLCR--FILNGVFCNN-TIYKLQCVISQ-ARFVRDMVILLNVALVPVLFILFTYTRIF-VISYRS-SKEVRKKAAQTCLPHLIVLINF
LOR12                    VLPTA--MSAKEKLCR--FILNGVFCNN-TIYKLQCVISQ-ARFVRDMVILLNVALVPVLFILFTYTRIF-VISYRS-SKEVRKKAAQTCLPHLIVLINF
LOR13                    VLPTA--MSAKEKLCR--FILNGVFCNN-TIYKLQCVISQ-ARFVRDMVILLNVALVPVLFILFTYTRIF-VISYRS-SKEVRKKAAQTCLPHLIVLINF
LOR14                    VLPTA--MSAKEKLCR--FILNGVFCNN-TIYKLQCVISQ-ARFVRDMVILLNVALVPVLFILFTYTRIF-VISYRS-SKEVRKKAAQTCLPHLIVLINF
LOR15                    VLPTA--MSAKEKLCR--FILNGVFCNN-TIYKLQCVISQ-ARFVRDMVILLNVALVPVLFILFTYTRIF-VISYRS-SKEVRKKAAQTCLPHLIVLINF
LOR16                    VLPTA--MSAKEKLCR--FILNGVFCNN-TIYKLQCVISQ-ARFVRDMVILLNVALVPVLFILFTYTRIF-VISYRS-SKEVRKKAAQTCLPHLIVLINF
LOR17                    VLPTA--MSAKEKLCR--FILNGVFCNN-TIYKLQCVISQ-ARFVRDMVILLNVALVPVLFILFTYTRIF-VISYRS-SKEVRKKAAQTCLPHLIVLINF
LOR18                    VLPTA--MSAKEKLCR--FILNGVFCNN-TIYKLQCVISQ-ARCVRDMVILLNVALVPVLFILFTYTRIF-VISYRS-SKEVRKKAAQTCLPHLIVLINF
LOR19                    VLPTA--MSAKEKLCR--FILDGVFCNN-TIYKLQCVISQ-ARFVRDMVILLNVALVPVLFILFTYTRIF-VISYRS-SKEVRKKAAQTCLPHLIVLINF
LOR20                    VLPTA--MSAKEKLCR--FILNGVFCNN-TIYKLQCVISQ-ARFVRDMVILLNVALVPVLFILFTYTRIF-VISYRS-SKEVRKKAAQTCLPHLIVLINF
LOR21                    VLPTA--MSAKEKLCR--FILNGVFCNN-TIYKLQCVISQ-ARFVRDMVILLNVALVPVLFMLFTYTRIF-VISYRS-SKEVRKKAAQTCLPHLIVLINF
LOR22                    VLPTA--MSAKERLCR--FILNGVFCNN-TIYKLQCVISQ-ARFVRDMVILLNVALVPVLFILFTYTRIF-VISYRS-SKEVRKKAAQTCLPHLIVLINF
LOR23                    VLPTA--MSAKEKLCR--FILNGVFCNN-TIYKLQCVISQ-ARFVRDMVILLNVALVPVLFILFSYTRIF-VISYRS-SKEVRKKAAQTCLPHLIVLINF
LOR24                    AVPAG--LSAHKNLCS--FVLKGFFCNN-TVYKLQCVISR-EQIIHDMVILLNVALVPVLFILFTYTRIF-VISYRS-SKEVRKKAAQTCLPHLIVLINF
LOR25                    AVPAG--LSAHKNLCS--FVLKGFFCNN-TVYKLQCVISR-EQIIHDMVILLNVALVPVLFILFTYTRIF-VISYRS-SKEVRKKAAQTCLPHLIVLINF
LOR26                    AVPAG--LSAHKNLCS--FVLKGFFCNN-TVYKLQCVISR-EQIIHDMVILLNVALVPVLFILFTYTRIF-VISYRS-SKEVRKKAAQTCLPHLIVLTNF
LOR27                    AGPIV--QNVNKKLCK--FIFKGIICNS-TVHKLHCVEPR-IEKIYGLIVFVNLLIIPVLFILFTYTRIF-IITYRS-CREVRRKAAQTCLPHL------
LOR28                    AGPIV--QNVNKKLCK--FIFKGIICNS-TVHKLHCVEPR-IEKIYGLIVFVNLLIIPVLFILFTYTRIF-IITYRS-CREVRRKAAQTCLPHL------
LOR29                    AGPIV--QNVNKKLCK--FIFKGIICNS-TVHKLHCVEPR-IEKIYGLIVFVNLLIIPVLFILFTYTRIF-IITYRS-CREVRRKAAQTCLPHL------
LOR31                    GTCVY--LASRLPLCG--NKIPKVFCANWPIVKLSC-ISTVINNLVGMLVSTTTVFLPLAFVLYTYARIF-LICRKR-SSEFKAKVIQSCLPHIITFVNY
LOR32                    GTCVY--LASRLPLCG--NKIPKVFCANWPIVKLSC-ISTVINNLVGMLVSTTTVFLPLAFVLYTYARIF-LICRKR-SSEFKAKVIQSCLPHIITFVNY
LOR33                    GTCVY--LASRLPLCG--NKMPRVFCANWPIVKLSC-ISTVINNLVGMLVSTTTVFLPLAFVLYTYARIF-LICRKR-SSEFKAKVIQSCLPHIITFVNY
LOR34                    GTCVY--LASRLPLCG--NKIPKVFCANWPIAKLSC-ISTVINNLVGMLVSTTTVFLPLAFVLYTYARIF-LICRKR-SSEFKAKVIQSCLPHIITFVNY
LOR35                    GTCVY--LASRLPLCG--NKIPKVFCANWPIVKLSC-ISTVINNLVGMLVSTTTVFLPLAFVLYTYARIF-LICRKR-SSEFKAKVIQSCLPHIITFVNY
LOR37                    GTLVG--LADSLRFCSS-NLIQHCYCDHMALVSLACDSTE-KNTAAGLAVIICFVGVDIPLIFFSYMKILSAVLRATAPDEDRWKAFHTCGTHLIIMMCF
LOR38                    GTLVG--LADSLRFCSS-NLIQHCYCDHMALVSLACDSTE-KNTAAGLAVIICFVGVDIPLVFFSYMKILSAVLRATAPDEDRWKAFHTCGTHLIIMMCF
LOR39                    GTLVG--LADSLRFCSS-NLIQHCYCDHMALVSLACDSTE-KNTAAGLAVIICFVGVDIPLILFSYMKILSAVLRATAPDEDRWKAFHTCGTHLIIMMCF
LOR40                    GTLVG--LADSLRFCSS-NLIQHCYCDHMALVSLACDSTE-KNTAAGLAVIICFVGVDIPLIFFSYMKILSAVLRTTAPDEDRWKAFHTCGTHLIIMMCF
LOR41                    GTLVG--LADSLRFCSS-NLIQHCYCDHMALVSLACDSTE-KNTAAGLAVIICFVGVDIPLIFFSYMKILSAVLRATAPDEDRWKAFHTCGTHLIIMMCF
LOR42                    GTLVG--LADSLRFCSS-NLIQHCYCDHMALVSLACDSTE-KDTAAGLAVIICFVGVDIPLIFFSYMKILSAVLRATAPDEDRWKAFHTCGTHLIIMMCF
LOR43                    VSLFS--LQTRLPRCK--SVIMNVFCDNPSLLKLTCG-NRAVNNIIGLFNTAVMQAVSVSLQAFSYVKIL-ITCLVSRRSEAKTKAINTCVAQLVILIIF
LOR44                    VSLFS--LQTRLPRCK--SVIMNVFCDNPSLLKLTCG-NRAVNNIIGLFNTAVMQAVSVSLQAFSYVKIL-ITCLVSRRSEAKTKAINTCVAQLVILIIF
LOR45                    VSLFS--LQTRLPRCK--SVIMNVFCDNPSLLKLTCG-NRAVNNIIGLFNTAVMQAVSVSLRAFSCVKIL-ITCLVSRRSEAKTKAINTCVAQLVILIIF
LOR46                    VSLFS--LQTRLPRCK--SVIMNVFCDNPSLLKLTCG-NRAVNNIIGLFNTAVMQAVSVSLRAFSYVKIL-ITCLVSRRSEAKTKAINTCVAQLVILIIF
LOR47                    VSLFS--LQTRLPRCK--SVIMNVFCDNPSLLKLTCG-NRAVNNIIGLFNTAVMQAVSVSLQAFSYVRIL-ITCLVSRRSEAKTKAINTCVAQLVILIIF
LOR48                    GILLG--LTIRLNRCR--SIIMNPYCDNASLFKLSCD-SVFINNVYGITFTVVLLTSSIGTMVLTYTNIT-VVCLTSKNKSLNSKALRTCSTHLVVYLIM
LOR49                    GILLG--LTIRLNRCR--SIIMNPYCDNASLFKLSCD-SVLINNVYGITFTVVLLTSSIGTMVLTYTNIT-VVCLTSKNKSLNSKALRTCSTHLVVYLIM
LOR50                    GILLG--LTIRLNRCR--SIIMNPYCDNASLFKLSCD-SVFINNVYGITFTVVLLTSSIGTMVLTYTNIT-VVCLTSKNKSLNSKALRTCSTHLVVYLIM
LOR51                    GILLG--LTIRLNRCR--TLIMNAHCDNAGLFKLSCE-NVFINNVYGLTFTVVLFTGSIGSMVLTYTKIT-VVCLTSNNRSLNSTAFKTCSTHLFVYLIM
LOR52                    GILIS--LTVRLNRCR--TMIMNPFCDNASLFKLSCE-SVFINNVYGLTFTVALYVVSIGSIVLTYTKIA-VVCLTSKNKSLNSKALKTCSTHLVVYLIM
LOR53                    GILIS--LSVRLNRCR--TMIMSPFCDNPSLFKLSCE-SVFINNVYGLTFTVVLLTASIGSMVFTYTKIT-VVCLTSKNKSLNSKALKTCSTHLVVYLIM
LOR54                    GILLG--LTIRLNRCR--TIIMNPYCDNASLFKLSCD-SVFINNVYGLTFTVVLLTSSIGTMVLTYTNIT-VVCLTSKNKSLNSKALRTCSTHLVVYLIM
LOR55                    GILLG--LTIRLNRCR--TIIMNPCCDNASLFKLSCD-SVFINNVYGLTFTVVLLTSSIGTMVLTYTNIT-VVCLTSKNKSLNSKALRTCSTHLVVYLIM
LOR56                    GILLG--LTIRLNRCR--TIIMNPYCDNASLFKLSCD-SVFINNVYGLTFTVVLLTSSIGTMVLTYTNIT-VVCLTSKNKSLNSKALRTCSTHLVVYLIM
LOR57                    GILLG--LTIRLNRCR--TIIMNPYCDNASLFKLSCD-SVFINNVYGLTFTVVLLTSSIGTMVLTYTNIT-VVCLTGKNKSLNSKALRTCSTHLVVYLIM
LOR58                    GILLG--LTVRLNRCR--TIIMNPYCDNASLFKLSCD-SVFINNVYGLTFTVVLLTSSIGTMVLTYTNIT-VVCLTSKNKSLNSKALRTCSTHLVVYLIM
```

```
LOR59                    GILLG--LTIRLNRCR--TIIMNPYCDNASLFKLSCD-SVFINNVYGLTFTVVLLTSSIGTMVLTYTNIT-VVCLTSKNKSLNSKALRTCSTHLVVYLIM
LOR60                    GILLG--LTIRLNRCR--TIIMNPYRDNASLFKLSCD-SVFINNVYGLTFTVVLLTSSIGTMVLTYTNIT-VVCLTSKNKSLNGKALRTCSTHLVVYLIM
LOR61                    GILLG--LTIRLNRCR--TMIMNPYCDNPSLFKLSCE-NVFINNVYGLTFTVVLLTSSIGSIVLTYTKIT-VVCLTSKNKSLNSKALRTCSTHLVIYLIM
LOR62                    GILLG--LTLRLNRCR--TLITNPFCDNASLFKLSCE-SVSINDVYGLTFTVVLFTGSIGSMVLTYTKIA-VVCLTSKNKSLNSKALKTCSTHLVVYLIM
LOR63                    GILLG--LTIRLNRCR--TLIMNAHCDNAGLFKLSCE-NVFINNVYGLTFTVVLFTGSIGSMVLTYTKIT-VVCLTSNNRSLNSTAFKTCSTHLFVYLIM
LOR64                    GILLG--LTIRLNRCR--TMIMNAHCDNAGLFKLSCE-NVFINNVYGLTFTVVLFTGSIGSMVLTYTKIT-VVCLTSNNRSLNSTAFKTCSTHLFVYLIM
LOR65                    GILLG--LTIRLNRCR--TMIMNAHCDNAGLFKLSCE-DVFINNVYGLTFTVVLFTGSIGSMVLTYTKIT-VVCLTSNNRSLNSTAFKTCSTHLFVYLIM
LOR66                    GILLG--LTIRLNRCR--TLITNPYCDNASLFKLSCE-SVFINNVYGLTFTVVLLSSSIGSIILTYFKIA-AACLTNKSNSLNSKALKTCSTHLCLYLIM
LOR67                    GILLG--LTIRLNRCR--TLITNPYCDNASLFKLSCE-SVFINNVYGLTFTVVLLSSSIGSIILTYFKIA-AACLTNKSNSLNSKALKTCSTHLCLYLIM
LOR68                    AILLS--LTIRLNRCR--TLITNPYCDNASLFKLSCE-SVFINNVYGLTFTVVLLSSSIGSIILTYFKIA-AACLTNKSNSLNSKALKTCSTHLCLYLIM
LOR69                    GILLG--LTIRLNRCR--TLITNPYCDNASLFKLSCE-SVFINNVYGLTFTVVLLSSSIGSIILTYFKIA-AACLTNKSNSLNSKALKTCSTHLCLYLIM
LOR70                    GILLG--LTIRLNRCR--TMIMNPYCDNPSLFKLSCE-NVFINNVYGLTFTVVLLTSSIGSIVLTYTKIT-VVCLTSKNKSLNSKALKTCSTHLCLYLIM
LOR71                    GILLG--LTIRLNRCR--TLITNPYCDNASLFKLSCE-SVFINNVYGLTFTVVLLSSSIGSIILTYFKIA-AACLTNKSNSLNNKALKTCSTHLCLYLIM
LOR72                    LIRVL--LLLNFPFDQLQSLQMTDFCSDIAMLLGPMSDIYDIAYTGFVFVSAGVAVTCSYIGVMVAARSAS-TDKVSARKARNTVLLHLMQLGLTLLSTM
LOR73                    LIRVL--LLLKFPFEELPSLQMTDVCSDIAMFLGPMSDIYDKAYTGFVFLSAGVAVTCSYVGVMVAARSAS-TDKASAHKARNTVLLHLMQLGLSLLSTM
LOR74                    LVQVL--LLLTFPFEELPSLQMTDICSYEAMFLVPMSDLYDKAYTGFVFLSAGVAVTCSYIGVMVAARSAS-TDKASAHKARNTLLLHLVQLGLILLSTM
LOR75                    LIQVI--LLLKFPFEKLPSLQMTDICAYVAMFLMPMSDIYDKAYTGFVFVSAGVTVTCSYIGVMVAARSAS-TDKASARKTRNTLLLHLVQLGLSLLSTM
LOR76                    LIRVL--LLLKFPFEELPSLQMTDVCSDIAMFLGPMSDIYDKAYTGFVFLSAGVAVTCSYVGVMVAARSAS-TDKASAHKARNTVLLHLVQLGLSLLSTM
LOR77                    LIQVI--LLLKFPFEKLPSLQMTDICAYVAMFLMPMSDIYDKAYTGFVFVSAGVTVTCSYIGVMVAARSAS-TDKASAHKARNTLLLHLVQLGLSLLSTM
LOR78                    LIRVL--LLLDFPFEDLGSLQMTEICSLIAVSIGPMSDIYDKAYTGFVFVSAGVTVTCSYIGVMVAATSAS-TDKASARKGRNTLLLHLVQLGLSLLSTM
LOR79                    LIRVL--LLLDFPFEDLGSLQMTEICSLIAVSIGPMSDIYDKAYTGFVFVSAGVTVTCSYIGVMVAATSAS-TDKASARKGRNTLLLHLVQLGLSLLSTM
LOR80                    LIRVL--LLLDFPFEDLDSLQMTEICSLIAVSIGPMSDIYDKAYTGFVFVSAGVTVTCSYIGVMVAATSAS-TDKASARKGRNTLLLHLVQLGLSLLSTM
LOR81                    LIRVL--LLLDFPFEDLDSLQMTEICSLIAVSIGPMSDIYDKAYTGFVFVSAGVTVTCSYIGVMVAATSAS-TDRASARKGRNTLLLHLVQLGLSLLSTM
LOR82                    LTAVF--MLLNFSFEDQQNLQMKDYCRREIMFLNPVSDLYDKVYTYILFCSAGVAVSFSYIGVMVAARSAT-TDKASAQKASKTLLLHLVQLGLILLSAL
LOR83                    MSDLF--ITLTT--QPLDFFHSQVFCLRQTVFPSPLIIKKRDITYSVFLVIVWGTILYTYFRILFTAKTASKD----AKKARNTILLHVFQLLLCMATYV
LOR84                    MSDLF--ITLTT--RPLDFFHSQVFCLRQTVFPSPLIIKKRDITYSVFLVIVWGTILYTYFRILFTAKTASKD----AKKARNTILLHVFQLLLCMATYV
LOR85                    MSDLF--ITLTT--QPLDFFHSQVFCLRQTVFPSPLIIKKRDITYSVFLVIVWGTILYTYFRILFAAKTASKD----AKKARNTIILHVFQLLLCMATYV
LOR86                    MSDLF--ITLTT--QPLDFFHSQVFCLRRNVFPSPLIPKKRDITYSVFLVIVWGTILYTYFRILFAAKTASKD----AKKARNTIILHVFQLLLCMATYV
LOR87                    LSDLF--ITLAT--QPLDFFHSRVFCLRKTVFPSPLIIKKRDITYSVFLVIVWGTILYTYFRILFAAKTASKD----AKKARNTIILHVFQLLLCMATYV
LOR88                    LSDLF--ITLAT--QPLDFFHSQMSCIRRNVFPSPLIPKKRDITYSVFLGIVWCTIFYTYFRILFAAKTASKD----AKKARNTIILHVFQLLLCMATYV
LOR89                    MSDLF--ITLTT--QPLDFFHSQVFCLRQTVFPGPLIIKKRDITYSVFLVIVWGTILYTYFRILFTAKTASKD----AKKARNTILLHVFQLLLCMATYV
LOR90                    MSDLF--ITLTT--QPLDFFHSQVFCLRQTVFPSPLIIKKRDITYSVFLVIVWGTILYTYFRILFTAKTASKD----AKKARNTILLHVFQLLLCMATYV
LOR91                    LPDLF--ITLAT--QPLDFFYTRVFCLRETAFSNPHIIKKRDITYIVYLVIVWFVIFYTYFRILFTAKTASKD----AKKARNTIILNGFQLLLCMAIYA
LOR92                    ISDLF--ITFAT--EPLDFFHSQVFCLRETAFPSPLIVEKRVITYSVFLVIVWCTIFYTYFRILFTAKTASKD----AKKARNTILLNGFQLLLCMAIYA
LOR93                    IVILS--VFCAS--ASKSLYTQYKICPMEIFVSLRWQNNIKTAIYQLYFLIMSITIILSYVKIMKVAKAASGENKKSTRKGLRTVILHGFQVFLCLVQLW
LOR94                    IVILS--VFCAS--ASKSLYTQYKICSMEIFVSLRWQNNIKTAIYQLYFLIMSITIILSYVKIMKVAKAASGENKKSTRKGLRTVILHGFQVFLCLVQLW
LOR95                    IVILS--VFCAS--ASKSLYTQYKICSMEIFVSLRWQNNIKTAIYQLYFLIMSITVILSYVKIMKVAKAASGENKKSTRKGLRTVILHGFQVFLCLVQLW
LOR96                    IVILS--VFCAS--ASKSLYTQYKICSMEIFVSLRWQNNIKTAIYQLYFLIMSITIILSYVKIMKVAKAASGENKKSTRKGLRTVILHGFQVFLCLVQLW
LOR97                    IVILS--VFCAS--ASRSLYTQYKICSMEIFVSLRWQNNIKTAIYQLYFLIMSITIILSYVKIMKVAKAASGENKKSTRKGLRTVILHAFQVFLCLIQLW
LOR98                    IVILS--VFCAS--ASKSLYTQYKICSMEIFVSLRWQNNIKTAIYQLYFLIMSITIILSYVKIMKVAKAASGENKKSTRKGLRTVILHAFQVFLCLVQLW
LOR99                    IVILS--TFFAS--ASFSLYKQPRLCEMELLMLYRWQDHVRSAVHQFYFLIMAIIILFSYVKIMKVAKAASGEDKKSSWKGLRTVILHGLQLLLCLIQLW
LOR100                   IVILS--TAFAT--ASLSFYTQPMLCSVETFMLYRWQDHVRSAVHQFYFLIMAIIILFSYVKIMKVAKAASGEDKKSSWKGLRTVILHGFLLLLCLIQLW
LOR101                   IVVLS--TFFAS--ASLSLYKQYSSCSVEILILHRWQGHVRSAVHQFYFLIMVIIILFSYVKIMKVAKAASGEDKKSSRKGLRTVILHGFQLLLCLIQLW
LOR102                   IVVLS--TFFAS--ASFSLYKQYSSCSVEILILHRWQGHVRSAVHQLYFLIMVIIILFSYVKIMKVAKAASGEDKKSSWKGLRTVILHGFQLLLCLIQLW
LOR103                   IVVLS--TFFAS--ASFSLYKQYSSCSVEILILHRWQGHVRSAVHQFYFLIMVIIILFSYVKIMKVAKAASGEDKKSSWKGLRTVILHGFQLLLCLIQLW
LOR104                   IVVLS--TFFAS--ASFSLYKQYSSCSVEILILHRWQGHVRSAVHQFYFLIMVIIILFSYVKIMKVAKAASGEDKKSSWKGLRTVILHGFQLLLCLIQLW
LOR105                   IVVLS--TFFAS--ASFSLYKQYSSCSVEIFMLHRWQDHVRSAVQQFYFLIMVIIIVFAYVKIMKVAKAASGEDKKSSWKGLRTVILHGFQLLLCLIQLW
LOR106                   IVVLS--TFFAS--ASFSLYKQYRLCAIKLFMLYRWQDHVISAVQEFYFLVMVIIILFSYVKIMKVAKAASGEDKKSSWKGLRTVILHGFQPLLCLIQLW
LOR107                   IVILS--TFFAS--ASLNLYKQHKLCTVEMLILYRWQGHVRSAVHEFYFLIMVIIILFSYVKIMKVAKAASGEDKKSLWKGLRTVILHGFQLLLCLIQMW
LOR108                   IVVLS--TFFAS--ASFSLYKQYRLCAIQLFMLYRWQDHVISAVQEFYFLIMAIIILFSYVKIMKVAKAASGEDKKSSWKGLRTVILHGFQLLLCLIQLW
LOR109                   IVVLS--TFFAS--ASFSLYKQYKICAIKIFMLYRWQDHVISAVQEFYFLIMVIIILFSYVKIMKVAKAASGEDKKSSWKGLRTVILHGFQLLLCLIQLW
LOR110                   IVILS--TFFAS--ASFSLYKQPMICAIKIFMLYRWQDHVISAVQEFYFLIMVIIILFSYVKIMKVAKAASGEDKKSSWKGLRTVILHGFQLLLCLIQLW
LOR111                   IVILS--TFFAS--ASFSLYKQPMICAIKIFMLYRWQDHVISAVQEFYFLIMVIIILFSYVKIMKVAKAASGEDKKSSWKGLKTVILHGFQLLLCLIQLW
Fugu_OR123-1             FFNTI--TTSRYRLCG--SHIQRIYCVTYMIAKLSCSAS--IANAVLAYINYIFYFCHFSAVVWSYVYLI-QKCLG--SKESRTKFMQTCLPHLLCLLTM
Fugu_OR6765-1            IVITV--STSTLRLCG--SQISKLYCVTYVIRKLQCSSS--ISTVIFPAFIITFYSCHVLLVIYSYFYIM-KTCLT--SKEDRRKFIQTCLPHVSSLIVV
Fugu_OR4133-1            LITIT--LTSTLRLCG--SQISRIYCVNYVIRRLECTPS--ITTVFSPAFTITFYFCHFLYVIYSYFYIM-KTCLT--RKEERMKFMQTCLPHLSSLIIA
Fugu_OR3630-2            AVPTI--MNANRKLCN--FIFKGIICNS-TVFNLHCQRSE-TFNIYGLMVLVNLLIVPVMFILFSYARIL-LISYQS-SKEVRKKAAQTCLPHLMILINF
Fugu_OR8617-1            AVPTI--RNANRKLCN--FIFKGIICNS-TVFNLHCQRSE-TFNIYGLMVLVNLVIVPVMFILFSYARIL-LISYQS-SKEVRKKAAQTCLPHLMILINF
Fugu_OR5510-1            AVPTI--MNANRKLCN--FIFKGIICNS-TMFNLHCQRSE-TFNIYGLMVLVNLLIVPVMFILFSYARIL-LISYQS-SKEVRKKAAQTCLPHLMILINF
Fugu_OR117-1             AACVY--LSSMLPLCG--NAIPKVFCANWPVVKLSC-VPTWLNNLIGMLVSVTTVFLPLAFVLYTYGRIF-FICRKC-SSDFKSKVVHSCLPHIITFVNY
Fugu_OR2346-5            AVGLY--LSVRLPLCG--NEIKRVFCGNWNVVKLSC-VSTIVNNIYGMFVTILTIIFPLLYVLYTYIQIV-ATCWKG-SAGFNRRVFQSCVPHIISFVIN
Fugu_OR5287-2            AALVG--LASSLRFCGS-NTIQHCYCDHMALVSLACGSTE-RNSAIGLVVIICFVGLDIPLILFSYIKILSVVSRASVGSEDRWKAFHTCGTHLMVMMCF
Fugu_OR142-1             LLLFV--LQMRLPHCK--SVIMNVFCDNPSLLKLTCA-NTTINNVVGLFNTAVIQAVSLSVQAFSYVKIL-IACMVSRRSEAKMKAINTCVAQLMILAIF
Fugu_OR1026-2            GILLG--LTIRLNRCR--TLIANTFCDNASLFKLSCD-SVVINNIYGLTFTVVLFIASIGSIVVTYTKIT-IVCVTSNNKSVNSKALKTCSTHLVMYLIM
Fugu_OR8298-1            GILLG--LTIRLNRCR--TLITNPYCDNASLFKLSCD-SVVINNIYGLTFTVVLFIASIGSIVVTYTKIT-IVCVTSNNKSVNGKALKTCSTHLLMYLIM
Fugu_OR6030-1            LIQVS--MVD---YPEMESLQMEVYCSSITLFVGPLSDTYDKAYTCLLFVSAGLAIAFSYIGVTVAARLAS-TDKTSSQKARNTLLLHLVQLGLSLSSTV
Fugu_OR59-1              LIRVS--LVN---YPELKGLQMEEFCSSITLFVGPLSDTYDRAYTCLLFVSAGVAISFSYIGVTVAVRLAS-TDKTSSQKARNTLLLHLVQLGLSLSSTV
Fugu_OR4208-3            LPDLF--VTLAT--QPLDFFYSQVFCLRETAFPNPHIIRKRDITYVVYLVIVWLIIFYTYFRIMFTAKTASKD----ARKARNTIILHGFQVLLCMATYA
Fugu_OR4208-2            TSDLV--ITLAT--EHLTIVHSQVFCLRHTAFPLDVITTKRDVTYALFLVIIWITIFYTYFQILLMAKTAGKD----TVKARNTIILHSFQLLLCMASYL
Fugu_OR6818-1            AVVLS--MLFAS--ASYNFYTQVKICSVEILIIHQWQSHLRSAISQFYFLIMCVVILLSYIKVMKVAKAASGENKKSTWKGLRTVILHAFQLLLSLIQLW
Fugu_OR7903-1            FIILS--TFIAA--ASSSVYTQHKRCSMEMFVSIPWQNHFKFAVYQFYFFIMSITITFSYVKIMKVAKAASGENKKSTWKGLSTVILHAFQLLLCLIQLW
Pufferfish_OR8981-2      LVIII--STSTIRLCS--SKISRVYCVTYAKRRLECSVS--ITTVLFPAFMITFYLCLFLLVIYSYFHIM-KTCLT--SKDDRIKFLQTCLPHLMSFIVM
Pufferfish_SCAF8981      IITII--ITSTLRLCG--SEISRIYCVNYAIRRLECSAS--ITATVLPAFMIAIYFCVFLLVIYSYFHIM-KTCLS--SKDDRIKFLQTCLPHLMSFIVV
Pufferfish_OR15134-1     VVPTI--TNANKKLCH--FVFKGIICNS-TIYDLLCQRSQ-ILNIYGLIVLVNLVILPVIFILFSYIRIL-LVSYQS-SREVRRKAAQTCLPHLLILINF
Pufferfish_OR14339-2     TPFVY--LSSILPLCG--NAIPKVFCANWHVVKLSC-VSTGLNNLLGMLVTTPVVFLPFVFVLYTYGRIF-LICRNR-SSDFKRKVVQSCLPHIITFVIY
Pufferfish_OR14328-1     TPFVY--LSSTLPLCG--NAIPKVFCANWLVVKLSC-VPTGLSDLFGMLVTTPVVFLPFVFVLYTYGRIF-LICRKR-SSDFKRKVVQSCLPHIITFVNY
Pufferfish_OR14677-8     ATLVG--LASSLTFCGS-NTIQHCYCDHMALVSLACDSTE-KNSAAGLAVIICFVGMDIPLILFSYAKILSVVSRASVASEDRWKAFHTCGTHLMVMMCF
Pufferfish_OR14536-2     LIRIS--LVD---FSELENLQMKEFCSGLTVFVGPLSDTYDRASTCLLFVSAGAAIIFSYIGVTVAAKSAS-TDKTTSQKARHTLLLHLVQLGLSLSSTL
Pufferfish_OR14536-1     LIRVS--LVD---FSELENLQMKEFCSSLTVFVGPLSDTYDRASTCLLFVSAGATILFSYIGVTVAAKSAS-TNKTTSQKARHTLLLHLVQLGLSLSSTM
Pufferfish_OR10960-2     LPDLL--VTLAT--QPLGFFRSRVFCLRETAFPNPHIIRKRDVTYIAYLLAVWLVIFYTYFRILFTATSASGD----SRKARRTIVLHGVQVLLCMATYA
Pufferfish_OR10960-3     ASDLL--LTLAT--ERLAIVRSQVFCLRHTAFPHPVIVKKRDATYSLFLVMVWITMFYAYFRILVAAKTASKD----AKKARNTIVLHSFQLLLCMAAYL
Pufferfish_OR12434-1     FIILS--TFFAA--APSSVYKQYKLCSMEMFVAVQWQKQLKFSVYQSYFVIMLITITLSYVKIMKIAKAASEETKTSTFKGLKTVILHSFQLLLCLIQLW
Medaka_ORUn.33           ATLVA--LAGSLRFCGS-NVIQHCYCDHMALVSLACDSTD-RSWSAGVAVIVCFVGVDIPIIFFSYVKILRVVLRAAR--EDRSKAFHTCSTHLMVIMCF
Medaka_ORUn.2            TLLVA--LAGARSFCS--DVIHHCVCEHMALVELACGSTA-LNSLAGLMAIFLIPLLDFLIISASYIVIFSTVLSSAR---SGGKALHTCVTHIMVLSLS
Medaka_OR21.18           MTVVS--LTSTLKLCG--SHIDKLYCENWSLVKLACGST--KANDVTGLIFVVFYICHVLWIVFSYVQLI-RSALK--SREGKGRFVQTCVPHFLCVLNV
Medaka_OR21.19           GLNVF--LTSRLKLCS--PHLSRLFCVNWSIVRLACPQEDTTANNIVANMTIIIYVCHGLFIVWSYMYLV-KTCMN--SIENRAKFMQTCVPHLVSLITF
Medaka_OR21.20           AVNTC--LTSSIKLCS--RHLSRLFCVNWSIVRLACPQEEMTANNVVANITIIIYVCHGLFIVWSYMYLV-KTCMN--SIENRAKFMQTCVPHLVSLITF
Medaka_OR13.14           TINLA--IFYSA--ASTTFLTQRGICYGERFILFTWQGQFRSAVSQLYFFIMFTIIVFSYIKIMKVAKTASGENKKSTWKGLRTVVLHGFQLFLSLIQLW
Medaka_OR13.12           IVILS--IFFAA--VSPTYFTQSTICSAEMFILFSWQRHLRSAISQFYFLIMVIIIVISYVKILKVAKTASGENKKSTRKGLRTVTLHGFQLQLGLIQLW
Medaka_ORUn.23           LTRVL--MFLKFPFEKIKPT---DVCANVSVMLAPLTADYDRAYTCVVFVSAGVAVTSSYVGVIVAARSAS-TDKALARKAQHTLLLHLVQLGLSLSSTI
Medaka_ORUn.21           LTRVL--MFLKFPFEKMEVY---DLCSNIALVLGALTAEYDRAYTCVVFVSAGVAVTSSYVGVIVAARSAS-TDKALARKARHTLLLHLVQLGLSLSSTI
Medaka_OR14.15           LTRVL--MFLKFPFEKMEVY---DLCSNIALVLGVLTAEYDRAYTCVVFVSAGVAVTSSYVGVIVAARSAS-TDKALARKARHTLLLHLVQLGLSLSSTI
Medaka_OR14.13           SILLG--LTIRLNRCR--TLISNSYCDNASLFKLSCE-SVVINNIYGLTFTVVLLTSSIGCMVLTYTSIS-VVCLTSKNKSLNSKALKTCSTHLVAYFLM
Medaka_OR14.4            AVLLG--LTIRLNRCR--TLIENPFCDNASLFKLSCE-SSVINNIYGLTFTAVLLTSSIGSIVLTYSKIT-AVCLTSKSKSLNNKALQTCSTHLVVYLIM
Medaka_OR14.6            AVLLG--LTIRLNRCR--TLIENPFCDNASLFKLSCE-SSVINNIYGLAFTVVLLTSSIGSIVLTYSKIT-AVCLTSKSKSLNNKALQTCSTHLVVYLIM
Medaka_OR13.4            GVLFI--LLLRLNNCR--SEMSNPYCDNPNLLSLVCG-DTTINNIYGLFISALSQAVANGIVFYTYLRVL-IACFRSKQSDTRAKALQTCATHLVVFLLL
Medaka_OR13.5            FTLFM--LLARFKFCR--TNIADWYCNNPSLLKLVCS-DITLNNYYGLFTIILDMGAPLILILYTYAQIL-FTCVKTNNTNGRKKAIQTCSTHLVVFLVL
Medaka_OR13.6            LTLFM--LLFRFKICR--ADVVDLFCNYPSVLKLVCG-DITLNNNYGLFTLIVIMGVPLMIILYTYVQIL-VTCMMTNNPDGRRKALQTCCTHVLVFLVL
Medaka_OR13.7            LLLFS--LQARLPRCT--ALIRNVFCDNPSLLKLTCV-NTAVQNITGLFNTAVIQAISISIQVYSYVKIL-ITCLATRTSEAKTKAVHTCVAQLVILVIF
Medaka_ORUn.1            AVGLV--LVLQLPLCG--NRISKMFCTNWELVKLACQPA--SYDDVYSFLITVLHLSQAALIVVSYAQLI-RTASR--SRSDRRKFLQTCVPHLAALLVF
Medaka_OR21.2            CIVVG--GNVNSELCS--FTLNGIFCNN-SLNYLFCAGSK-ALLTFGLVTLFNIAILPMLFIIFTYIKIL-LVVNKG-CREVRRKAAQTCLPHLLVLINY
Medaka_OR21.3            VVPAI--QNSKKELCS--FKLTGIFCNS-SLNDLFCAGLG-EPMISGFIILFNVVILPMLFIIFTYTKIL-IVVYKS-SRKVRGKAAQTCLPHLLVLINY
Stickleback_OR1.1        ATLVS--LAGSLRFCGS-DVIRHCYCDHMALVNLACGSTE-RNMVAGLAVIVCFVGVDIPLIFFSYVKILDAALRAATASEDRRKAFHTCGTHLMVMMCF
Stickleback_OR16.3       STNMI--LTSRLHLCS--PYIDRLYCMNWNIVKLACFPADTIFNNTLANVTNIFYLCHGVLMVWSYLYVI-KICVN--SLENRAKFMQTCVPHLACLLIF
Stickleback_OR1.12       IVVLS--IFFAS--VSHNFYSKGRVCSVEMFILYKWQGHLRSAVSQFYFFIMFIAIVFCYIKIMKVAKAASGEDKKSTRKGLKTVIFHAFQLSLCLIQLW
Stickleback_OR1.13       FVLLS--TFFAS--ASHSFYSQVRECAVEMFIFHKWQGHLRSAMSQFYFFIMFIAIVFCYIKIMKVAKAASGEDKKSTGKGLKTVILHAFQLSLCLFQLW
Stickleback_ORUn.9       LTQVV--LLLDFPFQDLQSLRMDDFCSKGRVMLGAASEVYDRGYTCFLFVSTSVAVASSYVAVTMAARSAS-SGGASARRARDTLLLHLVQVGLSLSSTV
Stickleback_OR7.1        FSQFV--LFVRL---ENT-ITVMRICS-QDIFQLQIYLAMNKGFTITIFVLVSMISIYTYVVVIVAVKSAS-STVEKSSKPHKTMLLHLIQLCLCVTSTL
Stickleback_ORUn.6       GILLG--LTLRLNRCR--TLITNPYCDNASLFKLSCE-SVLINNIYGLTFTVVLLTGSIGSMVITYTKIT-VVCLTSNSKSLNSKALKTCSTHLVVFLIV
Stickleback_ORUn.8       GILLG--LTLRLNRCR--TLITNPYCDNASLFKLSCE-SVLINNIYGLTFTVVLFTGSIGSMVITYTKIT-VVCLTSNSKSLNSKALKTCSTHLVVYLIM
Stickleback_OR1.18       TSLFS--LQTRLPRCK--SVIMNVFCDNPSLLKLTCG-NTTVNNVLGLFTTAAMQAVSVSIQVFSYVKIL-ITCMVTRRSEAKTKAVNTCVAQLLILIIF
Stickleback_OR16.10      ATCVY--LSSRLPLCG--NKIPKVFCANWPVVKLSC-VSTVINNLVGMFVSISTVFLPLAFVMFTYIRII-LNCRKR-SSEFKSKVIQNCLPHIVTFVNY
Stickleback_OR16.32      LISTV--TTATSRLCG--SHIPKIFCVNWLISRMACSAS--VGTIVIPAFNYTFYFGHCVFVCWSYIYMI-STCRK--SKENMSKFMQTCVPHLFSLIIV
Stickleback_OR16.1       ATCVY--LSFRLPLCG--NKIPKVFCANWPVVKLSC-VSTVINNLVGMFLSISTAFLPLAFVMFTYIRII-LICRKR-SSEFKRKVIQNCLPHIVTFVIY
Stickleback_OR16.5       ATCVY--LSCRLPLCG--NKVPKVFCANWPVVKLSC-VSTVINNLVGMVALITTVFLPLAFVMFTYIRII-LICRKR-SSEFKSKVVQNCLPHIVTFVIY
Stickleback_ORUn.1       AVPVT--LSANTKLCT--FTLRGIFCNN-SIYKLHCVSSR-VLSLYGVIVLCNVNLFPVLFIVFTYTKIF-VITYRS-CGEVRMKVAQTCLPHLLVLINF
Stickleback_ORUn.17      SVLIL--LNAKRKICT--FHLKGILCNS-SVQTLQCARSL-IVNIYG-LILVTFLLFPVVFIFFTYSKIL-VVSYR--CRGVRRRAAQTCLPHLLVLINF
Zebrafish_OR126-7        SLLIV--LLSRLVLCG--STIEKLYCEIWAVAKLSCFSN--TVNNVFGLTVILVYFGHGVLIYCSYFQLI-RKCVK--STEGRRKFMQTCVPHLLSLINV
Zebrafish_or126-3        AVLIL--LIARLTLCG--FRIEKLYCEIWGVAKLSCFST--TLNNVFGYIVIIAYVGHAILIFCSYIYLI-RKCQK--SIESRHKFMQTCVPHMISLLNV
Zebrafish_OR123-1        ILLIL--MTLRFPICI--KKISKLYCETWVLERLVCGVD--TAQFVFNGILTCAVNALVWFVFLSYINIL-VACKH--SVQNHKKFISTCLPHLIAFLNY
Zebrafish_OR125-1        VASIL--LSNLRAFCK--YHIDKLYCDNWSIVKLSCVSS--FVNNVFGYGIAVIFFSFVVLIIFSYIKLV-AACKA--SLENRKKFWQTCLPHIFSLINF
Zebrafish_OR127-1        ALVIF--FSAKLPLCG--TDVTRLYCSNWSVVRLSCGTSTLSNNILGFFVTTATVFLPAFFILYTYIRIL-IICQKS-SKEFKGKALQTCLPHIVSFVNY
Zebrafish_OR128-10       GIAAI--LNARLTMCG--NKLWKVYCHNWEIVKLSC-ASTIINNVFGFFIVTTTVIMPLSFILYSYVKIL-IICRKS-SPEFRRKAYQTCIPHIVILLNF
Zebrafish_OR116-2        ILLFA--LQSKYSRCR--SDILNVFCDNPSLLKLTCS-NTIVSNIIGLFNTAMIQIISVSIQAFSYVKIL-ITCLATSKSDAKNKAINTCVSQLVIFIMF
Zebrafish_zor6a          STLVA--LASPLSFCGS-NVIYHCYCDHMALVSLACDSIA-QNQTMGLIVIICFVGIDTSVIFFSYVKILHVVL-GTAAGEDRWKAFHTCGTHLMVMICF
Zebrafish_OR117-1        TVLFS--LQARLVKCK--SFITNVYCDNPSLVLLSCGGDLSVNNIYGLFLTAFIQITSVFIQMFSYGCIV-ITCVKQTHADSRVKALNTCVAQFTTFILF
Zebrafish_OR115-15       SILIG--LTLRLSRCR--TFISNPFCDNASLFKLSCE-DVTINNLYGLIYTVLLFGSSMGSIAVTYIKIT-AVCLVTKSKMLNSRALKTCSTHLSLYLIM
Zebrafish_OR133-7        LSEMI--IFYSF---DNTNISMNLFCSRTTLFRLQIYKKFDIAFTCIFFVLVSFVIIFTYASISAVAKTAA-CDKLSAKKANKTVLLHLIQLGLGASSIL
Zebrafish_OR132-1        VIDII--IALIN---NPSYLAEIAFCTLEKLFIAKWQIDKSQGFDVLYFVSVAVTIIFTYIMIMITARSVS-ADKESAKKALKTVMLHLIQLGLCLTSFL
Zebrafish_OR134-1        ITDLF--VTLAT--EPLSFFQTSVFCLRQNVFKDPVLLYKRQAFDGIYFSLVFLTLLCTYLKIMFTAHSHSTENTS-AKRATNTILLHGLQLIMCLLSYI
Zebrafish_OR131-2        LFIFI--VSFTY--TPPNAMNSYVVCSNDVMFQVKWLAEMRALSQQLLFVIMLCIVGSTYIKIMVAAKSASAENKKSTYKGLRTVILHGLQLILGMMQLI
```

```
Zebrafish_OR131-1        LFVFI--GSFAY--TPPNAMNSYVVCSIEVMLQVKWLADTRATSQQLLFVIMLCIVGSTYIKIMIAARSASATNKKSTYKGLRTVILHGIQLILGMMQFI
Goldfish_OR3             CVVIV--LTSRLILCG--SSIEKLYCENWSVVKLSCFST--TVNNVIGYVIIIIYFGHAVLIFCSYIHLI-GKCRK--SKEGRNKFIQTCVPHLLALLNV
Goldfish_OR2             LMMAI--RAYPLPYCSGNT-IIHCYCDHISITSLACTN-RALYGVPAFAIAMVVLLGPLAFIIFSYCAII-VAVLHISSTQGRLKSFSTCSPQLIIIALY
Rainbow trout OR500-1    LIAVS--LAVRLPLCG--SRIDKIFCDIPSILKHACSPI--TINQNLNKFVIVVHVLQILFIVFSYCQIV-RTCVK--SAKGRIKFTQTCVPHLITIFIF
Cutthroat trout OR600-1  GILLG--LTIRLSRCR--SNIFNPYCDNASLFKLSCE-NVFINNIYGLIFTVVLFVSSIGSISLTYLKIA-IVCLSSKNSSLNSKALTTCSTHLLVYLIV
Brown trout OR500-1      LIAVS--LTARLSLCG--CRIDKIFCDNPSILKHACLPI--TINQNLNKCLIVVHVLQIFFIVFSYSQIV-RTCVK--SAKGRIKFTQTCVAHLITIFMF
Atlantic salmon SORB     GILLG--LTIRLSRCR--STILNPFCDNASLFKLSCE-SVFINNMYGLTFTIVLFISSIGSIALTYMKIA-AECFISKNKAMNSKALKTCSTHLTVYLIM
Medaka_mfOR2             TVAISGKVIINQRPCS--FSLEGIFCDS-ALQRLLCIKLE-ELAVLDMIILFNITVLPSLFILFTYICIL-KISYRS-SKEVRSKAAQTCLPHMLVLVNF
Medaka_mfOR1             TVAISGKVIINQRPCS--FSLEGIFCDS-ALQLLLCKKLE-ELAVLDTLILVNVVVLPSLFILFTYIRIL-KISYRS-SKEVRSKAAQTCLPHLLVLVNF
Medaka_OR_Y3             GILLG--LTIRLSRCR--TLIENPFCDNASLFKLSCE-SVVINNIYGLTFTVVLFSSSIGSTVLTYTSIS-VVCLTSKNKSLNRKALKTCSTHLFVYMVM
Medaka_ORY1              AVLLG--LTIRLNRCR--TLIENPFCDNASLFKLSCE-SSVINNIYGLTFTAVLLTSSIGSIVLTYSKIT-AVCLTSKSKSLNNKALQTCSTHLVVYLIM
Frog_OR-52D1             AILVL--LTIRLPLCG--SVIQKIYCDNISVVKLSC-TDTTANNVFGLLITAAIIGLMPILTLMSYAQIL-RVCMKA-SKAFRAKALQTCTPHLVTLTYF
Frog_OR-52E8             TIHVI--LTIRLPLCG--SVIQKIYCDNWSVVRLSC-IDTTLNNVFGLLMSS-ITGLLPGGVLISYVKIL-RVCMKS-SKDVRAKALQTCTPHIVSLLYF
Frog_OR-52A1             CPLVI--LTSMVQFCRL-NIILNFTCENMALLSLGCGDTT-KPQIAGLIVRVLVTVLDVSLLMISYSTILYTAMKSATG-KSQHKALNTCGTHLLVAMVV
Frog_OR-52P1             CPLIV--LISMVQFCRS-NVILNFACENMALLSLGCGDTT-KPQIAGLIVRIIATVLDGSLLLISYSTILYTAMKTATG-KSRHKALNTCGTHLLVAMVV
Frog_OR-52A5             IADCI--ILSTS--VPSSFFSLHVICARSAFMNTSVQTLIRNLTHILSFTLVGLIIIFTYIKIMMVALRVDSGKES-AIKASRTVMLHAIQLFLCLMAFS
Frog_OR-2M5              VADFI--TFSLS--ANKRYFSLHVICSRESLTVNPTQNTIRSNVLVITVSLVALIILYTYIKVMLVARKVGSGTSS-AIKAGKTVLLHGVQLLLSLMSLI
Frog_OR-2M2              SIVAV--IVGQLPYCGPNR-VKNCFCVNSAVAVLACVD-VTLARRTVFILAMCVLLLPLAVIILSYVLII-RVIHS-TNTENSWKAFYTCSTHLMVIGLY
zebrafish_OR10.1         LYAVF--SILTLSFCGSNE-MNSYFCDYTPVLVLACSD-ITQQLNYATAATILFIGVASIFIFFTYIGIL-RAVFRMKSSQSRFKALATCTEHLVLVCLY
zebrafish_OR10.14        MTATI--FISRLSFCKVIVTVNSFYCDHGPIYRSACSN-NFPSSVIGSLYPIVILGLPVFFIIFSYTCIA-VTLFRVTTPQDRQRATKTCTAHLILVAVF
zebrafish_OR15.39        IFPVI--FASRLPYCSSRA-VLSCCCEHGPVYRLACTD-TTYNRQLGTVKTMITLLGPLFFIVFTYVIVV-IAVMRIASVTQRWKAFHTCLTHMMLVMLY
Medaka_ORUn.6            AYSVA--LITKLSFCSSVK-VFSYFCDYAPVYRLACND-NSKQWFAGTFFSYLNLLGPFTFIVLSYVSIL-VTVFRMKTSDNRRKTLATCAEHIILVAIF
Medaka_ORUn.28           STAAG--LLTRLSFCNSVV-INSYFCDHGQIFGIACND-YSPSYIFSDVLIAIILWIPLIIVISSYVCIC-FALFKVSTIQEGVKAFKTCTAHLSLVAIY
Medaka_ORUn.27           LISVG--LLTRLSFCKSVV-INSYFCDHGQLYRLACND-NFPNDVVSFFYPVVIFWLPLVFILSSYLYIG-YTLVKVATLQQGIKAFKTCLAHLSLVVVY
Stickleback_OR7.3        AHDTA--IMTRLSFCNSVR-VNSYFCDYAPVFRLACND-YTLHWSIASTSSMVNLMVPFTVIILSYLSIL-ATVFRMKSVSNRMKALATCIEHLILVAIF
Stickleback_OR7.4        AFSTG--IMTQLSFCKSVR-VFSYFCDYAPVFRLACND-YAMQWSAASFLSFMILGGPFVFIILSYVTIL-VTVFRMKSLDSRVKALATCTEHVILVAVF
Stickleback_OR7.8        LIAVG--LITRLSFCRSVA-INSYFCDHGQIYRLACND-NFPNYVLSCLFPVLIFWLPFIFILLSYLYIG-CTLAKVATVQEGLKAFRTCTAHLSLVAIY
Fugu_ORUn.83             LIAVG--LITRLSFCKSVV-IHSYFCDHGQIYRLACND-HFPSYVVSCLYPVFIFWLPLLFILLSYLYIS-CTLAKVATVQQGLKAFRTCIGHLLLVAIY
Fugu_ORUn.20             AFSTG--IMTWLSFCKSVT-VRSYFCDYAPTFRLACND-YTLHWAVSSTASVVNLVAPFTFILLTYAAIL-VTLFLMKSVNSRMKALGTCVEHLVLVAIF
Pufferfish_OR7.1         LIAVG--LITRLSFCKSVV-VHSYFCDHGQIYRLACND-HFPNYVVSCLYPVFIFWLPLLFILLSYLYIS-CTLAKVANFQRGLKALKTCTGHLLLVAIY
Pufferfish_OR7.3         AFSAS--IMTWLSFCKSVE-VYSYFCDYAPTFRLACND-NTLHWAVSSTASVLNLVAPFVFIVLTYTAIL-LTLFRMKSVNSRMKALSTCVEHLVLVAVF
zebrafish_OR10.29        TVATL--DTQQLPYCGPNL-ILQCYCDHISITNLACAE-NSKQLLVALCMALLVLLLPLVCIIYSYCHII-ASVMRLSSSQSRWKSFATCSTQLCIITLF
zebrafish_OR10.31        SISLS--LTLKLPFCGPNM-IAHCFCDSLSMNQLACAD-ASFYNLIAFGVAMFVLLVPLSFIIFSYASIL-VSVLQIANAKGRLKALSTCATQLTIISIY
zebrafish_OR15.58        VGVTL--LSLSVKYCGSNV-IPHCYCDHVSISKLACGD-AATMKVTSTAIAMFVLWGPLSFILFSYVSII-ISVSKISKSEGRYKTFSTCTPQLLIICLY
zebrafish_OR15.57        TFMVY--NTFDEPYCGSNI-ITQNYCDHNSIIKLSCGD-IGQKKFYSFSFAMFVLLGPLIFIIFSFTAII-ISVFRISDTQARFKTFSTCTPQLLIICLY
Medaka_OR13.1            MGIVL--HASSLSFCDS-K-ILQCFCDHASITKLACGENVAFVQEVAFANAMVTLLFPLSFIIFSYFSII-VAVLKMTKAEGYHKVLSTCAPQILTTCLY
Medaka_OR13.3            MTIVL--HALTLSYCKSNI-IAQCYCDHISLTNQGCGGNLREVQVTALCIAMLCLLLPLAFILFSYVSII-VVILKMSSSSGRKKTISTCTPQIFITCLF
Stickleback_OR1.25       LTIVL--HTLTLPFCKSNV-IAQCYCDHISITSQACGENVQTVLVTALCVAMFCLLLPLAFILFSYILIF-ADILKMSNATGRMRTISTCTPQILITCLF
Fugu_ORUn.8              VAVIV--QALALPYCKSNV-IVQCFCDHISITNLACGEGVQSVRVGTLCIALLCLLLPLTFIVFSYVTII-VVIAKMSSTAGRRKTLSTCTPQLFVTCLF
Pufferfish_OR16.7        VTIIV--QALALTFCEPKL-IVQCFCDHISITNQACGEGVQSVRVSTLCIALFCLLLPLAFIVFSYVSII-VVIMRRSNGAGRRKTLSTCTPQLFITCLF
Frog_OR669.1             AVITI--LDSTVPICNKNK-IKSCFCTNTGLTALSCAD-VTFVKRLAFGLAMFVLLLPFTFIIFSYIAII-KAICSRDHFENWRKTFYTCATHLLVIGLY
Amphioxus_105.1          SLPLI--LVRDAGDNVSCLTKTRSFQPAFVIRIIGMVAQG----------LSVLVIIGLYFIVLKEAKRQQERDEQRPVKFYKTRGFKTMAPLAILLTTT
Amphioxus_39.1           IGQTL--------IKRLQNPCESLFCQPEAFTSTSPAAIFKNVCIVGVLLAVLAIIIVYYLVYKEARKQQ-ERDEHRNLWLYQTKAFKTLVPHIVVLVVS

                                310       320       330       340       350       360       370       380       390       400
                         ....|....|....|....|....|....|....|....|....|....|....|....|....|....|....|....|....|....|....|....|
LOR1                     VVSLLFDLLYMRFGSQ------TLSQNVKNFMAMEFLLVPPIINPLIYGFKLTQIRNRIIQ------------FLSGKRI--------------------
LOR2                     VVSLLFDLLYMRFGSQ------ALSQNVKNFMAMEFLLVPPIINPLIYGFKLTQIRNRIIQ------------FLSGKRI--------------------
LOR3                     VVSLLFDLLYMRFGSQ------TLSQNVKNFMAMEFLLVPPIINPLIYGFKLTQIRNRIIQ------------FLSGKRI--------------------
LOR4                     VVSLLFDLLYMRFGSQ------TLSQNVKNFMAMEFLLVPPIINPLIYGFKLTQIRNRIIQ------------FLTGKRI--------------------
LOR5                     VVSLLFDLLYMRFGSQ------TLSQNVKNFMAMEFLLVPPIINPLIYGFKLTQIRNRIIQ------------FLSGKRI--------------------
LOR6                     VVTLLFDLLYMRFGSQ------TLSQSVKNFMAMEFILIPPTINPLIYGFKLTKIRNRIQS------------FLNCKFHS-------------------
LOR7                     VVTLLFDLLYMRFGSQ------TLSQSVKNFMAMEFILIPPTINPLIYGFKLTKIRNRIQS------------FLNCKFHS-------------------
LOR8                     VVTLLFDLLYMRFGSQ------TLSQSVKNFMAMEFILIPPTINPLIYGFKLTKIRNRIQS------------FLNCKFHS-------------------
LOR9                     VVTLLFDLLYMRFGSQ------TLSQSVKNFMAMEFILIPPTINPLIYGFKLTKIRNRIQS------------FLNCKFHS-------------------
LOR10                    VGTLLFDLLYMRFGSK------TLSQSVKNFMAIEFILIPPTINPLIYGFKLTKIRNRIQS------------FLNCKFHS-------------------
LOR11                    SCLCAYDVIVVGLEAD-------FPKTVRLIMALQIILYHPLFNPIMYGLKMKEIHKHLKR------------LFFPAKIK-------------------
LOR12                    SCLCAYDVIVVGLEAD-------FPKTVRLIMALQIILYHPLFNPIMYGLKMKEIHKHLKR------------LFFPARIK-------------------
LOR13                    SCLCAYDVIVVGLEAD-------FPETFRLIMTLQMILYHPLFNPIMYGLKMKEIHKHLKR------------LFFPAKIK-------------------
LOR14                    SCLCAYDVIVVGLEAD-------FPKTFRLIMTLQMILYHPLFNPIMYGLKMREIHKHLKR------------LFFPAKIK-------------------
LOR15                    SCLCAYDVIVVGLEAD-------FPKTFRLIMTLQMILYHPLFNPIVYGLKMKEIHKHLKR------------LFFPAKIK-------------------
LOR16                    SCLCAYDVIVVGLEAD-------FPKTVRLIMALQIILYHPLFNPIMYGLKMKEIHKHLKR------------LFFPARIK-------------------
LOR17                    SCLCAYDVIVVGLEAD-------FPKTFRLIMTLQMILYHPLFNPIMYGLKMKEIHKHLKR------------LFFPAKIK-------------------
LOR18                    SCLCAYDVIVVGLEAD-------FPKTVRLIMALQIILYHPLFNPIMYGLKMKEIHKHLKR------------LFFPARIK-------------------
LOR19                    SCLCAYDVIVVGLEAD-------FPKTVRLIMALQIILYHPLFNPIMYGLKMKEIHKHLKR------------LFFPAKIK-------------------
LOR20                    SCLCAYDVIVVGLEAD-------FPKTVRLIMALQIILYHPLFNPIMYGLKMKEIHKHLKR------------LFFPAKIK-------------------
LOR21                    SCLCAYDVIVVGLEAD-------FPKTVRLIMALQIILYHPLFNPIMYGLKMKEIHKHLKR------------LFFPARIK-------------------
LOR22                    SCLCAYDVIVVGLEAD-------FPKTVRLIMALQIILYHPLFNPIMYGLKMKEIHKHLKR------------LFFPARIK-------------------
LOR23                    SCLCAFDVITVQLEAD-------FPKTVRFIMTLQIVMYHPLFNPIMYGLKMKEIHKHLKR------------LFFPAKIK-------------------
LOR24                    SCLCAFDVITVQLEAD-------FPKTVRFIMTLQIVMYHPLFNPIMYGLKMKEIHKYLKR------------LFFPAKIK-------------------
LOR25                    SCLCAFDVITVQLEAD-------FPKTVRFIMTLQIVMYHPLFNPIMYGLKMKEIHKYLKR------------LFFPAKIK-------------------
LOR26                    SCLCAFDVITVQLEAD-------FPKTVRFIMTLQIVMYHPLFNPIMYGLKMKEIHKYLKR------------LFFPAKIK-------------------
LOR27                    ----------------------------------------------------------------------------------------------------
LOR28                    ----------------------------------------------------------------------------------------------------
LOR29                    ----------------------------------------------------------------------------------------------------
LOR31                    SITVFCDVALSRIDLE------NLNPFLAVILSLEFVVIPPILNPLVYGLKLPEIRKLILK------------MLSGPKPDKQTKSHK------------
LOR32                    SITVFCDVALSRIDLE------NLNPFLAVILSLEFVVIPPILNPLVYGLKLPEIRKLILK------------MLSGPKPDKQTKSHK------------
LOR33                    SITVFCDVALSRIDLE------NLNPFLAVILSLEFVVIPPILNPLVYGLKLPEIRKLILK------------MLSGPKPDKQTKSHK------------
LOR34                    SITVFCDVALSRIDLE------NLNPFLAVILSLEFVVIPPILNPLVYGLKLPEIRKLILK------------MLSGPKPDKQTKSHK------------
LOR35                    SITVFCDVALSRIDLE------NLNPFLAVILSLEFVVIPPILNPLVYGLKLPEIRKLILK------------MLSGPKPDKQTKSHK------------
LOR37                    YLVGSVTFLSHNLNIP-------IPTDVNTFMGLMYILFPATVNPIIYGVRTKEIRNGFFRIFK---LRVQTAMTVKVAPAGKRQM--------------
LOR38                    YLVGSVTFLSHNLNIP-------IPTDVNTFMGLMYILFPATVNPIIYGVRTKEIRNGFFRIFK---LRVQTAMTVKVAPAGKRQM--------------
LOR39                    YLVGSVTFLSHNLNIP-------IPTDVNTFMGLMYILFPATVNPIIYGVRTKEIRNGFFRIFK---LRVQTAMTVKVAPAGKRQM--------------
LOR40                    YLVGSVTFLSHNLNIP-------IPTDVNTFMGLMYILFPATVNPIIYGVRTKEIRNGFFRIFK---LRVQTAMTVKVAPAGKRQM--------------
LOR41                    YLVGSVTFLSHNLNIP-------IPTDVNTFMGLMYILFPATVNPIIYGVRTKEIRNGFFRIFK---LRVQTVMTVKVAPAGKRQM--------------
LOR42                    YLVGSVTFLSHNLNIP-------IPTDVNTFRGLMYTLFPATVNPIIYGVRTKEIRNGFFRIFK---LRVQTVMTVKVAPAGKRQM--------------
LOR43                    EIVGTFTILSHRFTN--------VSADLQKIMGMLIFLVPPLLNPIVYGLYTSEIRNTMLR-------------VIKKRVSN------------------
LOR44                    EIVGTFTILSHRFTN--------VSADLQKIMGMLIFLVPPLLNPIVYGLYTSEIRNTMLR-------------VIKKRVSN------------------
LOR45                    EIVGTFTILSHRFTN--------VSADLQKIMGMLIFLVPPLLNPIVYGLYTSEIRNTLLR-------------VIKKRVSN------------------
LOR46                    EIVGTFTILSHRFTN--------VSADLQKIMGMLIFLVPPLLNPIVYGLYTSEIRNTMLR-------------AIKKRVSN------------------
LOR47                    EIVGTFTILSHRFTN--------VSADLQKIMGMLIFLVPPLLNPIVYGLYTSEIRNTMLR-------------AIKKRVSN------------------
LOR48                    VFNGMSIITLHRFP---------HYSDYRKLCTILFHIIPCSINPIIYGVQSKEIKKFFSK------------SFKSKKTLPSFHQS-------------
LOR49                    VFNGMSIITLHRFP---------HYSDYRKLCTILFHIIPCSINPIIYGVQSKEIKKFFSK------------SFKSKKTLPSFHQS-------------
LOR50                    VFNGMSIITLHRFP---------HYSDYRKLCTILFHIIPCSINPIIYGVQSKEIKKFFSK------------SFKSKKTLPSFHQS-------------
LOR51                    FLCGMFVVILHRFP---------QYSDYRKLCSILFHIVPGSLNPIIYGIQSKEMKKFFLK------------LFKSKNTLPLI----------------
LOR52                    VFNGMSIITLHRFP---------HYSDYRKLCTILFHIIPCSINPIIYGVQSKEIKKFFSK------------SFKSKKTLPSFHQS-------------
LOR53                    VFNGMSIITLHRFP---------HYSDYRKLCTILFHIIPCSINPIIYGVQSKEIKKFFSK------------SFKSKKTLPSFHQS-------------
LOR54                    VFNGMSNIALHRFP---------QYSDYRRLCSILFHIVPGSLNPIIYGVQSKEMKKFFLK------------LFKSKNTLPLI----------------
LOR55                    VFDGMSNIALHRFP---------QYSDYRRLCSILFHIVPGSLNPIIYGVQSKEMKKFFLK------------LFKSKNTLPLI----------------
LOR56                    VFDGMSNIALHRFP---------QYSDYRRLCSILFHIVPGSLNPIIYGVQSKEMKKFFLK------------LFKSKNTLPLI----------------
LOR57                    VFNGMSNIALHRFP---------QYSDYRRLCSILFHIVPGSLNPIIYGVQSKEMKKFFLK------------LFKSKNTLPLI----------------
LOR58                    VFNGMSNIALHRFP---------QYSDYRRLCSILFHIVPGSLNPIIYGVQSKEMKKFFLK------------LFKSKNTLPLI----------------
LOR59                    VFNGMSNIALHRFP---------QYSDYRRLCSILFHIVPGSLNPIIYGVQSKEMKKFFLK------------LFKSKNTLPLI----------------
LOR60                    VFNGMSNIALHRFP---------QYSDYRRLCSILFHIVPGSLNPIIYGVQSKEMKKFFLK------------LFKSKNTLPLI----------------
LOR61                    LFSGMSNITLHRFP---------QYSDYRKLCSILFHIVPGSLNPIIYGVQSKEMKKFFLK------------LFKSKNTLPLI----------------
LOR62                    VFNGMSIITLHRFP---------HYSDYRKLCTILFHIIPCSINPIIYGVQSKEIKKFFSK------------SFKSKKTLPSFHQS-------------
LOR63                    FLCGMFVVILHRFP---------QYSDYRKLCSILFHIVPGSLNPIIYGIQSKEMKKFFLK------------LFKSKNTLPLI----------------
LOR64                    FLCGMFVIILHRFP---------QYSDYRKLCSILFHIVPGSLNPIIYGVQSKEMKKFFLK------------LFKSKNTLPLI----------------
LOR65                    FLCGMFVIILHRFP---------QYSDYRKLCSILFHIVPGSLNPIIYGVQSKEMKKFFLK------------LFKSKNTLPLI----------------
LOR66                    LVSGMILITLHCFP---------QYAEYRKISAILFNVVPGSLNPVIYGLQSKKIYKSLSN------------IFHPKRIMLF-----------------
LOR67                    LVSGMILITLHRFP---------QYAEYRKISAILFNVVPGSLNPVIYGLQSKEIYKSLSN------------IFHPKRIMLF-----------------
LOR68                    LVSGMILITLHRFP---------QYAEYRKISAILFNVVPGSLNPVIYGLQPKEIYKSLSN------------IFHPKRIMLF-----------------
LOR69                    LVSGMILITLHRFP---------QYAEYRKISAILFNVVPGSLNPVIYGLQSKEIYKSLSN------------IFHPKRIMLF-----------------
LOR70                    LVSGMILITLHRFP---------QYAEYRKISAILFNVVPGSLNPVIYGLQSKEIYKSLSN------------IFHPKRIMLF-----------------
LOR71                    LVSGMILITLHRFP---------QYAEYRKISAILFNVVPGSLNPVIYGLQSKEIYKSLSN------------IFHPKRIMLF-----------------
LOR72                    HGPIVISLSTTLQR--------LLMVRIKNIFYVFIYILPRCLSSLIYGLRDQTIRPVLMYY-----------LCCRLPK--FQ----------------
LOR73                    HASIIISLSTTLQR--------LLIVRIKSILYVFIYILPRCLSSLIYGLRDQTIRPVLMYY-----------LCCRLPK--FQ----------------
LOR74                    HGTIIVSLSTTLPR--------LLIIRIRSSFYVFIYILPRCLSSLIYGLRDQTIRPVLMYY-----------LCCRLPK--FQ----------------
LOR75                    HSSIIVSLSTIPQR--------ILFVHIRSTFYVFIYILPRCLSSLIYGLRDQTVRPVLMYY-----------LCCRLPK--FQ----------------
LOR76                    HASIIISLSTTLQR--------LLIVRIKSILYVFIYILPRCLSSLIYGLRDQTIRPVLMYY-----------LCCRLPK--FQ----------------
LOR77                    HSSIIISLSTIPQR--------ILFVHIRSTFYVFIYILPRCLSSLIYGLRDQTVRPVLMYY-----------LCCRLPK--FQ----------------
LOR78                    HSSMIVSLSTTLPT--------SQIIRIRSIFYVFIYVLPRCLSSLIYGLRDQTIRPVLMYY-----------LCCRLPK--FQ----------------
LOR79                    HSSMIVSLSTTLPT--------SQIIRIRSIFYVFIYVLPRCLSSLIYGLRDQTIRPVLMYY-----------LCCRLPK--FQ----------------
LOR80                    HSSMIVSLSTTLPT--------SQIIRIRSIFYVFIYVLPRCLSSLIYGLRDQTIRPVLMYY-----------LCCRLPK--FQ----------------
LOR81                    HSSMIVSLSTTLPT--------SQIIRIRSIFYVFIYVLPRCLSSLIYGLRDQTIRPVLMYY-----------LCCRLPK--FQ----------------
LOR82                    HTPSIQAISKVVDA--------ITSDRLHIVHYVFIIILPRCLSSLIYGLRDQTIRPVLMYY-----------LCCRLPK--FQ----------------
LOR83                    APQLLDILQQWFPK---------NRTDSLFAYYIIVQILPRSVSPIIYGIRDNTFRKYLKRY-----------LFCKDSIL-------------------
LOR84                    APQLLDILQQWFPK---------NRTDSLFAHYIIVQILPRSVSPIIYGIRDNTFRKYLKRY-----------LFCKDSIL-------------------
LOR85                    APQLLDILQQWFPK---------NRTDSLFAYYIIVQILPRSVSPIIYGIRDNTFRKYLKRY-----------LFCKDSIL-------------------
LOR86                    APQLLDILQQWFPK---------NRTDSLFAYYIIVQILPRSVSPIIYGIRDNTFRKYLKRY-----------LFCKDSIL-------------------
LOR87                    APQLLDILQQWFPK---------NRTDSLFAYYIIVQILPRSVSPIIYGIRDNTFRKYLKRY-----------LFCKDSIL-------------------
```

```
LOR88                    APQLLDILQQWFPK---------NRTDSLFAHYIIVQILPRSVSPIIYGIRDNTFRKYLKRY-----------LFCKDSIL-------------------
LOR89                    APQLLDILQQWFPK---------NRTDSLFAYYIIVQILPRSVSPIIYGIRDNTFRKYLKRY-----------LFCKDSIL-------------------
LOR90                    APQLLDILQQWFPK---------NRTDSLFAYYIIVQILPRSVSPIIYGIRDNTFRKYLKRY-----------LFCKDSIL-------------------
LOR91                    APQLLDALQKWFPK---------NFTDSLFAYYIIVQVLPRSISPFIYGIRDNTFRRYLKRY-----------LLCKDSKP-------------------
LOR92                    APQLLDALQKWFPK---------NFTDSLFAYYIVVQVLPRSISPFIYGIRDNTFRRYLKRY-----------LLCKDSKP-------------------
LOR93                    CPFIEVALFQ-IDL--------MLYVNVRYFNYITFILAPRCLSPLIYGLRDEKFLLALKHK-----------VPFGFHCVKIKARTGFVRQVGCRDRLP
LOR94                    CPFIEVALFQ-IDL--------MLYVNVRYFNYITFILAPRCLSPLIYGLRDEKFLLALKHK-----------VPFGFHCVKIKARTGFVCQVGCRDRLP
LOR95                    CPFIEVALFQ-IDL--------MLYVNVRYFNYITFILAPRCLSPLIYGLRDEKFLLALKHK-----------VPFGFHCVKIKARTGFVRQVGCRDRLP
LOR96                    CPFIEVALFQ-IDL--------MLYVNVRYFNYITFILAPRCLSPLIYGLRDEKFLLALKHK-----------VPFGFHCVKIKARTGFVCQVGCRDRLP
LOR97                    CPFIEVALFQ-IDL--------MLYVNVRYFNYITFILAPRCLSPLIYGLRDEKFLLALKHK-----------VPFGFHCVKIKARTGFVCQVGCRDRLP
LOR98                    CPFIEVALFQ-IDL--------MLYVNVRYFNYITFILAPRCLSPLIYGLRDEKFLLALKHK-----------VPFGFHCVKIKARTGFVCQVGCRDRLP
LOR99                    TPFIEGAVFQ-IDF--------MLFINVRFSSYILFALAPRCLSPLIYGLRDETFFHALKNY-----------EFFGLYKRNV-----------------
LOR100                   TPFIEGAVFQ-IDF--------MLFINVRFSSYILFALAPRCLSPLIYGLRDETFFHALKNY-----------EFFGLYKRNV-----------------
LOR101                   SPFIESTLLR-FDF--------MLFINVRYSNYVLFNLTPRCLSPLIYGLRDETFFHALKNY-----------EFFGLYKRNV-----------------
LOR102                   GPFIESTLLR-FDF--------MLFINVRYSNYVLFNLTPRCLSPLIYGLRDEAFFHALKNY-----------EFFGLYKRNV-----------------
LOR103                   SPFIESTLLR-FDF--------MLFINVRYSNYVLFNLTPRCLSPLIYGLRDETFFHALKNY-----------EFFGLYKRNV-----------------
LOR104                   SPFIESTLLQ-FDF--------MLFINVRYSKYVLFNLTPRCLSPLIYGLRDETFFHALKNY-----------EFFGLYKRNV-----------------
LOR105                   TPFIESTLLR-FDL--------MLFAHVRLSNFILFGLTPKCLSPLIYGLRDETFFHALKNY-----------EFFGLYKRNV-----------------
LOR106                   SPFIESTLLR-FDF--------MLFINVRYSNYVLFNLTPRCLSPLIYGLRDETFFHALKNY-----------EFFGLYKRNV-----------------
LOR107                   CPFIEAAVFQ-IDL--------ILFINVRFYNYVLFNLTPRCLSPLIYGLRDETFFHALKNY-----------EFFGLYKRNV-----------------
LOR108                   TPFIESTLLH-FDL--------MLFAYVRVSNLILFGLTPRCLSPLIYGLRDETFFHALKNY-----------EFFGLYKRNV-----------------
LOR109                   TPFIESTLLQ-FDL--------MLFFHVRLSNFILFGLTPKCLSPLIYGLRDETFFHALKNY-----------EFFGLYKRNV-----------------
LOR110                   TPFIESTLLR-FHL--------MLFTHVRLSNFILFGLTPKCLSPLIYGLRDETFFHALKNY-----------EFFGLYKRNV-----------------
LOR111                   TPFIESTLLR-FDL--------MLFAHVRLSNFILFGLTPKCLSPLIYGLRDETFFHALKNY-----------EFFGLYKRNV-----------------
Fugu_OR123-1             VICMLFDLLYMRFGTK------NLPESIQNFIAIQFILIPPILNPLIYGFKLQKIRRRIQY------------FLWTKHVCI------------------
Fugu_OR6765-1            IVCLLFDLVHMRFDSG------TLSDNARNFMAIQFLLFPPLINPLIYGIKLTPIRNRIQN------------FLGKEI---------------------
Fugu_OR4133-1            KICLLFDLLHMRFDSG------QLSEGARNFMAIQFLLFPPLINPLIYGIKLTPIRNRIQS------------FLCMKSL--------------------
Fugu_OR3630-2            SCLTVYDVLLLRLNTV-------VPKTVHFLITLQIIMYHPLFNPIVYGLKMKEISKQLKK------------LFGKKGLF-------------------
Fugu_OR8617-1            SCLTVYDVLLLRLNTV-------VPKTVHFLITLQIIMYHPLFNPIVYGFKMKEISKQLKK------------LFGKKGLF-------------------
Fugu_OR5510-1            SCLTVYDVLLLRLNTV-------VPKTVHFLITLQIIMYHPLFNPIVYGFKMKEISKRLKK------------LFGKKRFVLTCVYTLHHK---------
Fugu_OR117-1             SVTVFCDVALSRIDLE------SLNPFIAVVLSLEFVVIPPLVNPLVYGLKLPEIRKCILR------------MFHNPK---------------------
Fugu_OR2346-5            SLTVFCDIALSRNNIE------EINPFLAVIFSLEFVVIPPVLNPLVYGLKLEQIRREILR------------LFTRHNIII------------------
Fugu_OR5287-2            YLVGSVTFLSHNLNIN-------IPADVNTFMGVMYILFPATVNPIIYGVRTTEIRNGLLKIFK---LQVKKLI--KVSPV-------------------
Fugu_OR142-1             EFVATFTILSHRFNT--------VSADLQKIMGMLIFLIPPLLNPIVYGLYTGEIRNALLR-------------IIKKRSISV-----------------
Fugu_OR1026-2            SASGILIIALHRFP---------QYSDYRKICAILFHTIPGSMNPIIYGIQSKEIQKYLYK------------LFQARKIMSSY----------------
Fugu_OR8298-1            FASGILVIALHRVP---------QYSDYRKICAILFHIIPGSLNPIIYGVQSKEIQKFLLR------------QFFHQKVFAVKINYLVAPLVH------
Fugu_OR6030-1            YKPILAALSRIVSR--------IVLVRLQNVLYVCLFILPRCLSALIYGIRDQLIRPVLFYY-----------LCCRLTLSVFPSKGYK-----------
Fugu_OR59-1              YKPILAALSRIVTR--------LVLIRVQNVLYVCFFILPRCLSALIYGIRDQLIRPVLFYY-----------LCCRLTLSVFPAKGYK-----------
Fugu_OR4208-3            EPLLKQALLLWFPR---------NYSDSLFACYIIVQILPRSISPIIYGVRDKTFRRHLTRN-----------LLCRPPRAPEEPAQT------------
Fugu_OR4208-2            APALRELLQKLFPK---------NSTDILFVSYIVVQVLPRSTSPMIYGLRDPTFRKYLKRY-----------MLARSS---------------------
Fugu_OR6818-1            CPFIESAIFQ-IDY--------MLFINVRYFNYVTFILAPRCLSPLIYGLRDEKFFNALKYL-----------ALCGLYKKRLDLFDE------------
Fugu_OR7903-1            CPFIESAIIK-IDL--------LLYINVRYFNYVTFILAPRCLSPLIYGLRDEKFLNALKYL-----------ALCGLYKKKIDY---------------
Pufferfish_OR8981-2      SMCLLFDILHEGFSSE------RIPESARNFIAIQFLLVPPLINPLIYGLKLNKVRNRINR------------FMCRKS---------------------
Pufferfish_SCAF8981      NMCLLFDMLYEKLSTE------KIPKSTQNFIAIEFLFFPPLINPLIYGLKLNKVRNRIEK------------FLCRKS---------------------
Pufferfish_OR15134-1     SSFGIYDVLLLRLNTT-------VPKTVRFLITLQMIMYQPLFNPIIYGLKMREISKHLKK------------LFARKVLG-------------------
Pufferfish_OR14339-2     SITVFCNVALSRIDLE------SLNPYIAVILSLEFVVIPPLVNPLVYGLKLPEIRKCVLR------------MFQNPEK--------------------
Pufferfish_OR14328-1     SINFFCDVALSRIDLE------SLNPYIAVILSLEFVVIPPLVNPLVYGLKLPEIRKCILR------------MFQNPEKKV------------------
Pufferfish_OR14677-8     YLVGSVTFLSRNLNIY-------IPADVNTFMGVMYILFPATVNPIIYGVRTTEIRNGFFKLCK---IRVKKLT--KVSPVGKRQT--------------
Pufferfish_OR14536-2     YTPILTALSRTVIR--------IVLVRVQIVLYVWFFILPRCLSALVYGIRDRTIRPVFFYC-----------LCCQLTISVFPAKGYK-----------
Pufferfish_OR14536-1     YTPILTALSRTVIR--------IVLVRVQIVLYVWFFILPRCLSALIYGIRDRTIRPVFFYC-----------LCCQLTISVFPAKGYK-----------
Pufferfish_OR10960-2     EPLLKQALQLWFPR---------SYSDSLFACYIIVQILPRSISPIIYGVRDQTFRRYLRRN-----------LLCRAQRAPERPGDGGPKPAACFCLD-
Pufferfish_OR10960-3     TPALRDALQRRFPE---------NSVDVLFASYVLVQILPRSLSPLVYSVRDQTFRRYLRRY-----------LLRWRR---------------------
Pufferfish_OR12434-1     CPFIESVVFE-NDK--------VIFSYVRFFNYVVFYIAPRCLSPLIYGLRDEKIFLALKVR-----------VLFLRSW--------------------
Medaka_ORUn.33           YLVGSVAFLSRDL-----------SSGVGAFMGVLYILLPATVNPIIYGIRTKEIRNGLLRLLK---VRTRNGSQ-KTSPEGRRRS--------------
Medaka_ORUn.2            LTVALVAFLSYRVRNS-------LPTAVRVFFSTMYLLFPSCVNPIIYGIRTTEIRQHIMKTLT---RQT------RVSGTC------------------
Medaka_OR21.18           TASLLFDIMYSRYGSA------SLPQTLKNFMAIQFLLGPPLLNPVVYGLILTKIRKRMIR------------VLYVVCNRLKLKNNV------------
Medaka_OR21.19           LVTILFDLMNMRLTSK------NLDQIFQNFIAMEFLLIPPIMNPLIYGFKLTKIQRKIAK------------LTTFRSK--------------------
Medaka_OR21.20           MVTILTDVISLRLGFD------GMPQTLQNLIAIEFLLIPPIMNPLIYGFKLTKIRYKIIK------------LLTFRVK--------------------
Medaka_OR13.14           SVLIEDAAFK-VSL--------TLYNNIRYFNYVMFFLAPRCLSPFIYGLRDELFFQALKYY-----------ASCGLYKR-------------------
Medaka_OR13.12           CPFIEAAVYN-VDP--------IVYNNLRFFNYIVFFLAPRCLSPLIYGLRDELFFQALKYH-----------ALRGLLKNI------------------
Medaka_ORUn.23           NNPVIVALSKLLTR--------MAFLWIQGIFYIFFIIFPRCMTSLIYGLRDQTIRPVLMYH-----------LSCHFKVSVGGLKK-------------
Medaka_ORUn.21           YNPLVIALSKVLTR--------MAFLWIQNIFYVTFIIFPRCMTSLIYGLRDQTIRSLLLDH-----------LCCRLKVRIRG----------------
Medaka_OR14.15           YNPLVVALSKVLTR--------MAFLWIQNIFYVTFIIFPRCMTSLIYGLRDQTIRSLLLDH-----------LCCRLKDRIRG----------------
Medaka_OR14.13           FACGMINILLHRFP---------EYSYYRSITAFMFHILPGTLDPIIYGVQSKEIRHFLSK------------LLMSKKVVSRFR---------------
Medaka_OR14.4            SISGVSIIVLHRFP---------QYSEYRKLSAILFVMLPPSLNPIIYGVQSREIQKFLSE------------KCCSRKIPFEC----------------
Medaka_OR14.6            SFSGVSIVVLHRFP---------QYSEYRKLSAILFVMIPPSLNPIIYGVQSREIQKFLSE------------KCCSRKRLKKNPI--------------
Medaka_OR13.4            ECLGLFTIISHRLQN--------ISPHLRRFMGLSTLIFPPTLNPIIYGLKTKEIREKILQ-------------FFRIGVHPLKPMFS------------
Medaka_OR13.5            QVNTTFTLISHRFEN--------ASPFLRRALGVSVLIFPPFLDPLIYGLRTRELRQCMVV-------------FLKRNFSFTK----------------
Medaka_OR13.6            QINSNAAYILHRSQS--------KLTVLRKAFGFSVILFPPFLDPLIYGLNTRELRQCIIM-------------FLRRNAGSTK----------------
Medaka_OR13.7            EVVGTFTILSHRFKD--------VSADLQKIMGMLIFLVPPLLNPLVYGLYTSEIRKTLLG-------------VFKNRVI-------------------
Medaka_ORUn.1            TGSLLFDGAFS-RYGG------TSIEMLQNALAAEFLVVPPALNPIIYGMNLKEVRSRIYF------------RFTTRPKLSM-----------------
Medaka_OR21.2            SCLMIYDIIIIRLESN-------FSKTARFIMTLQMIMYNPLCNPFIYGLKMTEIYKHLKK------------LFCFSRYYSSS----------------
Medaka_OR21.3            SCLAIYDVTIVRVESN-------LSKTARFIMTLQLIMYTPLCNPIIYGLKMTEIYKHLRK------------LFCNSRCVSSS----------------
Stickleback_OR1.1        YLVGSVTFLSHNLNIA-------IPTNVNTLMGVAYILLPATINPVIYGVRTKEIRSGFLKLFS---AK-------RVSAAGTTKT--------------
Stickleback_OR16.3       LGTILLDFMHIRYGSN-------LPQAFKNFIAIEYLVILPIMNPLIYGFKLTNIRKRIVA------------VITFKMTLLIS----------------
Stickleback_OR1.12       CPLIEAAVLQ-INL--------ILFINVRYFDYITFILAPRCISPLVYGLRDEMFFHALKYY-----------ALCGLYKKQSSNIP-------------
Stickleback_OR1.13       CPFIETAVLQ-INF--------ILYINVRYFDYIAFILAPRCISPLVYGLRDEMFFQALKYY-----------ALCGLYNKQSTNVP-------------
Stickleback_ORUn.9       YSPVLMALSRSVNR--------LLFVRLQNVLYVAIFIFPRCLSSLIYGLRDKSIQPVLLQH-----------LSCGLKLPIGTSPPSPRDVDV------
Stickleback_OR7.1        FYMINPINQWKGNR--------KMAIHIQYGLFLVLIILPKCLSPLIYGLRDQAFRHVFEYY-----------FTFGSVKRFPKS---------------
Stickleback_ORUn.6       VLSALSNIVLHRFP---------QYSDYRKLSSILFHIVPGSLNPIIYGVQSKEVQHFLFK------------WFQSKKVLPLL----------------
Stickleback_ORUn.8       LISGMCIIVLHRFP---------QYSDYRKLSAILFLIVPGSLNPIIYGMQSAEIGKALSK------------LFPSKQVMP------------------
Stickleback_OR1.18       EIVSTFTILSHRFKN--------VSADLQKIMGMLIFLVPPLLNPIVYGLYTSEIRNTLLT-------------VSANIFTQVGWLR-------------
Stickleback_OR16.10      SITVFCDVVLSRIDLE------ALNPFLAIILSLEFVVIPPMVNPLVYGLKLAEIRKCVLK------------VFLRSVPVKTNHKK-------------
Stickleback_OR16.32      VASLLFDLLYMRFGSK------DLPQGAQNVMAMEFLLIPPIINPLIYGLKLTKIRNSIQN------------VMCSKNSRVGLQKKKKFFKCLSFGKNT
Stickleback_OR16.1       SITVFCDVALSRIDLE------ALNPFLAIILSLEFVVIPPIVNPLVYGLKLAEIRKCVLN------------VFSHSEPVKTNHKK-------------
Stickleback_OR16.5       SSTVFCDVALSRIDLE------ALNPFLAIILALEFVVIPPMVNPLVYGLKLAEIRKCVLK------------VFLRSEPVKTN----------------
Stickleback_ORUn.1       CSLVTYDVIAVKLESE-------IPKAIRFIMTLQVILYHPLLNPIIYGLKMTEISKHIRR------------LFHQDKHN-------------------
Stickleback_ORUn.17      TCLISYSLILDQLELD-------SPKTVRFIITIQAVIYQPLFNPLIYGLKMRRIYDHLKK------------LFCRMVLCLHTDESQCFVS--------
Zebrafish_OR126-7        TVALLFDVLYSRFGSK------NVPHSVRNFMALEFLLIPPILNPLIYGLNLTIVRQQVIK------------LFSKNKVGI------------------
Zebrafish_or126-3        TVAFLFDVLFSRYGSK------NVSQNLRNFMALEFILIPPFLNPLIYGLNLTAVRQEVIR------------LFSRKQLEISK----------------
Zebrafish_OR123-1        VFWSLFSTLYELFGIS------SLSQEFKNFLSVTFMIIPPLINPVIYGIVLTPIRTKAKH------------VFQSFRPKHID----------------
Zebrafish_OR125-1        TFAFLFDFMYNRYGSK------DIPESLRHFLALELVIVPPLFNPLMYGLNIRAVRKRVFT------------SCGAAKVNILEKVHKFIMH--------
Zebrafish_OR127-1        SIASFCDIALSRNDSD------KIK-ILTIIFSVEFLVIPPVLNPLIYGLNLPEIRKKIAC------------LFRRSKIGHFIE---------------
Zebrafish_OR128-10       SAALFCEVTLSRVQNL------ELPIGLSIILSLEFLIVPPILNPLIYGLNFPEIRKKIMC------------IVKTCK---------------------
Zebrafish_OR116-2        EIVATFTILSHRFTS--------VNADLQKIMGMLIFLVPPLLNPIVYGLNSHELRNNLLK-------------VIHRKIAHM-----------------
Zebrafish_zor6a          YFVGSVTFLSRNLNIP-------IPIDVNTFLGVMYIVFPASVNPIIYGVRTKEIRTALFKIFK---NKINKVSIVKVSTIRN-----------------
Zebrafish_OR117-1        EMVTAVAIISYRIPD--------FPPTAQRVCGLMIYAVLPVVNPVIYGLKMKDIRIALFV-------------VLRKRKVVPGRKTDVVKIRK------
Zebrafish_OR115-15       LISGLIIIVLHRFP---------AYSDYRKIASLLFHIIPSILNPIIYGVQSSEVRHVLIQVL----------SFKKRRAVKDV----------------
Zebrafish_OR133-7        VGVIQEAIYVYTDF--------LTALNVMYFCFLVFIIFPKCLSPLIYGMRDQAFCCLFKYY-----------FTFGKGKIKPVTIELHV----------
Zebrafish_OR132-1        YATIERTLYMVIGSGS------PLFINLRYLNYIIVLILPRCLSPLIYGMRDEAVWPLFKYF-----------VCFRSSKIKPSVNVH------------
Zebrafish_OR134-1        SPSVEGILNIIFPG---------RVLEIRFANYLIVYILPRFLSPIIYGVRDKKFRKYLRRY-----------FVCGVSTMETRVECKDGD---------
Zebrafish_OR131-2        TPYIDILTLK-VDI--------MLFINVKFSNFMLFWIFPRCLSPLVYGLRDKKFYNALKYY-----------AFCGIYVYKKHKIKDSKTIGGALSISI
Zebrafish_OR131-1        TPYTEMSLWK-IDV--------MLFLNVRYSNFILFWILPRSLSPLVYGLRDKKFYYALKYY-----------AFCGVFVVSKHKVRDIKISRRKRC---
Goldfish_OR3             SFALLFDVLYTRYGSM------TMPQDLRNFMALEFLLVPPMLNPLIYGLNLTKLRKEVLR------------LLKKEV---------------------
Goldfish_OR2             FLPRCFIYLSS-NIGI------NFSTDLRLVIIMMYSLFPPMINPLIYCLKTKDVKETLLK------------KIKVSMNPLFIPTKVNVSTVCV-----
Rainbow trout OR500-1    ITVTLFDNLQG-WNNV------NSTLNMRNAMAVQFLVIPPVFNPLIYGLNLQQIRRAVFR------------KCNAHKIIDMRC---------------
Cutthroat trout OR600-1  LGCGFTIIVLHRFP---------AFADLRKVSSVLGSVVPTCLNPIIYGLQTKEIKQRIFR------------VFHRAKVAA------------------
Brown trout OR500-1      ITVTLFDNLQG-WNNV------NITLNMRNAMAIQFLVIPPVFNPVIYGLNLKQIRRAVFR------------KCNARKIIDMRC---------------
Atlantic salmon SORB     LISGSIIIILHRFP---------HYSDYRKVAALLFHIIPGSLNPIIYTLRNPTHRHVPVIR--------------------------------------
Medaka_mfOR2             SCLGSFEIIIGFFWSE-------YPKTLHLTVILQIIIYQPLFNPIIYGLKMKEISKHVKM------------LIWGR----------------------
Medaka_mfOR1             SCLSSFDVIIGFFGSG-------YPKTLHLTVMLQIIIYQPLFNPIIYGLKMKEISKHVKM------------LIWGR----------------------
Medaka_OR_Y3             ISCGLLNIVLHRFP---------EYSDQRKLIVILFHIIPGSLNPIIYGLQSKEVQRFFRSKGL---CPHFDVAAMHSLFFSRFKK--------------
Medaka_ORY1              SISGVSIIVLHRFP---------QYSEYRKLSTILFVMLPPSLNPIIYGVQSREIQKFLSE------------KCCSRKIPFEC----------------
Frog_OR-52D1             VADVLFEILLPRFPST------TLPYELRVLMSVQAFVIAPILHPLIYGWKLREIRLRVLQ------------MFGAKPIADLHNNL-------------
Frog_OR-52E8             VVDILCEVVLLRFPFN------SIPYELRIIISVQAFVFAPLLNPLMYGLKLRKIRVKIGQ------------IFCPKNKTEGHGKM-------------
Frog_OR-52A1             YLCGLASSIVYRMETT-------ISPDVKNLFSAIYLMVPATLNPFIYGLRVSEIRKSLMKYG----MKKNLFPS-------------------------
Frog_OR-52P1             YLCALASTIVQRMETS-------ISTDIKNLFIALYLIIPALLNPFIYGFRVSEIRKSLLGYWK---ERKNVFSSS------------------------
Frog_OR-52A5             N----NLLEIYLME---------YMYLLPLGSFFLFMCLPRFISPLIYGIRDEVFRNYIKRF-----------MLCRQLRVHKIKIVVFK----------
Frog_OR-2M5              T----SYIEANVKE---------YVVLLAISNFLFFMCLPRFLGPLIYGLRDEVFGKHLR-------------------KLHSVCIEIR-----------
Frog_OR-2M2              FIPRVFVYSTS-QIPL------TLDADINVLLLCLYTFIPHLANPVIYCLRTKDIRNIFAQ------------SFNNIFHAKI-----------------
zebrafish_OR10.1         FVPIVIIFNLT-FFGI------IWSPNVGLVCLSLSSLLPPCVNPVIYSLKTKEIRSRVYS------------LFTRRLFVHPLKNGH------------
zebrafish_OR10.14        YLPITITYS----LAS------IINTNTRIINLSLTSALPPMLNPIIYTFKTEEFMVSVKK------------LLKRKIIFPYLK---------------
zebrafish_OR15.39        YMPVIIAYVLG-NLRL------VQNVDLLTAILTVSVTVPAMLNPIIYSLKTDELKERMVK------------LFRQSKVAQQIIKTEVKALQQ------
Medaka_ORUn.6            YIPFFVIYTMG-FYLG------PVNPDLRVLSLSMASCLPPCINPVVYSLKTKELKTRALA------------LVRKKGQVVSVNSNRIIVVLRTRK---
Medaka_ORUn.28           FFPILITFT----NTN-------IHPNHRIINLSLTAVFPPMLNPIIYVLQTQEIKASFKK------------VLKIRSRFKVSGK--------------
Medaka_ORUn.27           FTPVLITFT----LMD------KIHPNNRIINLSLTSVFPPMLNPIIYVLQTKEIQTSLKK------------ISKIYRKSKMKIKSLP-M---------
Stickleback_OR7.3        YIPIFTIFLMG-LYVR------SIDPDQRVLSLSLATCIPPCINPIVYSLKTREIKTRALA------------LVQR--IKTSP----------------
Stickleback_OR7.4        YVPIITIFTLG-FFLR------LTDPDQRVLSLSLASCIPPCINPIVYSLKTKEIKMRAVA------------LVRRNVIGTDQRKMKPLRVKKSFRL--
Stickleback_OR7.8        FIPVLITFT----LME------KINPNARIMNLSLTSILPPTLNPIIYVLQTQEIKKSVKK------------LLKIRRKSKMIKKCTPNMTHSL-----
Fugu_ORUn.83             FIPLLITFT----LME------NIHPNARIINLSLTSVFPPMLNPIIYVLQTREIKDSLKR------------LLKLGRKSKTTPRKLPNLPQTAACKC-
Fugu_ORUn.20             YIPLFTIFILG-FYVR------LIDADQRVLSLSLASCIPPCVNPIVYSLKTREIKIRAVA------------LIRKNKIKGTKF---------------
```

```
Pufferfish_OR7.1         FIPLLITFT----LME------NIHPNARIINLSLTSVFPPMLNPIIYVLQTQEIKDSLRR------------LLKNSQSLKWAQRKHPILPQPSGSRC-
Pufferfish_OR7.3         YIPLFTIFIFG-FYVR------LIDADLRVLSLSMASCIPPCINPIVYSLKTKEIKIRAVA------------LFRKHKIRAIKA---------------
zebrafish_OR10.29        YMPRFTVYLTT-FLQI------QISKDFRILLVLIYCLVPPLVNPFIYCLRTQEIRMIVSR------------WVSRQQALR-VACKINVITL-------
zebrafish_OR10.31        YVPRFAVYITSNVPNA------QMDKAEKIALVMFYSLLPPLMNPFIYFIRIREIRQVFLK------------CCAQRKRM--ISSSTTVSK--------
zebrafish_OR15.58        YLPRTFVYITN-ISGY------ELSNDTRMVVSMMYSLIPAVINPFIYCFRTKEIKEAIFK------------RFKKNK----VDRKISLG---------
zebrafish_OR15.57        YVPRCVVYVHD-VT-I------AISPGIRVMLIMWYTLIPPIVNPMIYCFRTKEIKDAIKK------------KLKDRK----VNVQI------------
Medaka_OR13.1            YVPRCVVYITD-NLKV------KVSPDARIIIALLYSLIPAAVNPLIYCLKTADIKEALMK------------RFKNRK----INLAFKSNTKP------
Medaka_OR13.3            YLPRCFVYVAS-TVGF------SFSLDIRILLILLYSLLPPALNPLIYCFKTRDIKLTLLR------------KLKMYR----VAIEIQMFAK-------
Stickleback_OR1.25       YLPRCFVYVSN-TVGY------SFSLDVRILVVLLYSLLPAAVNPIIYCFKTQYIKQHLIK------------KIKTTR----IGIQLNLFFL-------
Fugu_ORUn.8              YLPRCFVYVAN-TVGF------YFSIDVRILLTLLYSLVPPAVNPFIYCFKTQDIKKILMK------------KLRNTG----FGMKVNIYS--------
Pufferfish_OR16.7        YLPRCFVYVAN-TVGF------YFSVDVRILLVLLYSLVPPAVNPFIYCFKTQDIKQTLIK------------KLRNTR----IGTEVNVSF--------
Frog_OR669.1             FIPRIFVYISN-QVQL------ILEEDLNVLLLCLYTFVPHMANPIIYCLRTKEIRKTIAR------------FFQKIVIKVKNPISVSVIVN-------
Amphioxus_105.1          IVSTLFMTVTVAQGNARDEQPSVAVVILEKASILLNLTLSSMLNPIIYSLRLPEFRRAMREMCGRRPTAVAPAVPTSQRRVHVIEVRGCDSASNAGVGLD
Amphioxus_39.1           VASYVFLVASGRALLNDGEKASTSLLITVIVANRIFLTLSSMVNPIVYSFRHPEFRQALGELFS---PTSIPSVPAPVPPPVTLQRRHDVAVIEVPHYHD

                                 410       420       430
                         ....|....|....|....|....|....|..
LOR1                     --------------------------------
LOR2                     --------------------------------
LOR3                     --------------------------------
LOR4                     --------------------------------
LOR5                     --------------------------------
LOR6                     --------------------------------
LOR7                     --------------------------------
LOR8                     --------------------------------
LOR9                     --------------------------------
LOR10                    --------------------------------
LOR11                    --------------------------------
LOR12                    --------------------------------
LOR13                    --------------------------------
LOR14                    --------------------------------
LOR15                    --------------------------------
LOR16                    --------------------------------
LOR17                    --------------------------------
LOR18                    --------------------------------
LOR19                    --------------------------------
LOR20                    --------------------------------
LOR21                    --------------------------------
LOR22                    --------------------------------
LOR23                    --------------------------------
LOR24                    --------------------------------
LOR25                    --------------------------------
LOR26                    --------------------------------
LOR27                    --------------------------------
LOR28                    --------------------------------
LOR29                    --------------------------------
LOR31                    --------------------------------
LOR32                    --------------------------------
LOR33                    --------------------------------
LOR34                    --------------------------------
LOR35                    --------------------------------
LOR37                    --------------------------------
LOR38                    --------------------------------
LOR39                    --------------------------------
LOR40                    --------------------------------
LOR41                    --------------------------------
LOR42                    --------------------------------
LOR43                    --------------------------------
LOR44                    --------------------------------
LOR45                    --------------------------------
LOR46                    --------------------------------
LOR47                    --------------------------------
LOR48                    --------------------------------
LOR49                    --------------------------------
LOR50                    --------------------------------
LOR51                    --------------------------------
LOR52                    --------------------------------
LOR53                    --------------------------------
LOR54                    --------------------------------
LOR55                    --------------------------------
LOR56                    --------------------------------
LOR57                    --------------------------------
LOR58                    --------------------------------
LOR59                    --------------------------------
LOR60                    --------------------------------
LOR61                    --------------------------------
LOR62                    --------------------------------
LOR63                    --------------------------------
LOR64                    --------------------------------
LOR65                    --------------------------------
LOR66                    --------------------------------
LOR67                    --------------------------------
LOR68                    --------------------------------
LOR69                    --------------------------------
LOR70                    --------------------------------
LOR71                    --------------------------------
LOR72                    --------------------------------
LOR73                    --------------------------------
LOR74                    --------------------------------
LOR75                    --------------------------------
LOR76                    --------------------------------
LOR77                    --------------------------------
LOR78                    --------------------------------
LOR79                    --------------------------------
LOR80                    --------------------------------
LOR81                    --------------------------------
LOR82                    --------------------------------
LOR83                    --------------------------------
LOR84                    --------------------------------
LOR85                    --------------------------------
LOR86                    --------------------------------
LOR87                    --------------------------------
LOR88                    --------------------------------
LOR89                    --------------------------------
LOR90                    --------------------------------
LOR91                    --------------------------------
LOR92                    --------------------------------
LOR93                    --------------------------------
LOR94                    --------------------------------
LOR95                    --------------------------------
LOR96                    --------------------------------
LOR97                    --------------------------------
LOR98                    --------------------------------
LOR99                    --------------------------------
LOR100                   --------------------------------
LOR101                   --------------------------------
LOR102                   --------------------------------
LOR103                   --------------------------------
LOR104                   --------------------------------
LOR105                   --------------------------------
LOR106                   --------------------------------
LOR107                   --------------------------------
LOR108                   --------------------------------
LOR109                   --------------------------------
LOR110                   --------------------------------
LOR111                   --------------------------------
Fugu_OR123-1             --------------------------------
Fugu_OR6765-1            --------------------------------
Fugu_OR4133-1            --------------------------------
Fugu_OR3630-2            --------------------------------
Fugu_OR8617-1            --------------------------------
```

```
Fugu_OR5510-1            --------------------------------
Fugu_OR117-1             --------------------------------
Fugu_OR2346-5            --------------------------------
Fugu_OR5287-2            --------------------------------
Fugu_OR142-1             --------------------------------
Fugu_OR1026-2            --------------------------------
Fugu_OR8298-1            --------------------------------
Fugu_OR6030-1            --------------------------------
Fugu_OR59-1              --------------------------------
Fugu_OR4208-3            --------------------------------
Fugu_OR4208-2            --------------------------------
Fugu_OR6818-1            --------------------------------
Fugu_OR7903-1            --------------------------------
Pufferfish_OR8981-2      --------------------------------
Pufferfish_SCAF8981      --------------------------------
Pufferfish_OR15134-1     --------------------------------
Pufferfish_OR14339-2     --------------------------------
Pufferfish_OR14328-1     --------------------------------
Pufferfish_OR14677-8     --------------------------------
Pufferfish_OR14536-2     --------------------------------
Pufferfish_OR14536-1     --------------------------------
Pufferfish_OR10960-2     --------------------------------
Pufferfish_OR10960-3     --------------------------------
Pufferfish_OR12434-1     --------------------------------
Medaka_ORUn.33           --------------------------------
Medaka_ORUn.2            --------------------------------
Medaka_OR21.18           --------------------------------
Medaka_OR21.19           --------------------------------
Medaka_OR21.20           --------------------------------
Medaka_OR13.14           --------------------------------
Medaka_OR13.12           --------------------------------
Medaka_ORUn.23           --------------------------------
Medaka_ORUn.21           --------------------------------
Medaka_OR14.15           --------------------------------
Medaka_OR14.13           --------------------------------
Medaka_OR14.4            --------------------------------
Medaka_OR14.6            --------------------------------
Medaka_OR13.4            --------------------------------
Medaka_OR13.5            --------------------------------
Medaka_OR13.6            --------------------------------
Medaka_OR13.7            --------------------------------
Medaka_ORUn.1            --------------------------------
Medaka_OR21.2            --------------------------------
Medaka_OR21.3            --------------------------------
Stickleback_OR1.1        --------------------------------
Stickleback_OR16.3       --------------------------------
Stickleback_OR1.12       --------------------------------
Stickleback_OR1.13       --------------------------------
Stickleback_ORUn.9       --------------------------------
Stickleback_OR7.1        --------------------------------
Stickleback_ORUn.6       --------------------------------
Stickleback_ORUn.8       --------------------------------
Stickleback_OR1.18       --------------------------------
Stickleback_OR16.10      --------------------------------
Stickleback_OR16.32      RYVHST--------------------------
Stickleback_OR16.1       --------------------------------
Stickleback_OR16.5       --------------------------------
Stickleback_ORUn.1       --------------------------------
Stickleback_ORUn.17      --------------------------------
Zebrafish_OR126-7        --------------------------------
Zebrafish_or126-3        --------------------------------
Zebrafish_OR123-1        --------------------------------
Zebrafish_OR125-1        --------------------------------
Zebrafish_OR127-1        --------------------------------
Zebrafish_OR128-10       --------------------------------
Zebrafish_OR116-2        --------------------------------
Zebrafish_zor6a          --------------------------------
Zebrafish_OR117-1        --------------------------------
Zebrafish_OR115-15       --------------------------------
Zebrafish_OR133-7        --------------------------------
Zebrafish_OR132-1        --------------------------------
Zebrafish_OR134-1        --------------------------------
Zebrafish_OR131-2        HN------------------------------
Zebrafish_OR131-1        --------------------------------
Goldfish_OR3             --------------------------------
Goldfish_OR2             --------------------------------
Rainbow trout OR500-1    --------------------------------
Cutthroat trout OR600-1  --------------------------------
Brown trout OR500-1      --------------------------------
Atlantic salmon SORB     --------------------------------
Medaka_mfOR2             --------------------------------
Medaka_mfOR1             --------------------------------
Medaka_OR_Y3             --------------------------------
Medaka_ORY1              --------------------------------
Frog_OR-52D1             --------------------------------
Frog_OR-52E8             --------------------------------
Frog_OR-52A1             --------------------------------
Frog_OR-52P1             --------------------------------
Frog_OR-52A5             --------------------------------
Frog_OR-2M5              --------------------------------
Frog_OR-2M2              --------------------------------
zebrafish_OR10.1         --------------------------------
zebrafish_OR10.14        --------------------------------
zebrafish_OR15.39        --------------------------------
Medaka_ORUn.6            --------------------------------
Medaka_ORUn.28           --------------------------------
Medaka_ORUn.27           --------------------------------
Stickleback_OR7.3        --------------------------------
Stickleback_OR7.4        --------------------------------
Stickleback_OR7.8        --------------------------------
Fugu_ORUn.83             --------------------------------
Fugu_ORUn.20             --------------------------------
Pufferfish_OR7.1         --------------------------------
Pufferfish_OR7.3         --------------------------------
zebrafish_OR10.29        --------------------------------
zebrafish_OR10.31        --------------------------------
zebrafish_OR15.58        --------------------------------
zebrafish_OR15.57        --------------------------------
Medaka_OR13.1            --------------------------------
Medaka_OR13.3            --------------------------------
Stickleback_OR1.25       --------------------------------
Fugu_ORUn.8              --------------------------------
Pufferfish_OR16.7        --------------------------------
Frog_OR669.1             --------------------------------
Amphioxus_105.1          STASNARECATVLGSERDVRAASNGGGPGSAE
Amphioxus_39.1           NDSQ----------------------------
```
