# Supplementary material for: Family structure and phylogenetic analysis of odorant receptor genes in the large yellow croaker (Larimichthys crocea)
Source: BMC Evol Biol. 2011 Aug 11;11:237. doi: 10.1186/1471-2148-11-237 (PMC3162931; doi:10.1186/1471-2148-11-237)
Supplement: Additional file 7 — The result of a multiple sequence alignment of 230 OR genes from different species after manual adjustment is included in this file. [file 1471-2148-11-237-S7.PDF]

```
                              10        20        30        40        50        60        70        80        90       100
                     ....|....|....|....|....|....|....|....|....|....|....|....|....|....|....|....|....|....|....|....|
LOR1                 RVTLFVLTLLCYCVIWLINLAIIVTIIMDKSLHEPMYIFLCNLCINGLYETAGFYPKFLIDLLSTFHVISYAGCLLQGFVLHSSACADFSILVLMAYDRY
LOR2                 RVTLFVLTLLCYCVIWLINLAIIVTIIMDKSLHEPMYIFLCNLCINGLYGTAGFYPKFLIDLLSTFHVISYAGCLLQGFVLHSSACADFSILVLMAYDRY
LOR3                 RVTLFVLTLLCYCVIWLINLAIIVTIIMDKCLHEPMYIFLRNLCINGLYGTAGFYPKFLIDLLSTFHVISYAGCLLQGFVLHSSACADFSILVLMAYDRY
LOR4                 RVTLFVLTLLCYCVIWLINLAIIVTIIMDKSLHEPMYIFLCNLRINGLYGTAGFYPKFLIDLLSTFHVISYAGCLLQGFVLHSSACADFSILVLMAYDRY
LOR5                 RVTLFVLTLLCYCVIWLINLAIIVTIIMDKSLHEPMYIFLCNLCINGLCGTAGFYPKFLIDLLSTFHVISYAGCLLQGFVLHSSACADFSILVLMAYDRY
LOR6                 RGTLFALTLLCYCVIWLVNLTIIVVIIMDKSLHEPMYIFLCNLCINGLYGTTGFYPKFLIDLLSTTHVISYAGCFLQGFVLHSSACADFSLLALMAFDRY
LOR7                 RGTLFALTLLCYCVIWLVNLTIIVVIIMDKSLHEPMYIFLCNLCINGLYGTTGFYPKFLIDLLSTTHVISYAGCFLQGFVLHSSACADFSLLALMAFDRY
LOR8                 RGTLFALTLLCYCVIWLVNLTIIVVIIMDKSLHEPMYIFLCNLCINGLYGTTGFYPKFLIDLLSTTHVISYAGCFLQGFVLHSSACADFSLLALMAFDRY
LOR9                 RGTLFALTLLCYCVIWLVNLTIIVVIIMDKSLHEPMYIFLCNLCINGLYGTTGFYPKFLIDLLSTTHVISYAGCFLQGFVLHSSACADFSLLALMAFDRY
LOR10                RGTLFALTLLCYCVIWLVNLTIIVVIIMDKSLHEPMYIFLCNLCINGLYGTTGFYPKFLIDLLSTTHVISYAGCFLQGFVLHSSACADFSLLALMAFDRY
LOR11                RYVYFVIMFTIYILIICSNSVIVYLIWVHPNLHEPMYIFIAALLINSVLFSTTIYPKLLIDFLSDKQIISHQACLVQFHMFYSLAGAEFLLLAAMAYDRY
LOR12                RYVYFVIMFTIYILIICSNSVIVYLIWVHPNLHEPMYIFIAALLINSVIFSTTIYPKLLIDFLSDKQIISHQACLVQFHMFYSLAGAEFLLLAAMAYDRY
LOR13                RYVYFVIMFTIYILIICSNSVIVYLIWVHPNLHEPMYIFIAALLINSVLFSTTIYPKLLIDFLSDKQSISHQACLVQFHMFYSLAGAEFLLLAAMAYDRY
LOR14                RYVYFVIMFTIYILIICSNSVIVYLIWVHPNLHEPMYIFIAALLINSVLFSTTIYPKLLIDFLSDKQIISHQACLVQFHMFYSLVGAEFLLLAAMAYDRY
LOR15                RYVYFVIMFTIYILIICSNSVIVYLIWVHPNLHEPMYIFIAALLINSVLFSTTIYPKLLIDFLSDKQIISHQACLVQFHMFYSLAGAEFLLLAAMAYDRY
LOR16                RYVYFVIMFTIYILIICSNSVIVYLIWVHPNLHEPMYIFIAALLINSVLFSTTIYPKLLIDFLSDKQIITYQACLVQFHMFYSLGGSEFLLLAAMAYDRY
LOR17                RYVYFVIMFTIYILIICSNSVIVYLIWVHPNLHEPMYIFIAALLINSVLFSTTIYPKLLIDFLSDKQIITYQACLVQFHMFYSLGGSEFLLLAAMAYDRY
LOR18                RYVYFVIMFTIYILIICSNSVIVYLIWVHPNLHEPMYIFIAALLINSVLFSTTIYPKLLIDFLSDKQIITYSACLLQSFFYYSLGCSEFLLLAAMSYDRY
LOR19                RYVYFVIMFTIYILIICSNSVIVYLIWVHPNLHEPMYIFIAALLVNSVPFSTTIYPKLLIDFLSDKQIITYQACLVQFHMFYSLGGSEFLLLAAMAHDRY
LOR20                RYVYFVIMFTIYILIICSNSVIVYLIWVHPNLHEPMYIFIAALLINSVLFSTTIYPKLLIDFLSDKQIITYQACLVQFHMFYSLGGSEFLLLAAMAYDRY
LOR21                RYVYFVIMFTIYILIICSNSVIVYLIWVHPNLHEPMYIFIAALLINSVLFSTTIYPKLLIDFLSDKQIITYQACLVQFHMFYSLGGSEFLLLAAMAYDRY
LOR22                RYVYFVIMFTIYILIICSNSVIVYLIWVHPNLHEPMYIFIAALLINSVLFSTTIYPKLLIDFLSDKQIITYLACLVQFHMFYSLGGSEFLLLAAMAYDRY
LOR23                RYVYFVIMFTIYILIICSNSVIVYLIWVHPNLHEPMYIFIAALLINSVLFSTTIYPKLLIDFLSDKQIISHQACLVQFHMFYSLAGAEFLLLAAMAYDRY
LOR24                RYLYFVIMFTIYILIICSNSVIVYLIWVHPNLHEPMYMFIAALLINSVLFSTTIYPKLLIDFLSDKQIITYQACLVQFHMFYSLGGSEFLLLAAMAYDRY
LOR25                RYLYFVIMFTIYILIICSNSVIVYLIWVHPNLHEPMYIFIAALLINSVLFSTTIYPKLLIDFLSDKQIITYQACLVQFHMFYSLGGSEFLLLAAMAYDRY
LOR26                RYLYFVIMFTIYILIICSNSVIVYLIWVHPNLHEPMYIFIAALLINSVLFSTTIYPKLLIDFLSDKQIITYQACLVQFHMFYSLGGSEFLLLAAMAYDRY
LOR27                ------------------------------------YVLIAALLINSVVYSTVFYPKLLIDFLSDKQIITYSACLFQWFIFYSLAGSEFLLLAAMSYDRY
LOR28                ------------------------------------YVLIAALLINSVVYSTVFYPKLLIDFLSDKQIITYSACLFQWFIFYSLAGSEFLLLAAMSYDRY
LOR29                ------------------------------------YVLIAALLINSVVYSTVFYPKLLIDFLSDKQIITYSACLFQWFIFYSLAGSEFLLLAVMSYDRY
LOR31                RYPTFVLCLLLYALIVLANFLIIVVISQEKTLHEPMYFFIMCLSINSLYGSAGFFFRFLRDLLYDTHLISRSACFTQTYVIYSYASYELTLLGIMAYDRY
LOR32                RYPTFVLCLLLYALIVLANFLIIVVISQEKTLHEPMYFFIMCLSINSLYGSAGFFFRFLRDLLYDTHLISRSACFTQTYVIYSYASYELTILGIMAYDRY
LOR33                RYPTFVLCLLLYALIVLANFLIIVVISQEKTLHEPMYFFIMCLSINSLYGSAGFFFRFLRDLLYDTHLISRSACFTQTYVIYSYASYELTLLGIMAYDRY
LOR34                RCPTFVLCLLLYALIVLANFLIIVVISREKTLHGPMYFFIMCLSINSLYGSAGFFFRFLRDLLYDTHLISRSACFTQTYVIYSYASYELTLLGIMAYDRY
LOR35                RYPTFVLCLLLYALIVLANFLIIVVISQEKTLHEPMYFFIMCLSINSLYGSAGFFFRFLRDLLYDTHLISRSACFTQTYVIYSYASYELTLLGIMAYDRY
LOR37                RRLLALPFTASYLSVLLGNSLLLYVICSVESLHSPMYLLICMLCVIDILVVTAIIPNMLLGLLFDWDETSLAGCLTQMFFTHFLSSLESTLLLVMALDRY
LOR38                RRPLALPFTASYLSVLLGNSLLLYVICSVESLHSPMYLLICMLCVIDILVVTAIIPNMLLGLLFDWDEISLAGCLTQMFFTHFLSSLESTLLLVMALDRY
LOR39                RRLLALPFTASYLSVLLGNSLLLYVICSVESLHSPMYLLICMLCVIDILVVTAIIPNMLLGLLFDWDEISLAGCLTQMFFTHFLSSLESTLLLVMALDRY
LOR40                RRLLALPFTASYLSVLLGNSLLLYVICSVESLHSPMYLLICMLCVIDILVVTAIIPNMLLGLLFDWDEISLAGCLTQMFFTHFLSSLESTLLLVMALDRY
LOR41                RRLLALPFTASYLSVLLGNSLLLYVICSVESLHSPMYLLICMLCVIDILVVTAIIPNMLLGLLFDWDEISLAGCLTQMFFTHFLSSLESTLLLVMALDRY
LOR42                RRLLALPFTASYLSVLLGNSLLLYVICSVESLHSPMYLLICMLCVIDILVVTAIIPNMLLGLLFDWDEISLAGCLTQMFFTHFLSSLESTLLLVMALDRY
LOR43                KYPIFFLGLIIYLFGIFCNLTLLSLIILQKNLHKPMYFILFSLPLNDLVGITAMLPKVLSDIVTNK--VYYPLCVLQAFLLHMYGGGILFILAAMSFDRY
LOR44                KYPIFFLGLIIYLFGIFCNLTLLSLIILQKNLHKPMYFILFSLPLNDLVGITAMLPKVLSDIVTNK--VYYPLCVLQAFLLHMYGGGILFILAAMSFDRY
LOR45                KYPIFFLGLIIYLFGIFCNLTLLSLIILQKNLHKPMYFILFSLPLNDLVGITAMLPKVLSDIVTNK--VYYPLCVLQAFLLHMYGGGILFILAAMSFDRY
LOR46                KYPIFFLGLIIYLFGIFCNLTLLSLIILQKNLHKPMYFILFSLPLNDLVGITAMLPKVLSDIVTNK--VYYPLCVLQAFLLHMYGGGILFILAAMSFDRY
LOR47                KYPIFFLGLIIYLFGIFCNLTLLSLIILQKNLHKPMYFILFSLPLNDLVGITAMLPKVLSDIVTNK--VYYPLCVLQAFLLHMYGGGILFILAAMSFDRY
LOR48                IYPVFLSILFSYLFIMVTNVGIAALVFIDKNLHQPMYLLFCNLPINDILGNSILLPRLLVDILLSERLISYYECVVQAFTTHMFGTTSHTVLMIMAFDRY
LOR49                IYPVFLSILFSYLFIMVTNVGIAALVFIDKNLHQPMYLLFCNLPINDILGNSILLPRLLVDILLSERLISYYECVVQAFTTHMFGTTSHTVLMIMAFDRY
LOR50                IYPVFLSILFSYLFIMVTNVGIAALVFIDKNLHQPMYLLFRNLPINDILGNSILLPRLLVDILLSERLISYYECVVQAFTTHMFGTTSHTVLMIMAFDRY
LOR51                IYPVFLSILFSYLFIMVTNVGIAALVFIDKNLHQPMYLLFRNLLINDILGNSIKLPRLLLDMLRSERLISYYECVVQAFTTHMYGTASHTVLMIMAFDRY
LOR52                IYPVFLSILFSYLFIMVTNVGIAALVFIDKNLHQPMYLLFCNLPINDILGNSILLPRLLVDILLSERLISYYGCVVQAFLTQFFGTTSHTVLMIMAFDRY
LOR53                IYPVFLSILFSYLFIMVTNVGIAALVFIDKNLHQPMYLLFCNLPINDILGNSILLPRLLVDILLSERLISYYACVVQAFITQFFGTTSHTVLMIMAFDRF
LOR54                IYPVYLFFFFSYLFIMVTNVGITVLIFIDKNLHQPMYLLFCNLPVNDILGNSILLPRLLLDMLRSERLISYYECVVQAFTSHMFGTTSHTVLMIMAFDRY
LOR55                IYPVYLFFFFSYLFIMVTNVGITVLIFIDKNLHQPMYLLFCNLPVNDILGNSILLPRLLLDMLRSERLISYYECVVQAFTSHMFGTTSHTVLMIMAFDRY
LOR56                IYPVYLFFFFSYLFIMVTNVGITVPIFIDKNLHQPMYLLFCNLPVNDILGNSILLPRLLLDMLRSERLISYYECVVQAFTSHMFGTTSHTVLMIMAFDRY
LOR57                IYPVYLFFFLSYLFIMVTNVGITVLIFIDKNLHQPMYLLFCNLPVNDILGNSILLPRLLLDMLRSERLISYYECVVQAFTSHMFGTTSHTVLMIMAFDRY
LOR58                IYPVYLFFFFSYLFIMVTNVGITVLIFIDKNPHQPMYLLFCNLPVNDILGNSILLPRLLLDMLRSERLISYYECVVQAFTSHMFGTTSHTVLMIMAFDRY
LOR59                IYPVYLFFFFSYLFIMITNVGITVLIFIDKNLHQPMYLLFCNPPVNDILGNSILLPRLLLDMLRSERLISYYECVVQAFTSHMFGTTSHTVLMITAFDRY
LOR60                IYPVYLFFFFSYLFIMITNVGITVLIFIDKNLHQPMYLLFCNLPVNDILGNSILLPRLLLDMLRSERLISYYECVVQAFTSHMFGTTSHTVLMIMAFDRY
LOR61                IYPVYLFFFFSYLFIMVTNVGITVLIFIDKNLHQPMYLLFCNLPVNDILGNSIMLPRLLLDMLRSERLISYYECVVQAFISHMFGTASHTVLMIMAFDRY
LOR62                IYPVYLFFFFSYLFIMVTNVGITVLIFIDKNLHQPMYFLFRNLLINDILGNSIKLPRLLLDMLRSERLISYYECVVQAFSTHMFGTTSHTVLMIMAFDRY
LOR63                IYPVYLFFFFSYLFIMVTNVGITVLIFIDKNLHQPMYFLFRNLLINDILGNSIKLPRLLLDMLRSERLISYYECVVQAFTTHMYGTASHTVLMIMAFDRY
LOR64                IYPVYLFFLFSYLFIMVTNVGITVLIFIDKNLHQPMYLLFRNLLINDILGNSIKLPRLLLDMLRSERLISYYECVVQAFTTHMYGTASHTVLMIMAFDRY
LOR65                IYPVYLFFFFSYLFIMVTNVGITVLIFIDKNLHQPMYLLFRNLLINDILGNSIKLPRLLLDMLRSERLISYYECVVQAFTTHMYGTASHTVLMIMAFDRY
LOR66                KYPIFIFLLLSYMFILIANVGIVILIWKERSLHQPMYLLFCNLSINDVMGNSLLVPRVLADILVSDRLIHYYECVMQAFTTHMFSTNAHTVLMIMAFDRY
LOR67                KYPIFIFLLLSYMFILIANVGIVILIWKERSLHQPMYLLFCNLSINDVMGNSLLVPRVLADILVSNRLIHYYECVMQAFTTHMFSTNAHTVLMIMAFDRY
LOR68                KYPIFIFLLLSYMFILIANVGIVILIWKERSLHQPMYLLFCNLSINDVMGNSLLVPRVLADILVSDRLIHYYECVMQAFTTHMFGTNAHTVLMIMAFDRY
LOR69                KYPIFIFLLLSYMFILIANVGIVILIWKERSLHQPMYLLFCNLSINDVMGNSLLVPRVLADILVSNRLIHYYECVMQAFTTHMFSTNAHTVLMIMAFDRY
LOR70                KYPIFIFLLLSYMFILIANVGIVILIWKERSLHQPMYLLFCNLSINDVMGNSLLVPRVLADILVSERLIHYYECVMQAFTTHMFSTNAHTVLMIMAFDRY
LOR71                KYPIFIFLLLSYMFILIANVGIVILIWKERSLHQPMYLLFCNLSINDVMGNSLLVPRVLADILVSERLIHYYECVMQAFTTHMFSTNAHTVLMIMAFDRY
LOR72                RVVFSTLIGMPCCLFLFINGIMLFTLRSKAFRETCRYILLYNLLFSDTVL---LALSQLMYIMACRILPTYPVCGVLTMLNRLTNEISPLTLVVMCMERY
LOR73                RVVFSMLIGMPCCLFLFINGIMLFTLRSKTFRETCRYILLYNLLFADTVM---LGLSQLLYILSSRIFPVYPVCGVLIMLTSLTNEISPLTLVVMCLERY
LOR74                RVVFSTLIGMPCCLFLFINGIMLFTLRSKAFRETCRYILLYNLLFSDTVL---LALSQLMYIMACRILPTYPVCGVLTMLNRLTNEISPLTLVVMCMERY
LOR75                RVVFSMLIEMPCCLFLFINGIMLFTLRSKAFRETSRYILLYNLLFADTVL---LALSQLLYIMAFRIFTTYPVCGVLAMLNKLTNEISPLTLVVMSLERY
LOR76                RVVFSTLIGMPCCLFLFINGIMLFTLRSKAFRETCRYILLYNLLFSDTVL---LALSQLMYIMACRILPTYPVCGVLTMLNRLTNEISPLTLVVMCMERY
LOR77                RVVFSTLIGMPCCLFLFINGIMLFTLRSKAFRETCRYILLYNLLFSDTVL---LALSQLMYIMACRILPTYPVCGVLTMLNRLTNEISPLTLVVMCMERY
LOR78                RILFTTLTGMPCCLFLLINGIMLFTLRSKAFRETSRYILLYNLLFADTFM---LAVSQLLYIMACRLITTYPVCGVVTLLTILTNEISPLTLVVMCLERY
LOR79                RILFTTLTGMPCCLFLLINGIMLFTLRSKAFRETSRYILLYNLLFADTFM---LALSQLLYIMACRLITTYPVCGVITLLTILTNETSPLTLVVMCLERY
LOR80                RILFTTLTGMPCCLFLLINGIMLFTLRSKAFRETSRYILLYNLLFADTFM---LALSQLLYIMACRLITTYPVCGVITLLTILTNEISPLTLVVMCLERY
LOR81                RILFTTLTGMPCCLFLLINGIMLFTLRSKAFRETSRYILLYNLLFADAFM---LALSQLLYIMACRLITTYPVCGVITLLTILTNEISPLTLVVMCLERY
LOR82                RILFTTLTGMPCCLFLLINGIMLFTLRSKAFRETSRYILLYNLLFADAFM---LALSQLLYIMACRLITTYPVCGVITLLTILTNEISPLTLVVMCLERY
LOR83                AVTKNVIVVFIGISITYINASLIHTFSKHQFYTNPRYILFIHLVINDMIQ---VSLTIILFVISTIYRINVSVCCVFILLALFTTENTPLNLACMAAECY
LOR84                AVTKNVIVVFIGISITYINASLIHTFSKHQFYTNPRYILFIHLVINDMIQ---VSLTIILFVISTIYRINVSVCCVFILLALFTTENTPLNLACMAAECY
LOR85                AVTKNVIVVLIGISITYINASLIHTFSKHQFYTNPRYILFIHLVTNDMIQ---VSLTIILFVISTIYRINVSVCCVFILLALFTTENTPLNLACMAAECY
LOR86                AVTKNVIVVLIGISITYINASLIHTFSKHQFYTNPRYILFIHLVTNDMIQ---VSLTIILFVISTIYRINVSVCCVFILLALFTTENTPLNLACMAVECY
LOR87                AVTKNVIVVFIGISITYINASLIHTFSKHQFYTNPRYILFIHLVINDMIQ---VSLTIILFVISTIYRINVSVCCVFILLALFTTENTPLNLACMAVECY
LOR88                AVTKNVIVVFIGISITYINASLIHTFSKHQFYTNPRYILFIHLVINDMIQ---VSLTIILFVISTIYRINVSVCCVFILLALFTTENTPLNLACMAVECY
LOR89                AVTKNVIVVFIGISITYINASLIHTFSKHQFYTNPRYILFIHLVINDMIQ---VSLTIILFVISTIYRINVSVCCVFILLALFTTENTPLNLACMAAECY
LOR90                AVTKNVIVVFIGISITYINASLIHTFSKHQFYTNPRYILFIHLVINDMIQ---VSLTIILFVISTIYRINVSVCCVFILLALFTTENTPLNLACTAAECY
LOR91                AVTKNVIVVFIGISITYINASLIHTFSKHQFYTNPRYILFIHLVINDMIQ---VSLTIILFVISTIYRINVSVCCVFILLALFTTENTPLNLACMAVECY
LOR92                AVTKNVIVVFIGISITYINASLIHTFSKHQFYTNPRYILFIHLVINDMIQ---VSLTIILFVISTIYRINVSVCCVFILLALFTTENTPLNLACMAVECY
LOR93                MNGLVIIVQILVVIFLCVNMLLIVTFFSKEFYTTMRYILFAVTLLSDSLM---LFVSDILLILSYRVTMQFGLCIIFYILSVICIFVTPVTLTAMALERY
LOR94                MNDLVIIVQILVVIFLCVNMLLIVTFFSKEFYTTMRYILFAVTLLSDSLM---LFVSDILLILSYRVTMQFGLCIIFYILSVICIFVTPVTLTAMALERY
LOR95                MNDLVIIVQILVVIFLCVNMLLIVTFFSKEFYTTMRYILFAVTLLSDSLM---LFVSDILLILSYRVTTQFGLCIIFYILSVICIFVTPVTLTAMALERY
LOR96                MNDLVIIVQILVVIFLCVNMLLIVTFFSKEFYTTMRYILFAVTLLSDSLM---LFVSDILLILSYRVTMQFGLCIIFYILSVICIFVTPVTLTAMALERY
LOR97                MNDLVIIVQILVVIFLCVNMLLIVTFFSKEFYTTMRYILFAVTLLSDSLM---LFVSDILLILSYRVTMQFGLCIIFYILSVICIFVTPVTLTAMALERY
LOR98                MNDLVIIVQILVVIFLCVNMLLIVTFFSKEFYTTMRYILFAVTLLSDSLM---LFVSDILLILSYRVTMQFGLCIIFYILSVICIFVTPVTLTAMALERY
LOR99                -------------------------------------------------------------------------------------------------ERY
LOR100               -------------------------------------------------------------------------------------------------ERY
LOR101               -------------------------------------------------------------------------------------------------ERY
LOR102               -------------------------------------------------------------------------------------------------ERY
LOR103               -------------------------------------------------------------------------------------------------ERY
LOR104               -------------------------------------------------------------------------------------------------ERY
LOR105               -------------------------------------------------------------------------------------------------ERY
LOR106               -------------------------------------------------------------------------------------------------ERY
LOR107               -------------------------------------------------------------------------------------------------ERY
LOR108               -------------------------------------------------------------------------------------------------ERY
LOR109               -------------------------------------------------------------------------------------------------ERY
LOR110               -------------------------------------------------------------------------------------------------ERY
LOR111               -------------------------------------------------------------------------------------------------ERY
Fugu_OR123-1         KITLFVFTFLYYCVILQVNLTVILTIIVDKSLHEPMYIFLCNLCINGLYGTTGFYPKFLIDILSTSHVISYVGCLLQAFVLHSSACADFSILVLMAYDRY
Fugu_OR6765-1        KFTLFALTFVCYCVIVQVNVTLILTIIMDKGLHEPMYIFLCNLCINSLYGTAGFYPKFLIDILSTSHVISYAGCLVQSLVVHSSVCADFSILVLMAYDRY
Fugu_OR4133-1        RFTLFALTFVCYCVIVQVNVTLILTIIMDKSLHEPMYIFLCNLCINSLYGTAGFYPKFLIDILSTSHIISYAGCLVQSLVVNSSACAVFSFLALMAYDRY
Fugu_OR3630-2        RYLYFVIMLTAYILIICSNSTIVFLICFHRNLHEPMYVLIAALLINSVLYSTAIYPKLLIDFLSEEQTITYSVCLFQWFMFYSLGGSEFLLLAVMAYDRY
Fugu_OR8617-1        RYLYFVIMLTAYILIICSNSTIVFLICFHRNLHEPMYVLIAALLINSVLYSTAIYPKLLIDFLSEEQTITYSACLFQYFMFYSLGASEFLLLAVMAYDRY
Fugu_OR5510-1        RYLYFVIMLTAYILIICSNSTIVFLICFHRNLHEPMYVLIAALLINSVLYSTAIYPKLLIDFLSEEQTITYSVCLFQWFMFYSLGGSEILLLAVMAYDRY
Fugu_OR117-1         RYLAFVLCFILYASIVFANVIIIVVISRENALHEPMYIFIMCLSVNSLYGSAGFFFRFLKDLLSSTHLISRWGCFAQIYVIYTYASYELTLLSIMAYDRY
Fugu_OR2346-5        RYLAFVFCLLLYSFMISANFVIILVVSRERTLHEPMYIFIALLSVNSLYGSTGFFPRFLMDLLLDTHFMSRSACFTQIYVFYTYISFEMTILGIIAYDRH
Fugu_OR5287-2        RRLLALPFSVSYLSVLLGNCLLVYVICSVEHLHSPMYLLICSLCVVDVLAATAILPSMLLSLLFDVDTISLASCLTQMFFTHFLSSLESTLLLAMALDRY
Fugu_OR142-1         KYPIFFLGVTIYCFGVFCNMTLLTLIILQRNLHKPMYFILFSLPLNDLIGITAMLPKVLSDIVMNR--VHYGLCVFQAFILHMYGGGILFILAAMSFDRY
Fugu_OR1026-2        VYPVFFFFLVSYIFIMLSNIGIVALIFIDSSLHQPMYLLFCNLPFNDVLGNSILIPRLLMDLLHSERLITYYECVVQAFTTHMFGTTSHTVLMIMAFDRY
Fugu_OR8298-1        VYPVFFFFLVSYIFIIVANIAIVALIFIDSSLHQPMYLLFCNLPFNDVLGNSILIPHLLMDLLRSERLITYYECVAQAFTTHMFGTTSHTVLIIMAFDRY
Fugu_OR6030-1        RMMFSSLTTVPCCVFLYINGIMLFSLRSKTFCETSRYILLFNLLFADSIQ---MVLGQALYILACLIKMTYPVCGILTALGVLTSNISPLTLVVMSLERY
Fugu_OR59-1          RMMFSSLTTVPCCAFLYINGIMLFSLRSKTFCETSRYILLFNLLFADSIQ---MVFSQVLYILACLIKMSYPLCGIVNVVGVITSDISPLTLVVMSLERY
Fugu_OR4208-3        LITR-----------CGLTCCVCPRCPVPQFYTNPRYILFFHLVVNDMIQ---VMLTVMLFIISVLYKINVSFCGVFILLALITTENTPLNLACMALECY
Fugu_OR4208-2        ------------------------------FYMNPRYILFIHLVINDLIQ---VNVTIMLFVISTIYRINICMCWVFMLLAVVATENSPLNLACMALECY
Fugu_OR6818-1        INSRVILVQVLVLVFLCINFLLIVTFSMRHFYTTMRYILFAVTLLSDSVL---LLLTDVMLILSFAVTIEMWVCVTLYVVLALYTFVTPVTLTVMTLERY
Fugu_OR7903-1        IFERIIITQLIVSIFVSDNLFLIATVFSNQFLSTMRYILFVITLVSDSVF---LIIANLLLILHTYFTIEVSLCIVFYLIVCLSVFVTPVTLTAMTLERY
Pufferfish_OR8981-2  RFILFALTFVFYCVIVQVNVTLIVIIIIDKSLHEPMFIFICNLCINALYRTAGFYPKFLMDILSDSHVISYAGCFVQSLVVTSSTCVDFSFLVLMAYDRY
Pufferfish_SCAF8981  RFTLFALTFVCYCVIVQVNVTLILIIIIDKSLHEPMFIFLCNLCINALYGTAGFYPKFLMDILSTSHVISYAGCLVQSLVVTSSTCNDFFFLVIMGYDRY
```

```
Pufferfish_OR15134-1          RYLYFVLMLTAYLLILCTNSTVVYLICLHRDLHQPMYVLIAALLLNSVIYSSTIYPKLLQDFLSEEQTITYSACLFQYFAFYSLAGSEFLLLAVMAYDRY
Pufferfish_OR14339-2          RYLAFVLFFILYLFIVFANIIIIVVTARENTLHEPMYIFIMCLAVNSLYGSAGFFLRFLRDLLSSSHLFSRSECFTQIYVIYTYASYELTFLSVMAYDRY
Pufferfish_OR14328-1          RYLAFVLFFILYLFIVFANVIIIVVTARENALHEPMYIFIMCLAVNSLYGSAGFFLRFLRDLLSSSHLISRPECFVQIYVIYTYGSFELSFLSVMAYDRY
Pufferfish_OR14677-8          RRLLVLPFSATYLLVLLGNSLLVYVICNMEHLHSPMYLLICSLCVVDILAATAIFPSMLLSLLFDMDKISLASCLTQMFFTHFLSSLESTLLLAMALDRY
Pufferfish_OR14536-2          RLMLSFLTTVPCCIFFYINTIMLFTLRSKTFCETSRYVLLFNLLFADTLQ---MVLCQVLFILACWIKMSYPVCGFLNVIGVLTSDISPLTLVVMSLERY
Pufferfish_OR14536-1          RLMFSFLTTVPCCIFFYINAIMLFTLRSKTFCETSRYVLLFNLLLADTIQ---MVTSQLLYILACLIRLTYPVCGFLNVIGVLTSDISPLTLVVMSLERY
Pufferfish_OR10960-2          AVTKNVIVVVLSISINYVNVGLVQTFHRHQFYSNPRYILFFHLVVNDMIQ---VTLTVLLFVISVLYKIEVSVCCVFILLALFTTENTPLNLAAMALECY
Pufferfish_OR10960-3          AVTKNAVVLVLGLFINYINRGLIHTFCKHQFYTNSRYVLFIHLVINDMLH---VNLTMVLFLISTVYRVNVCVCWLLLLPALITTENTPLNLAAMALECY
Pufferfish_OR12434-1          LFERVLITQILAFMFISTNLVLLATVFSKPFYSTMRYILFTITLVSDSIT---LIISDLLLLLNFSVPLQVSLCIICYLIVLFCGFITPVTLTAMTLERY
Medaka_ORUn.33                RRLLALPFFLSLLSVLLGNSALILVIRREERLRGPMHLLICALCLVDILVVVAILPNMLLGLTLDHSRISLAACLTQMFFTHFLSSVESTLLLTMALDRL
Medaka_ORUn.2                 RPLLFLPFSFAFLVSLLANGLLVYVVFSQRSLHQPMSVLIAGMAVVEVCLPVFFVPNMLLSFLFGWRDISLIGCLTQMHFIHTFGAFQTTFLVWMALDRY
Medaka_OR21.18                RLTLFCLSLLCYCLIWMVNVFLILVIIFDQSLREPMYIILCSLCINGLYGTAGFYPKFLSDLLSDRYVISYIGCFLQIYVIYSYAKVDFSIIALMAYDRY
Medaka_OR21.19                RPTLFVLSLLCYCLILLVNVSLVIIIILDKSLHEPMYILLCAFCINGLYGTTGFYPKFLWDLLSPVHMISYYGCLIQAQVMYSFACSDLSILSVMAYDRY
Medaka_OR21.20                RPTLFVLSLLCYCLILLVNVSLVIIIILDKSLHEPMYILLCAFCINGLYGTTGFYPKFLWDLLSPVHMISYYGCLIQVQVMYSFACSDLSILSVMAYDRY
Medaka_OR13.14                ISKDIIIVQVLIAIFLSINFFLISTFFRKNFHTSMRYILFAVTLLSDCLF---LMLANVTLIMTFGFTIQMWLCLIIYLLTSVYSYVTPVTLTAMTLERY
Medaka_OR13.12                INDRIIIVQVSIAVFLSINFFLISTFFRRDFYNTMRYILFAETLLSDCLF---LILTDSLLILSFGCTMQMWLCLFIYIILSVYSFVTPITLTAMTLERY
Medaka_ORUn.23                VIILSTLSTVPACVFLVINGIMLFTLRSKPFRETCRFVLLFNLLLADTLQ---LAQSQLHLLFSCRVQLAYSLCIALNMIVNLTTGISPLTLVLMPLERY
Medaka_ORUn.21                TLIFSIISTVTSCVFLVINGIMLFTLRSKPFRETCRFILLYNLLLADTLQ---LAQSQLHLLLSCRVKVAYSLCIALNMIVNLATGISPLTLVLMPLERY
Medaka_OR14.15                TLIFSIISTVTSCVFLVINGIMLFTLRSKPFRETCRFILLYNLLLADTLQ---LAQSQLHLLLSCRVKVAYSLCIALNMIVNLATGISPLTLVLMPLERY
Medaka_OR14.13                KYPVFFLFLFIYLLIMLTNVGILVLIIIDRNLHQPMYLLFCNLSVSDVIGNTYMIPRLLSDILKSERLISYYECVLQAFTSHMFGTTSHTVLMIMAFDRY
Medaka_OR14.4                 TEYAFFLFFFSYIVIMILNLGILAIIAIDKSLHQPMYLLFCNLPVSDIVGCTHILPRLLSDMLVSERLISYYECVVQAFTTHMFGTTSHTVLMVMAFDRY
Medaka_OR14.6                 TEYAFFLFFFSYIVIMILNLGILAIIAIDKSLHQPMYLLFCNLPVSDIVGCTHILPRLLSDMLVSERLISYYECVVQAFTTHMFGTTSHTVLMVMAFDRY
Medaka_OR13.4                 VVPAFLFATLSYMVILFCNLTLILTIVLNKSLHQPMHLILLNLPINDLIGSSALFPQLIKEILTRR--IQYSACVAQAFFIHVYAAGTVFHLSAMAYDRF
Medaka_OR13.5                 IYPAFVFGTLFYLIIVFCNLLVLTTIAVSKKLHKPMFILLFSLPVSDIVGATAFFPHLVFSILTRL--ISSEACITQAFFVHFYGTGNLMFLTAMAYDRY
Medaka_OR13.6                 IYPSFVFGTLLYLITVFCNLLVLLTIAVSKKLHKPMFILLLNLPISDIVGATALFPHFLFSIITRL--ISSEACITQAFFIHIYGTGNLIILSAMAFDRY
Medaka_OR13.7                 KYPIILLGIAIYLFGMSCNLTLLTLIILKKNLHKPMYFILFSLPLNDLIGITAMMPKVLSDIVTND--VYYPLCVLQAFLLHMYGGAVLFILAAMSFDRY
Medaka_ORUn.1                 RQVSFGLALTAYLFTLFVNLTLIGTVLLEKHLHQPMYFFLCNLCINGICGASSFYPKLLHDLLADAHVVSYAGCVAQMFVGYSYIFCEFTSLTVMAYDRY
Medaka_OR21.2                 RYFYFFMMLPVYIFIVCSNCTIISLIVIHKNLHEPMYLFIAGLLINSVLFSTNIYPKLLTDLLATKQVISYQACLFQVFVFYSFSCSEFLLLAAMSYDRY
Medaka_OR21.3                 RYFYFFMMLTFYILIVCSNCTIISLIVIHKNLHEPMYIFIASLLINSVLFSTNIYPKLLADFLSNEQVISFQACLLQRIFFYSFGGSEFFLLAVMSYDRY
Stickleback_OR1.1             RQLLALPLCASYLVVLLGNGLLVLVVLRTESLKGPMYVLIAALCAVDLLAATAIVPKMLLGLLFDWNRISLASCLAQMFSTHFLSSLESTLLLAMALDRY
Stickleback_OR16.3            RVALFSVMLLCYFFIVLVNVVLVVTIILDKNMHEPMYILLCSLCMNGLYGTTGFYPKILWDLLSPVHVISYSGCLVQAFVIHAFVSNDMSILAVMSYDRY
Stickleback_OR1.12            FNYRVVIVQVLVSVFLFINFWLIRTFFVRDFYTVMRYILFATTLLSDSIF---LIITDILLILSIKITMQVWLCVIIYIISSLCIFVTPVTLTAMTLERY
Stickleback_OR1.13            INYRLVVVQVLVSVFLFINFLLIRTFFVRDFYTVMRYILFATTLLSDSLF---LIITDILLIFSIKITMQMWLCVIIFIVSSLYTFVTPVTLTAMTLERY
Stickleback_ORUn.9            SVAFSMLTTVPCCVFLLVNAAMLLTLRSRPFRETPRYVLLLNLLCADTAQ---MALSQLLYLLASRAPLTYPACGVLVMLADLTNAVSPLTLVAMSLERY
Stickleback_OR7.1             SPVRVLLSMLPCILFLYINGVMLFALLSKPLLESSRYILFANLLIADSLQ---LLSAMLMYIFATMIKLSSFLCALVVLVARVTVKTSPLNLAMMSLERY
Stickleback_ORUn.6            FYPVFIFLLFSYLFIIIANVGIAAVILLDKSLHQPMYLLFCNLPLNDLLGNSILLPRLLLDMLRSELFISYYECVVQAFTTHVFGTTSNTVLMIMAFDRY
Stickleback_ORUn.8            FYPAFIFLLLSYIFIMLMNVGIAAVILLDKSLHQPMYLLFCNLPLNDLLGNSILLPRLLLDMLRSELFISYYECVVQAFTTHMSGTTAHTVLMIMAFDRY
Stickleback_OR1.18            KYPMFLFGIAIYLFSVFCNLMLLSLIILKRNLHKPMYFILFSLPLNDLIGVSAMLPKVLSDLVTNT--VYYPLCVLQAFLLHMYGGGILFILAAMSFDRY
Stickleback_OR16.10           RYLAFVLCLLLYAFIIFSNLVIIVLISREKTLHEPMYIFIMCLSINSLYGSAGFFFRFLRDLLSDTNLISRTACFAQIYVIYTYASYELTLLGIMAYDRY
Stickleback_OR16.32           RGFLFALTLLCYSVIWLVNLTIIVTVIADKKLHEPMYIFLCNLCINGLYGTAGFYPKFLMDLLSPTHVISYAGCLLQGFVLHSSACADFSLLVLMAYDRY
Stickleback_OR16.1            RYLAFVLCLLLYAFIIFSNLVIIVLISREITLHEPMYIFIMCLSINSLYGSAGFFFRFLRDLLSDTHLISRTACFAQIYVIYTYASYEMTLLGIMAYDRY
Stickleback_OR16.5            RYLAFVLCLLLYAFIIFSNLVIIVLISREITLHEPMYIFIMCLSINSLYGSAGFFFRFLRDLLSDTHLISRTACFAQIYVIYTYASYELTLLGIMAYDRY
Stickleback_ORUn.1            KYIYFVMMLTMYILIICTNSTIVYLIVTHNNLHQPMYIFIAALLGNSVVFSTAIYPKLLMDFLSEEQVISYPACLFQCFLYYSLDGSEFLLLSAMAYDRY
Stickleback_ORUn.17           RYLYFVVMFTVYILIISSNVTIVCIIMIHKNLHEPMYIFIAALLINSVLFSTAIYPKLLIDFLSEKQIISYPACLLQWFSSHWLAVSDFFLLSIMAYDRY
Zebrafish_OR126-7             RYVLFSLTVLFYPLMVFCNVTVIVAIISHKKLHEPMYVFICNLCANALFGTAGFYPKFLYDLLSQDHVISYAGCMIQTFVIYSYALCDVSTLTVMAYDRY
Zebrafish_or126-3             RYVLLSFTALFYPVVIFCNVTVILTIFLHKKLHEPMYIFLCNLCINGIFGTSGFYPKFMYDLLAPEHVISYAGCLIQIFVIYSSVFCDFSTLTVMAYDRY
Zebrafish_OR123-1             RITIFSFALPGYFLTIFVNTLLILIIVLEKVLHQPMYIFLCNLCINGLYGATGFYPPLLYYLLSESNVISLKECAIQSFAIFTYAIGEFTNLSVMAFDRY
Zebrafish_OR125-1             RHIYFICFLVLYLLILSINICLVMVIIIEKVLHEPMYIFLCHLCINGVYGATGFYPKILSDLILDSYVISSHMCALQTYVIYSSLLSEITILTVMSYDRY
Zebrafish_OR127-1             RYVILTLTLTVYLAIIVCNAIILFVVFKERSLHEPMYILISCLSVNDLYGSAGFFPRLIADLLSDTNAISRPACFVQIFTIYSYAMSEYTILTLMAYDRY
Zebrafish_OR128-10            RYALFSLGIILYSAILLFNFLVILAIFLERTLHQPMYILISCLSFNSVFGTAAFFPRLLTDLLSDTHLVPREACLLQAFVIYSYASNENTILMLMAFDRY
Zebrafish_OR116-2             KYPIFIFGIFIYMFGAFCNLTLLLLIFLTESLHKPMYFILFSLPLNDFVGITSMLPKVLSDILTNK--TYYPLCVLQGFLLHMYGGGVLFVLAAMAFDRY
Zebrafish_zor6a               RRFLFLPFFLTYLLLLVGNSLLLFVIKSTESLHSPMYIFVSALAVVDIIVPTAIIPAMLLGFLFDLNEITLSGCLTQMFVTHFFSSVESTILLAMALDRF
Zebrafish_OR117-1             KYPVFFAGILVYMFSVICNTTILGLIISQKSLHKPMFYILFSLPLIDLIGITAGLPRVLVDIVTND--VYYPTCVLQAFLIHLYGGGTLFLLAAMSLDRY
Zebrafish_OR115-15            TYPVFVFFLLFYVAIMVLNIGIFIVITTHRSLHEPMYMLFCNLPINDILGNSIMVPRLLMDILKSQRYISYVECVVQAFSTHTYGTTSHTILIIMAIDRY
Zebrafish_OR133-7             APTKAFLFVTPCVIFLYVNGVMLFTLRKKAFQETSRYILFGHMLWVDTLN---LFMSVVLYICASRILIMKNVCLLLLIAATALFQVSTLNLALMSLERY
Zebrafish_OR132-1             TSTKTAVAVLTSLFFFSVNCVMISVLKSKRFYETPRYILFGHMLMNDSVL---LLVTTVMYTLACFLPIHKSICTLLVLISYCTFFNAPLTLALMSLERY
Zebrafish_OR134-1             AFMKNFIVVLVWFLLSYINGSVVATFFRHQFYEDPRYILFIHMVINDAVQ---LTVTIALFLVSILYTISVGVCCFFILVAVFTTRSTPVNLAGMAIERY
Zebrafish_OR131-2             LKEKSLTVQVLVGILLYVNGLMIFTFLKKEFRDT-RYILFAQTLFVDSAL---MLFADLTLVGSYELFIHIISCYIFCTVMALLSICSPVTLVAMCLERY
Zebrafish_OR131-1             LKEKALLVQVLVGILLYVNGLMIFTFLKKEFRDT-RYILFAQTLFVDSSF---MIFADLTLLCSYQLIIHNISCYVFCTVMSVLSICSPVTLVAMCLERY
Goldfish_OR3                  RFMFFSLTALYYPFIVLCNVIIIYTVISHKKLHEPMYVFICNLCMNGLFGTAGFYPKFMYDLLSQYHLISYAGCLVQIFVIYSSVLCDFSTLTVMAYDRY
Goldfish_OR2                  YGAVAALLLFAYVCILVGNGIFIALFTLEKRLHKPMYNIILNLVVSDLLFSTTTLPKIISRYWF-DGSISFTGCFIQMYFVHYFGSVSSVILAIMAFDRY
Rainbow_trout_OR500-1         KSVYFILPLITYLLIITVNLPLIITIIQEKGLHEPMYIFLCSLCVNGLYGTAGFYPKFLLDLQSDVQVISYGGCFTQAYVIYTSVMCELSTLTVMSYDRY
Cutthroat_trout_OR600-1       AYPAFILFLIIYIFTMVANIGLISLISMERSLHQPMYILFCNLPLNDIVGATVMIPRLLWDILTPERYITYTECVIQAFYTHVYATTSHTVLMIMAFDRY
Brown_trout_OR500-1           KSVYFILSLITYLLIITVNLTLIKTIIQEKGLHEPMYIFLCSLCVNGLYGTTGFYPKLLLDLQSDVHVISYGGCFTQAYVIYTSVMCEISTLTVMSYDRY
Atlantic_salmon_SORB          MYPVFIILFFSYVIIMFTNVGIVVLISMEKSLHQPMYILSCNLPFNDIIGNSITFPRLLADILVSERYITYSECVVQAFCTQMFGTTSHTILMIMAFDRY
Medaka_mfOR2                  RYLYFLIVFTVFILILLCNCTIIFIIVVQKSLHEPMYIFIAALPTNSVMFSMAVYPKLLVDILSKRQIITYYACFFQHGVYYCLGASEFLLLTAMSFDRY
Medaka_mfOR1                  RCLYFLIVFTVFILILFCNCTIIFIIVVQKSLHEPMYIFIAALLTNSVMFSMAVYPKLLVDILSKRQIITYYACLLQYVVYYCLGASEFLLLTAMSFDRY
Medaka_OR_Y3                  KYYIFLIFFFSYIFIMVTNVGILVLIIIDRNLHQPMYLLFCNLPINDVMGNSIMIPRLLSVILKSERLISYYECVLQAFTAHMFGTTSNTVLMIMAFDRY
Medaka_ORY1                   TEYAFFLFFFSYIVIMILNLGILAIIAIDKSLHQPMYLLFCNLPVSDIVGCTHILPRLLSDMLVSERLISYYECVVQAFTAQMFGSTSHTVLMVMAFDRY
Frog_OR-52D1                  KYFYSTLVLLCFMIIVVSNSAVISAIAMHRSLQEPMFIFIAFLCINGLYGSVIFFPFLFVNLLSKTQVISYVGCIIQVFSIHTYISCEVTILAVMALDRY
Frog_OR-52E8                  KYLYGAIVFLIFLMIVASSSAVIGTIILHRSLHEPMYMFIAALCMNGLYGGICFFPALFVNLLSQVQTVSYIGCLIQVFGIHSYYGCEITVLAVMAFDRY
Frog_OR-52A1                  RPLLAIPFFSVYLVILSGNSLLICLICIKKTLHSPMYLLISVLFAINITSCTAILPKFLLDLLFQLNQVSLTGCLLQMFVIYFMAVCESNVMLLMSLDRY
Frog_OR-52P1                  RPLLAIPFFSIYLVILSGNSLLICLICIKKTLHSPMYFLISLLFAINITSITTTLPKFLLDLLFHLNQVSLTGCLLQMFVIYFVTACESTVLLLMSLDRY
Frog_OR-52A5                  RTTFFVSVLVCLFFFAYLVTIILTVFFTKPLRENSRYVLFAHMLINDVVY---LVMTLYLAIITLPMRLPAHVCYAIVMLSSASYKVTAYNLGVMALERY
Frog_OR-2M5                   RMTFLILSFLCFGFFIYLVAIILYIFLSNAVRESARYVLFAHILVNDLLY---LSVGFYLLVSYLVF-IPVPVCYIIVTLASCTFWVTPYNLALMSLERY
Frog_OR-2M2                   FIPVSITMFLVYCVSLVANSSVIILVLQKEQLHQPMYIIIRNLALSDLLFDTITLPKIIAKYWF-AGSITFYGCFFQLFCVNSLGSLYSFIIMLMAIDRY
zebrafish_OR10.1              SNVYVMFLTVLFVITVICNVFLISIIVYDQRLHVPKFMAVGNLAAVDLILSTALVPGMIKTYIA-DNFVPFNLCLLQMYIYYTCTCLESFSLCILSYDRF
zebrafish_OR10.14             MRYYYIFLSFVYIISFLGNSCLMFVIIIDRNLHTAKYIAVFNLSLSDICESTAVIPQLLDTFLF-NQLIPYRLCLYNMFSVFVSISTQSLTLAVLSFDRL
zebrafish_OR15.39             KNYLILGLAIIYIITLLGNLVLLSVVLMNSSLQNPKYLAVCNLAVVDISINSVIIPQMVPVFVF-QNYISFGACFSQMFFMHFFGDMESFSLALLAYDRL
Medaka_ORUn.6                 VNVFFIFLAIVYVVTVLFNGLLIIVVVINPSLHTPKFLAVINLAVIDLCFTACSIPGMIKIFLF-DNFVPFNQCLVQMFFYYVFAALESYALMILAYDRL
Medaka_ORUn.28                MNYYYIFLFFVFFISVLGNTAVMTVIYLDHNLRTAKYVAVFNLAFVDLLGNTALVPKVIDTFLF-HYYISYNECLTYFFFCYMCLSMQALNLVALCYDRL
Medaka_ORUn.27                VKYYYIFLFFVYIVSVVGNTAVMAVIYMDNSLRTPKYIAVFNLAFVDLLGNSALVPKVLDIFLF-HNVIPYNDCMTFLFFCYTCLCMQSFNLVALSYDRL
Stickleback_OR7.3             ISVYIIFLAFVYAVTLVFNILLISLIARDHCLHTPKFMAVFNLAVIDVILSTCTIPSMIKVFLF-DNFVPFNLCLVQMYFYYAILSMESYALAILAYDRF
Stickleback_OR7.4             ISVYFIFLSFVYVVTVMFNSLVIYVIAFNHCLHSPKFLAVINLAVIDIVLNTTTIPGMVKIFLV-DNFIPFNLCLVQMFSYYCFASMESFALAILAYDRL
Stickleback_OR7.8             MKHYYVFLCFVYIVSVLGNTVVMAVIYLDHNLRTPKYIAVFNLAFVDLLGSSTLMPKLLDIFLF-HRYISYNDCLTFLFFSYTFLCMQSFNLLALSYDRL
Fugu_ORUn.83                  IKFYYVFLFFVYIVSVLGNSVVMAVICLDHNLRTPKYVAVFNLAFVDLFGNSALVPKLLDIFLF-HRHIPYNDCLTFLFFCYTCLSMQALNLVALAYDRL
Fugu_ORUn.20                  IRFYFIFLAFVYVVTVFFNCLLIYVIVSNRCLHTPKFLAVVNLAVIDIVLNSTTIPSMIKTFLL-DNFFPFNICLLQMFVYYSSISLESFALAILAYDRL
Pufferfish_OR7.1              IRFYYIFLFFVYIVSVLGNSSVMAVICLDRSLRTPKYVAVFNLAFVDLFGNSALVPKLLDIFLF-HRRIPYNDCLTFLFFCYTCLSMQALNLVALAYDRL
Pufferfish_OR7.3              IRFYFIFLAFVYVVTLLFNCLLIYVIASHPCLHTPKFLAVVNLAVIDIVLSSSTIPSMIRTFLL-ENFFPFNLCLLQMFTYYSFATLESLALAILAYDRL
zebrafish_OR10.29             YGLVSAIFFVIYITTVAGNMFFLVLFVTTESLRKPMYIILASLALSDMCFSTVALPKIISKYWF-AGATPFHACFFQMELIHYFGSLNSLIMMIMALDRY
zebrafish_OR10.31             YSLTAALLFCIYIAVITGNSLIVVLFVIERSLHKPMYIIMLSLSVSDICFCTVALPKVISRYWF-DGFISFYVCLFQRQLIHYFGTLNSLIMMLMALDRY
zebrafish_OR15.58             HGSVGALLFILYLVLATGNIFIIVFVMIEKSIQKPTYIIFCNLAMADLALGTTTYPRVIAKYWM-DKIISFSGCFTQMFFIHFLGATNSFLMALMALDRF
zebrafish_OR15.57             YGAVGFLLFFVYLTLAGGNIFIIAFIAYEKSLQKPTYLIFCNLAVCDIAIGTVTLPKAIAKYFF-SSEITFLSCFVQMFFVHYLGSINSFLMLLMAVDRF
Medaka_OR13.1                 HGLASFVFLAVYLTILFGNSFILAVIIFERVLHKPSYLIFAHLAMTDLIFGTLTLPKVIARYLW-DMITPFTTCFAQMFFVHSLGAIHSFILLVMALDRF
Medaka_OR13.3                 YAPVSALFFFIYLTTATGSVFILALVICEKSLQKPSYLVFCHLALNDLTFSTFTIPKIIAKYWF-DSDISFNSCFTQMFMVHYLGSVMSFLLLVMALDRF
Stickleback_OR1.25            YRPVSALLFFFYLAIAIGNIFIFTFVVCEKSLQKPTYLLFCHLALNDLTFGTLTLPKIISKYWF-DGFISFYGCFTQMFFVHYSGSVTSFMLLAMALDRF
Fugu_ORUn.8                   YRPVSALFLIIYLVIAFGNIFILTFVMCQKSLQKPMYLVFCHLALIDLTFSTVTIPKIISKYWF-GGDISFYGCFAQMFFVHYLGSVTSFILLVMALDRF
Pufferfish_OR16.7             YGPVSALFLIIYLAIVLGNVFILTFVMCHKSLQKPMYLVFCHLALIDLMFGTVTLPKIISKYWF-NGDISFHGCFTQMFFVHYLGSVTSFILLVMALDRF
Frog_OR669.1                  DVPVSIGLFLAYIISLFANATVILLIAFSRHLHQPMYKIMVNLAVSDLLFDTITLPKIIAKYWF-AGSIPYCVCLLQLFCVHFLGSFDAYIIMLMALDRY
Amphioxus_105.1               SQGLQTAYLVISLIVIVAANLLIILLVCRKYLRQPRYFLRCHLAVNDILHALVIIPRYLDLIMQSNSRFYGISCSDVRIFTSAVALSKYGIYLLMAIDIY
Amphioxus_39.1                SRGLQTLYLVTSLVVAVGCSSLVIFLVWKTSLQKPRHFLRCNLALDDIIFTGSMIPLEIYGLFV-DDSVVRRLCGVQNMLIPTFTASMFGAYLLMATELY

                                      110       120       130       140       150       160       170       180       190       200
                              ....|....|....|....|....|....|....|....|....|....|....|....|....|....|....|....|....|....|....|....|
LOR1                          VAICRPLVYHSVMTTQRVCVFIFFAWLIPLYLVFMSSITTARSRLCG-SHIPKIYCINFLVGKLACTTS--IANVIIPAFNYTFYFLHVMFIAWSYMYLI
LOR2                          VAICRPLVYHSVMTTQRVCVFIFFAWLIPLYLVFMSSITTARSRLCG-SHIPKIYCINFLVGKLACTTS--IANVIIPAFNYTFYFLHVMFIAWSYMYLI
LOR3                          VAICRPLVYHSVMTTQRVCASIFFAWLIPLYLVFMGSITTARSRLCG-SHIPKIYCINFLVGKLACTTS--IANVIIPAFNYTFYFLHVMFIAWSYMYLI
LOR4                          VAICRPLVYHSVMTTQRVCASIFFAWLIPLYLVFMGSITTARSRLCG-SHIPKIYCINFLVGKLACTTS--IANVIIPAFNYTFYFLHVMFIAWSYMYLI
LOR5                          VAICRPLVYHSVMTTQRVCASIFFAWLTPLYLVFMGSITTARSRLCG-SHIPKIYCINFLVGKLACTTS--IANVIIPAFNYTFYFLHVMFIAWSYMYLI
LOR6                          VAICRPLVYHSVMTKQRVCLFVFFAWLIPFYLMFLSSITTLISKLCG-SHIPKIYCVNTLIGNLACSAS--IAKIIIPAFNYTFYFGHVISVVWSYIYLI
LOR7                          VAICRPLVYHSVMTKQRVCLFVFFAWLIPFYLMFLSSITTLISKLCG-SHIPKIYCVNTLIGNLACSAS--IAKIIIPAFNYTFYFGHVIFVVWSYIYLI
LOR8                          VAICRPLVYHSVMTKQRVCLFVFFAWLIPFYLMFLSSITTLISKLCG-SHIPKICCVNTLIGNLACSAS--IAKIIIPAFNYTFYFGHVIFVVWSYIYLI
LOR9                          VAICRPLVYHSVMTKQRVCLFVFFAWLIPFYLMFLSSITTLISKLCG-SHIPKIYCVNTLIGNLACSAS--IAKIIIPAFNYTFYFGHVIFVVWSYIYLI
LOR10                         VAICRPLAYHSVMTKQRVCLFVFFAWLIPFYLIFLSSITTLISKLCG-SHIPKIHCVNTLIGNLARSAS--IAKIIIPAFNYTFYFGHVIFVIWSYIYLI
LOR11                         VSICKPLQYPTIMSKTTVSIFLVSAWLLPACQLVLPTAMSAKEKLCR-FILNGVFCNN-TIYKLQCVISQ-ARFVRDMVILLNVALVPVLFILFTYTRIF
LOR12                         VSICKPLQYPTIMSKTTVSIFLVSAWLLPACQLVLPTAMSAKEKLCR-FILNGVFCNN-TIYKLQCVISQ-ARFVRDMVILLNVALVPVLFILFTYTRIF
LOR13                         VSICKPLQYPTIMSKTTVSIFLVSAWLLPACQLVLPTAMSAKEKLCR-FILNGVFCNN-TIYKLQCVISQ-ARFVRDMVILLNVALVPVLFILFTYTRIF
LOR14                         VSICKPLQYPTIMSKTTVSIFLVSAWLLPACQLVLPTAMSAKEKLCR-FILNGVFCNN-TIYKLQCVISQ-ARFVRDMVILLNVALVPVLFILFTYTRIF
LOR15                         VSICKPLQYPTIMSKTTVSIFLVSAWLLPACQLVLPTAMSAKEKLCR-FILNGVFCNN-TIYKLQCVISQ-ARFVRDMVILLNVALVPVLFILFTYTRIF
LOR16                         VSICKPLQYPTIMSKTTVSIFLVSAWLLPACQLVLPTAMSAKEKLCR-FILNGVFCNN-TIYKLQCVISQ-ARFVRDMVILLNVALVPVLFILFTYTRIF
LOR17                         VSICKPLQYPTIMSKTTVKIFLVSAWLLPACQLVLPTAMSAKEKLCR-FILNGVFCNN-TIYKLQCVISQ-ARFVRDMVILLNVALVPVLFILFTYTRIF
LOR18                         VSICKPLQYSTIMSKTTVKIFLVSAWLLPACQLVLPTAMSAKEKLCR-FILNGVFCNN-TIYKLQCVISQ-ARCVRDMVILLNVALVPVLFILFTYTRIF
LOR19                         VSICKPLQYPTIMGKTTVSIFLVSAWLLPACQLVLPTAMSAKEKLCR-FILDGVFCNN-TIYKLQCVISQ-ARFVRDMVILLNVALVPVLFILFTYTRIF
LOR20                         VSICKPLQYPTIMGKTTVSIFLVSAWLLPACQLVLPTAMSAKEKLCR-FILNGVFCNN-TIYKLQCVISQ-ARFVRDMVILLNVALVPVLFILFTYTRIF
LOR21                         VSICKPLQYPTIMSKTTVSIFLVSAWLLPACQLVLPTAMSAKEKLCR-FILNGVFCNN-TIYKLQCVISQ-ARFVRDMVILLNVALVPVLFMLFTYTRIF
LOR22                         VSICKPLQYPTIMSKTTVSIFLVSAWLLPACQLVLPTAMSAKERLCR-FILNGVFCNN-TIYKLQCVISQ-ARFVRDMVILLNVALVPVLFILFTYTRIF
LOR23                         VSICKPLQYPTIMSKTTVSIFLVSAWLLPACQLVLPTAMSAKEKLCR-FILNGVFCNN-TIYKLQCVISQ-ARFVRDMVILLNVALVPVLFILFSYTRIF
LOR24                         VSICKPLQYPTIMSKTTISIFLVLAWILPASQVAVPAGLSAHKNLCS-FVLKGFFCNN-TVYKLQCVISR-EQIIHDMVILLNVALVPVLFILFTYTRIF
LOR25                         VSICKPLQYPTIMSKTTISIFLVLAWILPASQVAVPAGLSAHKNLCS-FVLKGFFCNN-TVYKLQCVISR-EQIIHDMVILLNVALVPVLFILFTYTRIF
LOR26                         VSICKPLQYPTIMSKTTISIFLVLAWILPASQVAVPAGLSAHKNLCS-FVLKGFFCNN-TVYKLQCVISR-EQIIHDMVILLNVALVPVLFILFTYTRIF
LOR27                         VSICKPLQYPTIMRKKTVIMFLVLAWLVPFCKIAGPIVQNVNKKLCK-FIFKGIICNS-TVHKLHCVEPR-IEKIYGLIVFVNLLIIPVLFILFTYTRIF
```

LOR28	VSICKPLQYPTIMRKKTVIMFLVVAWLVPFCKIAGPIVQNVNKKLCK-FIFKGIICNS-TVHKLHCVEPR-IEKIYGLIVFVNLLIIPVLFILFTYTRIF
LOR29	VSICKPLQYPTIMRKKTVIMFLVLAWLVPFCKIAGPIVQNVNKKLCK-FIFKGIICNS-TVHKLHCVEPR-IEKIYGLIVFVNLLIIPVLFILFTYTRIF
LOR31	VAVCQPLHYHNKMTSKLVSKLVTFAWTYPAFSVGTCVYLASRLPLCG-NKIPKVFCANWPIVKLSC-ISTVINNLVGMLVSTTTVFLPLAFVLYTYARIF
LOR32	VAVCQPLHYHNKMTSKLVSKLVTFAWIYPAFSVGTCVYLASRLPLCG-NKIPKVFCANWPIVKLSC-ISTVINNLVGMLVSTTTVFLPLAFVLYTYARIF
LOR33	VAVCQPLHYHNKMTSKLVSKLVTFAWIYPAFSVGTCVYLASRLPLCG-NKMPRVFCANWPIVKLSC-ISTVINNLVGMLVSTTTVFLPLAFVLYTYARIF
LOR34	VAVCQPLHYHNKMTSKLVSKLVTFAWIYLAFSVGTCVYLASRLPLCG-NKIPKVFCANWPIAKLSC-ISTVINNLVGMLVSTTTVFLPLAFVLYTYARIF
LOR35	VAVCQPLHYHNKMTSKLVSKLVTFAWIYPAFSVGTCVYLASRLPLCG-NKIPKVFCANWPIVKLSC-ISTVINNLVGMLVSTTTVFLPLAFVLYTYARIF
LOR37	VAICHPLRYTEIVDSSLFMKLLLFTLLRSGSIMGTLVGLADSLRFCSSNLIQHCYCDHMALVSLACDSTE-KNTAAGLAVIICFVGVDIPLIFFSYMKIL
LOR38	VAICHPLRYTEIVDSSLFMKLLLFTLLRSGSIMGTLVGLADSLRFCSSNLIQHCYCDHMALVSLACDSTE-KNTAAGLAVIICFVGVDIPLVFFSYMKIL
LOR39	VAICHPLRYTEIVDSSLFMKLLLFTLLRSGSIMGTLVGLADSLRFCSSNLIQHCYCDHMALVSLACDSTE-KNTAAGLAVIICFVGVDIPLILFSYMKIL
LOR40	VAICHPLRYTEIVDSSLFMKLLLFTLLRSGSIMGTLVGLADSLRFCSSNLIQHCYCDHMALVSLACDSTE-KNTAAGLAVIICFVGVDIPLIFFSYMKIL
LOR41	VAICHPLRYTEIVDSSLFMKLLLFTLLRSGSIMGTLVGLADSLRFCSSNLIQHCYCDHMALVSLACDSTE-KNTAAGLAVIICFVGVDIPLIFFSYMKIL
LOR42	VAICHPLRYTEIVDSSLFMKLLLFTLLRSGSIMGTLVGLADSLRFCSSNLIQHCYCDHMALVSLACDSTE-KDTAAGLAVIICFVGVDIPLIFFSYMKIL
LOR43	AAICMPLRYSTIMTPRIVVCIISLVWGLDFVLIVSLFSLQTRLPRCK-SVIMNVFCDNPSLLKLTCG-NRAVNNIIGLFNTAVMQAVSVSLQAFSYVKIL
LOR44	VAICMPLRYSTIMTPRIVVCIISLVWGLDFVLIVSLFSLQTRLPRCK-SVIMNVFCDNPSLLKLTCG-NRAVNNIIGLFNTAVMQAVSVSLQAFSYVKIL
LOR45	AAICMPLRYSTIMTPRIVVCIISLVWGLDFVLIVSLFSLQTRLPRCK-SVIMNVFCDNPSLLKLTCG-NRAVNNIIGLFNTAVMQAVSVSLRAFSCVKIL
LOR46	VAICMPLRYSTIMTPRIVVRIISLVWGLDFVLIVSLFSLQTRLPRCK-SVIMNVFCDNPSLLKLTCG-NRAVNNIIGLFNTAVMQAVSVSLRAFSYVKIL
LOR47	VAICMPLRYSTITTPRIVVCIISLVWGLDFVLIVSLFSLQTRLPRCK-SVIMNVFCDNPSLLKLTCG-NRAVNNIIGLFNTAVMQAVSVSLQAFSYVRIL
LOR48	VAICKPLHYTAIMTNKMVMKLTVSAWGVAFVLVGILLGLTIRLNRCR-SIIMNPYCDNASLFKLSCD-SVFINNVYGITFTVVLLTSSIGTMVLTYTNIT
LOR49	VAICKPLHYTAIMTNKMVMKLTVSAWGVAFVSVGILLGLTIRLNRCR-SIIMNPYCDNASLFKLSCD-SVLINNVYGITFTVVLLTSSIGTMVLTYTNIT
LOR50	VAICKPLHYTAIMTNKMVMKLTVSAWGVAFVLVGILLGLTIRLNRCR-SIIMNPYCDNASLFKLSCD-SVFINNVYGITFTVVLLTSSIGTMVLTYTNIT
LOR51	VAICNPLRYSTIMNDKMVMKLTVSAWGVAFVLVGILLGLTIRLNRCR-TLIMNAHCDNAGLFKLSCE-NVFINNVYGLTFTVVLFTGSIGSMVLTYTKIT
LOR52	VAICYPLRYSAIMSNKMVTKLSVSAWGSAFVLVGILISLTVRLNRCR-TMIMNPFCDNASLFKLSCE-SVFINNVYGLTFTVALYVVSIGSIVLTYTKIA
LOR53	VAICYPLRYSTIMTNKMVMKLTVFAWGSAFFFVGILISLSVRLNRCR-TMIMSPFCDNPSLFKLSCE-SVFINNVYGLTFTVVLLTASIGSMVFTYTKIT
LOR54	VAICKPLHYTAIMTNKMVMKLTVSAWGVAFVLVGILLGLTIRLNRCR-TIIMNPYCDNASLFKLSCD-SVFINNVYGLTFTVVLLTSSIGTMVLTYTNIT
LOR55	VAICKPLHYTAIMTNKMVMKLTVSAWGVAFVLVGILLGLTIRLNRCR-TIIMNPCCDNASLFKLSCD-SVFINNVYGLTFTVVLLTSSIGTMVLTYTNIT
LOR56	VAICKPLHYTAIMTNKMVMELTVSAWGVAFVLVGILLGLTIRLNRCR-TIIMNPYCDNASLFKLSCD-SVFINNVYGLTFTVVLLTSSIGTMVLTYTNIT
LOR57	VAICKPLHYTAIMTNKMVMKLTVSAWGVAFVLVGILLGLTIRLNRCR-TIIMNPYCDNASLFKLSCD-SVFINNVYGLTFTVVLLTSSIGTMVLTYTNIT
LOR58	VAICKPLHYTAIMTNKMVMKLTVSAWGVAFVLVGILLGLTVRLNRCR-TIIMNPYCDNASLFKLSCD-SVFINNVYGLTFTVVLLTSSIGTMVLTYTNIT
LOR59	VAICKPLHYTAIMTNKMVMKLTVSAWGVAFVLVGILLGLTIRLNRCR-TIIMNPYCDNASLFKLSCD-SVFINNVYGLTFTVVLLTSSIGTMVLTYTNIT
LOR60	VAICKPLHYTAIMTNKMVMKLTVSAWGVAFVLVGILLGLTIRLNRCR-TIIMNPYRDNASLFKLSCD-SVFINNVYGLTFTVVLLTSSIGTMVLTYTNIT
LOR61	VAICKPLHYTAIMTNKMVMKLTVSAWGVSFVLVGILLGLTIRLNRCR-TMIMNPYCDNPSLFKLSCE-NVFINNVYGLTFTVVLLTSSIGSIVLTYTKIT
LOR62	VAICYPLRYSAIMSNKMVMKLTVSAWGVAFVLVGILLGLTLRLNRCR-TLITNPFCDNASLFKLSCE-SVSINDVYGLTFTVVLFTGSIGSMVLTYTKIA
LOR63	VAICNPLRYSTIMNDKMVMKLTVSAWGVAFVLVGILLGLTIRLNRCR-TLIMNAHCDNAGLFKLSCE-NVFINNVYGLTFTVVLFTGSIGSMVLTYTKIT
LOR64	VAICNPLRYSTIMNDKMVMKLTVSAWGVAFVLVGILLGLTIRLNRCR-TMIMNAHCDNAGLFKLSCE-NVFINNVYGLTFTVVLFTGSIGSMVLTYTKIT
LOR65	VSICNPLRYSTIMNDKMVMKLTVSAWGVAFVLVGILLGLTIRLNRCR-TMIMNAHCDNAGLFKLSCE-DVFINNVYGLTFTVVLFTGSIGSMVLTYTKIT
LOR66	VAICNPLRYSIIMNDEMVLKLTVSAWGVAFVLVGILLGLTIRLNRCR-TLITNPYCDNASLFKLSCE-SVFINNVYGLTFTVVLLSSSIGSIILTYFKIA
LOR67	VAICNPLRYSIIMNDKMVLKLTVSAWGVAFVLVGILLGLTIRLNRCR-TLITNPYCDNASLFKLSCE-SVFINNVYGLTFTVVLLSSSIGSIILTYFKIA
LOR68	VAICNPLHYSIIMNDKMVIKLTVSAWGVAFVLVAILLSLTIRLNRCR-TLITNPYCDNASLFKLSCE-SVFINNVYGLTFTVVLLSSSIGSIILTYFKIA
LOR69	VAICNPLHYSIIMNDKMVIKLTVSAWGVAFVLVGILLGLTIRLNRCR-TLITNPYCDNASLFKLSCE-SVFINNVYGLTFTVVLLSSSIGSIILTYFKIA
LOR70	VAICNPLRYSAIMSNKMVMKLTVSAWGVSFVLVGILLGLTIRLNRCR-TMIMNPYCDNPSLFKLSCE-NVFINNVYGLTFTVVLLTSSIGSIVLTYTKIT
LOR71	VAICNPLRYSIIMNDKMVLKLTVSAWGVAFVLVGILLGLTIRLNRCR-TLITNPYCDNASLFKLSCE-SVFINNVYGLTFTVVLLSSSIGSIILTYFKIA
LOR72	VAVCYPLRHATIITVRNTAIAIIVVWAFSSLNVLIRVLLLLNFPFDQLSLQMTDFCSDIAMLLGPMSDIYDIAYTGFVFVSAGVAVTCSYIGVMVAARSA
LOR73	VAVCYPLRHATIITIRNTAVAIIVVWAFSSLNVLIRVLLLLKFPFEELSLQMTDVCSDIAMFLGPMSDIYDKAYTGFVFLSAGVAVTCSYVGVMVAARSA
LOR74	VAVCYPLRHATIITVRNTAIAIIAVWAFSSLNVLVQVLLLLTFPFEELSLQMTDICSYEAMFLVPMSDLYDKAYTGFVFLSAGVAVTCSYIGVMVAARSA
LOR75	VAVCYPLRHATIITVRNTAVAIIVVWAFSSLNVLIQVILLLKFPFEKLSLQMTDICAYVAMFLMPMSDIYDKAYTGFVFVSAGVTVTCSYIGVMVAARSA
LOR76	VAVCYPPRHATIITIRNTAVAIIVVWAFSSLNVLIRVLLLLKFPFEELSLQMTDVCSDIAMFLGPMSDIYDKAYTGFVFLSAGVAVTCSYVGVMVAARSA
LOR77	VAVCYPLRHATIITVRNTAVAIIVVWAFSSLNVLIQVILLLKFPFEKLSLQMTDICAYVAMFLMPMSDIYDKAYTGFVFVSAGVTVTCSYIGVMVAARSA
LOR78	VAVCYPLRHATIITVRNTAGAIVVVWAFSSLNVLIRVLLLLDFPFEDLSLQMTEICSLIAVSIGPMSDIYDKAYTGFVFVSAGVTVTCSYIGVMVAATSA
LOR79	VAVCYPLRHATIITVRNTAGAIVVVWAFSSLNVLIRVLLLLDFPFEDLSLQMTEICSLIAVSIGPMSDIYDKAYTGFVFVSAGVTVTCSYIGVMVAATSA
LOR80	VAVCYPLRHATIITVRNTAGAIVVVWAFSSLNVLIRVLLLLDFPFEDLSLQMTEICSLIAVSIGPMSDIYDKAYTGFVFVSAGVTVTCSYIGVMVAATSA
LOR81	VAVCYPLRHATIITVRNTAGAIVVVWAFSSLNVLIRVLLLLDFPFEDLSLQMTEICSLIAVSIGPMSDIYDKAYTGFVFVSAGVTVTCSYIGVMVAATSA
LOR82	VAVCYPPRHATIITIRNTGLAIVVIWAICSLNVLTAVFMLLNFSFEDQNLQMKDYCRREIMFLNPVSDLYDKVYTYILFCSAGVAVSFSYIGVMVAARSA
LOR83	IAVCMPLRHVEICTIKRTLMLIGLIWTTTMLSVMSDLFITLTT--QPLFFHSQVFCLRQTVFPSPLIIKKRDITYSVFLVIVWGTILYTYFRILFTAKTA
LOR84	IAVCMPLRHVEICTIKRTSMLIGLIWTTTMLSVMSDLFITLTT--RPLFFHSQVFCLRQTVFPSPLIIKKRDITYSVFLVIVWGTILYTYFRILFTAKTA
LOR85	IAVCMPLRHVEICTIKRTLMLIGLIWTTTMLSVMSDLFITLTT--QPLFFHSQVFCLRQTVFPSPLIIKKRDITYSVFLVIVWGTILYTYFRILFAAKTA
LOR86	IAVCIPLRYVQICTIKRTLMLIALTWTTTMLSVMSDLFITLTT--QPLFFHSQVFCLRRNVFPSPLIPKKRDITYSVFLVIVWGTILYTYFRILFAAKTA
LOR87	IAVCMPLRHVQICTIQRTLILIGLIWTVTSFNVLSDLFITLAT--QPLFFHSRVFCLRKTVFPSPLIIKKRDITYSVFLVIVWGTILYTYFRILFAAKTA
LOR88	IAVCMPLRHVQICTIQRTLILIGFIWTVTSFNVLSDLFITLAT--QPLFFHSQMSCIRRNVFPSPLIPKKRDITYSVFLGIVWCTIFYTYFRILFAAKTA
LOR89	IAVCMPLRHVEICTIKRTLMLIGLIWTTTMLSVMSDLFITLTT--QPLFFHSQVFCLRQTVFPGPLIIKKRDITYSVFLVIVWGTILYTYFRILFTAKTA
LOR90	IAVCMPLRHVEICTIKRTLMLIGLIWTTTMLSVMSDLFITLTT--QPLFFHSQVFCLRQTVFPSPLIIKKRDITYSVFLVIVWGTILYTYFRILFTAKTA
LOR91	IAICMPLRHVQICTVRRTLMLIGLIWATSMLSVLPDLFITLAT--QPLFFYTRVFCLRETAFSNPHIIKKRDITYIVYLVIVWFVIFYTYFRILFTAKTA
LOR92	IAVCMPLRHVEICTIKRTLMLIGLIWTTTMLSVISDLFITFAT--EPLFFHSQVFCLRETAFPSPLIVEKRVITYSVFLVIVWCTIFYTYFRILFTAKTA
LOR93	VAICMPLRHAQLCSTRSTMHCILIIHSLSSVPCIVILSVFCAS--ASKLYTQYKICPMEIFVSLRWQNNIKTAIYQLYFLIMSITIILSYVKIMKVAKAA
LOR94	VAICMPLRHAQLCSTRSTMHCILIIHSLSSVPCIVILSVFCAS--ASKLYTQYKICSMEIFVSLRWQNNIKTAIYQLYFLIMSITIILSYVKIMKVAKAA
LOR95	VAICMPLRHAQLCSTRSTMHCILIIHSLSSVPCIVILSVFCAS--ASKLYTQYKICSMEIFVSLRWQNNIKTAIYQLYFLIMSITVILSYVKIMKVAKAA
LOR96	VVICMPLRHAQLCSTRSTMHCILIIHSLSSVPYIVILSVFCAS--ASKLYTQYKICSMEIFVSLRWQNNIKTAIYQLYFLIMSITIILSYVKIMKVAKAA
LOR97	VAICMPLRHAQLCSTRSTMHCILIIHSLSSVPCIVILSVFCAS--ASRLYTQYKICSMEIFVSLRWQNNIKTAIYQLYFLIMSITIILSYVKIMKVAKAA
LOR98	VAICMPLRHAQLCSTRSTMHCILIIHSLSSVPCIVILSVFCAS--ASKLYTQYKICSMEIFVSLRWQNNIKTAIYQLYFLIMSITIILSYVKIMKVAKAA
LOR99	VAICMPLRHAELCSTRSTMHCIFIIHSLSSVPCIVILSTFFAS--ASFLYKQPRLCEMELLMLYRWQDHVRSAVHQFYFLIMAIIILFSYVKIMKVAKAA
LOR100	VAICMPMRHAELCSTRSTMHCILIIHGLSSVPCIVILSTAFAT--ASLFYTQPMLCSVETFMLYRWQDHVRSAVHQFYFLIMAIIILFSYVKIMKVAKAA
LOR101	VAICMPLRHAELCSTRSTMYCIIIIHGLSSVPCIVVLSTFFAS--ASLLYKQYSSCSVEILILHRWQGHVRSAVHQFYFLIMVIIILFSYVKIMKVAKAA
LOR102	VAICMPLRHAELCSTRSTMYCILIIHGLSSVPCIVVLSTFFAS--ASFLYKQYSSCSVEILILHRWQGHVRSAVHQLYFLIMVIIILFSYVKIMKVAKAA
LOR103	VAICMPLRHAELCSTRSTMYCIFIIHGLSSVPCIVVLSTFFAS--ASFLYKQYSSCSVEILILHRWQGHVRSAVHQFYFLIMVIIILFSYVKIMKVAKAA
LOR104	VAICMPLRHAELCSTRSTMYCILIIHGLSSVPCIVVLSTFFAS--ASFLYKQYSSCSVEILILHRWQGHVRSAVHQFYFLIMVIIILFSYVKIMKVAKAA
LOR105	VAICMPLRHAELCSTRSTMHCILIIHGFSSVPCIVVLSTFFAS--ASFLYKQYSSCSVEIFMLHRWQDHVRSAVQQFYFLIMVIIIVFAYVKIMKVAKAA
LOR106	VAICMPLRHAELCSTRSTMYCILIIHGLSSVPCIVVLSTFFAS--ASFLYKQYRLCAIKLFMLYRWQDHVISAVQEFYFLVMVIIILFSYVKIMKVAKAA
LOR107	VAICMPLRHAELCSTRSTMHCILIIHGLSSVPCIVILSTFFAS--ASLLYKQHKLCTVEMLILYRWQGHVRSAVHEFYFLIMVIIILFSYVKIMKVAKAA
LOR108	VAICMPLRHSQLCSIHSTKHCILIIHSLSSVPCIVVLSTFFAS--ASFLYKQYRLCAIQLFMLYRWQDHVISAVQEFYFLIMAIIILFSYVKIMKVAKAA
LOR109	VAICMPLRHAELCSTRSTMHCIIIIHGFSSVPCIVVLSTFFAS--ASFLYKQYKICAIKIFMLYRWQDHVISAVQEFYFLIMVIIILFSYVKIMKVAKAA
LOR110	VAICMPLRHAELCSTRSTMHCILIIHGLSSVPCIVILSTFFAS--ASFLYKQPMICAIKIFMLYRWQDHVISAVQEFYFLIMVIIILFSYVKIMKVAKAA
LOR111	VAICMPLRHAELCSTRSTMHCILIIHGLSSVPCIVILSTFFAS--ASFLYKQPMICAIKIFMLYRWQDHVISAVQEFYFLIMVIIILFSYVKIMKVAKAA
Fugu_OR123-1	VAICRPLAYHSVMNPQRVYLLIFITWLIPFCSLFFNTITTSRYRLCG-SHIQRIYCVTYMIAKLSCSAS--IANAVLAYINYIFYFCHFSAVVWSYVYLI
Fugu_OR6765-1	VAICQPLVYHSVMNPQRVSALVFAAWMLPLTQFIVITVSTSTLRLCG-SQISKLYCVTYVIRKLQCSSS--ISTVIFPAFIITFYSCHVLLVIYSYFYIM
Fugu_OR4133-1	VAICQPLVYHSVMNPQRMCLLVFAAWILPVGQLLITITLTSTLRLCG-SQISRIYCVNYVIRRLECTPS--ITTVFSPAFTITFYFCHFLYVIYSYFYIM
Fugu_OR3630-2	VAICKPLQYSAIMRRRTVTIFLLLAWIAPTAQVAVPTIMNANRKLCN-FIFKGIICNS-TVFNLHCQRSE-TFNIYGLMVLVNLLIVPVMFILFSYARIL
Fugu_OR8617-1	LAICKPLQYSAIMRRRTVTIFLLLAWIAPTAQVAVPTIRNANRKLCN-FIFKGIICNS-TVFNLHCQRSE-TFNIYGLMVLVNLVIVPVMFILFSYARIL
Fugu_OR5510-1	LAICKPLQYSAIMRRRTVTIFLLLAWIAPTAQVAVPTIMNANRKLCN-FIFKGIICNS-TMFNLHCQRSE-TFNIYGLMVLVNLLIVPVMFILFSYARIL
Fugu_OR117-1	VAVCQPLHYHNKITKRSVMRLILLASLYPAFSVAACVYLSSMLPLCG-NAIPKVFCANWPVVKLSC-VPTWLNNLIGMLVSVTTVFLPLAFVLYTYGRIF
Fugu_OR2346-5	VAICNPLHYHRKMTHKTVVLLASSACLIPVFTIAVGLYLSVRLPLCG-NEIKRVFCGNWNVVKLSC-VSTIVNNIYGMFVTILTIIFPLLYVLYTYIQIV
Fugu_OR5287-2	VAICQPLRYARIINLSMLARLLLFTLVRSGSIMAALVGLASSLRFCGSNTIQHCYCDHMALVSLACGSTE-RNSAIGLVVIICFVGLDIPLILFSYIKIL
Fugu_OR142-1	VAICMPLHYNAIMVPRVVVFIISLVWGLDFSLILLLFVLQMRLPHCK-SVIMNVFCDNPSLLKLTCA-NTTINNVVGLFNTAVIQAVSLSVQAFSYVKIL
Fugu_OR1026-2	VAICNPLRYAAIMNHKMVIKLTVSAWAVAFVLVGILLGLTIRLNRCR-TLIANTFCDNASLFKLSCD-SVVINNIYGLTFTVVLFIASIGSIVVTYTKIT
Fugu_OR8298-1	VAICNPLRYAAIMNHRMVIKLTISAWAVAFVLVGILLGLTIRLNRCR-TLITNPYCDNASLFKLSCD-SVVINNIYGLTFTVVLFIASIGSIVVTYTKIT
Fugu_OR6030-1	VAVCYPLRHAALITIGNTGLMISVIWAFSLLSVLIQVSMVD---YPEMSLQMEVYCSSITLFVGPLSDTYDKAYTCLLFVSAGLAIAFSYIGVTVAARLA
Fugu_OR59-1	VAVCYPLRHAAVITIRNTGLMIYVIWAFSLLSGLIRVSLVN---YPELGLQMEEFCSSITLFVGPLSDTYDRAYTCLLFVSAGVAISFSYIGVTVAVRLA
Fugu_OR4208-3	IAVCFPLRHGQICTIRRTLLLIGLIWTTSMFSVLPDLFVTLAT--QPLFFYSQVFCLRETAFPNPHIIRKRDITYVVYLVIVWLIIFYTYFRIMFTAKTA
Fugu_OR4208-2	SAVCFPLHHLQVWTIRRTLTLILLMWTTTTVSATSDLVITLAT--EHLIVHSQVFCLRHTAFPLDVITTKRDVTYALFLVIIWITIFYTYFQILLMAKTA
Fugu_OR6818-1	VAICLPLRHSELCSMRRALHCILLVHGVSFLPCAVVLSMLFAS--ASYFYTQVKICSVEILIIHQWQSHLRSAISQFYFLIMCVVILLSYIKVMKVAKAA
Fugu_OR7903-1	VAICLPLRHPELCSLHNTQKCILIILTVSSVPCFIILSTFIAA--ASSVYTQHKRCSMEMFVSIPWQNHFKFAVYQFYFFIMSITITFSYVKIMKVAKAA
Pufferfish_OR8981-2	VAICRPLVYHSVMNTQRVSVLVFAAWFVPVVQMLVIIISTSTIRLCS-SKISRVYCVTYAKRRLECSVS--ITTVLFPAFMITFYLCLFLLVIYSYFHIM
Pufferfish_SCAF8981	VAICRPLVYHSVMNTQRVSVLVFAAWFVSVGQMIITIIITSTLRLCG-SEISRIYCVNYAIRRLECSAS--ITATVLPAFMIAIYFCVFLLVIYSYFHIM
Pufferfish_OR15134-1	VAICTPLRYPAVMRRRRVAALLLLAWVAPSILVVVPTITNANKKLCH-FVFKGIICNS-TIYDLLCQRSQ-ILNIYGLIVLVNLVILPVIFILFSYIRIL
Pufferfish_OR14339-2	VAVCQPLHYHNKITKRTVILLIGLAVTIPAITVTPFVYLSSILPLCG-NAIPKVFCANWHVVKLSC-VSTGLNNLLGMLVTTPVVFLPFVFVLYTYGRIF
Pufferfish_OR14328-1	VAVCQPLHYHNKITKKTVILLIGLAVTIPAIIVTPFVYLSSTLPLCG-NAIPKVFCANWLVVKLSC-VPTGLSDLFGMLVTTPVVFLPFVFVLYTYGRIF
Pufferfish_OR14677-8	VAICQPLRYARIINFSMLVRLLVFTFVRSSSIMATLVGLASSLTFCGSNTIQHCYCDHMALVSLACDSTE-KNSAAGLAVIICFVGMDIPLILFSYAKIL
Pufferfish_OR14536-2	VAVCFPLRHAAVITTRNTGLAIFAIWAFSLFSSLIRISLVD---FSELNLQMKEFCSGLTVFVGPLSDTYDRASTCLLFVSAGAAIIFSYIGVTVAAKSA
Pufferfish_OR14536-1	VAVCFPLRHAAVITTRNTGLAIFAIWAFSFFSSLIRVSLVD---FSELNLQMKEFCSSLTVFVGPLSDTYDRASTCLLFVSAGATILFSYIGVTVAAKSA
Pufferfish_OR10960-2	VAVCRPLRHAQICTVRRTLLLIALIWTTSCVSILPDLLVTLAT--QPLFFRSRVFCLRETAFPNPHIIRKRDVTYIAYLLAVWLVIFYTYFRILFTATSA
Pufferfish_OR10960-3	VAVCLPLRHAQICTVRRTRTLILLMWTTTVCSSASDLLLTLAT--ERLIVRSQVFCLRHTAFPHPVIVKKRDATYSLFLVMVWITMFYAYFRILVAAKTA
Pufferfish_OR12434-1	VAICLPLQHPRLCSLHNTWNCMLIINALSCVPCFIILSTFFAA--APSVYKQYKLCSMEMFVAVQWQKQLKFSVYQSYFVIMLITITLSYVKIMKIAKAA
Medaka_ORUn.33	AAICEPLRYRFIMDSCMLLRLLLFTLLRSGSIMATLVALAGSLRFCGSNVIQHCYCDHMALVSLACDSTD-RSWSAGVAVIVCFVGVDIPIIFFSYVKIL
Medaka_ORUn.2	FAICRPLYYHQQMALQSFIRFVVPLVLRNVVVITLLVALAGARSFCS-DVIHHCVCEHMALVELACGSTA-LNSLAGLMAIFLIPLLDFLIISASYIVIF
Medaka_OR21.18	VAICRPLEYHSVMSVQRTAVLVMLSWLVPLCCEMTVVSLTSTLKLCG-SHIDKLYCENWSLVKLACGST--KANDVTGLIFVVFYICHVLWIVFSYVQLI
Medaka_OR21.19	VAICRPLEYHFRMSKQNVVLLVCFSWFTPFCIMGLNVFLTSRLKLCS-PHLSRLFCVNWSIVRLACPQEDTTANNIVANMTIIIYVCHGLFIVWSYMYLV
Medaka_OR21.20	VAICRPLEYRSLVSKQKVLVLVGFSWFTPFCIVAVNTCLTSSIKLCS-RHLSRLFCVNWSIVRLACPQEEMTANNVVANITIIIYVCHGLFIVWSYMYLV
Medaka_OR13.14	VAICMPLRHAELCTPHGALQLILIIHSISAVPCTINLAIFYSA--ASTFLTQRGICYGERFILFTWQGQFRSAVSQLYFFIMFTIIVFSYIKIMKVAKTA
Medaka_OR13.12	VAICMPLRHAELCTPHGALQLILIIHSISAVSCIVILSIFFAA--VSPYFTQSTICSAEMFILFSWQRHLRSAISQFYFLIMVIIIVISYVKILKVAKTA
Medaka_ORUn.23	VAVCYPLRHASIITIRNTAAAVIAVWAVSSLNNLTRVLMFLKFPFEKIPT---DVCANVSVMLAPLTADYDRAYTCVVFVSAGVAVTSSYVGVIVAARSA
Medaka_ORUn.21	VAVCYPLRHASVITVRNTVAAVIAVWAVSSLNSLTRVLMFLKFPFEKMVY---DLCSNIALVLGALTAEYDRAYTCVVFVSAGVAVTSSYVGVIVAARSA
Medaka_OR14.15	VAVCYPLRHASVITVRNTVAAVIAVWAVSSLNSLTRVLMFLKFPFEKMVY---DLCSNIALVLGVLTAEYDRAYTCVVFVSAGVAVTSSYVGVIVAARSA
Medaka_OR14.13	VAICYPLRYANIMTNRMVLKLTVFAWGVAFVLVSILLGLTIRLNRCR-TLISNSYCDNASLFKLSCE-SVVINNIYGLTFTVVLLTSSIGCMVLTYTSIS
Medaka_OR14.4	VAICNPLRYANIMTSRMIIKLTVFAWGVPFVWVAVLLGLTIRLNRCR-TLIENPFCDNASLFKLSCE-SSVINNIYGLTFTAVLLTSSIGSIVLTYSKIT
Medaka_OR14.6	VAICNPLRYANIMTSRMIIKLTVFAWGVPFVWVAVLLGLTIRLNRCR-TLIENPFCDNASLFKLSCE-SSVINNIYGLAFTVVLLTSSIGSIVLTYSKIT
Medaka_OR13.4	LAICYPLQYNTVMTNAHIMKLIALVWSSSLVLIGVLFILLLRLNNCR-SEMSNPYCDNPNLLSLVCG-DTTINNIYGLFISALSQAVANGIVFYTYLRVL
Medaka_OR13.5	MAICSPLKYNAVMNSQNLLRIICLIWFLNFALMFTLFMLLARFKFCR-TNIADWYCNNPSLLKLVCS-DITLNNYYGLFTIILDMGAPLILILYTYAQIL
Medaka_OR13.6	IAICCPLNYNSILTSQNLMRIIFLVWFLNVSMILTLFMLLFRFKICR-ADVVDLFCNYPSVLKLVCG-DITLNNNYGLFTLIVIMGVPLMIILYTYVQIL
Medaka_OR13.7	VAICMPLRYSSIMTLRMVTGIVTLVWGLDFVLILLLFSLQARLPRCT-ALIRNVFCDNPSLLKLTCV-NTAVQNITGLFNTAVIQAISISIQVYSYVKIL
Medaka_ORUn.1	VAICSPLRYQELMTLQKVALLLLLTWTFSLLETAVGLVLVLQLPLCG-NRISKMFCTNWELVKLACQPA--SYDDVYSFLITVLHLSQAALIVVSYAQLI
Medaka_OR21.2	VSICKPLQYTIIMRRTIVFVLLGLAWIVPFCHLCIVVGGNVNSELCS-FTLNGIFCNN-SLNYLFCAGSK-ALLTFGLVTLFNIAILPMLFIIFTYIKIL
Medaka_OR21.3	VSICKPLQYAIIMKKIKAVVFVGLAWGVPVCHLVVPAIQNSKKELCS-FKLTGIFCNS-SLNDLFCAGLG-EPMISGFIILFNVVILPMLFIIFTYTKIL

```
Stickleback_OR1.1        LAVCQPLRYTEMVGASALSRLLLLALVRSGSIMATLVSLAGSLRFCGSDVIRHCYCDHMALVNLACGSTE-RNMVAGLAVIVCFVGVDIPLIFFSYVKIL
Stickleback_OR16.3       VAICRPLVYHSVMSKKRLLILVSFSWFTTFCISSTNMILTSRLHLCS-PYIDRLYCMNWNIVKLACFPADTIFNNTLANVTNIFYLCHGVLMVWSYLYVI
Stickleback_OR1.12       VAVCRPLRHGELCSTRRALHCVLIIHSISSIPCIVVLSIFFAS--VSHFYSKGRVCSVEMFILYKWQGHLRSAVSQFYFFIMFIAIVFCYIKIMKVAKAA
Stickleback_OR1.13       VAICKPLRHGELCSTRRALHCVLIIHSISAIPSFVLLSTFFAS--ASHFYSQVRECAVEMFIFHKWQGHLRSAMSQFYFFIMFIAIVFCYIKIMKVAKAA
Stickleback_ORUn.9       VAVCRPLRHAALVTPRSTMAAVAAVWAFSSLNVLTQVVLLLDFPFQDLSLRMDDFCSKGRVMLGAASEVYDRGYTCFLFVSTSVAVASSYVAVTMAARSA
Stickleback_OR7.1        VAICFPLRHASIVTTRMTGAAVAVMWTVGSLESFSQFVLFVRL---EN-ITVMRICS-QDIFQLQIYLAMNKGFTITIFVLVSMISIYTYVVVIVAVKSA
Stickleback_ORUn.6       VAICYPLQYNMIMTNKMVMKLTASAWGVALVLVGILLGLTLRLNRCR-TLITNPYCDNASLFKLSCE-SVLINNIYGLTFTVVLLTGSIGSMVITYTKIT
Stickleback_ORUn.8       VAICYPLQYNMIMTNKMVMKLTASAWGVALVLVGILLGLTLRLNRCR-TLITNPYCDNASLFKLSCE-SVLINNIYGLTFTVVLFTGSIGSMVITYTKIT
Stickleback_OR1.18       AAICMPLRYSSIMTPRVIICIISLVWGLDFVLITSLFSLQTRLPRCK-SVIMNVFCDNPSLLKLTCG-NTTVNNVLGLFTTAAMQAVSVSIQVFSYVKIL
Stickleback_OR16.10      VAICQPLNYHNKITSKAVSKLVAFAWIYPSFSIATCVYLSSRLPLCG-NKIPKVFCANWPVVKLSC-VSTVINNLVGMFVSISTVFLPLAFVMFTYIRII
Stickleback_OR16.32      VAICRPLVYHSGMTRQRMCVFVFFAWLIPFLLILISTVTTATSRLCG-SHIPKIFCVNWLISRMACSAS--VGTIVIPAFNYTFYFGHCVFVCWSYIYMI
Stickleback_OR16.1       VAICQPLNYHNKITSKAVSKLVAFAWTYPCFSIATCVYLSFRLPLCG-NKIPKVFCANWPVVKLSC-VSTVINNLVGMFLSISTAFLPLAFVMFTYIRII
Stickleback_OR16.5       VAICQPLNYHNKMTSKAVSKLVAFAWIYPCFSVATCVYLSCRLPLCG-NKVPKVFCANWPVVKLSC-VSTVINNLVGMVALITTVFLPLAFVMFTYIRII
Stickleback_ORUn.1       VSICKPLQYHTIMKKTTVRVLLAAAWCFPAAQIAVPVTLSANTKLCT-FTLRGIFCNN-SIYKLHCVSSR-VLSLYGVIVLCNVNLFPVLFIVFTYTKIF
Stickleback_ORUn.17      VSICNPLQYTTIMQRTTVYIFLCSAWILPACLVSVLILLNAKRKICT-FHLKGILCNS-SVQTLQCARSL-IVNIYG-LILVTFLLFPVVFIFFTYSKIL
Zebrafish_OR126-7        VAICRPLEYHSVMTSQRVVEWILFCWLAPLFCMSLLIVLLSRLVLCG-STIEKLYCEIWAVAKLSCFSN--TVNNVFGLTVILVYFGHGVLIYCSYFQLI
Zebrafish_or126-3        VAICRPLEYHSMMTNQKILKCVLFCWLSPFFCMAVLILLIARLTLCG-FRIEKLYCEIWGVAKLSCFST--TLNNVFGYIVIIAYVGHAILIFCSYIYLI
Zebrafish_OR123-1        LAICRPLYYHNIMTTVTVWRLLTFIWMFPCCTAILLILMTLRFPICI-KKISKLYCETWVLERLVCGVD--TAQFVFNGILTCAVNALVWFVFLSYINIL
Zebrafish_OR125-1        VAICKPLDYHSRLTKNTCVKLILFSWIVPTSFSVASILLSNLRAFCK-YHIDKLYCDNWSIVKLSCVSS--FVNNVFGYGIAVIFFSFVVLIIFSYIKLV
Zebrafish_OR127-1        VAICNPLKYHKIMSLRLTARYMAMASFCVVFSMALVIFFSAKLPLCG-TDVTRLYCSNWSVVRLSCGTSTLSNNILGFFVTTATVFLPAFFILYTYIRIL
Zebrafish_OR128-10       VAICKPLQYNSIMTPRNLSVLIALSWIYPMLCVGIAAILNARLTMCG-NKLWKVYCHNWEIVKLSC-ASTIINNVFGFFIVTTTVIMPLSFILYSYVKIL
Zebrafish_OR116-2        IAICMPLRYNALMTPRAVMGIVALVWGLDLFFIILLFALQSKYSRCR-SDILNVFCDNPSLLKLTCS-NTIVSNIIGLFNTAMIQIISVSIQAFSYVKIL
Zebrafish_zor6a          VAICKPLHYNEIMNSSMFLKLFLFTLIRSGTIMSTLVALASPLSFCGSNVIYHCYCDHMALVSLACDSIA-QNQTMGLIVIICFVGIDTSVIFFSYVKIL
Zebrafish_OR117-1        IAICNPLRYNSIMTPSRIFGLIAFAWGLDFAVITVLFSLQARLVKCK-SFITNVYCDNPSLVLLSCGGDLSVNNIYGLFLTAFIQITSVFIQMFSYGCIV
Zebrafish_OR115-15       VAICNPLRYQTIMTNKTVITLSALAWGIALLFISILIGLTLRLSRCR-TFISNPFCDNASLFKLSCE-DVTINNLYGLIYTVLLFGSSMGSIAVTYIKIT
Zebrafish_OR133-7        VAICFPLRHAEFTSFKRTHTAICVVWVLGLIQCLSEMIIFYSF---DNNISMNLFCSRTTLFRLQIYKKFDIAFTCIFFVLVSFVIIFTYASISAVAKTA
Zebrafish_OR132-1        VAICFPLXHCTIAHPKRTGIAIGIIWFLSSVNVVIDIIIALIN---NPYLAEIAFCTLEKLFIAKWQIDKSQGFDVLYFVSVAVTIIFTYIMIMITARSV
Zebrafish_OR134-1        IAICYPLRHAQICTVHRAYVLIGVIWFVSVVPDITDLFVTLAT--EPLFFQTSVFCLRQNVFKDPVLLYKRQAFDGIYFSLVFLTLLCTYLKIMFTAHSH
Zebrafish_OR131-2        VAICLPLRHASISSPKNTINGLLIIWGVSSVIPLFIFIVSFTY--TPPAMNSYVVCSNDVMFQVKWLAEMRALSQQLLFVIMLCIVGSTYIKIMVAAKSA
Zebrafish_OR131-1        VAICLPLRHASISSPKNIINGLLIVWGVSSVIPLFVFIGSFAY--TPPAMNSYVVCSIEVMLQVKWLADTRATSQQLLFVIMLCIVGSTYIKIMIAARSA
Goldfish_OR3             VAICRPLEYHSVMTNQRIIECILFCWLSPFFCMCVVIVLTSRLILCG-SSIEKLYCENWSVVKLSCFST--TVNNVIGYVIIIIYFGHAVLIFCSYIHLI
Goldfish_OR2             VAICNPLRYPNIVTKLNIFCLCLAAWVLTNTCPLMMAIRAYPLPYCSGT-IIHCYCDHISITSLACTN-RALYGVPAFAIAMVVLLGPLAFIIFSYCAII
Rainbow_trout_OR500-1    VAICRPLLYHTIVTSLTVRKLLLFSWCYPLFIALIAVSLAVRLPLCG-SRIDKIFCDIPSILKHACSPI--TINQNLNKFVIVVHVLQILFIVFSYCQIV
Cutthroat_trout_OR600-1  VAICNPLRYATIMTNKMVIRLSMSAWGVAIVLVGILLGLTIRLSRCR-SNIFNPYCDNASLFKLSCE-NVFINNIYGLIFTVVLFVSSIGSISLTYLKIA
Brown_trout_OR500-1      VAICRPLLYHTIVTSLTVRKLLLFSWCYPLFAGLIAVSLTARLSLCG-CRIDKIFCDNPSILKHACLPI--TINQNLNKCLIVVHVLQIFFIVFSYSQIV
Atlantic_salmon_SORB     VAICNPLRYSIIMTNKMIIRLSVSAWGVAIVLVGILLGLTIRLSRCR-STILNPFCDNASLFKLSCE-SVFINNMYGLTFTIVLFISSIGSIALTYMKIA
Medaka_mfOR2             VSICKPLQYQSLMSKRRVTVMLILSWLWPLFQLTVAISVIINQRPCS-FSLEGIFCDS-ALQRLLCIKLE-ELAVLDMIILFNITVLPSLFILFTYICIL
Medaka_mfOR1             VSICKPLQYQSLMSKRRVTVMLILSWLWPLFQLTVAISVIINQRPCS-FSLEGIFCDS-ALQLLLCKKLE-ELAVLDTLILVNVVVLPSLFILFTYIRIL
Medaka_OR_Y3             VAICNPLRYTNIMTNRMVLKLTVFAWGVAFVLVGILLGLTIRLSRCR-TLIENPFCDNASLFKLSCE-SVVINNIYGLTFTVVLFSSSIGSTVLTYTSIS
Medaka_ORY1              VAICNPLRYANIMTSRMIIKLTVFAWGVPFVWVAVLLGLTIRLNRCR-TLIENPFCDNASLFKLSCE-SSVINNIYGLTFTAVLLTSSIGSIVLTYSKIT
Frog_OR-52D1             VCICNPLRYSSIMSLATVFKLIGAAWLYVIALIAILVLLTIRLPLCG-SVIQKIYCDNISVVKLSC-TDTTANNVFGLLITAAIIGLMPILTLMSYAQIL
Frog_OR-52E8             MCICNPLRYHSLMTSGTVLKLIGAAWLYIIVLVTIHVILTIRLPLCG-SVIQKIYCDNWSVVRLSC-IDTTLNNVFGLLMSS-ITGLLPGGVLISYVKIL
Frog_OR-52A1             VAICRPLRYHNIMTKNLLAWLTVTVIVRSFILVCPLVILTSMVQFCRLNIILNFTCENMALLSLGCGDTT-KPQIAGLIVRVLVTVLDVSLLMISYSTIL
Frog_OR-52P1             IAICRPLRYHNIMTTSFLAWLTVIVLIRNCLLICPLIVLISMVQFCRSNVILNFACENMALLSLGCGDTT-KPQIAGLIVRIIATVLDGSLLLISYSTIL
Frog_OR-52A5             VAICFPLRHSEFCTRQRSAIALVGIWAIGLIPNIADCIILSTS--VPSFFSLHVICARSAFMNTSVQTLIRNLTHILSFTLVGLIIIFTYIKIMMVALRV
Frog_OR-2M5              VAICHPLRHTEICSGHRLTMAIALMWTLGLTPNVADFITFSLS--ANKYFSLHVICSRESLTVNPTQNTIRSNVLVITVSLVALIILYTYIKVMLVARKV
Frog_OR-2M2              VAICQPLRYHSIISNKLVTLLCYFLWVFAALIGSIVAVIVGQLPYCGPR-VKNCFCVNSAVAVLACVD-VTLARRTVFILAMCVLLLPLAVIILSYVLII
zebrafish_OR10.1         IAICFPLRHESINTNSRMAYIISAVWFFCVVTLLYAVFSILTLSFCGSE-MNSYFCDYTPVLVLACSD-ITQQLNYATAATILFIGVASIFIFFTYIGIL
zebrafish_OR10.14        VAICLPLRYHMIVTFRSMLVIIGLTWALTLLMVMTATIFISRLSFCKVVTVNSFYCDHGPIYRSACSN-NFPSSVIGSLYPIVILGLPVFFIIFSYTCIA
zebrafish_OR15.39        IAICWPLRYSTINTNLRMLLIIALIWILVTLLDIFPVIFASRLPYCSSA-VLSCCCEHGPVYRLACTD-TTYNRQLGTVKTMITLLGPLFFIVFTYVIVV
Medaka_ORUn.6            VAICFALRQNSINTVRSMCCIITITWCYILGVTAYSVALITKLSFCSSK-VFSYFCDYAPVYRLACND-NSKQWFAGTFFSYLNLLGPFTFIVLSYVSIL
Medaka_ORUn.28           VAIMFPLHYQVTVTPKIMLSIILAFWLFVIIAVSTAAGLLTRLSFCNSV-INSYFCDHGQIFGIACND-YSPSYIFSDVLIAIILWIPLIIVISSYVCIC
Medaka_ORUn.27           VAIIFPLHYQLRVNQTFMLSMIASFWVFTITAVLISVGLLTRLSFCKSV-INSYFCDHGQLYRLACND-NFPNDVVSFFYPVVIFWLPLVFILSSYLYIG
Stickleback_OR7.3        IAICLPMRQNLFNTLPIMLCIVGGTWVYNLGRIAHDTAIMTRLSFCNSR-VNSYFCDYAPVFRLACND-YTLHWSIASTSSMVNLMVPFTVIILSYLSIL
Stickleback_OR7.4        IAICFPLRHNSVNTLQSMSCIIGFTWSFSVGVIAFSTGIMTQLSFCKSR-VFSYFCDYAPVFRLACND-YAMQWSAASFLSFMILGGPFVFIILSYVTIL
Stickleback_OR7.8        IAIIHPLHYQVRVTHRFMLSLIAFFWVFATTAVLIAVGLITRLSFCRSA-INSYFCDHGQIYRLACND-NFPNYVLSCLFPVLIFWLPFIFILLSYLYIG
Fugu_ORUn.83             VAIIFPLHYQMRVTHRIMMSLIASFWAFIIVAVLIAVGLITRLSFCKSV-IHSYFCDHGQIYRLACND-HFPSYVVSCLYPVFIFWLPLLFILLSYLYIS
Fugu_ORUn.20             IAICFPLQQNSINTLRSMSCIVAVTWSYSLGLVAFSTGIMTWLSFCKST-VRSYFCDYAPTFRLACND-YTLHWAVSSTASVVNLVAPFTFILLTYAAIL
Pufferfish_OR7.1         VAITYPLHYQVRVTHRFMISMIASFWAFIIVAVLIAVGLITRLSFCKSV-VHSYFCDHGQIYRLACND-HFPNYVVSCLYPVFIFWLPLLFILLSYLYIS
Pufferfish_OR7.3         IAICFPLQHNSINTLWSMSWIVAVTWCYSLGLAAFSASIMTWLSFCKSE-VYSYFCDYAPTFRLACND-NTLHWAVSSTASVLNLVAPFVFIVLTYTAIL
zebrafish_OR10.29        VAICYSLRYQTVMTNRVTYILITTAWITAFIAPTVATLDTQQLPYCGPL-ILQCYCDHISITNLACAE-NSKQLLVALCMALLVLLLPLVCIIYSYCHII
zebrafish_OR10.31        LAICHPLRYPVLMTNRTMRLLVGFSWVTAMIAPSISLSLTLKLPFCGPM-IAHCFCDSLSMNQLACAD-ASFYNLIAFGVAMFVLLVPLSFIIFSYASIL
zebrafish_OR15.58        IAICNPLRYHTLIKNSTILIICAFVWTANMLQLVGVTLLSLSVKYCGSV-IPHCYCDHVSISKLACGD-AATMKVTSTAIAMFVLWGPLSFILFSYVSII
zebrafish_OR15.57        VAVCNPLRYCVLITNKTILLACALIWMLCISWLTFMVYNTFDEPYCGSI-ITQNYCDHNSIIKLSCGD-IGQKKFYSFSFAMFVLLGPLIFIIFSFTAII
Medaka_OR13.1            VAVWFPLRYPAIITNKTTSIACSLCWTLTFIRMMGIVLHASSLSFCDSK-ILQCFCDHASITKLACGENVAFVQEVAFANAMVTLLFPLSFIIFSYFSII
Medaka_OR13.3            IAVCIPLRYPVLITNNIISVLCGFAWFIPAPLMMTIVLHALTLSYCKSI-IAQCYCDHISLTNQGCGGNLREVQVTALCIAMLCLLLPLAFILFSYVSII
Stickleback_OR1.25       IAICIPLRYPVLVTNNTISVICGFAWFIPLPLMLTIVLHTLTLPFCKSV-IAQCYCDHISITSQACGENVQTVLVTALCVAMFCLLLPLAFILFSYILIF
Fugu_ORUn.8              ISICFPLRYTALVSKKVISALCGIAWFIPVSLLVAVIVQALALPYCKSV-IVQCFCDHISITNLACGEGVQSVRVGTLCIALLCLLLPLTFIVFSYVTII
Pufferfish_OR16.7        ISICLPLRYTALVSTKFISALCGIAWFFPVSLMVTIIVQALALTFCEPL-IVQCFCDHISITNQACGEGVQSVRVSTLCIALFCLLLPLAFIVFSYVSII
Frog_OR669.1             IAICHPLRYACIMTNRTTAILCCFFWIFAAAISAVITILDSTVPICNKK-IKSCFCTNTGLTALSCAD-VTFVKRLAFGLAMFVLLLPFTFIIFSYIAII
Amphioxus_105.1          YFICYPLQYETKVTTSRLAIGMVLVDTFSFICTSLPLILVRDAGDNVSLTKTRSFQPAFVIRIIGMVAQG----------LSVLVIIGLYFIVLKEAKRQ
Amphioxus_39.1           HFICNPLHYRSKVTTRRVIIGIVSVRAFALIFGIGQTL------IKRLNPCESLFCQPEAFTSTSPAAIFKNVCIVGVLLAVLAIIIVYYLVYKEARKQQ

                                210       220       230       240       250       260       270       280
                         ....|....|....|....|....|....|....|....|....|....|....|....|....|....|....|....|....|
LOR1                     -RKCLI--SSENRSKFMQTCLPHLICLIIVVVSLLFDLLYMRFGSQLSQNVKNFMAMEFLLVPPIINPLIYGFKLTQIRNRIIQF
LOR2                     -RKCLI--SSENRSKFMQTCLPHLICLIIVVVSLLFDLLYMRFGSQLSQNVKNFMAMEFLLVPPIINPLIYGFKLTQIRNRIIQF
LOR3                     -RKCLI--SSETRSKFMQTCLPHLICLIIVVVSLLFDLLYMRFGSQLSQNVKNFMAMEFLLVPPIINPLIYGFKLTQIRNRIIQF
LOR4                     -RKCLI--SSENRSKFMQTCLPHLICLIIVVVSLLFDLLYMRFGSQLSQNVKNFMAMEFLLVPPIINPLIYGFKLTQIRNRIIQF
LOR5                     -RKCLI--SSENRSKFMQTCLPHLICLIIVVVSLLFDLLYMRFGSQLSQNVKNFMAMEFLLVPPIINPLIYGFKLTQIRNRIIQF
LOR6                     -RTCRT--SKEGRSKFMQTCLPHLISLIVVVVTLLFDLLYMRFGSQLSQSVKNFMAMEFILIPPTINPLIYGFKLTKIRNRIQSF
LOR7                     -RTCRT--SKEGRSKFMQTCLPHLISLIVVVVTLLFDLLYMRFGSQLSQSVKNFMAMEFILIPPTINPLIYGFKLTKIRNRIQSF
LOR8                     -RTCRT--SKEGRSKFMQTCLPHLISLIVVVVTLLFDLLYMRFGSQLSQSVKNFMAMEFILIPPTINPLIYGFKLTKIRNRIQSF
LOR9                     -RACRT--SKEGRSKFMQTCLPHLISLIVVVVTLLFDLLYMRFGSQLSQSVKNFMAMEFILIPPTINPLIYGFKLTKIRNRIQSF
LOR10                    -RTCRT--SKEGRSKFMQTCLPHLISLIVVVGTLLFDLLYMRFGSKLSQSVKNFMAIEFILIPPTINPLIYGFKLTKIRNRIQSF
LOR11                    -VISYRS-SKEVRKKAAQTCLPHLIVLINFSCLCAYDVIVVGLEADFPKTVRLIMALQIILYHPLFNPIMYGLKMKEIHKHLKRL
LOR12                    -VISYRS-SKEVRKKAAQTCLPHLIVLINFSCLCAYDVIVVGLEADFPKTVRLIMALQIILYHPLFNPIMYGLKMKEIHKHLKRL
LOR13                    -VISYRS-SKEVRKKAAQTCLPHLIVLINFSCLCAYDVIVVGLEADFPETFRLIMTLQMILYHPLFNPIMYGLKMKEIHKHLKRL
LOR14                    -VISYRS-SKEVRKKAAQTCLPHLIVLINFSCLCAYDVIVVGLEADFPKTFRLIMTLQMILYHPLFNPIMYGLKMREIHKHLKRL
LOR15                    -VISYRS-SKEVRKKAAQTCLPHLIVLINFSCLCAYDVIVVGLEADFPKTFRLIMTLQMILYHPLFNPIVYGLKMKEIHKHLKRL
LOR16                    -VISYRS-SKEVRKKAAQTCLPHLIVLINFSCLCAYDVIVVGLEADFPKTVRLIMALQIILYHPLFNPIMYGLKMKEIHKHLKRL
LOR17                    -VISYRS-SKEVRKKAAQTCLPHLIVLINFSCLCAYDVIVVGLEADFPKTFRLIMTLQMILYHPLFNPIMYGLKMKEIHKHLKRL
LOR18                    -VISYRS-SKEVRKKAAQTCLPHLIVLINFSCLCAYDVIVVGLEADFPKTVRLIMALQIILYHPLFNPIMYGLKMKEIHKHLKRL
LOR19                    -VISYRS-SKEVRKKAAQTCLPHLIVLINFSCLCAYDVIVVGLEADFPKTVRLIMALQIILYHPLFNPIMYGLKMKEIHKHLKRL
LOR20                    -VISYRS-SKEVRKKAAQTCLPHLIVLINFSCLCAYDVIVVGLEADFPKTVRLIMALQIILYHPLFNPIMYGLKMKEIHKHLKRL
LOR21                    -VISYRS-SKEVRKKAAQTCLPHLIVLINFSCLCAYDVIVVGLEADFPKTVRLIMALQIILYHPLFNPIMYGLKMKEIHKHLKRL
LOR22                    -VISYRS-SKEVRKKAAQTCLPHLIVLINFSCLCAYDVIVVGLEADFPKTVRLIMALQIILYHPLFNPIMYGLKMKEIHKHLKRL
LOR23                    -VISYRS-SKEVRKKAAQTCLPHLIVLINFSCLCAFDVITVQLEADFPKTVRFIMTLQIVMYHPLFNPIMYGLKMKEIHKHLKRL
LOR24                    -VISYRS-SKEVRKKAAQTCLPHLIVLINFSCLCAFDVITVQLEADFPKTVRFIMTLQIVMYHPLFNPIMYGLKMKEIHKYLKRL
LOR25                    -VISYRS-SKEVRKKAAQTCLPHLIVLINFSCLCAFDVITVQLEADFPKTVRFIMTLQIVMYHPLFNPIMYGLKMKEIHKYLKRL
LOR26                    -VISYRS-SKEVRKKAAQTCLPHLIVLTNFSCLCAFDVITVQLEADFPKTVRFIMTLQIVMYHPLFNPIMYGLKMKEIHKYLKRL
LOR27                    -IITYRS-CREVRRKAAQTCLPHL-------------------------------------------------------------
LOR28                    -IITYRS-CREVRRKAAQTCLPHL-------------------------------------------------------------
LOR29                    -IITYRS-CREVRRKAAQTCLPHL-------------------------------------------------------------
LOR31                    -LICRKR-SSEFKAKVIQSCLPHIITFVNYSITVFCDVALSRIDLELNPFLAVILSLEFVVIPPILNPLVYGLKLPEIRKLILKM
LOR32                    -LICRKR-SSEFKAKVIQSCLPHIITFVNYSITVFCDVALSRIDLELNPFLAVILSLEFVVIPPILNPLVYGLKLPEIRKLILKM
LOR33                    -LICRKR-SSEFKAKVIQSCLPHIITFVNYSITVFCDVALSRIDLELNPFLAVILSLEFVVIPPILNPLVYGLKLPEIRKLILKM
LOR34                    -LICRKR-SSEFKAKVIQSCLPHIITFVNYSITVFCDVALSRIDLELNPFLAVILSLEFVVIPPILNPLVYGLKLPEIRKLILKM
LOR35                    -LICRKR-SSEFKAKVIQSCLPHIITFVNYSITVFCDVALSRIDLELNPFLAVILSLEFVVIPPILNPLVYGLKLPEIRKLILKM
LOR37                    SAVLRATAPDEDRWKAFHTCGTHLIIMMCFYLVGSVTFLSHNLNIPIPTDVNTFMGLMYILFPATVNPIIYGVRTKEIRNGFFRM
LOR38                    SAVLRATAPDEDRWKAFHTCGTHLIIMMCFYLVGSVTFLSHNLNIPIPTDVNTFMGLMYILFPATVNPIIYGVRTKEIRNGFFRM
LOR39                    SAVLRATAPDEDRWKAFHTCGTHLIIMMCFYLVGSVTFLSHNLNIPIPTDVNTFMGLMYILFPATVNPIIYGVRTKEIRNGFFRM
LOR40                    SAVLRTTAPDEDRWKAFHTCGTHLIIMMCFYLVGSVTFLSHNLNIPIPTDVNTFMGLMYILFPATVNPIIYGVRTKEIRNGFFRM
LOR41                    SAVLRATAPDEDRWKAFHTCGTHLIIMMCFYLVGSVTFLSHNLNIPIPTDVNTFMGLMYILFPATVNPIIYGVRTKEIRNGFFRM
LOR42                    SAVLRATAPDEDRWKAFHTCGTHLIIMMCFYLVGSVTFLSHNLNIPIPTDVNTFRGLMYTLFPATVNPIIYGVRTKEIRNGFFRM
LOR43                    -ITCLVSRRSEAKTKAINTCVAQLVILIIFEIVGTFTILSHRFTN-VSADLQKIMGMLIFLVPPLLNPIVYGLYTSEIRNTMLR-
LOR44                    -ITCLVSRRSEAKTKAINTCVAQLVILIIFEIVGTFTILSHRFTN-VSADLQKIMGMLIFLVPPLLNPIVYGLYTSEIRNTMLR-
LOR45                    -ITCLVSRRSEAKTKAINTCVAQLVILIIFEIVGTFTILSHRFTN-VSADLQKIMGMLIFLVPPLLNPIVYGLYTSEIRNTLLR-
LOR46                    -ITCLVSRRSEAKTKAINTCVAQLVILIIFEIVGTFTILSHRFTN-VSADLQKIMGMLIFLVPPLLNPIVYGLYTSEIRNTMLR-
LOR47                    -ITCLVSRRSEAKTKAINTCVAQLVILIIFEIVGTFTILSHRFTN-VSADLQKIMGMLIFLVPPLLNPIVYGLYTSEIRNTMLR-
LOR48                    -VVCLTSKNKSLNSKALRTCSTHLVVYLIMVFNGMSIITLHRFP--HYSDYRKLCTILFHIIPCSINPIIYGVQSKEIKKFFSKS
LOR49                    -VVCLTSKNKSLNSKALRTCSTHLVVYLIMVFNGMSIITLHRFP--HYSDYRKLCTILFHIIPCSINPIIYGVQSKEIKKFFSKS
LOR50                    -VVCLTSKNKSLNSKALRTCSTHLVVYLIMVFNGMSIITLHRFP--HYSDYRKLCTILFHIIPCSINPIIYGVQSKEIKKFFSKS
LOR51                    -VVCLTSNNRSLNSTAFKTCSTHLFVYLIMFLCGMFVVILHRFP--QYSDYRKLCSILFHIVPGSLNPIIYGIQSKEMKKFFLKL
LOR52                    -VVCLTSKNKSLNSKALKTCSTHLVVYLIMVFNGMSIITLHRFP--HYSDYRKLCTILFHIIPCSINPIIYGVQSKEIKKFFSKS
LOR53                    -VVCLTSKNKSLNSKALKTCSTHLVVYLIMVFNGMSIITLHRFP--HYSDYRKLCTILFHIIPCSINPIIYGVQSKEIKKFFSKS
LOR54                    -VVCLTSKNKSLNSKALRTCSTHLVVYLIMVFNGMSNIALHRFP--QYSDYRRLCSILFHIVPGSLNPIIYGVQSKEMKKFFLKL
LOR55                    -VVCLTSKNKSLNSKALRTCSTHLVVYLIMVFDGMSNIALHRFP--QYSDYRRLCSILFHIVPGSLNPIIYGVQSKEMKKFFLKL
LOR56                    -VVCLTSKNKSLNSKALRTCSTHLVVYLIMVFDGMSNIALHRFP--QYSDYRRLCSILFHIVPGSLNPIIYGVQSKEMKKFFLKL
LOR57                    -VVCLTGKNKSLNSKALRTCSTHLVVYLIMVFNGMSNIALHRFP--QYSDYRRLCSILFHIVPGSLNPIIYGVQSKEMKKFFLKL
LOR58                    -VVCLTSKNKSLNSKALRTCSTHLVVYLIMVFNGMSNIALHRFP--QYSDYRRLCSILFHIVPGSLNPIIYGVQSKEMKKFFLKL
```

```
LOR59                -VVCLTSKNKSLNSKALRTCSTHLVVYLIMVFNGMSNIALHRFP--QYSDYRRLCSILFHIVPGSLNPIIYGVQSKEMKKFFLKL
LOR60                -VVCLTSKNKSLNGKALRTCSTHLVVYLIMVFNGMSNIALHRFP--QYSDYRRLCSILFHIVPGSLNPIIYGVQSKEMKKFFLKL
LOR61                -VVCLTSKNKSLNSKALRTCSTHLVIYLIMLFSGMSNITLHRFP--QYSDYRKLCSILFHIVPGSLNPIIYGVQSKEMKKFFLKL
LOR62                -VVCLTSKNKSLNSKALKTCSTHLVVYLIMVFNGMSIITLHRFP--HYSDYRKLCTILFHIIPCSINPIIYGVQSKEIKKFFSKS
LOR63                -VVCLTSNNRSLNSTAFKTCSTHLFVYLIMFLCGMFVVILHRFP--QYSDYRKLCSILFHIVPGSLNPIIYGIQSKEMKKFFLKL
LOR64                -VVCLTSNNRSLNSTAFKTCSTHLFVYLIMFLCGMFVIILHRFP--QYSDYRKLCSILFHIVPGSLNPIIYGVQSKEMKKFFLKL
LOR65                -VVCLTSNNRSLNSTAFKTCSTHLFVYLIMFLCGMFVIILHRFP--QYSDYRKLCSILFHIVPGSLNPIIYGVQSKEMKKFFLKL
LOR66                -AACLTNKSNSLNSKALKTCSTHLCLYLIMLVSGMILITLHCFP--QYAEYRKISAILFNVVPGSLNPVIYGLQSKKIYKSLSNI
LOR67                -AACLTNKSNSLNSKALKTCSTHLCLYLIMLVSGMILITLHRFP--QYAEYRKISAILFNVVPGSLNPVIYGLQSKEIYKSLSNI
LOR68                -AACLTNKSNSLNSKALKTCSTHLCLYLIMLVSGMILITLHRFP--QYAEYRKISAILFNVVPGSLNPVIYGLQPKEIYKSLSNI
LOR69                -AACLTNKSNSLNSKALKTCSTHLCLYLIMLVSGMILITLHRFP--QYAEYRKISAILFNVVPGSLNPVIYGLQSKEIYKSLSNI
LOR70                -VVCLTSKNKSLNSKALKTCSTHLCLYLIMLVSGMILITLHRFP--QYAEYRKISAILFNVVPGSLNPVIYGLQSKEIYKSLSNI
LOR71                -AACLTNKSNSLNNKALKTCSTHLCLYLIMLVSGMILITLHRFP--QYAEYRKISAILFNVVPGSLNPVIYGLQSKEIYKSLSNI
LOR72                S-TDKVSARKARNTVLLHLMQLGLTLLSTMHGPIVISLSTTLQR--LMVRIKNIFYVFIYILPRCLSSLIYGLRDQTIRPVLMYL
LOR73                S-TDKASAHKARNTVLLHLMQLGLSLLSTMHASIIISLSTTLQR--LIVRIKSILYVFIYILPRCLSSLIYGLRDQTIRPVLMYL
LOR74                S-TDKASAHKARNTLLLHLVQLGLILLSTMHGTIIVSLSTTLPR--LIIRIRSSFYVFIYILPRCLSSLIYGLRDQTIRPVLMYL
LOR75                S-TDKASARKTRNTLLLHLVQLGLSLLSTMHSSIIVSLSTIPQR--LFVHIRSTFYVFIYILPRCLSSLIYGLRDQTVRPVLMYL
LOR76                S-TDKASAHKARNTVLLHLVQLGLSLLSTMHASIIISLSTTLQR--LIVRIKSILYVFIYILPRCLSSLIYGLRDQTIRPVLMYL
LOR77                S-TDKASAHKARNTLLLHLVQLGLSLLSTMHSSIIISLSTIPQR--LFVHIRSTFYVFIYILPRCLSSLIYGLRDQTVRPVLMYL
LOR78                S-TDKASARKGRNTLLLHLVQLGLSLLSTMHSSMIVSLSTTLPT--QIIRIRSIFYVFIYVLPRCLSSLIYGLRDQTIRPVLMYL
LOR79                S-TDKASARKGRNTLLLHLVQLGLSLLSTMHSSMIVSLSTTLPT--QIIRIRSIFYVFIYVLPRCLSSLIYGLRDQTIRPVLMYL
LOR80                S-TDKASARKGRNTLLLHLVQLGLSLLSTMHSSMIVSLSTTLPT--QIIRIRSIFYVFIYVLPRCLSSLIYGLRDQTIRPVLMYL
LOR81                S-TDRASARKGRNTLLLHLVQLGLSLLSTMHSSMIVSLSTTLPT--QIIRIRSIFYVFIYVLPRCLSSLIYGLRDQTIRPVLMYL
LOR82                T-TDKASAQKASKTLLLHLVQLGLILLSALHTPSIQAISKVVDA--TSDRLHIVHYVFIIILPRCLSSLIYGLRDQTIRPVLMYL
LOR83                SKD----AKKARNTILLHVFQLLLCMATYVAPQLLDILQQWFPK--NRTDSLFAYYIIVQILPRSVSPIIYGIRDNTFRKYLKRL
LOR84                SKD----AKKARNTILLHVFQLLLCMATYVAPQLLDILQQWFPK--NRTDSLFAHYIIVQILPRSVSPIIYGIRDNTFRKYLKRL
LOR85                SKD----AKKARNTIILHVFQLLLCMATYVAPQLLDILQQWFPK--NRTDSLFAYYIIVQILPRSVSPIIYGIRDNTFRKYLKRL
LOR86                SKD----AKKARNTIILHVFQLLLCMATYVAPQLLDILQQWFPK--NRTDSLFAYYIIVQILPRSVSPIIYGIRDNTFRKYLKRL
LOR87                SKD----AKKARNTIILHVFQLLLCMATYVAPQLLDILQQWFPK--NRTDSLFAYYIIVQILPRSVSPIIYGIRDNTFRKYLKRL
LOR88                SKD----AKKARNTIILHVFQLLLCMATYVAPQLLDILQQWFPK--NRTDSLFAHYIIVQILPRSVSPIIYGIRDNTFRKYLKRL
LOR89                SKD----AKKARNTILLHVFQLLLCMATYVAPQLLDILQQWFPK--NRTDSLFAYYIIVQILPRSVSPIIYGIRDNTFRKYLKRL
LOR90                SKD----AKKARNTILLHVFQLLLCMATYVAPQLLDILQQWFPK--NRTDSLFAYYIIVQILPRSVSPIIYGIRDNTFRKYLKRL
LOR91                SKD----AKKARNTIILNGFQLLLCMAIYAAPQLLDALQKWFPK--NFTDSLFAYYIIVQVLPRSISPFIYGIRDNTFRRYLKRL
LOR92                SKD----AKKARNTILLNGFQLLLCMAIYAAPQLLDALQKWFPK--NFTDSLFAYYIVVQVLPRSISPFIYGIRDNTFRRYLKRL
LOR93                SGENKKSTRKGLRTVILHGFQVFLCLVQLWCPFIEVALFQ-IDL--LYVNVRYFNYITFILAPRCLSPLIYGLRDEKFLLALKHV
LOR94                SGENKKSTRKGLRTVILHGFQVFLCLVQLWCPFIEVALFQ-IDL--LYVNVRYFNYITFILAPRCLSPLIYGLRDEKFLLALKHV
LOR95                SGENKKSTRKGLRTVILHGFQVFLCLVQLWCPFIEVALFQ-IDL--LYVNVRYFNYITFILAPRCLSPLIYGLRDEKFLLALKHV
LOR96                SGENKKSTRKGLRTVILHGFQVFLCLVQLWCPFIEVALFQ-IDL--LYVNVRYFNYITFILAPRCLSPLIYGLRDEKFLLALKHV
LOR97                SGENKKSTRKGLRTVILHAFQVFLCLIQLWCPFIEVALFQ-IDL--LYVNVRYFNYITFILAPRCLSPLIYGLRDEKFLLALKHV
LOR98                SGENKKSTRKGLRTVILHAFQVFLCLVQLWCPFIEVALFQ-IDL--LYVNVRYFNYITFILAPRCLSPLIYGLRDEKFLLALKHV
LOR99                SGEDKKSSWKGLRTVILHGLQLLLCLIQLWTPFIEGAVFQ-IDF--LFINVRFSSYILFALAPRCLSPLIYGLRDETFFHALKNE
LOR100               SGEDKKSSWKGLRTVILHGFLLLLCLIQLWTPFIEGAVFQ-IDF--LFINVRFSSYILFALAPRCLSPLIYGLRDETFFHALKNE
LOR101               SGEDKKSSRKGLRTVILHGFQLLLCLIQLWSPFIESTLLR-FDF--LFINVRYSNYVLFNLTPRCLSPLIYGLRDETFFHALKNE
LOR102               SGEDKKSSWKGLRTVILHGFQLLLCLIQLWGPFIESTLLR-FDF--LFINVRYSNYVLFNLTPRCLSPLIYGLRDEAFFHALKNE
LOR103               SGEDKKSSWKGLRTVILHGFQLLLCLIQLWSPFIESTLLR-FDF--LFINVRYSNYVLFNLTPRCLSPLIYGLRDETFFHALKNE
LOR104               SGEDKKSSWKGLRTVILHGFQLLLCLIQLWSPFIESTLLQ-FDF--LFINVRYSKYVLFNLTPRCLSPLIYGLRDETFFHALKNE
LOR105               SGEDKKSSWKGLRTVILHGFQLLLCLIQLWTPFIESTLLR-FDL--LFAHVRLSNFILFGLTPKCLSPLIYGLRDETFFHALKNE
LOR106               SGEDKKSSWKGLRTVILHGFQPLLCLIQLWSPFIESTLLR-FDF--LFINVRYSNYVLFNLTPRCLSPLIYGLRDETFFHALKNE
LOR107               SGEDKKSLWKGLRTVILHGFQLLLCLIQMWCPFIEAAVFQ-IDL--LFINVRFYNYVLFNLTPRCLSPLIYGLRDETFFHALKNE
LOR108               SGEDKKSSWKGLRTVILHGFQLLLCLIQLWTPFIESTLLH-FDL--LFAYVRVSNLILFGLTPRCLSPLIYGLRDETFFHALKNE
LOR109               SGEDKKSSWKGLRTVILHGFQLLLCLIQLWTPFIESTLLQ-FDL--LFFHVRLSNFILFGLTPKCLSPLIYGLRDETFFHALKNE
LOR110               SGEDKKSSWKGLRTVILHGFQLLLCLIQLWTPFIESTLLR-FHL--LFTHVRLSNFILFGLTPKCLSPLIYGLRDETFFHALKNE
LOR111               SGEDKKSSWKGLKTVILHGFQLLLCLIQLWTPFIESTLLR-FDL--LFAHVRLSNFILFGLTPKCLSPLIYGLRDETFFHALKNE
Fugu_OR123-1         -QKCLG--SKESRTKFMQTCLPHLLCLLTMVICMLFDLLYMRFGTKLPESIQNFIAIQFILIPPILNPLIYGFKLQKIRRRIQYF
Fugu_OR6765-1        -KTCLT--SKEDRRKFIQTCLPHVSSLIVVIVCLLFDLVHMRFDSGLSDNARNFMAIQFLLFPPLINPLIYGIKLTPIRNRIQNF
Fugu_OR4133-1        -KTCLT--RKEERMKFMQTCLPHLSSLIIAKICLLFDLLHMRFDSGLSEGARNFMAIQFLLFPPLINPLIYGIKLTPIRNRIQSF
Fugu_OR3630-2        -LISYQS-SKEVRKKAAQTCLPHLMILINFSCLTVYDVLLLRLNTVVPKTVHFLITLQIIMYHPLFNPIVYGLKMKEISKQLKKL
Fugu_OR8617-1        -LISYQS-SKEVRKKAAQTCLPHLMILINFSCLTVYDVLLLRLNTVVPKTVHFLITLQIIMYHPLFNPIVYGFKMKEISKQLKKL
Fugu_OR5510-1        -LISYQS-SKEVRKKAAQTCLPHLMILINFSCLTVYDVLLLRLNTVVPKTVHFLITLQIIMYHPLFNPIVYGFKMKEISKRLKKL
Fugu_OR117-1         -FICRKC-SSDFKSKVVHSCLPHIITFVNYSVTVFCDVALSRIDLELNPFIAVVLSLEFVVIPPLVNPLVYGLKLPEIRKCILRM
Fugu_OR2346-5        -ATCWKG-SAGFNRRVFQSCVPHIISFVINSLTVFCDIALSRNNIEINPFLAVIFSLEFVVIPPVLNPLVYGLKLEQIRREILRL
Fugu_OR5287-2        SVVSRASVGSEDRWKAFHTCGTHLMVMMCFYLVGSVTFLSHNLNINIPADVNTFMGVMYILFPATVNPIIYGVRTTEIRNGLLKI
Fugu_OR142-1         -IACMVSRRSEAKMKAINTCVAQLMILAIFEFVATFTILSHRFNT-VSADLQKIMGMLIFLIPPLLNPIVYGLYTGEIRNALLR-
Fugu_OR1026-2        -IVCVTSNNKSVNSKALKTCSTHLVMYLIMSASGILIIALHRFP--QYSDYRKICAILFHTIPGSMNPIIYGIQSKEIQKYLYKL
Fugu_OR8298-1        -IVCVTSNNKSVNGKALKTCSTHLLMYLIMFASGILVIALHRVP--QYSDYRKICAILFHIIPGSLNPIIYGVQSKEIQKFLLRQ
Fugu_OR6030-1        S-TDKTSSQKARNTLLLHLVQLGLSLSSTVYKPILAALSRIVSR--VLVRLQNVLYVCLFILPRCLSALIYGIRDQLIRPVLFYL
Fugu_OR59-1          S-TDKTSSQKARNTLLLHLVQLGLSLSSTVYKPILAALSRIVTR--VLIRVQNVLYVCFFILPRCLSALIYGIRDQLIRPVLFYL
Fugu_OR4208-3        SKD----ARKARNTIILHGFQVLLCMATYAEPLLKQALLLWFPR--NYSDSLFACYIIVQILPRSISPIIYGVRDKTFRRHLTRL
Fugu_OR4208-2        GKD----TVKARNTIILHSFQLLLCMASYLAPALRELLQKLFPK--NSTDILFVSYIVVQVLPRSTSPMIYGLRDPTFRKYLKRM
Fugu_OR6818-1        SGENKKSTWKGLRTVILHAFQLLLSLIQLWCPFIESAIFQ-IDY--LFINVRYFNYVTFILAPRCLSPLIYGLRDEKFFNALKYA
Fugu_OR7903-1        SGENKKSTWKGLSTVILHAFQLLLCLIQLWCPFIESAIIK-IDL--LYINVRYFNYVTFILAPRCLSPLIYGLRDEKFLNALKYA
Pufferfish_OR8981-2  -KTCLT--SKDDRIKFLQTCLPHLMSFIVMSMCLLFDILHEGFSSEIPESARNFIAIQFLLVPPLINPLIYGLKLNKVRNRINRF
Pufferfish_SCAF8981  -KTCLS--SKDDRIKFLQTCLPHLMSFIVVNMCLLFDMLYEKLSTEIPKSTQNFIAIEFLFFPPLINPLIYGLKLNKVRNRIEKF
Pufferfish_OR15134-1 -LVSYQS-SREVRRKAAQTCLPHLLILINFSSFGIYDVLLLRLNTTVPKTVRFLITLQMIMYQPLFNPIIYGLKMREISKHLKKL
Pufferfish_OR14339-2 -LICRNR-SSDFKRKVVQSCLPHIITFVIYSITVFCNVALSRIDLELNPYIAVILSLEFVVIPPLVNPLVYGLKLPEIRKCVLRM
Pufferfish_OR14328-1 -LICRKR-SSDFKRKVVQSCLPHIITFVNYSINFFCDVALSRIDLELNPYIAVILSLEFVVIPPLVNPLVYGLKLPEIRKCILRM
Pufferfish_OR14677-8 SVVSRASVASEDRWKAFHTCGTHLMVMMCFYLVGSVTFLSRNLNIYIPADVNTFMGVMYILFPATVNPIIYGVRTTEIRNGFFKT
Pufferfish_OR14536-2 S-TDKTTSQKARHTLLLHLVQLGLSLSSTLYTPILTALSRTVIR--VLVRVQIVLYVWFFILPRCLSALVYGIRDRTIRPVFFYL
Pufferfish_OR14536-1 S-TNKTTSQKARHTLLLHLVQLGLSLSSTMYTPILTALSRTVIR--VLVRVQIVLYVWFFILPRCLSALIYGIRDRTIRPVFFYL
Pufferfish_OR10960-2 SGD----SRKARRTIVLHGVQVLLCMATYAEPLLKQALQLWFPR--SYSDSLFACYIIVQILPRSISPIIYGVRDQTFRRYLRRL
Pufferfish_OR10960-3 SKD----AKKARNTIVLHSFQLLLCMAAYLTPALRDALQRRFPE--NSVDVLFASYVLVQILPRSLSPLVYSVRDQTFRRYLRRL
Pufferfish_OR12434-1 SEETKTSTFKGLKTVILHSFQLLLCLIQLWCPFIESVVFE-NDK--IFSYVRFFNYVVFYIAPRCLSPLIYGLRDEKIFLALKVV
Medaka_ORUn.33       RVVLRAAR--EDRSKAFHTCSTHLMVIMCFYLVGSVAFLSRDL----SSGVGAFMGVLYILLPATVNPIIYGIRTKEIRNGLLRS
Medaka_ORUn.2        STVLSSAR---SGGKALHTCVTHIMVLSLSLTVALVAFLSYRVRNSLPTAVRVFFSTMYLLFPSCVNPIIYGIRTTEIRQHIMK-
Medaka_OR21.18       -RSALK--SREGKGRFVQTCVPHFLCVLNVTASLLFDIMYSRYGSALPQTLKNFMAIQFLLGPPLLNPVVYGLILTKIRKRMIRV
Medaka_OR21.19       -KTCMN--SIENRAKFMQTCVPHLVSLITFLVTILFDLMNMRLTSKLDQIFQNFIAMEFLLIPPIMNPLIYGFKLTKIQRKIAKL
Medaka_OR21.20       -KTCMN--SIENRAKFMQTCVPHLVSLITFMVTILTDVISLRLGFDMPQTLQNLIAIEFLLIPPIMNPLIYGFKLTKIRYKIIKL
Medaka_OR13.14       SGENKKSTWKGLRTVVLHGFQLFLSLIQLWSVLIEDAAFK-VSL--LYNNIRYFNYVMFFLAPRCLSPFIYGLRDELFFQALKYA
Medaka_OR13.12       SGENKKSTRKGLRTVTLHGFQLQLGLIQLWCPFIEAAVYN-VDP--VYNNLRFFNYIVFFLAPRCLSPLIYGLRDELFFQALKYA
Medaka_ORUn.23       S-TDKALARKAQHTLLLHLVQLGLSLSSTINNPVIVALSKLLTR--AFLWIQGIFYIFFIIFPRCMTSLIYGLRDQTIRPVLMYL
Medaka_ORUn.21       S-TDKALARKARHTLLLHLVQLGLSLSSTIYNPLVIALSKVLTR--AFLWIQNIFYVTFIIFPRCMTSLIYGLRDQTIRSLLLDL
Medaka_OR14.15       S-TDKALARKARHTLLLHLVQLGLSLSSTIYNPLVVALSKVLTR--AFLWIQNIFYVTFIIFPRCMTSLIYGLRDQTIRSLLLDL
Medaka_OR14.13       -VVCLTSKNKSLNSKALKTCSTHLVAYFLMFACGMINILLHRFP--EYSYYRSITAFMFHILPGTLDPIIYGVQSKEIRHFLSKL
Medaka_OR14.4        -AVCLTSKSKSLNNKALQTCSTHLVVYLIMSISGVSIIVLHRFP--QYSEYRKLSAILFVMLPPSLNPIIYGVQSREIQKFLSEK
Medaka_OR14.6        -AVCLTSKSKSLNNKALQTCSTHLVVYLIMSFSGVSIVVLHRFP--QYSEYRKLSAILFVMIPPSLNPIIYGVQSREIQKFLSEK
Medaka_OR13.4        -IACFRSKQSDTRAKALQTCATHLVVFLLLECLGLFTIISHRLQN-ISPHLRRFMGLSTLIFPPTLNPIIYGLKTKEIREKILQ-
Medaka_OR13.5        -FTCVKTNNTNGRKKAIQTCSTHLVVFLVLQVNTTFTLISHRFEN-ASPFLRRALGVSVLIFPPFLDPLIYGLRTRELRQCMVV-
Medaka_OR13.6        -VTCMMTNNPDGRRKALQTCCTHVLVFLVLQINSNAAYILHRSQS-KLTVLRKAFGFSVILFPPFLDPLIYGLNTRELRQCIIM-
Medaka_OR13.7        -ITCLATRTSEAKTKAVHTCVAQLVILVIFEVVGTFTILSHRFKD-VSADLQKIMGMLIFLVPPLLNPLVYGLYTSEIRKTLLG-
Medaka_ORUn.1        -RTASR--SRSDRRKFLQTCVPHLAALLVFTGSLLFDGAFS-RYGGSIEMLQNALAAEFLVVPPALNPIIYGMNLKEVRSRIYFR
Medaka_OR21.2        -LVVNKG-CREVRRKAAQTCLPHLLVLINYSCLMIYDIIIIRLESNFSKTARFIMTLQMIMYNPLCNPFIYGLKMTEIYKHLKKL
Medaka_OR21.3        -IVVYKS-SRKVRGKAAQTCLPHLLVLINYSCLAIYDVTIVRVESNLSKTARFIMTLQLIMYTPLCNPIIYGLKMTEIYKHLRKL
Stickleback_OR1.1    DAALRAATASEDRRKAFHTCGTHLMVMMCFYLVGSVTFLSHNLNIAIPTNVNTLMGVAYILLPATINPVIYGVRTKEIRSGFLK-
Stickleback_OR16.3   -KICVN--SLENRAKFMQTCVPHLACLLIFLGTILLDFMHIRYGSNLPQAFKNFIAIEYLVILPIMNPLIYGFKLTNIRKRIVAV
Stickleback_OR1.12   SGEDKKSTRKGLKTVIFHAFQLSLCLIQLWCPLIEAAVLQ-INL--LFINVRYFDYITFILAPRCISPLVYGLRDEMFFHALKYA
Stickleback_OR1.13   SGEDKKSTGKGLKTVILHAFQLSLCLFQLWCPFIETAVLQ-INF--LYINVRYFDYIAFILAPRCISPLVYGLRDEMFFQALKYA
Stickleback_ORUn.9   S-SGGASARRARDTLLLHLVQVGLSLSSTVYSPVLMALSRSVNR--LFVRLQNVLYVAIFIFPRCLSSLIYGLRDKSIQPVLLQL
Stickleback_OR7.1    S-STVEKSSKPHKTMLLHLIQLCLCVTSTLFYMINPINQWKGNR--MAIHIQYGLFLVLIILPKCLSPLIYGLRDQAFRHVFEYF
Stickleback_ORUn.6   -VVCLTSNSKSLNSKALKTCSTHLVVFLIVVLSALSNIVLHRFP--QYSDYRKLSSILFHIVPGSLNPIIYGVQSKEVQHFLFKW
Stickleback_ORUn.8   -VVCLTSNSKSLNSKALKTCSTHLVVYLIMLISGMCIIVLHRFP--QYSDYRKLSAILFLIVPGSLNPIIYGMQSAEIGKALSKL
Stickleback_OR1.18   -ITCMVTRRSEAKTKAVNTCVAQLLILIIFEIVSTFTILSHRFKN-VSADLQKIMGMLIFLVPPLLNPIVYGLYTSEIRNTLLT-
Stickleback_OR16.10  -LNCRKR-SSEFKSKVIQNCLPHIVTFVNYSITVFCDVVLSRIDLELNPFLAIILSLEFVVIPPMVNPLVYGLKLAEIRKCVLKV
Stickleback_OR16.32  -STCRK--SKENMSKFMQTCVPHLFSLIIVVASLLFDLLYMRFGSKLPQGAQNVMAMEFLLIPPIINPLIYGLKLTKIRNSIQNV
Stickleback_OR16.1   -LICRKR-SSEFKRKVIQNCLPHIVTFVIYSITVFCDVALSRIDLELNPFLAIILSLEFVVIPPIVNPLVYGLKLAEIRKCVLNV
Stickleback_OR16.5   -LICRKR-SSEFKSKVVQNCLPHIVTFVIYSSTVFCDVALSRIDLELNPFLAIILALEFVVIPPMVNPLVYGLKLAEIRKCVLKV
Stickleback_ORUn.1   -VITYRS-CGEVRMKVAQTCLPHLLVLINFCSLVTYDVIAVKLESEIPKAIRFIMTLQVILYHPLLNPIIYGLKMTEISKHIRRL
Stickleback_ORUn.17  -VVSYR--CRGVRRRAAQTCLPHLLVLINFTCLISYSLILDQLELDSPKTVRFIITIQAVIYQPLFNPLIYGLKMRRIYDHLKKL
Zebrafish_OR126-7    -RKCVK--STEGRRKFMQTCVPHLLSLINVTVALLFDVLYSRFGSKVPHSVRNFMALEFLLIPPILNPLIYGLNLTIVRQQVIKL
Zebrafish_or126-3    -RKCQK--SIESRHKFMQTCVPHMISLLNVTVAFLFDVLFSRYGSKVSQNLRNFMALEFILIPPFLNPLIYGLNLTAVRQEVIRL
Zebrafish_OR123-1    -VACKH--SVQNHKKFISTCLPHLIAFLNYVFWSLFSTLYELFGISLSQEFKNFLSVTFMIIPPLINPVIYGIVLTPIRTKAKHV
Zebrafish_OR125-1    -AACKA--SLENRKKFWQTCLPHIFSLINFTFAFLFDFMYNRYGSKIPESLRHFLALELVIVPPLFNPLMYGLNIRAVRKRVFTS
Zebrafish_OR127-1    -IICQKS-SKEFKGKALQTCLPHIVSFVNYSIASFCDIALSRNDSDIK-ILTIIFSVEFLVIPPVLNPLIYGLNLPEIRKKIACL
Zebrafish_OR128-10   -IICRKS-SPEFRRKAYQTCIPHIVILLNFSAALFCEVTLSRVQNLLPIGLSIILSLEFLIVPPILNPLIYGLNFPEIRKKIMCI
Zebrafish_OR116-2    -ITCLATSKSDAKNKAINTCVSQLVIFIMFEIVATFTILSHRFTS-VNADLQKIMGMLIFLVPPLLNPIVYGLNSHELRNNLLK-
Zebrafish_zor6a      HVVL-GTAAGEDRWKAFHTCGTHLMVMICFYFVGSVTFLSRNLNIPIPIDVNTFLGVMYIVFPASVNPIIYGVRTKEIRTALFKS
Zebrafish_OR117-1    -ITCVKQTHADSRVKALNTCVAQFTTFILFEMVTAVAIISYRIPD-FPPTAQRVCGLMIYAVLPVVNPVIYGLKMKDIRIALFV-
Zebrafish_OR115-15   -AVCLVTKSKMLNSRALKTCSTHLSLYLIMLISGLIIIVLHRFP--AYSDYRKIASLLFHIIPSILNPIIYGVQSSEVRHVLIQS
Zebrafish_OR133-7    A-CDKLSAKKANKTVLLHLIQLGLGASSILVGVIQEAIYVYTDF--TALNVMYFCFLVFIIFPKCLSPLIYGMRDQAFCCLFKYF
Zebrafish_OR132-1    S-ADKESAKKALKTVMLHLIQLGLCLTSFLYATIERTLYMVIGSGSLFINLRYLNYIIVLILPRCLSPLIYGMRDEAVWPLFKYV
Zebrafish_OR134-1    STENTS-AKRATNTILLHGLQLIMCLLSYISPSVEGILNIIFPG--RVLEIRFANYLIVYILPRFLSPIIYGVRDKKFRKYLRRF
Zebrafish_OR131-2    SAENKKSTYKGLRTVILHGLQLILGMMQLITPYIDILTLK-VDI--LFINVKFSNFMLFWIFPRCLSPLVYGLRDKKFYNALKYA
```

```
Zebrafish_OR131-1        SATNKKSTYKGLRTVILHGIQLILGMMQFITPYTEMSLWK-IDV--LFLNVRYSNFILFWILPRSLSPLVYGLRDKKFYYALKYA
Goldfish_OR3             -GKCRK--SKEGRNKFIQTCVPHLLALLNVSFALLFDVLYTRYGSMMPQDLRNFMALEFLLVPPMLNPLIYGLNLTKLRKEVLRL
Goldfish_OR2             -VAVLHISSTQGRLKSFSTCSPQLIIIALYFLPRCFIYLSS-NIGIFSTDLRLVIIMMYSLFPPMINPLIYCLKTKDVKETLLKK
Rainbow_trout_OR500-1    -RTCVK--SAKGRIKFTQTCVPHLITIFIFITVTLFDNLQG-WNNVSTLNMRNAMAVQFLVIPPVFNPLIYGLNLQQIRRAVFRK
Cutthroat_trout_OR600-1  -IVCLSSKNSSLNSKALTTCSTHLLVYLIVLGCGFTIIVLHRFP--AFADLRKVSSVLGSVVPTCLNPIIYGLQTKEIKQRIFRV
Brown_trout_OR500-1      -RTCVK--SAKGRIKFTQTCVAHLITIFMFITVTLFDNLQG-WNNVITLNMRNAMAIQFLVIPPVFNPVIYGLNLKQIRRAVFRK
Atlantic_salmon_SORB     -AECFISKNKAMNSKALKTCSTHLTVYLIMLISGSIIIILHRFP--HYSDYRKVAALLFHIIPGSLNPIIYTLRNPTHRHVPVI-
Medaka_mfOR2             -KISYRS-SKEVRSKAAQTCLPHMLVLVNFSCLGSFEIIIGFFWSEYPKTLHLTVILQIIIYQPLFNPIIYGLKMKEISKHVKML
Medaka_mfOR1             -KISYRS-SKEVRSKAAQTCLPHLLVLVNFSCLSSFDVIIGFFGSGYPKTLHLTVMLQIIIYQPLFNPIIYGLKMKEISKHVKML
Medaka_OR_Y3             -VVCLTSKNKSLNRKALKTCSTHLFVYMVMISCGLLNIVLHRFP--EYSDQRKLIVILFHIIPGSLNPIIYGLQSKEVQRFFRSA
Medaka_ORY1              -AVCLTSKSKSLNNKALQTCSTHLVVYLIMSISGVSIIVLHRFP--QYSEYRKLSTILFVMLPPSLNPIIYGVQSREIQKFLSEK
Frog_OR-52D1             -RVCMKA-SKAFRAKALQTCTPHLVTLTYFVADVLFEILLPRFPSTLPYELRVLMSVQAFVIAPILHPLIYGWKLREIRLRVLQM
Frog_OR-52E8             -RVCMKS-SKDVRAKALQTCTPHIVSLLYFVVDILCEVVLLRFPFNIPYELRIIISVQAFVFAPLLNPLMYGLKLRKIRVKIGQI
Frog_OR-52A1             YTAMKSATG-KSQHKALNTCGTHLLVAMVVYLCGLASSIVYRMETTISPDVKNLFSAIYLMVPATLNPFIYGLRVSEIRKSLMKP
Frog_OR-52P1             YTAMKTATG-KSRHKALNTCGTHLLVAMVVYLCALASTIVQRMETSISTDIKNLFIALYLIIPALLNPFIYGFRVSEIRKSLLGS
Frog_OR-52A5             DSGKES-AIKASRTVMLHAIQLFLCLMAFSN----NLLEIYLME--YMYLLPLGSFFLFMCLPRFISPLIYGIRDEVFRNYIKRM
Frog_OR-2M5              GSGTSS-AIKAGKTVLLHGVQLLLSLMSLIT----SYIEANVKE--YVVLLAISNFLFFMCLPRFLGPLIYGLRDEVFGKHLR--
Frog_OR-2M2              -RVIHS-TNTENSWKAFYTCSTHLMVIGLYFIPRVFVYSTS-QIPLLDADINVLLLCLYTFIPHLANPVIYCLRTKDIRNIFAQS
zebrafish_OR10.1         -RAVFRMKSSQSRFKALATCTEHLVLVCLYFVPIVIIFNLT-FFGIWSPNVGLVCLSLSSLLPPCVNPVIYSLKTKEIRSRVYSL
zebrafish_OR10.14        -VTLFRVTTPQDRQRATKTCTAHLILVAVFYLPITITYS----LASINTNTRIINLSLTSALPPMLNPIIYTFKTEEFMVSVKKL
zebrafish_OR15.39        -IAVMRIASVTQRWKAFHTCLTHMMLVMLYYMPVIIAYVLG-NLRLQNVDLLTAILTVSVTVPAMLNPIIYSLKTDELKERMVKL
Medaka_ORUn.6            -VTVFRMKTSDNRRKTLATCAEHIILVAIFYIPFFVIYTMG-FYLGVNPDLRVLSLSMASCLPPCINPVVYSLKTKELKTRALAL
Medaka_ORUn.28           -FALFKVSTIQEGVKAFKTCTAHLSLVAIYFFPILITFT----NTNIHPNHRIINLSLTAVFPPMLNPIIYVLQTQEIKASFKKV
Medaka_ORUn.27           -YTLVKVATLQQGIKAFKTCLAHLSLVVVYFTPVLITFT----LMDIHPNNRIINLSLTSVFPPMLNPIIYVLQTKEIQTSLKKI
Stickleback_OR7.3        -ATVFRMKSVSNRMKALATCIEHLILVAIFYIPIFTIFLMG-LYVRIDPDQRVLSLSLATCIPPCINPIVYSLKTREIKTRALAL
Stickleback_OR7.4        -VTVFRMKSLDSRVKALATCTEHVILVAVFYVPIITIFTLG-FFLRTDPDQRVLSLSLASCIPPCINPIVYSLKTKEIKMRAVAL
Stickleback_OR7.8        -CTLAKVATVQEGLKAFRTCTAHLSLVAIYFIPVLITFT----LMEINPNARIMNLSLTSILPPTLNPIIYVLQTQEIKKSVKKL
Fugu_ORUn.83             -CTLAKVATVQQGLKAFRTCIGHLLLVAIYFIPLLITFT----LMEIHPNARIINLSLTSVFPPMLNPIIYVLQTREIKDSLKRL
Fugu_ORUn.20             -VTLFLMKSVNSRMKALGTCVEHLVLVAIFYIPLFTIFILG-FYVRIDADQRVLSLSLASCIPPCVNPIVYSLKTREIKIRAVAL
Pufferfish_OR7.1         -CTLAKVANFQRGLKALKTCTGHLLLVAIYFIPLLITFT----LMEIHPNARIINLSLTSVFPPMLNPIIYVLQTQEIKDSLRRL
Pufferfish_OR7.3         -LTLFRMKSVNSRMKALSTCVEHLVLVAVFYIPLFTIFIFG-FYVRIDADLRVLSLSMASCIPPCINPIVYSLKTKEIKIRAVAL
zebrafish_OR10.29        -ASVMRLSSSQSRWKSFATCSTQLCIITLFYMPRFTVYLTT-FLQIISKDFRILLVLIYCLVPPLVNPFIYCLRTQEIRMIVSRW
zebrafish_OR10.31        -VSVLQIANAKGRLKALSTCATQLTIISIYYVPRFAVYITSNVPNAMDKAEKIALVMFYSLLPPLMNPFIYFIRIREIRQVFLKC
zebrafish_OR15.58        -ISVSKISKSEGRYKTFSTCTPQLLIICLYYLPRTFVYITN-ISGYLSNDTRMVVSMMYSLIPAVINPFIYCFRTKEIKEAIFKR
zebrafish_OR15.57        -ISVFRISDTQARFKTFSTCTPQLLIICLYYVPRCVVYVHD-VT-IISPGIRVMLIMWYTLIPPIVNPMIYCFRTKEIKDAIKKK
Medaka_OR13.1            -VAVLKMTKAEGYHKVLSTCAPQILTTCLYYVPRCVVYITD-NLKVVSPDARIIIALLYSLIPAAVNPLIYCLKTADIKEALMKR
Medaka_OR13.3            -VVILKMSSSSGRKKTISTCTPQIFITCLFYLPRCFVYVAS-TVGFFSLDIRILLILLYSLLPPALNPLIYCFKTRDIKLTLLRK
Stickleback_OR1.25       -ADILKMSNATGRMRTISTCTPQILITCLFYLPRCFVYVSN-TVGYFSLDVRILVVLLYSLLPAAVNPIIYCFKTQYIKQHLIKK
Fugu_ORUn.8              -VVIAKMSSTAGRRKTLSTCTPQLFVTCLFYLPRCFVYVAN-TVGFFSIDVRILLTLLYSLVPPAVNPFIYCFKTQDIKKILMKK
Pufferfish_OR16.7        -VVIMRRSNGAGRRKTLSTCTPQLFITCLFYLPRCFVYVAN-TVGFFSVDVRILLVLLYSLVPPAVNPFIYCFKTQDIKQTLIKK
Frog_OR669.1             -KAICSRDHFENWRKTFYTCATHLLVIGLYFIPRIFVYISN-QVQLLEEDLNVLLLCLYTFVPHMANPIIYCLRTKEIRKTIARF
Amphioxus_105.1          QERDEQRPVKFYKTRGFKTMAPLAILLTTTIVSTLFMTVTVAQGNAAVVILEKASILLNLTLSSMLNPIIYSLRLPEFRRAMREV
Amphioxus_39.1           -ERDEHRNLWLYQTKAFKTLVPHIVVLVVSVASYVFLVASGRALLNSLLITVIVANRIFLTLSSMVNPIVYSFRHPEFRQALGEV
```
